# Spin-coordinated mixed-valence transport via indium-induced perovskite-spinel heterointerface in oxygen electrode for reversible protonic ceramic cells

**DOI:** 10.1038/s41467-026-75684-4

**Published:** 2026-07-15

**Authors:** Yinlin Chang, Tao Liu, Jingyuan Rao, Shuanglin Zheng, Min Fu, Hanping Ding, Zetian Tao

**Affiliations:** 1 School of Resources, Environment and Safety Engineering, University of South China, Hengyang, China; 2 School of Aerospace and Mechanical Engineering, University of Oklahoma, Norman, OK USA

**Keywords:** Materials for energy and catalysis, Fuel cells, Chemical engineering

## Abstract

Achieving efficient oxygen electrocatalysis in protonic ceramic electrochemical cells requires more than accelerating surface reactions, which demands precise coordination of electronic and spin states across the bulk-interface continuum. Conventional strategies based on vacancy engineering or catalyst infiltration increase active sites, yet they do not intrinsically couple bulk charge transport with interfacial redox kinetics. Here, we introduce spin-coordinated heterointerface engineering as a general design principle, exemplified by In-induced self-assembly of a Co_3_O_4_ spinel layer on Pr_0.5_Ba_0.5_Co_0.7_Fe_0.3_O_3-δ_ (PBCF), forming PBCFIn05. Indium incorporation triggers selective Co migration and interfacial spinel reconstruction, which in turn drives a bulk spin-valence reconfiguration from predominantly high-spin Co^3^⁺ to mixed-spin Co^3^⁺/Co^4^⁺. This evolution establishes complementary double-exchange conduction in the perovskite bulk and small-polaron hopping within the Co_3_O_4_ spinel, creating a continuous mixed-valence pathway that synergistically lowers the activation barriers for both oxygen reduction and oxygen evolution. These results demonstrate that self-assembled spin-active heterointerfaces provide a powerful route to overcome intrinsic spin-selection bottlenecks in ceramic electrocatalysis and offer a broadly applicable platform for advanced solid-state energy conversion systems.

## Abstract (web summary)

Oxygen electrodes in reversible protonic ceramic cells require coordinated charge and spin transport across the bulk and surface. Here, the authors report that indium-induced cobalt oxide interfaces regulate mixed-valence pathways and support reversible cell operation.

## Introduction

Global efforts to achieve carbon neutrality by mid-century demand disruptive advances in electrochemical energy technologies that can efficiently couple renewable electricity with chemical energy carriers. Proton-conducting ceramic fuel cells (PCFCs), particularly their reversible form, reversible protonic ceramic electrochemical cells (R-PCEC), are gaining increasing attention for their high efficiency, moderate operating temperatures, and near-zero carbon emissions^1–4^. Compared to oxygen-ion-conducting solid oxide cells, R-PCECs exhibit lower proton migration barriers, faster electrode kinetics, and higher round-trip efficiencies^5,6^, positioning them as promising building blocks for low-carbon energy infrastructures.

Perovskite oxides, such as Pr_0.5_Ba_0.5_Co_0.7_Fe_0.3_O_3 − δ_ (PBCF), offer high electronic conductivity and mixed ionic transport; however, their activity is often constrained by rigid lattice coordination and limited adaptability in accommodating dynamic redox states during cycling^7–9^. To overcome these challenges, numerous studies have sought to enhance the interaction between oxygen intermediates and active sites through elemental doping, nano-structuring, and interfacial engineering to expand the triple-phase boundary (TPB). For instance, selective doping in perovskites has been employed to introduce oxygen vacancies and modulate reaction sites^10,11^; composite air electrodes incorporating heterophases have been designed to enlarge the TPB area^12,13^; and catalyst infiltration into fuel electrode scaffolds has been used to increase the density of surface-active sites^14,15^. These strategies improve surface adsorption and expand active sites; however, their effects are largely confined to local reactivity and fail to govern the deeper electronic framework that controls charge transport. In particular, most approaches neglect the critical role of spin selection, namely that electron transfer during oxygen redox must be accompanied by spin-state matching between adsorbates and transition-metal cations. Without properly aligned spin configurations, even structurally favorable interfaces can suffer from intrinsic kinetic penalties that limit overall electrocatalysis efficiency.

Recent studies have underscored the importance of spin-state tuning in cobalt- and iron-based oxides. Reversible transitions between high- and low-spin configurations modify the occupancy of antibonding, *e*_g_ orbitals and the degree of metal-oxygen covalency, thereby reshaping electron-polaron hopping and oxygen-activation pathways. These changes, in turn, dictate the adsorption strengths and reaction barriers of key ORR/OER intermediates^16–18^. In essence, two descriptors, antibonding, *e*_*g*_ occupancy and M–O covalency, govern intermediate binding energies and kinetic barriers in oxygen redox reactions. This principle has been exploited in various systems: long-range spin ordering induced by external magnetic fields has enhanced OER kinetics in NiZnFeO_*x*_^19^; tailored spin configurations in bimetallic ferromagnetic clusters have promoted favorable O–O intermediate binding and broken conventional scaling relationships^20^; Jahn–Teller distortion in Cu–Ni–Fe layered double hydroxides has optimized Fe^3+^ spin states, accelerating the OH^*^–O_2_ conversion^21^; and Ni doping in Sr_2_Fe_1.3_Ni_0.2_Mo_0.5_O_6_ has induced high-spin Fe^4+^ states via double exchange, improving conductivity and CO_2_ adsorption^8^. In parallel, heterostructure engineering provides nanoscale control of electronic structure and spin population through interfacial coupling and lattice distortion. Such architectures often involve topological rearrangements of A or B-site cations, generating composition gradients and localized structural perturbations that promote defect formation and facilitate ion transport^22–24^. Notably, perovskites with, *e*_*g*_ occupancy approaching unity and strong M–O covalency exhibit improved OER activity, while O_2_-mediated spin transitions between neighboring cations can further accelerate electron transfer and intermediate activation^25–27^. Despite these insights, deliberate integration of spin regulation with interfacial charge transfer remains underexplored. Most vacancy- or strain-driven approaches modulate either bulk electronic transport or surface redox individually, failing to establish a continuous pathway that couples spin dynamics across the bulk-interface boundary. Therefore, a strategy capable of synchronously engineering bulk spin states and interfacial electronic pathways is essential to unlock high-performance oxygen electrocatalysis in PCECs.

To overcome these constraints, an ideal oxygen electrode design should not only enhance surface reactivity but also construct a coherent electronic pathway that bridges bulk charge transport with interfacial redox kinetics. Such an approach enables electrons and lattice ions to move cooperatively while maintaining favorable spin configurations throughout the reaction pathway. Achieving this requires a mechanism that can regulate both bulk spin-state distribution and surface charge-transfer flexibility, rather than treating them as independent domains. One promising direction lies in self-emergent interfacial architectures, where the functional surface layer is not externally deposited but evolves spontaneously from the parent lattice through controlled phase reconstruction. When properly regulated, such reconstructions can yield a spin-active interface that operates synergistically with the underlying perovskite framework. Crucially, this interface must not only provide abundant redox-active sites but also accommodate small-polaron hopping and spin-selective charge transport, enabling low-barrier electron exchange during both oxygen reduction and evolution. Despite growing recognition of the importance of spin-coordinated pathways in transition metal oxides, a fully integrated system that synchronizes bulk spin-state modulation with interfacial electron transport has yet to be realized. Existing modifications often enhance either ionic conductivity or surface activity, but rarely achieve continuous spin-aligned transport extending from the lattice interior to the active interface. Establishing such a coupled transport network represents a critical step toward efficient and durable oxygen electrocatalysis in ceramic systems.

In this work, we demonstrate a practical realization of this concept through spin-coordinated heterointerface engineering in an Pr_0.5_Ba_0.5_Co_0.7_Fe_0.3_O_3 − δ_ (PBCFIn0.5) with excess indium incorporation. Controlled indium incorporation induces selective cobalt migration and spontaneous reconstruction of a conformal Co_3_O_4_ spinel layer at the surface while simultaneously reconfiguring the bulk lattice into a mixed-spin Co^3+^/Co^4+^ state. This self-assembled interface and spin-regulated bulk cooperate to form a continuous electronic pathway that supports both double-exchange conduction and small-polaron hopping. These findings illustrate a viable route for synchronizing bulk electronic structure with interfacial redox functionality, offering a scalable framework for advancing oxygen electrocatalysis in protonic ceramic electrochemical systems.

## Results

### Spin-related mixed-valence transport

**Fig. 1:**
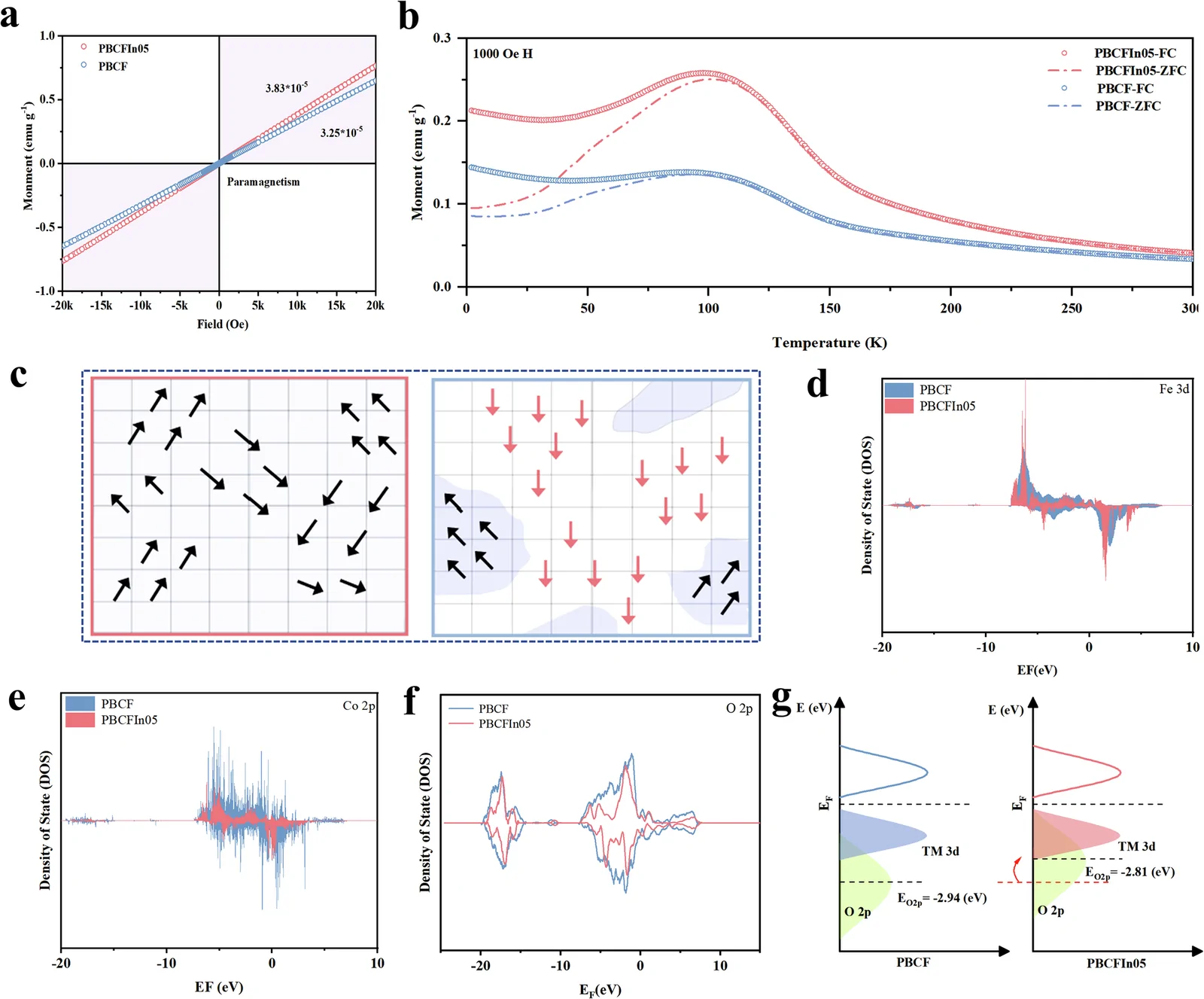
Spin-related mixed-valence transport across bulk–interface pathways. **a** Magnetic hysteresis loops of PBCF and PBCFIn05. **b** Zero-field-cooled (ZFC) and field-cooled (FC) magnetization curves of PBCF and PBCFIn05. **c** Schematic illustration of the competition between cluster spin-glass (CSG) and ferromagnetic (FM) phases. Black arrows represent randomly oriented spin clusters, while red arrows indicate collinearly aligned spins. The left panel shows the dominant CSG state in pristine PBCF, whereas the right panel depicts the coexistence of CSG and FM clusters in In-doped PBCFIn05. **d** Density of states (DOS) plots of the Fe 3*d* orbital. **e** DOS of Co 3*d* orbitals. **f** Schematic energy-level diagrams of O 2*p*. **g** DOS of O 2*p* states near the Fermi level. Source data for Fig.  1 are provided as a Source Data file.

Perovskite cobaltites remain among the most effective oxygen electrodes for PCECs, where long-range transport proceeds mainly through Fe-O-Co mixed-valence linkages, and surface exchange are governed by metal-oxygen covalency. To probe the interplay between lattice valence distribution and magnetic exchange, we examined a compositional series of Pr_0.5_Ba_0.5_Co_0.7_Fe_0.3_O_3 − δ_ (PBCF) and its excess indium incorporation variants, Pr_0.5_Ba_0.5_Co_0.7_Fe_0.3_O_3 − δ_–In_0.05_ (PBCFIn05) and Pr_0.5_Ba_0.5_Co_0.7_Fe_0.3_O_3 − δ_–In_0.1_ (PBCFIn10). The In^3+^ dopant was introduced at the B-site to redistribute the lattice valence and covalency, while simultaneously promoting the in situ formation of a self-assembled Co_3_O_4_ interfacial component. This dual modification aims to establish a more continuous pathway for bulk-to-interface charge and spin transport. To understand the spin correlations underlying the transport process, we first analyzed the magnetic fingerprints of PBCF and PBCFIn05 before evaluating their electrochemical performance. As shown in Fig. 1a, both materials exhibit nearly linear M–H curves, consistent with paramagnetic behavior; however, the steeper slope of PBCFIn05 indicates a larger population of unpaired spins, suggesting an enhanced mixed-valence state confirmed by spectroscopic evidence^8^. This was further confirmed by M-T measurements under zero-field-cooled (ZFC) and field-cooled (FC) conditions at H = 1000 Oe. Temperature-dependent magnetization (M-T) data (Fig. 1b and Supplementary Fig. 1) reveal that PBCFIn05 possesses a consistently higher magnetic susceptibility than pristine PBCF across the entire temperature range. Distinct magnetic anomalies appear near 92 and 99 K for PBCFIn05 and PBCF, respectively, corresponding to Néel temperatures associated with paramagnetic–antiferromagnetic transitions^28^. In the range of 250–300 K, the susceptibility follows the Curie–Weiss law, *χ* = *C*/(*T* − *θ*_*P*_), yielding effective magnetic moments (*μ*_*eff*_) of 8.3 *μ*_*B*_ for PBCFIn05 and 4.4 *μ*_*B*_ for PBCF, corresponding to approximately 5.7 and 2.43 unpaired electrons, respectively (see Supporting Information for calculation details, *μB*: Bohr magneton). The positive Weiss temperatures (*θ*_*P*_) indicate dominant ferromagnetic or ferrimagnetic exchange interactions. Collectively, these results demonstrate that In doping increases the Co^4+^/Co^3+^ fraction, enhances metal-oxygen covalency, and strengthens mixed-valence exchange interactions between Fe and Co ions.

Notably, both Co^4+^ (3*d*^5^) tend to stabilize in IS/HS configurations under octahedral fields, and Fe^3+^(3*d*^5^) typically adopts high-spin configurations, with theoretical magnetic moments of approximately 5.92 *μ*_*B*_, and are magnetically active in octahedral crystal fields. In cobaltite, LS-Co^3^⁺ (*t*_2g_^6^*e*_g_^0^) is commonly stabilized under strong octahedral fields and contributes little to the net moment, whereas Co^4+^ (3*d*^5^) and Fe^3+^ (3*d*^5^) provide unpaired spins and can participate in conduction. Rather than assigning specific Co spin states, we note that an increased Co^4+^ fraction together with stronger metal-oxygen covalency can raise the density of spin-active carriers, which could account for the larger *μ*_*eff*_ and positive *θ*_*P*_. In terms of magnetic coupling pathways, super exchange interactions between Fe^3+^-O^2–^-Fe^3+^ are generally antiferromagnetic and offer a limited net magnetic moment. By contrast, mixed-valence Fe^3+^-O^2–^-Co^4+^ interactions proceed predominantly via *e*_g_–*p*_σ_–*e*_g_ double exchange, which strongly favors ferromagnetic or ferrimagnetic coupling and serves as the primary mechanism for the enhanced *μ*_*eff*_^7^. Theoretically, assuming a 1:1 ratio of Fe^3^^+^ and Co^4+^ in the lattice, the expected *μ*_*eff*_ is approximately 8.37 *μ*_*B*_, which is in good agreement with the experimentally determined value, thus validating the proposed magnetic coupling model. Moreover, significant divergence between the ZFC and FC curves is observed below 130 K. The ZFC curve exhibits a typical peak, while the FC curve shows saturation at low temperatures, characteristic of a blocking phenomenon. Such behavior is suggestive of a cluster spin-glass state or the presence of short-range ferromagnetic clusters^29^. The corresponding schematic is shown in Fig. 1c. The increased Co^4+^ content in PBCFIn05 enhances the Fe^3+^-O-Co^4+^ double-exchange pathways, promoting the formation of local ferromagnetic clusters. These clusters, formed through interface-induced strain and oxygen-vacancy-driven coordination asymmetry, exhibit magnetic blocking at low temperatures, leading to a cluster-glass-like state superimposed on the predominant paramagnetic background. This mechanism is consistent with the observed high *μ*_*eff*_ and positive Weiss temperature *θ*_*P*_, confirming the emergence of strong magnetic coupling and local ordering induced by Co^4+^ enrichment.

Electronic-structure analyses further support this interpretation. Projected density of states (PDOS, Fig. 1d, e) shows that In doping induces asymmetry in the Co– and Fe–3*d* states around the Fermi level (*E*_F_), increasing, *e*_g_ orbital imbalance and generating spin-aligned, symmetry-matched *e*_g_–*p*_σ_–*e*_g_ acceptor states that reduce spin-mismatch penalties during Fe–Co hopping. Density-functional-theory (DFT) calculations (Fig. 1f, g) reveal that In^3+^ substitution shifts the O–2*p* band center upward from −2.53 eV to −2.39 eV, narrowing the energy gap between O–2*p* and transition-metal 3*d* orbitals. Notably, the obtained O–2*p* band-center values fall within the range reported in recent DFT+U studies on Co/Fe-based perovskite oxides, indicating consistency with the literature^30^. This upshift enhances M-3*d*-O–2*p* hybridization, signifying stronger Co/Fe–O covalency and improved lattice-oxygen mobility. The increased density of states near EF provides additional pathways for coupled electronic-ionic transport. Thus, spin-state selection not only reinforces super exchange interactions and local magnetic response but also reconstructs the electronic band structure, optimizing charge distribution and oxygen intermediate adsorption/desorption behavior. These synergistic effects underpin the enhanced electrocatalytic activity of the In-modified cobaltite electrodes.

### Indium-induced phase evolution and lattice reconstruction

**Fig. 2:**
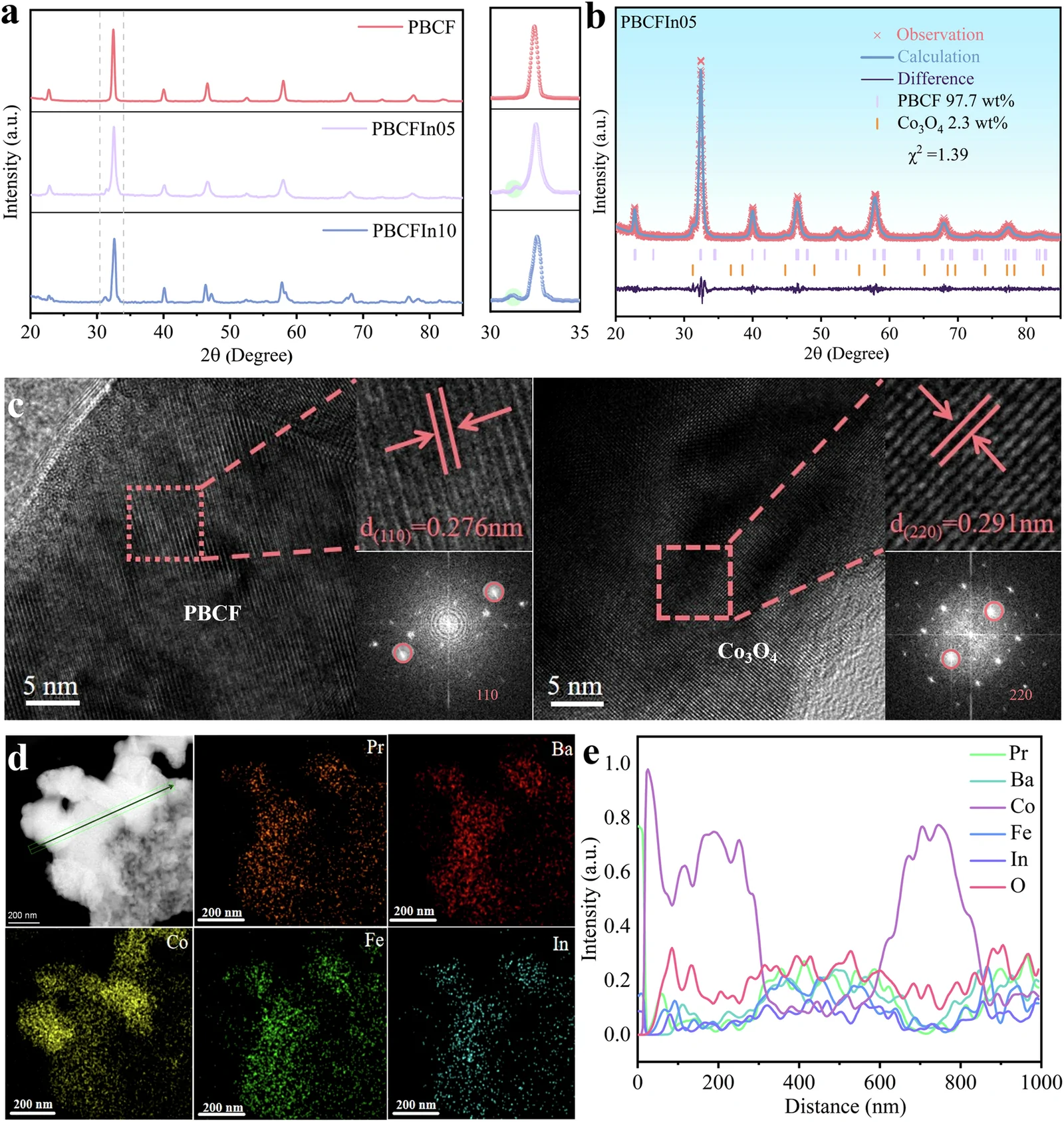
Phase composition and crystal structure analysis. **a** X-ray diffraction XRD patterns of PBCF, PBCFIn05, and PBCFIn05 powders. The right panel shows an enlarged view of the 2*θ* = 30–35° region, highlighting subtle changes in the main reflection (dashed-line reference) and a weak additional feature/shoulder (green) in the In-containing sample. **b** Rietveld refinement of as-synthesized PBCFIn05 powder. **c** HR–TEM images of PBCFIn05 and the corresponding lattice-fringe map. **d** Elemental mapping of PBCFIn05 obtained from energy-dispersive X-ray spectroscopy (EDS). **e** Line profiles of EDS intensity across the selected region in (**d**). Source data for Fig. 2 are provided as a Source Data file.

The X-ray diffraction (XRD) patterns of PBCF, PBCFIn05, and PBCFIn05 powders calcined at 1000 °C for 3 h are shown in Fig. 2a. Relative to the pristine PBCF, the In-doped samples exhibit an additional diffraction peak, indicating the emergence of a secondary phase. Rietveld refinements of the XRD data (Fig. 2b) reveal that PBCFIn05 consists primarily of a cubic perovskite PBCF phase (97.21%, *a* = *b* = *c* = 3.90 Å; space group Pm-3m) accompanied by a minor spinel Co_3_O_4_ phase (~2.79%, *a* = *b* = *c* = 8.10 Å, space group Fd-3m). The low refinement residuals (*R*_wp_ = 4.56, *R*_p_ = 3.61, and *R*_exp_ = 4.23) confirm the reliability of the fits. The detailed refinement results for all compositions are summarized in Supplementary Fig. 2 and Table 1. As the In concentration increases, the main perovskite reflections gradually shift to higher angles, signifying lattice contraction according to Bragg’s law (2*d* sin *θ* = *nλ*). The smaller ionic radius and lower electronegativity of In^3+^ relative to Co^3+^ perturb the Co-O-Co framework, inducing local bond-angle distortion and facilitating oxygen-vacancy formation. These vacancies relax the lattice and reduce the average interplanar spacing, consistent with the observed peak shift. Complementary XPS, TGA, and EPR analyses corroborate the increase in oxygen-vacancy concentration with higher In content. Furthermore, the emergence of a new diffraction peak around 31° provides additional evidence for Co_3_O_4_ precipitation. This secondary phase likely forms as nanoscale Co_3_O_4_-rich domains, consistent with the refined compositional analysis. Collectively, these results demonstrate that indium doping simultaneously drives lattice reconstruction and promotes surface spinel formation, laying the structural foundation for subsequent spin-coordinated interfacial evolution.

When In^3+^ excessively substitutes B-site Co ions, it perturbs the original Co-O-Co network, inducing local lattice and bond angle distortions. These structural perturbations modify crystal-field splitting and redistribute the electronic distribution within the B-site *d* orbitals. As the In concentration increases, accumulated distortions lead to substantial lattice strain^31^. To minimize free energy, the crystals relaxes this stress through cation redistribution and structural self-regulation. Under high-temperature conditions, part of the Co ions migrate toward the surface or grain boundaries, where they spontaneously segregate and crystallize as a Co_3_O_4_ phase. This “stress-assisted cation segregation” represents a self-driven phase separation process that relieves internal strain and lowers the overall lattice energy^32,33^. Similar mechanisms have been observed in doped BaFeO_3_, where lattice distortion promotes Fe segregation and BaFe_2_O_4_ secondary phase formation^22^.

From a thermodynamic perspective, the phase evolution associated with In incorporation can be expressed as:

Pr_0.5_Ba_0.5_Co_0.7_Fe_0.3_O_3_ + (*x*/2) In_2_O_3_ → (*x*/3) Co_3_O_4_ + Pr_0.5_Ba_0.5_Co_0.7 − x_In_x_Fe_0.3_O_3_ + (*x*/12) O_2_

Because O_2_ appears on the product side of the reaction, a lower oxygen partial pressure is expected to facilitate the formation of Co_3_O_4_. Under high-temperature calcination conditions, lattice strain induced by In substitution may further reduce the energetic stability of the homogeneous perovskite lattice, making partial cation redistribution thermodynamically accessible. To verify that the observed Co_3_O_4_ phase is intrinsic rather than an artifact of synthesis fluctuation, two independent re-syntheses of PBCFIn05 were performed. The resulting XRD patterns and Rietveld refinements show highly consistent phase fractions and lattice parameters within experimental uncertainty (Supplementary Fig. 3 and Tables 2, 3), confirming the reproducibility of spinel formation.

This behavior exemplifies a broader “structure–energy–reactivity” coupling, in which substitution-induced strain governs both the thermodynamic stability and reactivity of transition-metal oxides. In this system, In-induced Co_3_O_4_ precipitation, along with possible Ba-site defect formation, cooperatively modulates the electronic structure and oxygen-ion transport pathways, thereby enhancing its ORR activity under high-temperature air electrode conditions. To further validate the proposed mechanism, high-resolution transmission electron microscopy (HR-TEM) images of the PBCFIn05 (Fig. 2c) directly confirm this structural evolution.

Two distinct lattice fringes with spacings of 0.276 and 0.291 nm correspond to the (110) plane of PBCF and the (220) plane of Co_3_O_4_, respectively, verifying the coexistence of perovskite and spinel phases. The corresponding fast Fourier transform patterns further support these assignments. Scanning transmission electron microscopy (STEM) combined with energy-dispersive X-ray spectroscopy (EDX) mapping (Fig. 2d) shows homogeneous distributions of Pr, Ba, Fe, and In, indicating successful In incorporation into the PBCF lattice, whereas Co exhibits clear surface enrichment. Line-scan profiles (Fig. 2e) reveal two Co-intensity maxima at 0–200 nm and 600–800 nm, indicative of local Co-rich domains with slightly elevated oxygen signals in the same regions. Complementary SEM–EDS analysis (Supplementary Fig. 4) corroborates these observations, revealing Co-enriched surface regions consistent with a minor Co_3_O_4_ phase. Additional multi-region STEM–EDS mapping analyses further confirm the reproducibility and spatial distribution of the surface Co_3_O_4_ domains (Supplementary Fig. 5). Collectively, these findings demonstrate that indium substitution drives lattice strain-mediated Co segregation and self-assembly of a surface spinel layer, which reconstructs a coherent interface that underpins the enhanced electrochemical performances of the In-modified electrodes.

### Covalency/valence redistribution and pathway densification

**Fig. 3:**
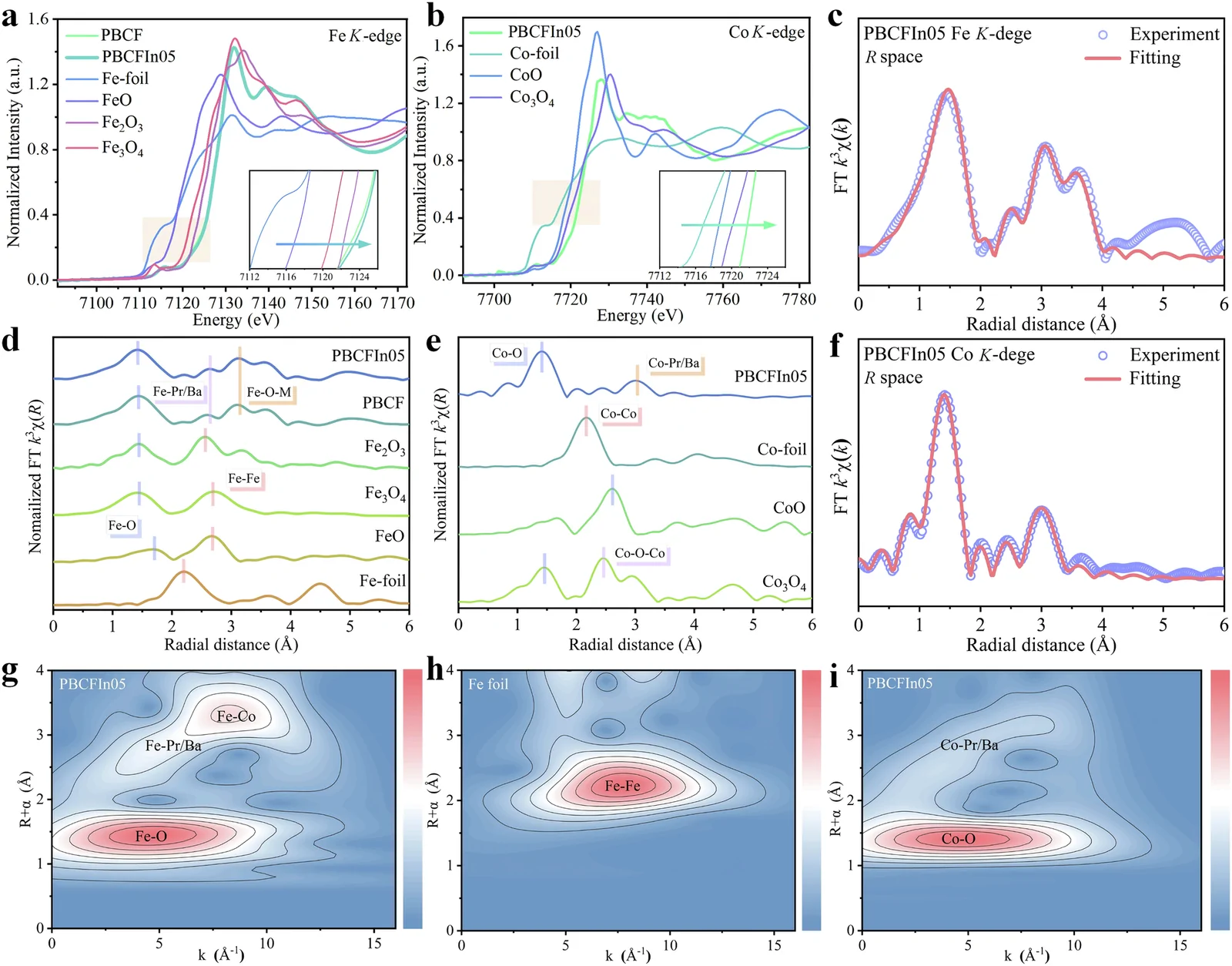
XAS characterization of PBCF and PBCFIn05. **a** Normalized Fe *K*-edge XANES spectra. **b** Normalized Co *K*-edge XANES spectra. **c** Fe *K*-edge Fourier-transformed (FT) FT–EXAFS spectra of PBCFIn05 in *R* space, along with experimental and fitted curves. **d** *K*^3^-weighted FT–EXAFS spectra of the Fe *K*-edge. **e** FT–EXAFS spectra of the Co *K*-edge. **f** Co *K*-edge FT-EXAFS spectra of PBCFIn05 in *R* space, along with experimental and fitted curves. **g** Fe *K*-edge EXAFS oscillations of PBCFIn05. **h** Fe *K*-edge EXAFS oscillations of Fe foil (reference). **i** Co *K*-edge EXAFS oscillations of PBCFIn05. Source data for Fig. 3 are provided as a Source Data file.

Synchrotron Fe *K*-edge EXAFS analysis reveals distinct differences in the local coordination and electronic structure of PBCFIn05 compared with pristine PBCF, linking lattice-level evolution to covalency reconfiguration. The Fe *K*-edge XANES spectra (Fig. 3a) display similar overall features, consistent with comparable perovskite backbones; however, the absorption edge of PBCFIn05 shifts to higher energy, indicating a higher average Fe valence. The inset of Fig. 3a (normalized intensity ~0.2–0.3) confirms an Fe oxidation state above +3 in both samples, with PBCFIn05 showing a slightly higher value. To quantitatively resolve local structural differences, EXAFS fitting and Fourier transforms (FT) were performed (Fig. 3c, d, Supplementary Fig. 6, and Table S4). In *R* space, the first-shell peak of PBCFIn05 shifts slightly toward lower *R* with increased amplitude, corresponding to a shorter Fe–O bond (1.97 Å vs. 2.00 Å) at comparable coordination (*N*_Fe–O_ ≈ 5.7). This bond contraction reflects enhanced Fe–O covalency and a higher mean Fe valence in PBCFIn05. The similar Debye–Waller factors (*σ*^2^ ≈ 0.009–0.008 Å^2^) and nearly identical Δ*E*_o_ values (≈−2.8 ± 1.5 vs. −2.5 ± 1.3 eV) rule out fitting artefacts, confirming genuine lattice effects. The amplitude factor *S*_o_^2^ = 0.73 (referenced to Fe foil) further supports the reliability of the analysis. Wavelet transform (WT) maps (Fig. 3g–i and Supplementary Fig. 7) provide additional resolution of scattering paths. Beyond the first shell, PBCFIn05 retains a higher Fe-Co effective coordination (5.5 vs. 4.6) at a similar Fe-Co distance (~4.02 Å), suggesting a denser and more continuous B-site sublattice. In *K* space, PBCFIn05 displays stronger oscillation amplitude. Given the similar first-shell *σ*^2^ and a common *S*_o_^2^, this difference more plausibly reflects a slightly shorter average bond length and lower overall disorder, rather than a single-cause increase in backscattering strength. Together with the XANES edge shift, these results indicate an increased Fe^4+^ fraction and strengthened Fe 3*d*-O 2*p* hybridization in PBCFIn05^34–36^.

The Fe K-edge pre-edge (~7111–7115 eV) further clarifies valence and symmetry effects. A higher-energy pre-edge centroid in PBCFIn05 reflects both elevated Fe valence and local crystal field modification, while its increased intensity signals centrosymmetric breaking in FeO_6_ octahedra due to 3*d*-4*p* orbital mixing (Supplementary Fig. 8)^37^. Under strong covalency or negative charge-transfer conditions, such vacancies manifest primarily as ligand holes on O 2*p* orbitals, accompanied by Fe–O bond contraction rather than elongation^38,39^. The resulting Fe–O contraction and improved Fe-Co connectivity amplify the *e*_g_–*p*_σ_–*e*_g_ overlap along the Fe-O-Co linkages, strengthening the double-exchange hopping integral (*t*_pdσ_ ∝ *d*^−3^ to *d*^−3.5^ Harrison scaling). A higher Fe-Co coordination (PBCFIn05: 5.5 > PBCF: 4.6) also implies a greater density of parallel Fe-O-Co transport channels.

Co K-edge XANES/EXAFS measurements reveal corresponding adjustments in Co coordination and electronic configuration (Fig. 3b, e, f, Supplementary Fig. 9, and Table S5). The pre-edge feature near 7726 eV, assigned to the 1*s* → 3*d* quadrupole transition, which is highly sensitive to Co-O octahedral symmetry and 3*d*-state occupancy (Supplementary Fig. 10). Compared with the reference Co_3_O_4_, in which octahedral Co^3+^ is predominantly stabilized in the low-spin (LS) state within the spinel lattice, PBCFIn05 exhibits a higher-energy pre-edge with enhanced intensity. This shift indicates an increased average Co valence and a partial distortion/under-coordination of the Co-O polyhedral. The stronger pre-edge feature is consistent with centrosymmetric breaking of CoO_6_ octahedra (3*d*-4*p* mixing) and the emergence of higher-valent Co^4+^ species. In addition, EXAFS fits give a lower Co-O coordination (5.3 ± 0.3) and a significantly shorter Co-O bond (1.89 ± 0.01 Å), indicating Co^3+^ →  Co^4+^ oxidation and the formation of under-coordinated, distorted octahedra or five-coordinate motifs. The shorter Co-O bond is consistent with stronger Co-O covalency and the smaller ionic radius of Co^4+^. Fit robustness is supported by an *R*-factor of ≈0.0118 and Δ*E*_o_ ≈ −4.0 ± 0.9 eV, with *S*_o_^2^ referenced to Co foil (0.74). Furthermore, wavelet transform (WT) analysis resolves the dominant Co-O and Co-Co scattering paths, highlighting the shortened Co-O coordination in PBCFIn05 compared with reference compounds (Supplementary Fig. 11).

Within an octahedral field, the higher Co valence and stronger Co-O covalency increase crystal-field splitting and induce ligand-hole character, driving a redistribution of valence and covalency. Through the disproportionation 2Co^3+^ ⇌ Co^2+^ +  Co^4+^, the system attains a higher Co^4+^ fraction while maintaining a small Co^2+^ component. Co^4+^ contributes partially unoccupied *e*_g_ states, while a limited population of IS-Co^3+^/HS-Co^2+^ increases *t*_2g_ electron count, bringing the effective *e*_g_ occupancy closer to unity, balancing carrier density and mobility—avoiding small-polaron trapping due to *e*_g_ overfilling and pathway dilution due to *e*_g_ depletion. In concert with the shorter Co-O bond, the hopping integral and bandwidth are both enhanced^37,40^. Coupled with robust Fe-Co linkage, cooperative *e*_g_–*p*_σ_–*e*_g_ double-exchange pathways are established across Fe^3+^(*t*_2g_^3^*e*_g_^2^)-O^2–^-Co^4+^(*t*_2g_^5^*e*_g_^0^) and Co^3+^(*t*_2g_^5^*e*_g_^1^)-O^2–^-Co^4+^(*t*_2g_^5^*e*_g_^0^) linkages within the perovskite lattice, while Co^2+^-O-Co^3+^ couples in interfacial Co_3_O_4_ contribute to polaronic hopping. Importantly, the self-assembled Co_3_O_4_ at the PBCFIn05/Co_3_O_4_ heterointerface should be regarded not as an inert inclusion but as a valence-buffering, polaron-friendly mediator that couples bulk conduction to interfacial redox. By stabilizing higher-valent Co species and ligand-hole states on the perovskite side, reinforcing local *e*_g_–*p*_σ_–*e*_g_ connectivity, and enabling small-polaron transport in the spinel phase, this heterointerface reduces interfacial charge-transfer resistance and accelerates oxygen-exchange kinetics. These mechanisms align with the experimentally observed improvements in dc conductivity and ORR/OER activity. Any potential spin involvement at the interface may provide a spin-matching environment, though confirmation would require dedicated magneto-electro-transport studies.

### Surface valence state distribution and catalytic activity

**Fig. 4:**
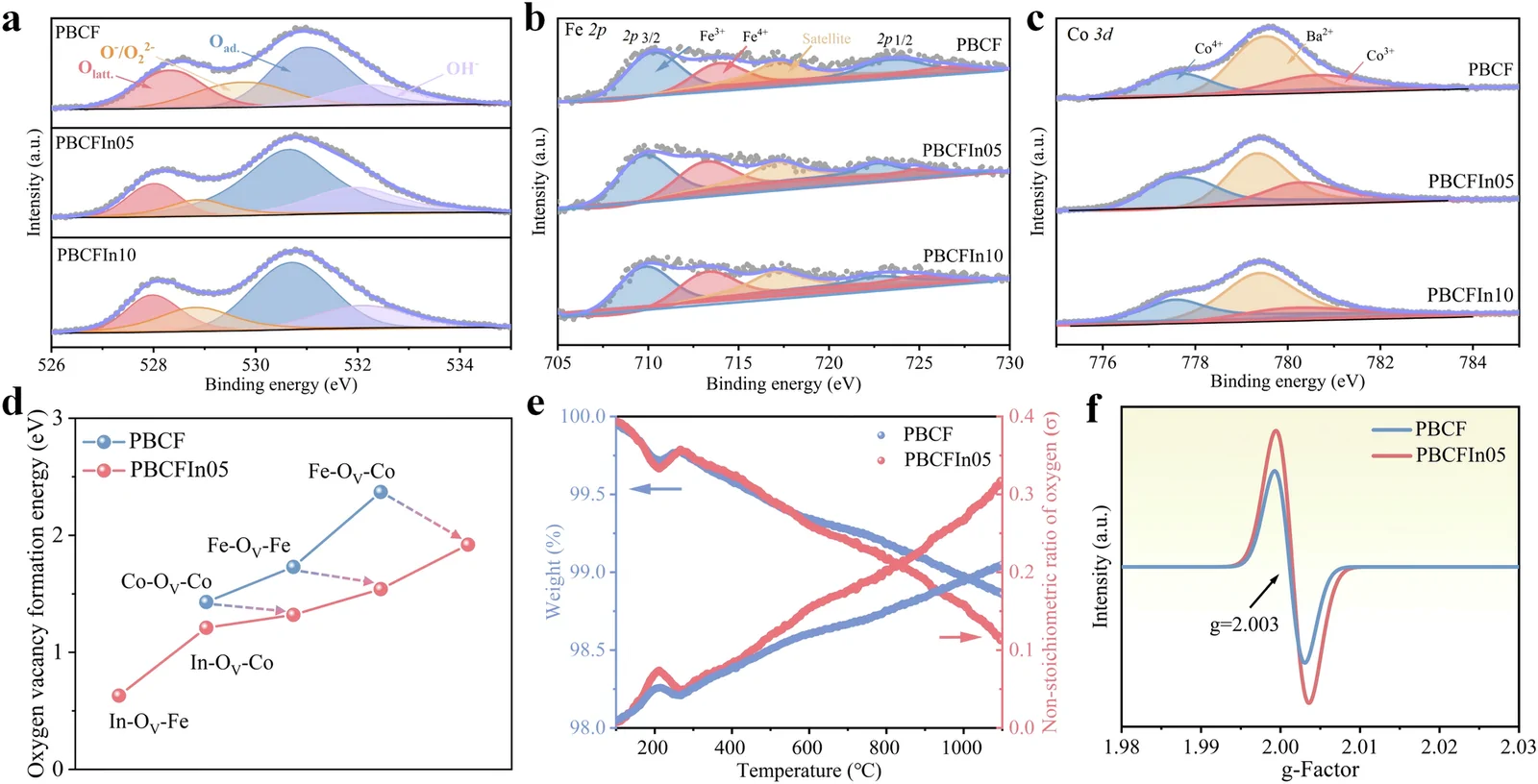
Surface valence state distribution and catalytic activity. **a** O 1*s* XPS spectra, **b** Fe *2p* XPS spectra, **c** Co 3*d* XPS spectra of PBCF, PBCFIn05, and PBCFIn10. **d** Calculated oxygen-vacancy formation energies derived from DFT simulations. Arrows show the lowered vacancy formation energy. **e** Thermogravimetric (TG) curves of PBCF and PBCFIn05 and the associated changes in oxygen nonstoichiometric ratios. **f** Electron paramagnetic resonance (EPR) spectra of PBCF and PBCFIn05. Source data for Fig. 4 are provided as a Source Data file.

The oxidation states and elemental compositions of Fe, Co, and O in PBCF, PBCFIn05, and PBCFIn10 were investigated using X-ray photoelectron spectroscopy (Supplementary Fig. 12). As shown in Fig. 4a, the O 1*s* core-level spectra can be deconvoluted into four distinct peaks at approximately 528.3, 529.1, 530.3, and 532.3 eV, corresponding to lattice oxygen (O_lat_), adsorbed oxygen species (O^–^/O_2_^2–^), highly oxidized oxygen species (O_ads_), and hydroxyl groups (OH^–^), respectively. The ratio of (O_ads_) to O_lat_, derived from the corresponding peak areas, serves as an indicator of oxygen vacancy concentration^41^. Among the samples, PBCFIn05 exhibits the highest O_ads_/O_lat_ ratio (Supplementary Table 6), suggesting a greater abundance of oxygen-vacancy-related surface species. Such enriched surface oxygen species facilitate proton absorption and accelerate oxygen-related reaction kinetics, which plausibly explains the enhanced catalytic activity of PBCFIn05 compared with PBCF and PBCFIn10 under air electrode operation.

To further elucidate the effect of In doping on oxygen vacancy formation and its influence on the electronic structure of transition metal ions, the Fe valence states were analyzed. The Fe 2*p* spectra (Fig. 4b) display two asymmetric peaks resulting from spin–orbit splitting, corresponding to Fe^3+^ (710.2 eV) and Fe^4+^ (712.2 eV). Deconvolution shows that the proportion of Fe^4+^ increases from 30.4% in PBCF to 34.5% in PBCFIn05, while Fe^3+^ decreases from 69.6 to 65.5% (Supplementary Table 7). The valence states of Co 2*p* spectra (Fig. 4c) exhibit a higher binding energy peak at ~781.7 eV assigned to Co^4+^ and a lower energy peak at ~777.8 eV assigned to Co^3+^^42^. In the pristine sample, the Co^4+^ content is 54.34%, which increases to 60.3% and 63.0% in PBCFIn05 and PBCFIn10, respectively (Supplementary Table 8). The slightly increased Co^4+^ fraction upon In^3+^ incorporation may arise from partial oxidation of Co due to the local coordination changes^43^. Notably, Co^4+^ species are widely recognized as active species that promote the ORR process^43,44^.

To elucidate the origin of enhanced oxygen vacancy formation, DFT calculations were performed to evaluate the oxygen vacancy formation energies. As shown in Fig. 4d, the calculated values for Co-V_O_-Co, Fe-V_O_-Fe, and Co-V_O_-Fe configurations in pristine PBCF are 1.43, 1.73, and 2.37 eV, respectively. In contrast, these values decrease to 1.21, 1.54, and 1.92 eV in PBCFIn05, demonstrating that In incorporation substantially promotes oxygen vacancy formation. Notably, the formation energies for oxygen vacancies near In-O-Fe and In-O-Co environments are further reduced to 0.63 eV and 1.32 eV, respectively, confirming that In^3+^ doping energetically favors vacancy generation. The corresponding relaxed structures and adsorption geometries of oxygen vacancies at different lattice sites are displayed in Supplementary Figs. 13 and 14. Thermogravimetric analysis (TGA) was also conducted to assess the release of lattice oxygen associated with cation reduction. As shown in Fig. 4e, PBCFIn05 exhibits a total weight loss of 1.43% over 30–1100 °C, compare with 1.14% for pristine PBCF, consistent with enhance oxygen non-stoichiometry. Iodometric titration further reveals δ values of approximately 0.20 for PBCF and 0.31 for PBCFIn05, indicating a higher concentration of oxygen vacancies in the In-doped sample^45,46^.

To gain further insight into defect structures, electron paramagnetic resonance (EPR) spectroscopy was performed on PBCF and PBCFIn05 (Fig. 4f). The PBCFIn05 sample exhibits a markedly stronger signal at *g* = 2.003, characteristic of unpaired electrons associated with vacancy-related paramagnetic centers, in agreement with XPS, DFT, and TGA analyses. The intensified EPR response also suggests increased spin-state variability and the coexistence of multiple spin configurations are often correlated with modified magnetic properties^21,28^. The above results collectively confirm that In^3+^ doping effectively enhances oxygen vacancy formation and stabilizes vacancy-related defect states. Since these vacancies serve as active sites for water adsorption and dissociation, the H_2_O-temperature-programmed desorption (H_2_O-TPD) measurements were performed to probe hydration (Supplementary Fig. 15). Compared with PBCF, PBCFIn05 exhibits stronger H_2_O desorption signals, with pronounced features in both the low-temperature region (50–200 °C) and medium-temperature (~400 °C) regions, indicating the presence of more weakly bound water and diverse binding sites. The protonic properties of the air electrode were further examined by quantifying the released water and proton uptake using Karl Fischer titration (Supplementary Fig. 16 and Table 9). Compared with pristine PBCF, the In-doped samples exhibit a markedly enhanced water content associated with protonic species and proton uptake, with PBCFIn05 showing the highest released H_2_O content (0.5845 mmol H_2_O mol^−1^ oxide) and corresponding proton uptake (1.169 mmol H mol^−1^ oxide). These results suggest that In incorporation modulates the defect chemistry and surface–water interaction of the oxide, leading to an enhanced capability to accommodate water-derived protonic species. Such a change is expected to be beneficial for proton participation in the electrode reactions under R-PCEC operation.

### R-PCECs performance and mechanisms

**Fig. 5:**
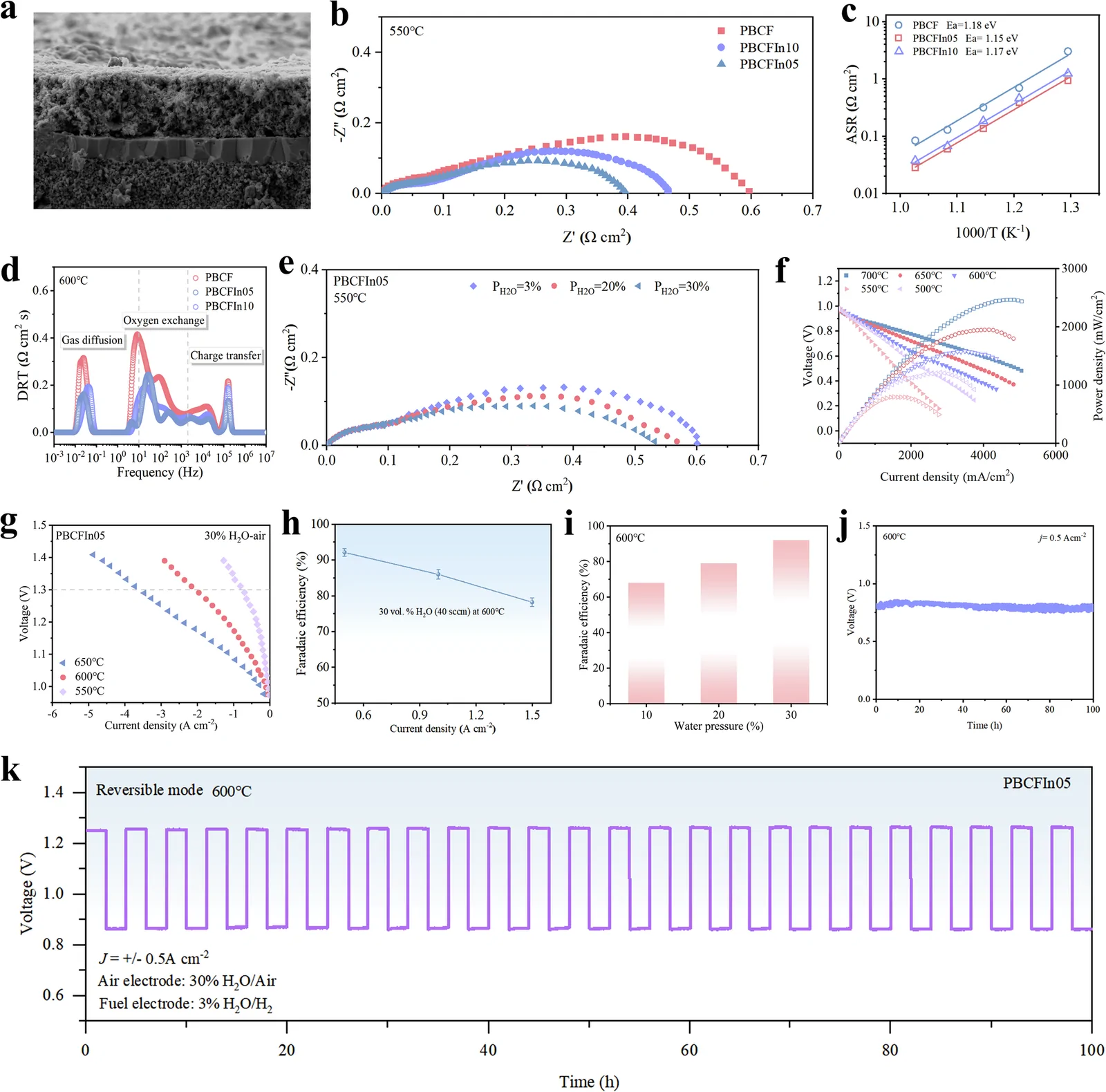
Electrochemical performance and stability of single cells in fuel-cell (FC) mode. **a** Cross-sectional SEM image of PBCFIn05 single-cell structure. **b** EIS plots of BZCYYb-supported single cell with PBCFIn05 electrode at 550 °C under 3%H_2_O–H_2_. **c** Arrhenius plot of the ASRs for PBCF, PBCFIn05, and PBCFI10. **d** The DRT analysis of EIS was measured at PBCF, PBCFIn05, and PBCFIn10 electrodes at 600 °C. **e** EIS plots of cell with PBCFIn05 air electrode tested at 500 °C under 3, 10, 30% H_2_O–air. **f** *I*–*V* and *I*–*P* curves of a BZCYYb-supported single cell with PBCFIn05 air electrode in FC mode at 500–700 °C. **g** *I*–*V* curves of cells with PBCFIn05 electrode measured in EC mode at 650–550 °C under 30% H_2_O–air. **h** FE of cells at different current densities. **i** FE of cells at different PH_2_O. **j** A short durability assessment of a PCFCIn05 at a consistent current density of 0.5 A cm^–2^ and a temperature of 600 °C. **k** A stability test of PBCFIn05 in ±0.5 A cm^−2^ for 100 h at 600 °C. The reported voltage values are not iR-corrected. Source data for Fig. 5 are provided as a Source Data file.

To evaluate the ORR activity of PBCFIn_*x*_ (*x* = 0, 0.05, 0.10) as air electrodes, electrochemical impedance spectroscopy (EIS) measurements were conducted on single cells comprising a Ni-BZCYYb fuel electrode, a dense BZCYYb electrolyte, and PBCFIn_*x*_ as the air electrode. The tests were performed under 3% H_2_O-H_2_ at 500–700 °C using the custom-built testing fixture shown in Supplementary Fig. 17. As shown in Fig. 5a and Supplementary Fig. 18, the cross-sectional SEM image of PBCF, PBCFIn05, and PBCFIn10-based single cell clearly reveals a dense electrolyte and well-bonded porous electrode layers. The temperature-dependent EIS spectra (Fig. 5b and Supplementary Fig. 19) show that PBCFIn05 exhibits the lowest area-specific resistance (ASR), reaching 0.39 Ω cm^2^ at 550 °C, substantially lower than both the undoped and heavily doped counterparts. As summarized in Fig. 5c, PBCFIn05 consistently shows the lowest ASR and apparent activation energy across the 500–700 °C tested range, underscoring its superior ORR catalytic performance. In contrast, excessive In^3+^ doping (*x* = 0.1) deteriorates electrode activity, as pronounced Co^3+^ segregation induces bulk Co_3_O_4_ formation that disrupts the perovskite lattice and hinders charge transport. Conversely, a moderate In level (*x* = 0.05) promotes the in situ formation of a thin, coherently coupled Co_3_O_4_ interfacial layer that buffers cation valence states and introduces additional small-polaron hopping pathways, thereby optimizing charge transfer and catalytic activity.

To further resolve the kinetic processes governing ORR, distribution of relaxation time (DRT) analysis was applied to EIS data at 600 °C. The DRT profiles were divided into three characteristic frequency regions: low frequency (LF, <10 Hz) associated with gas diffusion, intermediate frequency (IF, 10–2000 Hz) corresponding to surface oxygen exchange, and high frequency (HF, >2000 Hz) attributed to charge transfer. As shown in Fig. 5d, PBCF and PBCFIn10 exhibit larger LF and IF response areas, indicating sluggish oxygen diffusion and surface exchange kinetics^47,48^. In contrast, PBCFIn05 shows significantly reduced relaxation intensities in both regions, suggesting more facile O_2 (g)_ → 2O_ad_ dissociation and faster O_ad_ → O_ad_^–^ charge transport, which together accelerate overall ORR kinetics^47,49^. In addition, the effect of water partial pressure on electrode behavior under electrolysis operation was studied, and EIS spectra were recorded at 550 °C with varying water pressure. As shown in Fig. 5e, the polarization resistance (*R*_p_) decreases from 0.60 to 0.53 Ω cm^2^ as the water pressure increases from 3 to 30%, confirming that elevated humidity enhances proton transport and hydration at the electrode-electrolyte interface^50,51^. Supplementary Fig. 20 shows the ASR evolution of PBCFIn05 at 650 and 600 °C under different steam partial pressures (3, 20, and 30%), while Supplementary Fig. 21 compares *R*_p_ values for PBCF and PBCFIn10 at 550 °C and 30% H_2_O (0.97 and 0.72 Ω cm^2^, respectively). The progressive decrease in ASR and *R*_p_ with increasing steam partial pressure reflects enhanced electrolyte hydration and increased protonic defect concentration, which facilitate proton transport and interfacial charge-transfer kinetics. In addition, excess indium incorporation further lowers *R*_p_ under humid conditions by strengthening metal-oxygen covalency and promoting more efficient proton-coupled oxygen electrochemistry at the heterointerface. These results demonstrate that moderate In doping (*x* = 0.05) achieves an optimal balance between electronic conductivity, ORR catalytic activity, and gas transport characteristics.

The improved catalytic properties of PBCFIn05 are further validated under both fuel cell (FC) and electrolysis cell (EC) modes. Figure 5f presents the *I–V* and power density curves in FC mode. The PBCFIn05-based single cell delivers peak power densities (PPD) of 2.47, 1.95, 1.58, 1.21, and 0.79 W cm^–2^ at 700–450 °C, respectively. For comparision, the undoped PBCF-based cell exhibits significantly lower PPD values of 1.34, 1.01, 0.62, 0.28 and 0.14 W cm^−2^ at 700–500 °C under identical testing conditions (Supplementary Fig. 22). As summarized in Supplementary Fig. 23 and Supplementary Table 10, the PBCFIn05 cell delivers competitive PPD compared with reported air electrodes for fuel cell configurations^23,32,48,52–78^. The electrochemical performance in EC mode at 1.3 V is shown in Fig. 5g. The PBCFIn05-based cell achieves current densities of 3.72, 2.16, and 0.81 A cm^–2^ at 650, 600, and 550 °C, respectively. Supplementary Fig. 24 presents the *I*–*V* curves under different steam partial pressures (3 and 20%), while Supplementary Fig. 25 compares the corresponding results for PBCFIn10 and PBCF at 550 °C. Performance comparisons summarized in Supplementary Fig. 26 and Table 11 further confirm the good dual-mode reversibility and stability of PBCFIn05.

In electrochemical energy conversion systems, the Faradaic efficiency (FE) is a critical metric reflecting the fraction of charge effectively converted into the desired reaction products^79,80^. The FE of the PBCFIn05-based cell was evaluated using gas chromatography under varying current densities and steam concentrations at 600 °C (Fig. 5h, i, and Supplementary Table 12). As the applied electrolysis current density increases from 0.5 to 1.5 A cm^−2^, the FE gradually decreased from 92 to 78%, which is attributed to a reduced oxygen partial pressure at the fuel electrode that enhances the electrolyte’s electronic conductivity and induces minor electron leakage. The electrons generated within the electrolyte under these conditions can be driven by the applied electric field, forming an internal electronic leakage current that does not contribute to the intended electrolysis reaction^81^. As a result, although the total external current remains unchanged, the fraction of current effectively utilized for hydrogen production decreases, leading to a reduction in the measured FE^82,83^. Conversely, the FE increased with rising steam partial pressure, consistent with the suppression of electronic defect formation and the enhanced hydration of the electrolyte and interfacial region under high-humidity conditions, which favor protonic transport and reduce electronic leakage^79,84^. Detailed calculation formulas and procedures are provided in the Supporting Information. Electrochemical stability was further examined through short-term durability tests conducted at 0.5 A cm^−2^ for 100 h at 600 °C (Fig. 5j). Under these conditions, the cell exhibited a gradual and monotonic voltage decay without abrupt performance loss, indicating reasonably stable operation over the examined time scale. The observed mild degradation behavior is consistent with a persistent but slow interfacial or surface evolution process, rather than transient surface-site deactivation. Post-test SEM analyses reveal no pronounced morphological, structural, or chemical changes (Supplementary Fig. 27.

Thermal cycling durability was subsequently evaluated under three distinct heating-cooling protocols between 700 and 600 °C (Supplementary Fig. 28). In mode 1, the cell experienced five moderate thermal cycles at a rate of 1.6 °C min^−1^ to simulate fatigue conditions. In modes 2 and 3, more aggressive cycling profiles were applied (heating at 10 and 20 °C min^–1^; cooling at 5 and 10 °C min^–1^) for up to 65 cycles. Remarkably, the PBCFIn05 air electrode maintained nearly constant current output with negligible degradation across all thermal stress tests, confirming its stable thermomechanical behavior. The post-cycling SEM results show no noticeable particle coarsening, cracking, or interfacial delamination compared with the pristine electrode, indicating that the microstructure and electrode–electrolyte interface remain largely preserved after thermal cycling(Supplementary Fig. 29). The durability under reversible operation was further evaluated by galvanostatic current cycling between ±0.5 A cm^−2^ for 100 h at 600 °C with periodic switching between FC and EC modes (Fig. 5k). The R-PCEC employing the PBCFIn05 electrode exhibits excellent reversibility and tolerance during prolonged bidirectional cycling. Structural stability was further verified by XRD analysis of PBCFIn_*x*_ samples before and after exposure to 30% H_2_O-containing air at 600 °C for 50 h (Supplementary Fig. 30). No additional diffraction peaks or noticeable peak shifts were observed, indicating the excellent phase and structural integrity of PBCFIn05 under harsh operating conditions. To further evaluate the chemical compatibility between the PBCFIn05 air electrode and the BZCYYb electrolyte, a mixture of the two powders was heated at 900 °C for 3 h in air and subsequently analyzed by X-ray diffraction. As shown in Supplementary Fig. 31, the diffraction peaks can be indexed to the PBCFIn05 and BZCYYb phases without detectable impurity phases after heat treatment, indicating good chemical compatibility between the electrode and electrolyte under typical cell fabrication conditions.

### DFT calculations and mechanism analysis

**Fig. 6:**
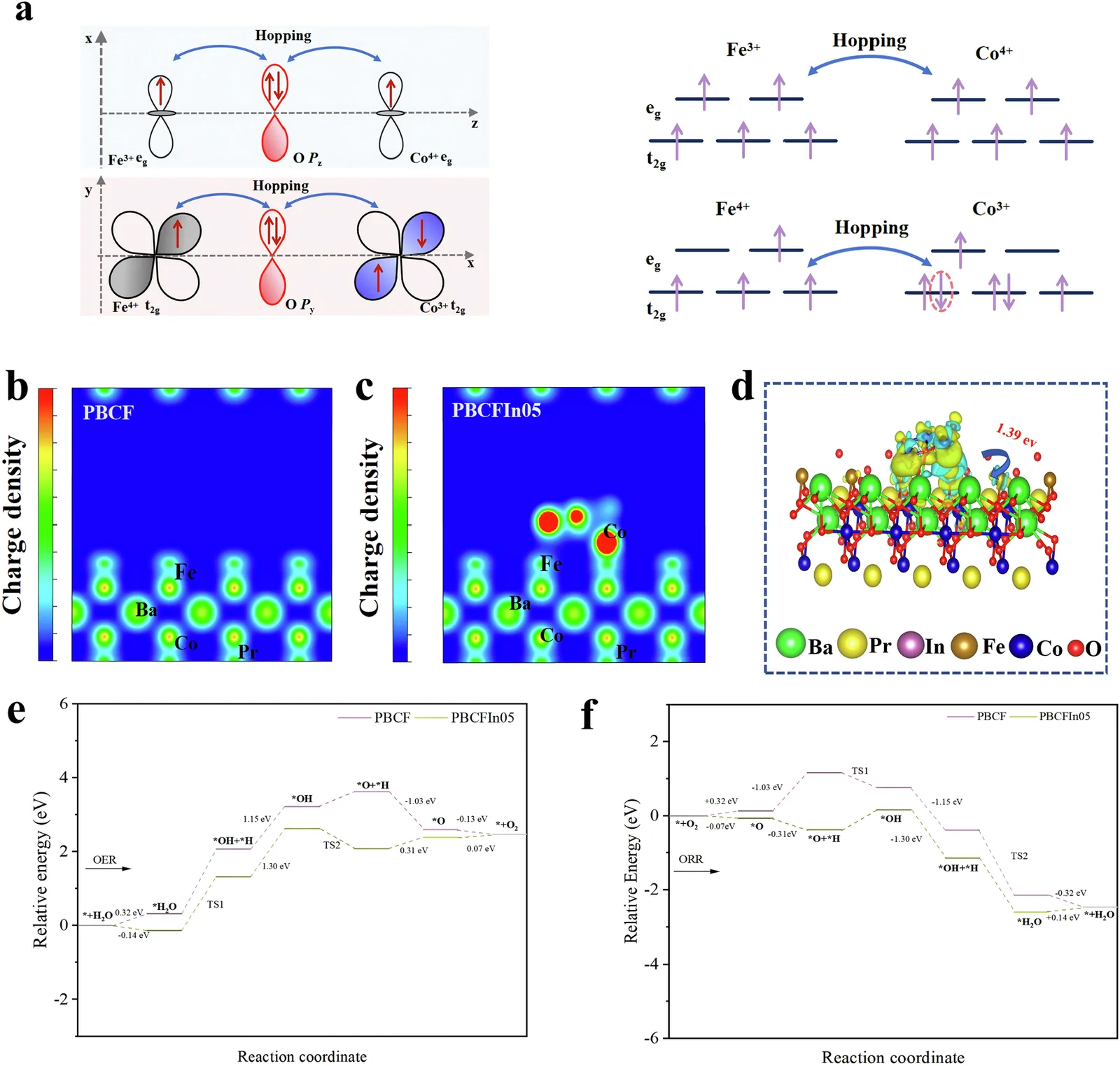
DFT calculations regarding mechanisms on the air electrodes of R-PCECs. **a** Schematic diagram of electronic hopping pathways in PBCFIn05. Top: Fe^3+^-O-Co^4+^ chain with *e*_g_–*p*_σ_–*e*_g_ overlap. Bottom: Fe^4+^-O-Co^3+^ linkage with *t*_2g_–*p*_π_–*t*_2g_ overlap. **b**, **c** Differential charge density in PBCF and PBCFIn05. **d** Differential charge density of PBCFIn05 (side view). **e**, **f** Calculated relative DFT energy profiles of the OER and ORR on PBCF and PBCFIn05. Source data for Fig. 6 are provided as a Source Data file and Supplementary Data 1 file.

The parent perovskite PBCF features a mixed-valent B-sublattice in which Fe and Co are interconnected through an extended Fe-O-Co framework. Electronic transport primarily proceeds via symmetry-matched *e*_g_–*p*_σ_–*e*_g_ hopping between neighboring B sites. However, the limited population of higher-valent Co and moderate oxygen-vacancy levels constrain the density of mixed-valence channels, leading to only moderate ORR/OER activity. While Fe–O–Fe superexchange interactions predominantly favor antiferromagnetic coupling and contribute little to long-range conduction, Fe^4+^-O-Co^3+^ linkages can serve as auxiliary pathways via *t*_2g_– *p*_π_–*t*_2g_ overlap or thermally activated small-polaron hopping, which are both weaker than the dominant Fe^3+^-O-Co^4+^ *e*_g_–*p*_σ_–*e*_g_ channel. Upon In incorporation (PBCFIn05), the system undergoes a valence/covalency redistribution, characterized by an increased Co^4+^ fraction, shortened Fe/Co–O bonds, and strengthened metal-oxygen covalency. This redistribution increases the population of partially unoccupied *e*_g_ states on neighboring sites, thereby densifying the *e*_g_–*p*_σ_–*e*_g_ pathways within the B-sublattice (Fig. 6a, top). Electrons on Fe^3+^ sites can thus hop into vacant *e*_g_ states of adjacent Co^4+^ through an O–2*p*_σ_ bridge, enhancing the mixed-valence hopping integral. Because Fe and Co centers are bridged by oxygen, their *e*_g_ orbitals align along the metal-oxygen–metal axis and couple effectively with the O *p*_z_ orbitals, forming an *e*_g_–*p*_z_–*e*_g_ conduction channel. In this configuration, Fe^3+^-O-Co^4+^ chains with predominant *σ*-type overlap allow electrons in the Fe^3+^ level to hop into the vacant state of adjacent Co^4+^. According to Hund’s rule, spin-allowed hopping is facilitated when partial vacancies exist in the *e*_g_ manifold. In parallel, Fe^4+^-O-Co^3+^ linkages, though less conductive to coherent *e*_g_–*p*_σ_–*e*_g_ exchange, can still contribute via *t*_2g_–*p*_π_–*t*_2g_ interactions or polaron hopping (Fig. 6a, down). These secondary channels help maintain the electronic connectivity of the B–O–B framework under vacancy-rich or locally distorted conditions.

Beyond lattice modulation, In doping also influences the synthesis environment, promoting the in situ formation of a tightly anchored Co_3_O_4_ spinel layer on the PBCF surface. This layer forms a strongly coupled heterointerface with the perovskite lattice, introducing Co^2+^/Co^3+^ redox couples and expanding the accessible valence window. At the perovskite-spinel boundary, Co^2+^–O–Co^3+^ chains within Co_3_O_4_ establish polaron-compatible transport channels that couple with the perovskite Fe-O-Co *e*_g_–*p*_σ_–*e*_g_ network. Spin interactions at this interface provide additional spin-matching conditions, while local lattice mismatch and disorder generate oxygen vacancies that enhance carrier mobility and act as soft adsorption sites for tuning OH*, O*, and OOH* binding energies. As a result, interfacial charge-transfer resistance is lowered, and oxygen-exchange kinetic are significantly accelerated without overbinding of intermediates. To probe the electronic structure and bonding features, differential charge-density analyses were carried out using DFT (Fig. 6b–d). In pristine PBCF, charge distribution around the Fe/Co–O framework is relatively uniform, suggesting minimal electronic polarization. In contrast, PBCFIn05 shows evident electron accumulation (yellow) at Fe/Co sites and depletion (blue) around neighboring oxygen. This redistribution arises from the in situ formation of the Co_3_O_4_ spinel and its intimate coupling with the PBCF lattice. Quantitatively, approximately 1.39 eV of charge transfer occurs across the local coordination environment, reflecting strong electronic interaction^74^. This reconstruction not only enhances the bulk Fe^3+^-O-Co^4+^ double-exchange channel but also introduces an additional interfacial charge-transfer pathway across the PBCF/Co_3_O_4_ boundary.

To gain mechanistic insight into the oxygen reaction energetics on PBCF and PBCFIn05 surfaces, DFT calculations were performed to evaluate surface intermediates and elementary reaction steps (Supplementary Figs. 32–35). The relative energies of selected surface intermediates and the CI-NEB-derived activation barriers were analyzed separately (Fig. 6e, f). The OER and ORR-related transition states are denoted as TS1 and TS2. For the OER-related steps, CI-NEB calculations show that the activation barrier for water dissociation ∗H_2_O → *OH + *H (TS1) decreases from 1.95 eV on pristine PBCF to 1.60 eV on PBCFIn05. The subsequent hydroxyl deprotonation step *OH → *O + *H (TS2) also exhibits a reduced barrier, decreasing from 0.71 eV on PBCF to 0.22 eV on PBCFIn05 (Supplementary Fig. 36). The ORR-related CI-NEB results involving TS1 and TS2 are provided in Supplementary Fig. 37. These results indicate that excess indium incorporation facilitates proton-transfer and deprotonation-related surface processes. To further evaluate hydrogen-related surface transport processes, CI-NEB calculations were also performed to determine the proton migration barriers on the PBCF and PBCFIn05 surfaces (Supplementary Fig. 38)^85^. The calculated migration barrier decreases from 2.014 eV on pristine PBCF to 1.011 eV on PBCFIn05, indicating that indium incorporation significantly facilitates proton transport across the surface. This reduced migration barrier further contributes to the enhanced oxygen reaction kinetics observed experimentally. It should be noted that the DFT models used here represent simplified and idealized structures, which may not fully capture the structural complexity, surface reconstruction, defect distribution, and gas–solid environment of the real electrode under operating conditions. Taken together, the coexistence of a bulk-regulated B-site electronic network and an interfacial Co_3_O_4_ spinel layer expands the spin-regulated hopping landscape through electronic reconstruction and defect engineering. This synergistic coupling markedly enhances electronic transport and oxygen-intermediate kinetics, in good agreement with the experimentally observed improvement in bifunctional activity. The resulting magnetic-electronic reconstruction bridges bulk charge transport and surface electrocatalysis, offering a powerful design strategy for next-generation oxygen electrode materials.

## Discussion

This study reveals a comprehensive structure–property–performance correlation in In-doped Pr_0.5_Ba_0.5_Co_0.7_Fe_0.3_O_3 − δ_ perovskites, demonstrating that moderate In^3+^ incorporation orchestrates a cooperative reconstruction spanning both the bulk lattice and the surface interface. From crystallographic, spectroscopic, and microscopic perspectives, In doping induces subtle yet consequential modifications to the perovskite framework. The substitution of In^3+^ for B-site cations perturbs the Fe-O-Co network, introducing local bond-angle distortions and promoting the formation of oxygen vacancies. These structural relaxations trigger a redistribution of cation valences and spin states, increasing the Co^4+^ fraction and strengthening Fe^3+^-O-Co^4+^ double-exchange pathways. Consequently, the lattice experiences enhanced *e*_g_–*p*_σ_–*e*_g_ orbital overlap, facilitating spin-allowed hopping and efficient bulk electronic conduction.

Concurrently, In incorporation alters the chemical environment during synthesis, leading to the in situ formation of a thin, coherently coupled Co_3_O_4_ spinel layer at the perovskite surface. This self-assembled heterointerface introduces complementary Co^2+^/Co^3+^ redox couples and small-polaron hopping channels that bridge the electronic states across the perovskite-spinel junction. The interfacial Co_3_O_4_ domains not only extend the mixed-valence transport network but also act as a dynamic redox buffer, accommodating valence fluctuations and stabilizing the oxygen sublattice under operation. The combination of lattice oxygen vacancies and interface-derived active sites establishes an optimal balance between oxygen-ion mobility and electron conductivity, while also tuning the adsorption energies of OH*, O*, and OOH* intermediates for accelerated oxygen exchange kinetics.

Electrochemically, these synergistic effects translate into markedly enhanced bifunctional activity. PBCFIn05 exhibits the lowest polarization resistance, fastest surface exchange, and highest ORR/OER activity among all compositions. As an air electrode in R–PCECs, it delivers exceptional performance, with PPD of 2.47, 1.95, 1.58, 1.21, and 0.79 W cm^–2^ at 700–500 °C, respectively, together with improved durability and thermomechanical resilience under prolonged cycling. The FE remains above 90% at moderate current densities, confirming high selectivity and minimal parasitic losses.

DFT calculations further corroborate these experimental findings. Electronic structure analyses reveal that In incorporation and Co_3_O_4_ coupling jointly raise the O 2*p* band center, increase the density of states near the Fermi level, and promote stronger Fe/Co-O hybridization. Differential charge-density mapping confirms substantial charge transfer (~1.39 eV) across the PBCF/Co_3_O_4_ interface, signifying robust electronic coupling and interfacial reconstruction. Reaction-pathway analyses based on CI-NEB calculations reveal that indium incorporation significantly lowers the activation barriers of key oxygen-reaction steps, with the water dissociation barrier decreasing from 1.95 eV (PBCF) to 1.60 eV (PBCFIn05) and the subsequent *OH dehydrogenation step decreasing from 0.71 eV to 0.22 eV. These results collectively demonstrate that In-induced interfacial modulation accelerates oxygen-reaction kinetics and promotes efficient electrochemical activity.

Overall, the improved performance of PBCFIn05 arises from a cooperative mechanism of bulk and interfacial reconstruction. Moderate In^3+^ doping simultaneously (i) enhances bulk electronic conduction through spin-selective *e*_g_–*p*_σ_–*e*_g_ hopping, (ii) enriches oxygen-vacancy concentration for improved ion transport, and (iii) constructs a catalytically active Co_3_O_4_ interface that facilitates fast oxygen adsorption/desorption and stabilizes redox dynamics. These findings collectively establish a clear mechanistic link between atomic-scale structural modulation and macroscopic electrochemical functionality. Beyond the specific R-PCEC application, this work highlights a broader materials-design principle: controlled B-site doping coupled with in situ interface engineering can drive magnetic and electronic reconstruction to unlock superior bifunctional oxygen catalysis. Such a design strategy is extendable to other perovskite-spinel or perovskite-oxide systems, offering a versatile pathway to tailor mixed ionic-electronic conductors and oxygen electrodes for electrochemical energy technologies.

## Methods

### Materials synthesis and cell fabrication

The PBCFIn_x_ (*x* = 0, 0.05, 0.075) powders were prepared via an EDTA–citrate sol-gel complexation method. Pr(NO_3_)_3_·6H_2_O (≥99.9%), BaCO_3_ (≥99.9%), Co(NO_3_)_3_·6H_2_O (≥99.9%), Fe(NO_3_)_3_·9H_2_O (≥99.9%), and In(NO_3_)_3_ (≥99.9%) (Shanghai Macklin Biochemical Co., Ltd) were used as raw materials in stoichiometric proportions. The total cation:EDTA:citric acid molar ratio was fixed at 1:1:2. The mixed solution was magnetically stirred at 80 °C for 6 h until gelation occurred, followed by self-combustion to generate precursor ashes. These ashes were subsequently calcined in air at 1000 °C for 3 h, yielding phase-pure PBCF, PBCFIn05, and PBCFIn10 powders. The electrolyte BaZr_0.1_Ce_0.7_Y_0.1_Yb_0.1_O_3 − δ_ (BZCYYb) and the NiO-BZCYYb composite anode were synthesized using the same sol-gel route. For the anode functional layer (AFL), NiO and BZCYYb were blended at a mass ratio of 6:4, ball-milled for 24 h, and dried at 80 °C. The anode-supported half-cells were fabricated by sequential co-pressing. Specifically, 0.36 g NiO–BZCYYb powder was first pressed into pellets (20 mm diameter, ~1.5 mm thick) under 30 MPa. Subsequently, 0.014 g AFL powder and 0.005 g BZCYYb electrolyte powder were applied layer by layer and pressed at 200 MPa. The resulting green bodies were co-sintered in ambient air using a multistep heating program: the temperature was first increased to 200 °C at a heating rate corresponding to a 60 min ramp and held at 200 °C for 60 min to remove organic residues, followed by heating to 1350 °C over 350 min and holding at 1350 °C for 5 h. After natural cooling, dense electrolytes with porous anode backbones were obtained.

For cathode preparation, PBCF and In-doped PBCF powders were mixed with terpineol and 10 wt% ethyl cellulose for 2 h in an agate mortar to form homogeneous pastes. The slurry was then screen-printed onto the sintered electrolyte surface through a mask to define an active area of 0.196 cm^2^. After drying at 80 °C, the cathode-coated cells were calcined in ambient air following a multistep heating program: the temperature was first ramped to 200 °C within 30 min and held for 60 min, then increased to 1000 °C over 330 min, followed by a dwell at 1000 °C for 3 h to achieve robust interfacial bonding between the cathode and electrolyte.

### Basic characterizations

The phase structure was analyzed using an X-ray diffractometer (D8 Advance, Bruker) with Cu Kα radiation. The surface chemical states of the PBCF, PBCFIn05, and PBCFIn10 cathode powders were examined using X-ray photoelectron spectroscopy (XPS; Thermo Scientific K-Alpha). Transmission electron microscopy (TEM; JEOL JEM2100F) was employed to assess the morphology and crystalline of the samples, while scanning electron microscopy (SEM; ZEISS Sigma 300) was used to investigate the microstructure of the cells. Thermogravimetric analysis (TGA; PerkinElmer STA 6000) was performed to monitor the mass changes of the samples during heating. H_2_O–TPD data were obtained using a mass spectrometer (Micromeritics AutoChem II 2920), with H_2_O–TPD data acquired after pre-treating the powders in humid air at 600 °C for 10 h. The proton concentration in hydrated samples was determined by Karl Fischer titration through quantification of thermally released water originating from protonic species, using a Karl Fischer moisture analyzer (MKC-710, Kyoto Electronics). After hydration at 600 °C for 24 h in humidified Ar–20% O_2_–10% H_2_O (P_H2O_ = 0.10), dense bulk samples were heated to 1000 °C, and the released water was carried by dry N_2_ into the KF titration cell. The water content was quantified electrochemically based on the iodide/iodine redox reaction and further converted to proton uptake.

Electron paramagnetic resonance (EPR; Bruker E500, Germany) operating at 9 GHz with a modulation frequency of 100 kHz was used to compare the concentration of oxygen vacancies in the samples. Inductively coupled plasma optical emission spectrometry (ICP-OES) was performed on Agilent 5110 instrument. The X-ray absorption spectra of O *K*-edge, Co K-edge, and Fe *K*-edge were measured on the sample at beamline 20 A of Shanghai Light Source (TLS). The hysteresis loop test was performed using a vibrating sample magnetometer (VSM, LakeShore 8604) with a field strength range of 2 T. The relationship between magnetic susceptibility and temperature 5 of the material was tested using a physical property measurement system (PPMS, Quantum Design, USA) to obtain zero-field-cooling and field-cooling curves. X-ray absorption spectroscopy (XAS) data were processed and analyzed using the Demeter software package^1^. A linear function was subtracted from the pre-edge region, and then the edge jump was normalized using Athena software. The *χ*(*k*) data were isolated by subtracting a smooth polynomial approximating the absorption background of an isolated atom. The *k*^3^–weighted *χ*(*k*) data were Fourier transformed after applying a Hanning window function (Δ*k* = 1).

### Electrochemical measurements

Following sample preparation, a series of electrochemical measurements were carried out to assess their properties and applicability. Single cells were assembled with silver paste and connected to custom-made fixtures for testing. For fuel cell evaluation, humidified H_2_ (3% H_2_O) was fed to the fuel electrode at 40 mL min^–1^, while ambient air was supplied to the cathode side. Electrolysis testing employed the same H_2_–H_2_O feed at the anode, with the cathode exposed to humidified air (40 mL min^–1^). All electrochemical characterizations were performed using a Naval Admiral workstation. Current–voltage (*I*–*V*) and power density (*I*–*P*) characteristics were obtained under linear sweep voltammetry, and EIS was performed under open-circuit conditions to probe polarization and dynamic responses. EIS spectra were recorded between 0.1 Hz and 1 MHz with amplitude of 10 mV. FE was determined by comparing the actual hydrogen evolution rate, measured by gas chromatography, with the theoretical production at a given current density. Hydrogen output was monitored under both OCV and steady-current electrolysis. During these tests, 10 mL min^–1^ H_2_ and 40 ml min^–1^ N_2_ were delivered to the anode, while the cathode received humidified air at 40 ml min^–1^. Each data set was collected in triplicate to ensure reproducibility and to allow statistical averaging. All electrochemical data were performed using an Admiral Instruments workstation, and the data were collected and processed using Squidstat software. All electrochemical measurements and cell cycling tests were carried out under ambient laboratory conditions at approximately 25 °C, without the use of a climatic/environmental chamber.

### Computational methods

All first-principles calculations were performed using DFT with the projector augmented wave (PAW) method, as implemented in the Vienna Ab initio Simulation Package. Exchange–correlation effects were treated with the Perdew–Burke–Ernzerhof functional under the generalized gradient approximation, while electron–ion interactions were described by PAW potentials^86^. The plane-wave basis set was truncated at 520 eV, and Brillouin zone integration employed Monkhorst–Pack meshes of 3 × 4 × 2 for bulk models and 2 × 2 × 1 for slab models. For structural relaxations, atomic positions were optimized until residual forces were less than 0.02 eV/Å. In surface calculations, only the upper four layers were allowed to relax, while deeper layers were fixed. The electronic structures were further analyzed by calculating the total and projected density of states (DOS/PDOS) from self-consistent results to resolve atomic orbital contributions. The most favorable doping positions and H_2_O adsorption sites were identified by comparing the total energies of several candidate configurations. Reaction pathways were investigated using the climbing-image nudged elastic band (CI-NEB) approach with six intermediate images, and transition states were confirmed by vibrational frequency analysis, ensuring a single imaginary mode^87^. Long-range dispersion interactions were included through the DFT–D3 correction scheme^88^. Defect properties were studied by calculating oxygen vacancy formation energies relative to pristine structures and the reference O_2_ molecule. Hydration energies were determined from the total energy difference between hydrated and defective configurations, with an isolated H_2_O molecule in vacuum as the reference.

## Supplementary information

Supplementary Information

Description of Additional Supplementary Files

Supplementary Data 1–23

Transparent Peer Review File

## Source data

Source Data

## Data Availability

All data needed to evaluate the conclusions in the paper are present in the paper and/or the Supplementary Information. All other data supporting the findings of the study are available from the corresponding authors if asked for. Source data are provided with this paper.
