# Supplementary material for: Spin-coordinated mixed-valence transport via indium-induced perovskite-spinel heterointerface in oxygen electrode for reversible protonic ceramic cells
Source: Nat Commun. 2026 Jul 15;17:8686. doi: 10.1038/s41467-026-75684-4 (PMC13490552; doi:10.1038/s41467-026-75684-4)
Supplement: Supplementary file 1 [file 41467_2026_75684_MOESM1_ESM.pdf]

Supplementary Information

Spin-coordinated mixed-valence transport via Indium-induced perovskite-spinel heterointerface in oxygen electrode for reversible protonic ceramic cells

Yinlin Chang[1*], Tao Liu[1*], Jingyuan Rao[1*], Shuanglin Zheng[2], Min Fu[1], Hanping Ding[2,#], Zetian Tao[1,#]

[1] School of Resources, Environment and Safety Engineering, University of South China, Hengyang, Hunan Province 421001, China

[2] School of Aerospace and Mechanical Engineering, University of Oklahoma, Norman, OK 73019 USA.

[*] Equal contribution
[#] Corresponding author
Email address: hding@ou.edu (H. Ding), taozetian@usc.edu.cn (Z. Tao).

Calculation Information

Calculation of the effective moments (μ_{eff}) and the unpaired electrons (n):

According to the Curie-Weiss law, magnetic susceptibility is inversely proportional to temperature:

$$\chi = \frac{C}{T} = \frac{N^2 g^2 \beta^2 \mu_B S(S+1)}{3kT} \quad (1)$$

Where $\frac{N^2\beta^2}{3k}$ is the constant 0.125, μ_B is the Bohr magneton, S is the spin state, T is temperature, g is the Landé g-factor.

$$\mu_{eff} = \sqrt{[g^2 S(S+1)]\mu_B} \quad (2)$$

μ_{eff} is the effective magnetic moment.

A direct relationship (equation 3) between μ_{eff} and magnetic susceptibility and temperature can be derived by combining equations 1 and 2.

$$\mu_{eff} = \sqrt{8 \times T \mu_B} \quad (3)$$

Then, the number of unpaired electron (n) can be calculated through the following equation:

$$\mu_{eff} = \sqrt{n(n+2) \mu_B} \quad (4)$$

Faradaic efficiency measurement

Faradaic efficiency (FE) was determined by comparing the electrochemically generated hydrogen rate with the theoretical hydrogen production rate at a given current density. The hydrogen evolution rate was quantified by gas chromatography (GC) under SOEC operation. During the measurements, a sweep gas consisting of 10% H_2/90% Ar at a total flow rate of 40 mL min^{-1} was supplied to the hydrogen electrode, while the PBCFIn05 electrode operated as

the oxygen electrode and was fed with humidified air (40 mL min^{-1}). GC analysis was used to determine the outlet H2 concentration, and the electrochemically produced hydrogen rate was calculated from the increase in H_2 concentration relative to the inlet sweep gas under steady-state conditions. The FE was then calculated by comparing this experimentally determined hydrogen production rate with the theoretical value derived from the applied current. Hydrogen output was monitored under both open-circuit voltage (OCV) and steady-current electrolysis conditions. Each data set was collected in triplicate, and the corresponding mean values and standard deviations were used to assess reproducibility.

The Faradaic efficiency (FE) was calculated using:

$$v=\frac{V_m}{2F}\times i$$

$$FE(\%)=\frac{v_e}{v}\times 100\%$$

Where v is the theoretical hydrogen production flow rate (standard cubic centimeter per minute, sccm), i is the input current (A), F is the Faraday constant (9.6485×10^4 C mol^{-1}); and V_m is the molar volume of a gas (22,400 mL mol^{-1}). The experimental hydrogen production flow rate v_e.

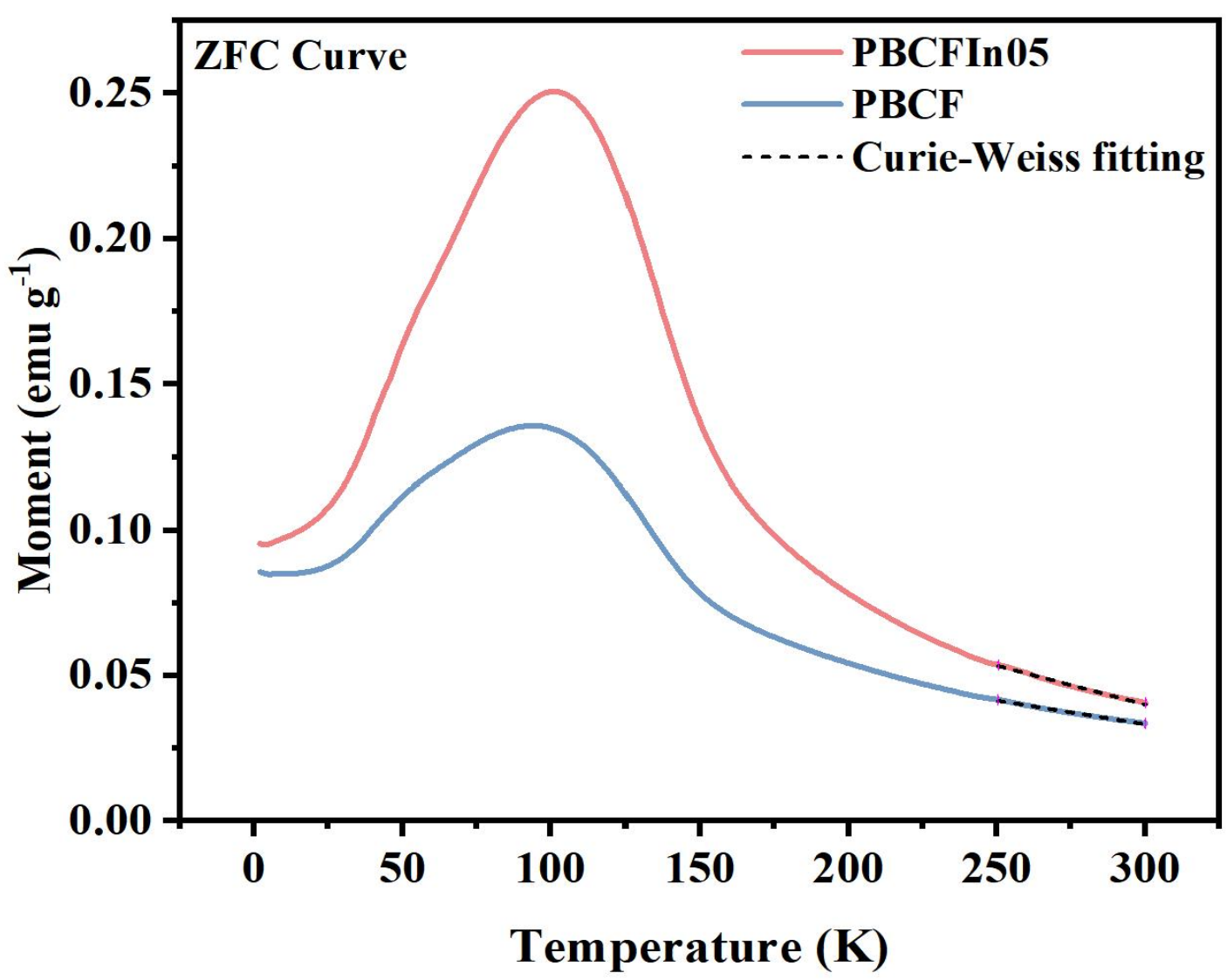

Supplementary Fig. 1. Zero-field-cooled (ZFC) magnetization curves of PBCF and PBCFIn05 measured under 1000 Oe in the temperature range of 5-300 K. Both samples display broad maxima around 90-110 K, indicative of magnetic anomalies consistent with the transitions discussed in the main text. The more pronounced peak intensity in PBCFIn05 suggests an increased density of spin-active clusters and enhanced local magnetic interactions. The dashed lines represent Curie–Weiss fitting performed in the high-temperature paramagnetic region (250-300 K). Source data for Supplementary Fig. 1 are provided as a Source Data file.

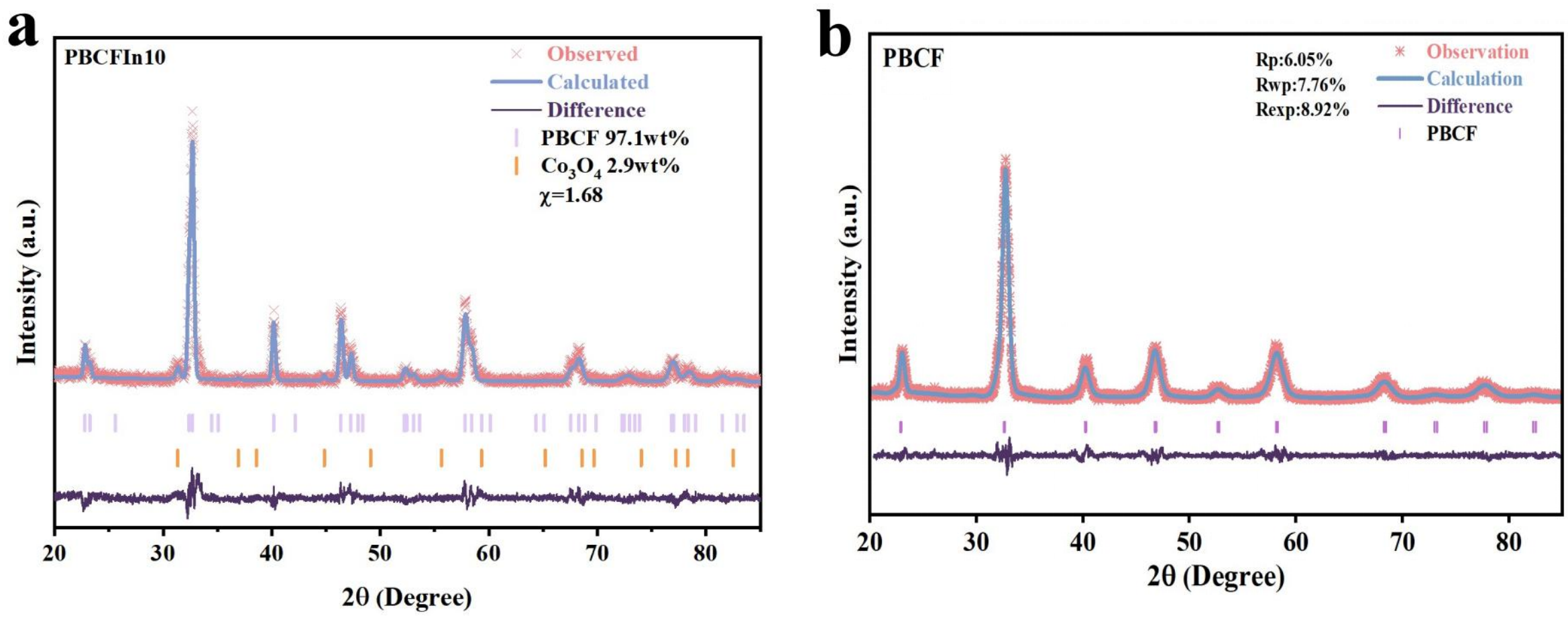

Supplementary Fig. 2. XRD refinements of as-synthesized PBCF and PBCFIn10 powder. The diffraction peaks of pristine PBCF can be indexed to a single-phase perovskite structure. In contrast, PBCFIn10 shows additional reflections attributable to Co_3O_4, indicating partial phase segregation induced by excess indium incorporation at higher nominal In content. The main perovskite framework is retained, while the Co_3O_4 phase appears as a secondary component. Source data for Supplementary Fig. 2 are provided as a Source Data file.

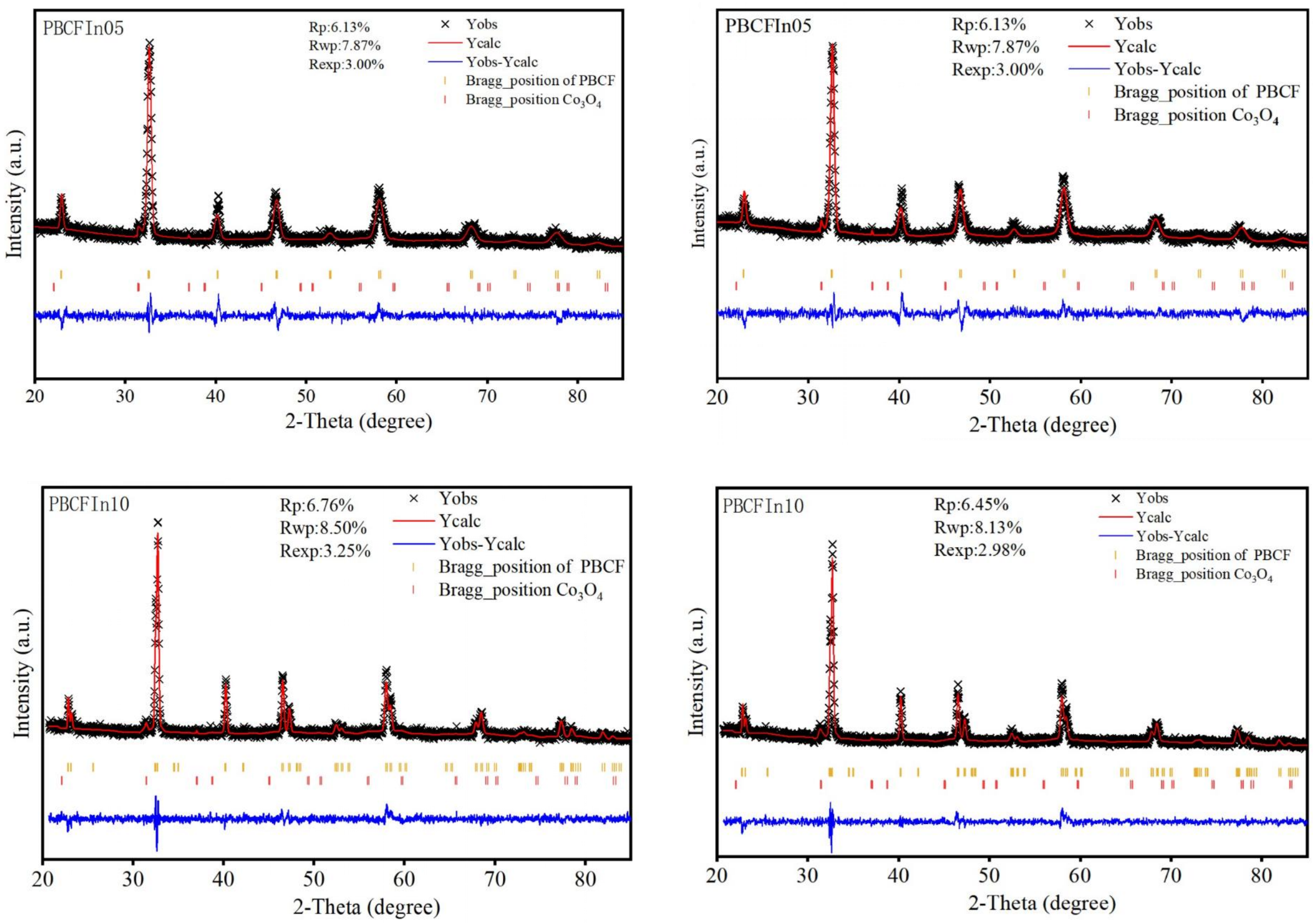

Supplementary Fig. 3. Rietveld refinement results of XRD patterns collected from independently synthesized batches of PBCFIn05 and PBCFIn10. Observed (Y_{obs}), calculated (Y_{calc}), and difference (Y_{obs}-Y_{calc}) profiles are shown together with the Bragg positions of the perovskite PBCF and spinel Co_3O_4 phases, demonstrating the reproducibility of the refinement results. Source data for Supplementary Fig. 3 are provided as a Source Data file.

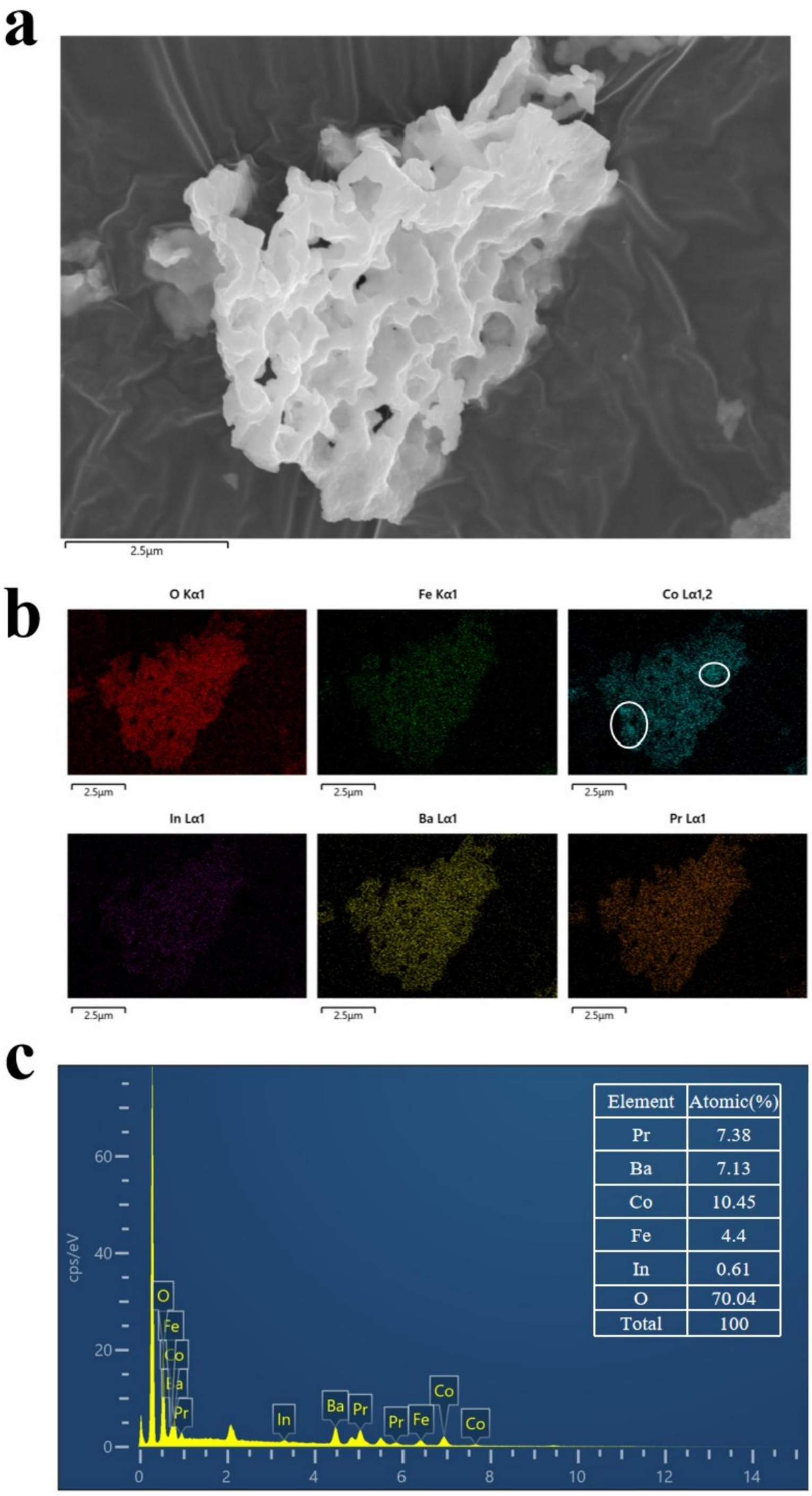

Supplementary Fig. 4. (a) SEM image of the as-synthesized PBCFIn05 powder showing porous and agglomerated morphology. (b) Corresponding EDS elemental mapping of O, Fe, Co, In, Ba, and Pr, confirming the homogeneous distribution of all cations across the particle. Local Co-enriched regions (circled) suggest partial surface segregation. (c) EDS spectrum and quantified atomic ratios, showing the presence of Pr, Ba, Co, Fe, In, and O with overall stoichiometry consistent with the nominal PBCFIn05 composition. Source data for Supplementary Fig. 4 are provided as a Source Data file.

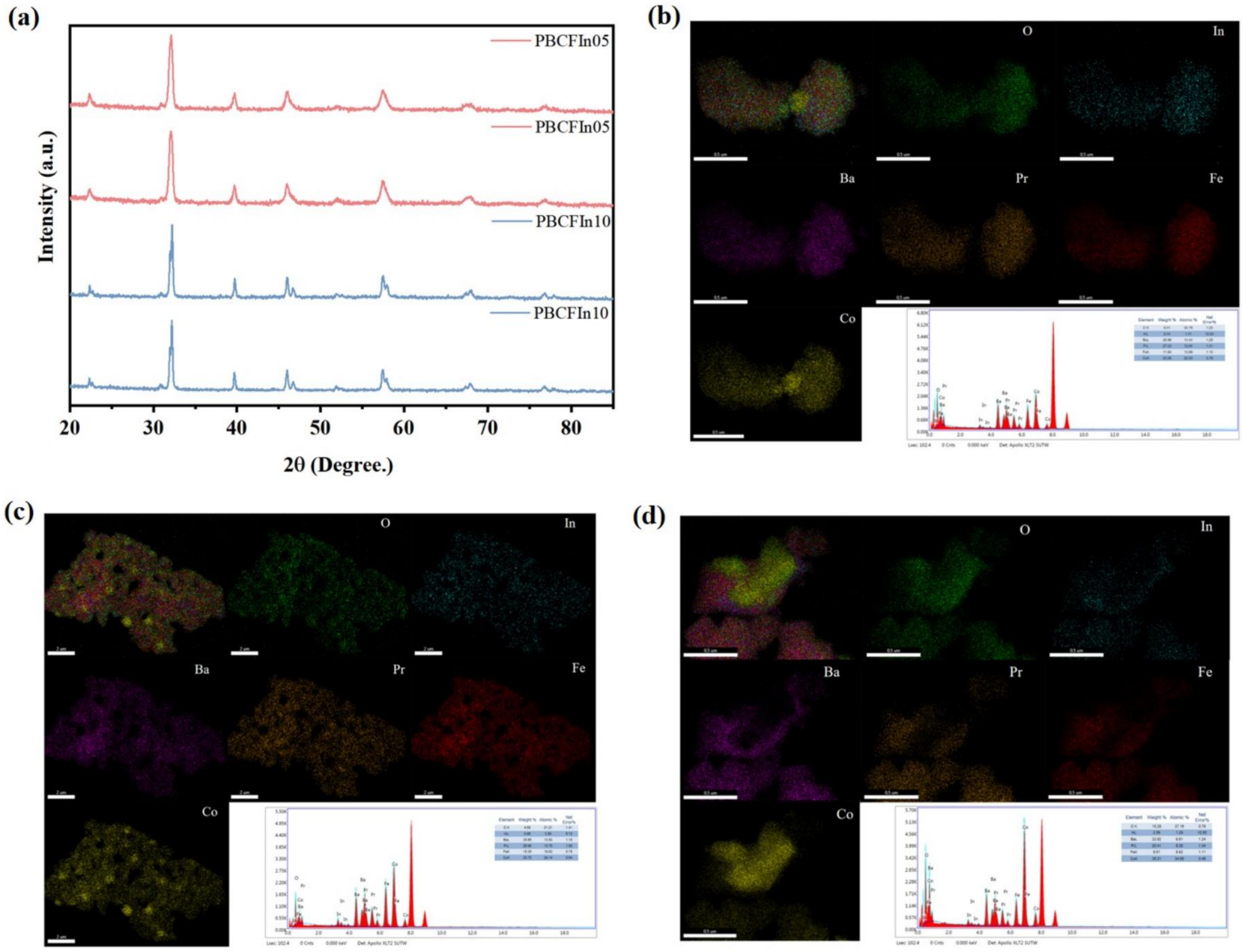

Supplementary Fig. 5. Reproducibility and spatial distribution of surface Co_3O_4 domains in PBCFIn samples. (a) XRD patterns of independently synthesized PBCFIn05 and PBCFIn10 samples, confirming the reproducibility of the phase composition and the emergence of a minor Co_3O_4 phase upon In doping, (b-d) STEM-EDS elemental mappings of PBCFIn05 collected from different spatial regions. Co shows a non-continuous, localized surface distribution rather than a uniform layer. Source data for Supplementary Fig. 5 are provided as a Source Data file.

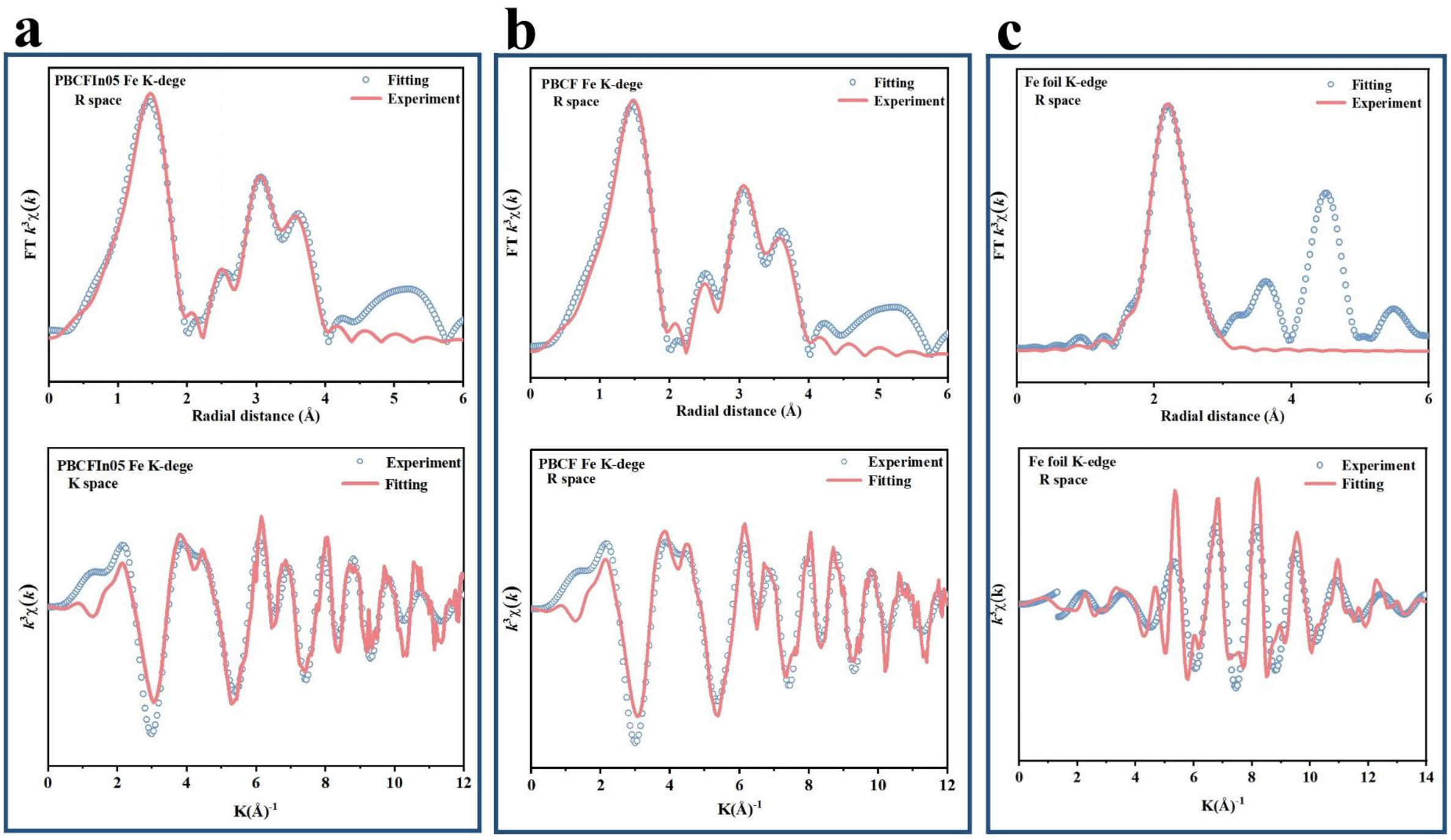

Supplementary Fig. 6. EXAFS information of Fe in PBCF and PBCFIn05 (a) Fourier transformed k^3 -weighted EXAFS spectra (R space) and EXAFS spectra in K space of Fe in PBCFIn05, (b) Fourier transformed k^3 -weighted EXAFS spectra (R space) and EXAFS spectra in K space of Fe in PBCF, (c) Fourier transformed k^3-weighted EXAFS spectra (R space) and EXAFS spectra in K space of Fe foil. Source data for Supplementary Fig. 6 are provided as a Source Data file.

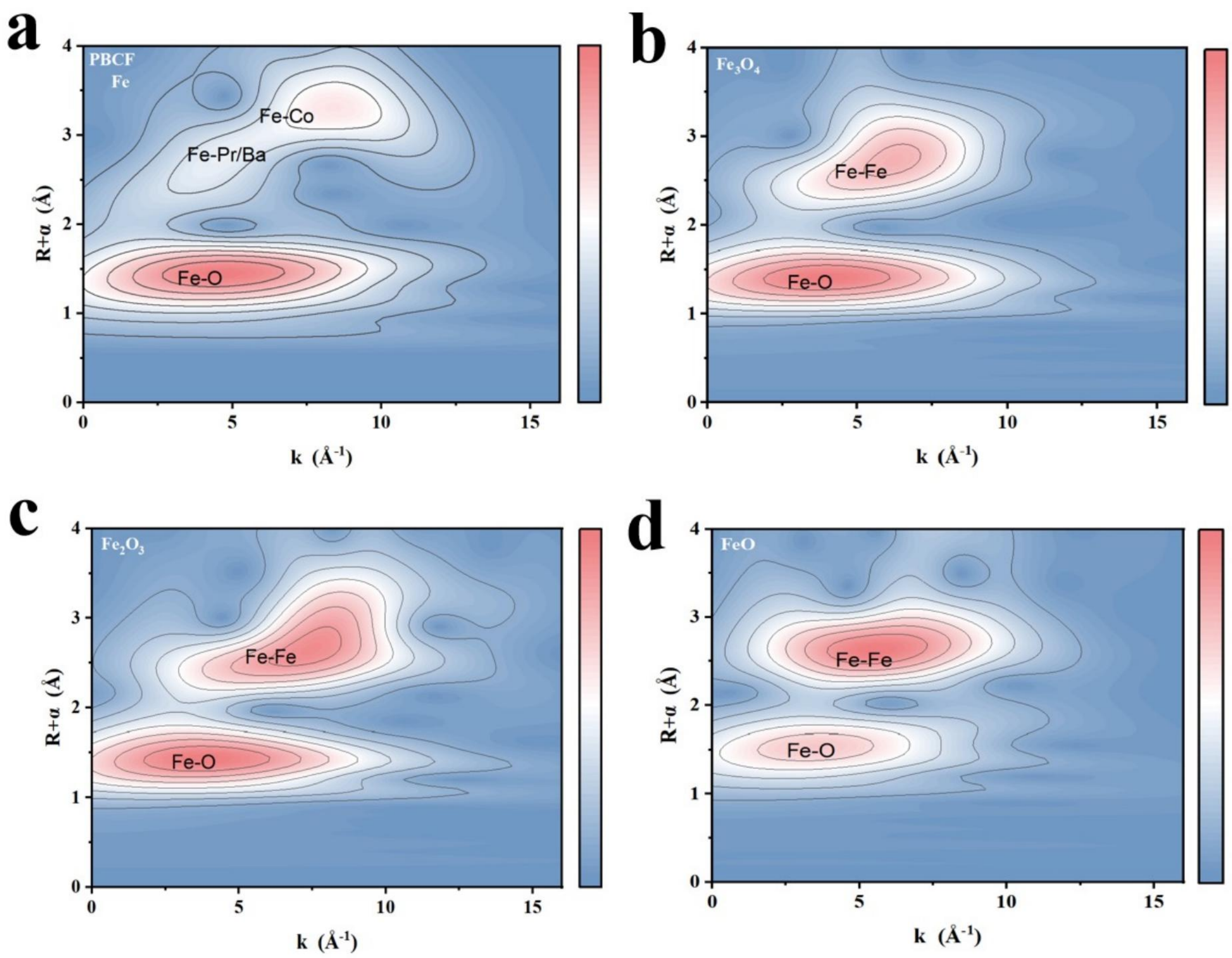

Supplementary Fig. 7. Wavelet transforms of the k^3-weighted EXAFS spectra of (a) PBCF, (b) Fe_3O_4, (c) Fe_2O_3, and (d) FeO.

For Wavelet Transform analysis, the χ(k) exported from Athena was imported into the Hama Fortran code. The parameters were listed as follow: R range, 0 - 4 Å; k range, 0 -16 Å-1; k weight, 3; and Morlet function with κ=10, σ=1 was used as the mother wavelet to provide the overall distribution[1, 2]. Source data for Supplementary Fig. 7 are provided as a Source Data file.

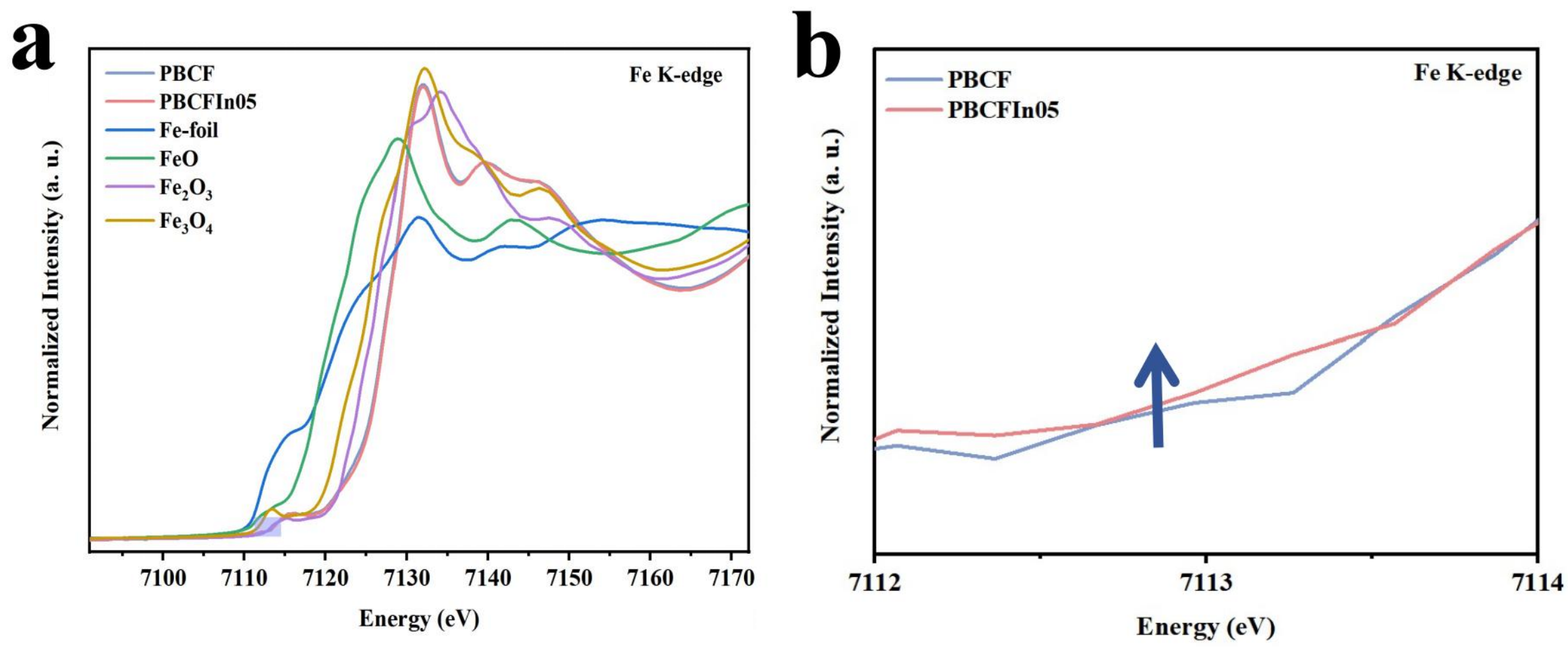

Supplementary Fig. 8. XANES information of Fe K-edge of PBCF and PBCFIn05. (a) Normalized Fe K-edge XAS of PBCF and PBCFIn05; (b) Enlarged Fe K-edge-pred region of PBCF and PBCFIn05, extracted from Fig. S8 a. Source data for Supplementary Fig. 8 are provided as a Source Data file.

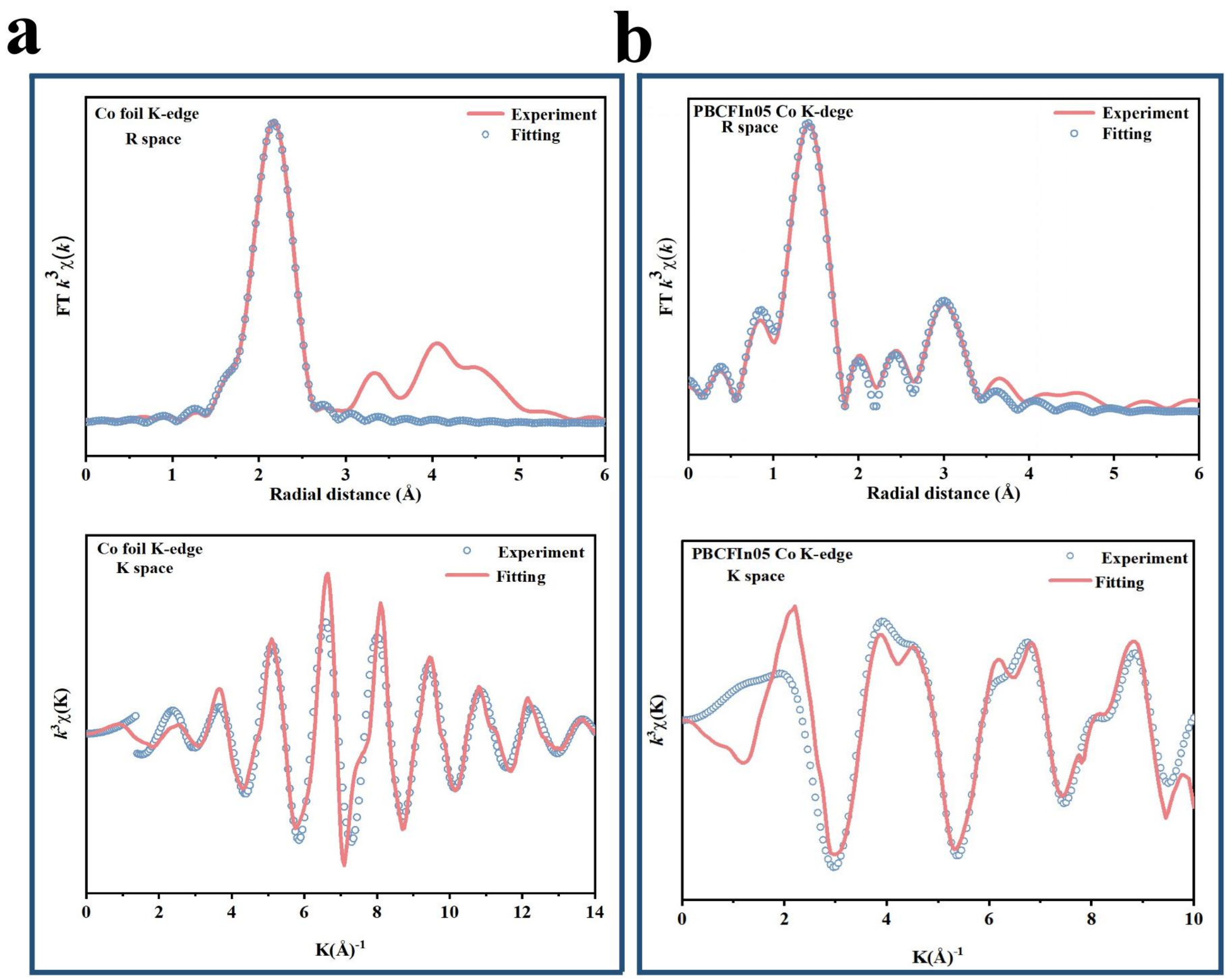

Supplementary Fig. 9. (a) Fourier transformed k^3-weighted EXAFS spectra (R space) and EXAFS spectra in K space of Co foil. (b) Fourier transformed k^3-weighted EXAFS spectra (R space) and EXAFS spectra in K space of Co in PBCFIn05.Source data for Supplementary Fig. 9 are provided as a Source Data file.

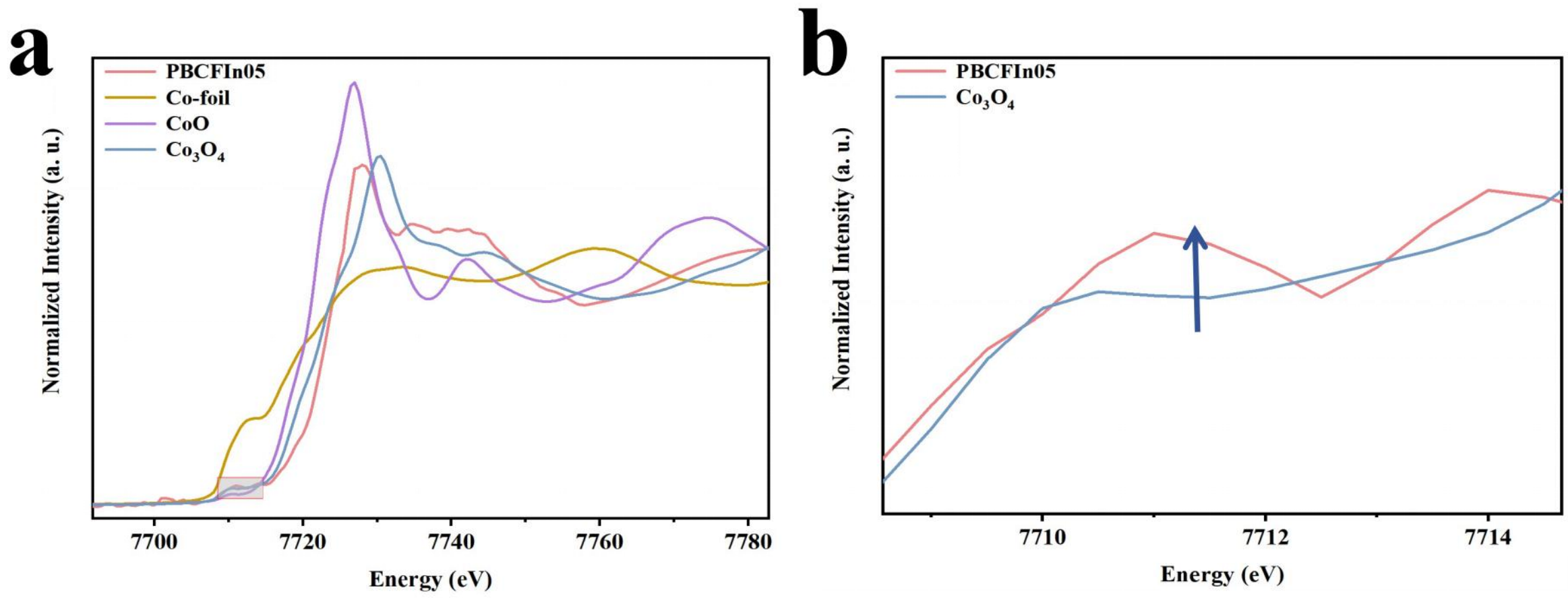

Supplementary Fig. 10. XANES information of Co K-edge of PBCFIn05. (a) Normalized Co K-edge XAS of PBCFIn05; (b) Enlarged Co K-edge-pred region of PBCFIn05, extracted from Fig. S11a. Source data for Supplementary Fig. 10 are provided as a Source Data file.

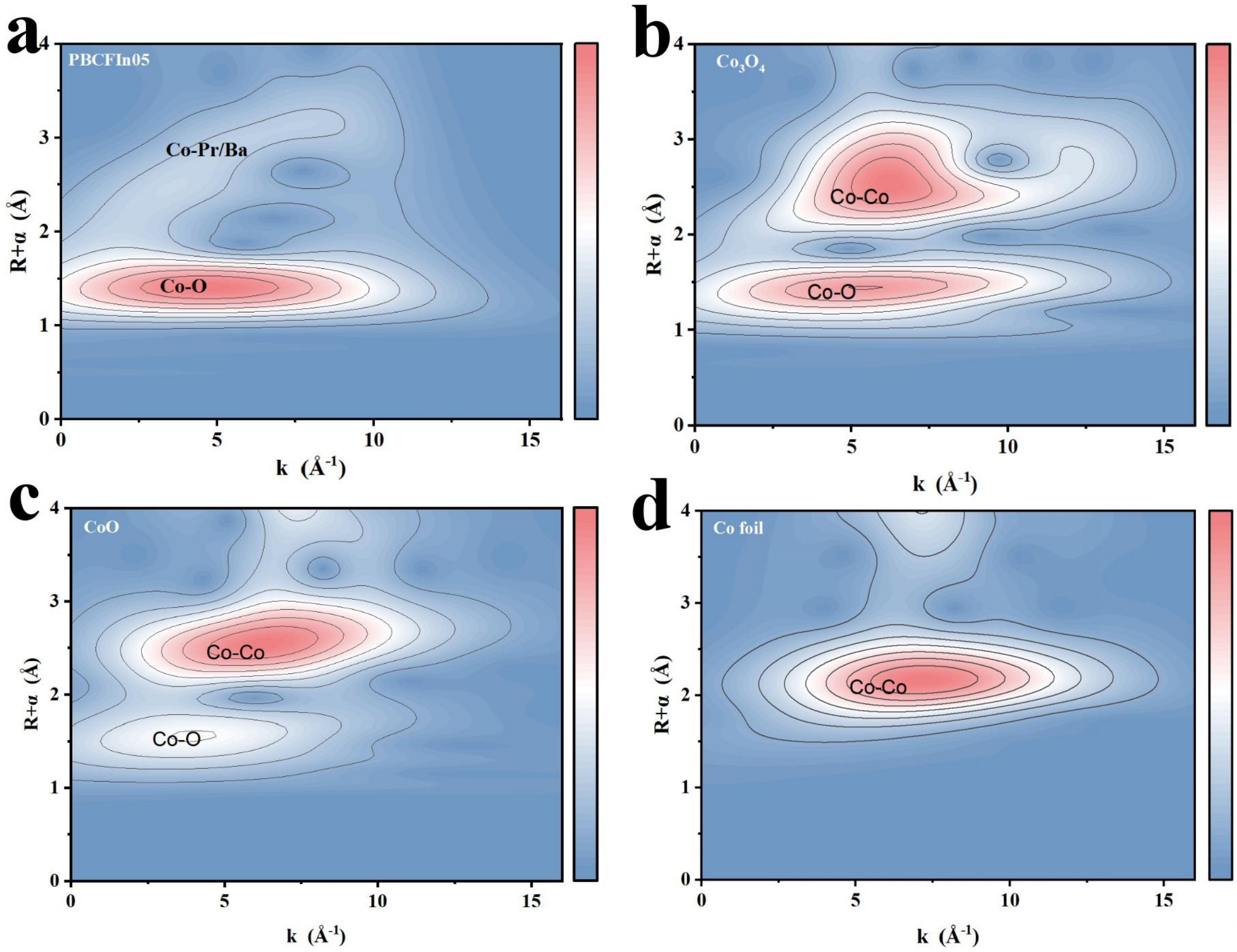

Supplementary Fig. 11. Wavelet transforms of the k^3-weighted EXAFS spectra of (a) PBCFIn05, (b) C_3O_4, (c) CoO, and (d) Co foil.

For Wavelet Transform analysis, the χ(k) exported from Athena was imported into the Hama Fortran code. The parameters were listed as follow: R range, 0 - 4 Å; k range, 0 - 16 Å^{-1}; k weight, 3; and Morlet function with κ=10, σ=1 was used as the mother wavelet to provide the overall distribution. Source data for Supplementary Fig. 11 are provided as a Source Data file.

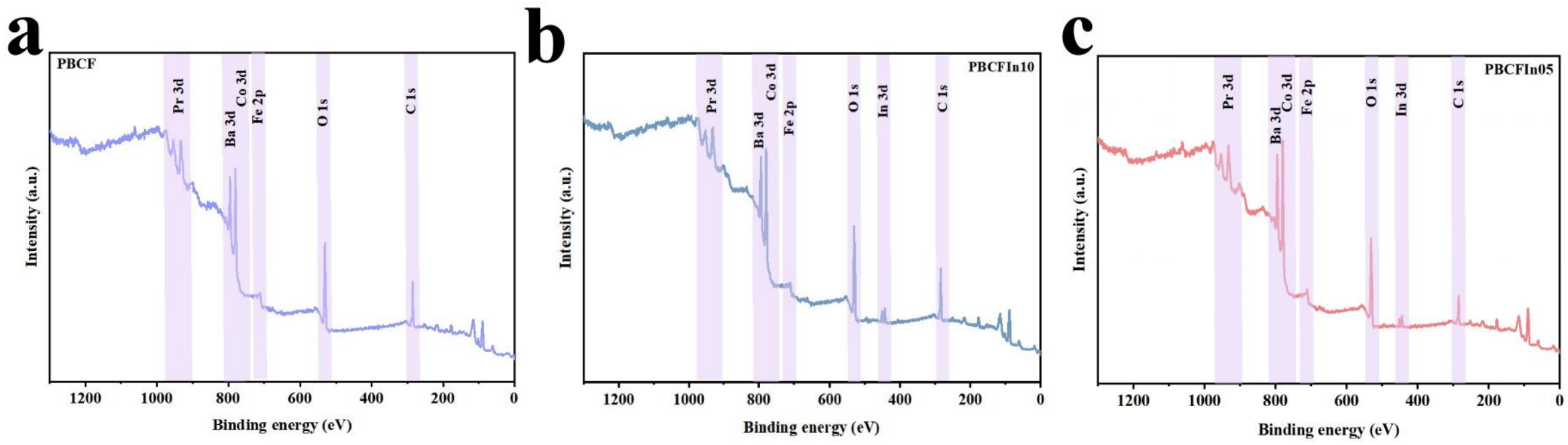

Supplementary Fig. 12. XPS of PBCF, PBCFIn05 and PBCFIn10. (a-c) Survey spectra of PBCF, PBCFIn05 and PBCFIn10. Source data for Supplementary Fig. 12 are provided as a Source Data file.

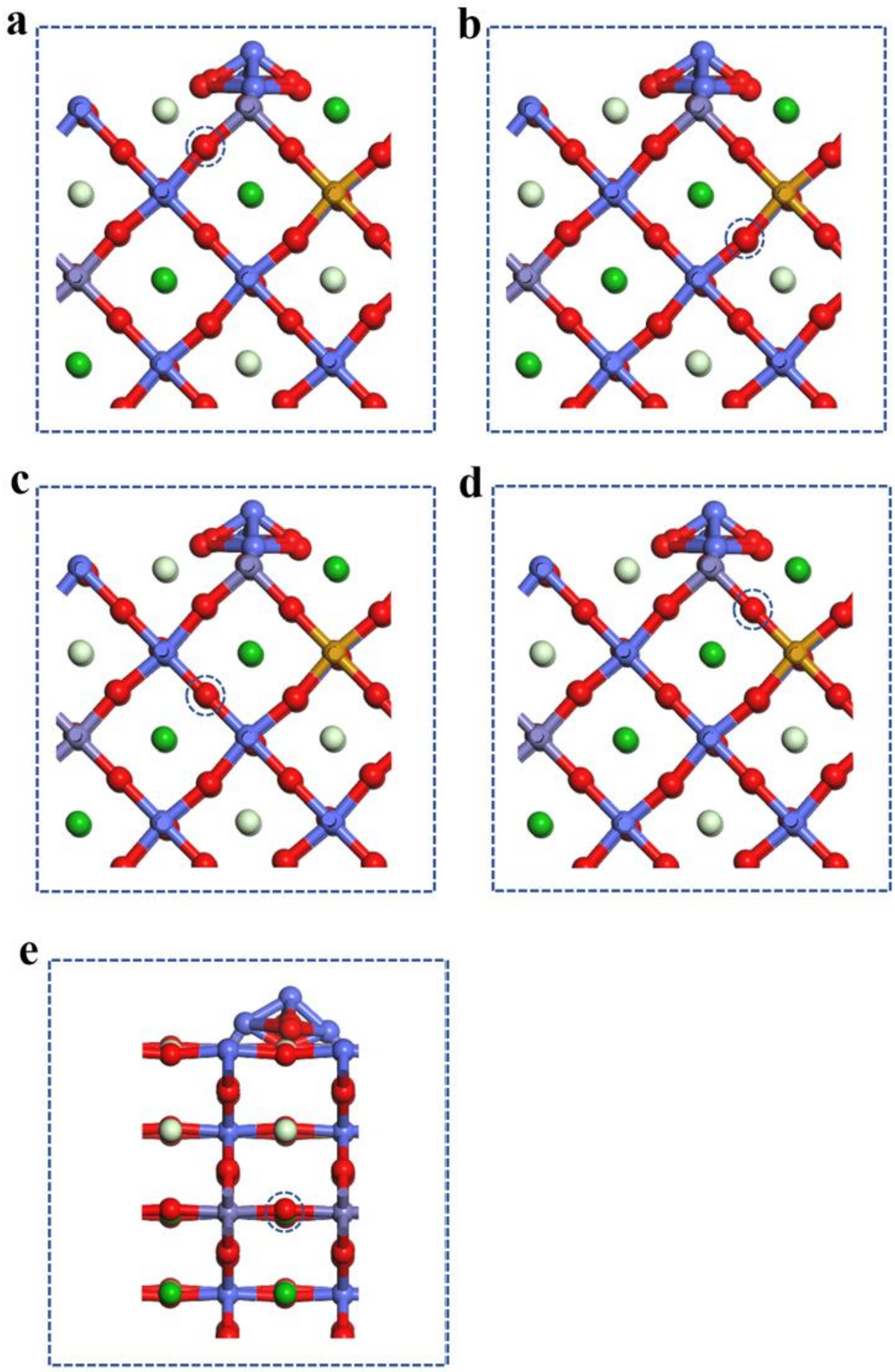

Supplementary Fig. 13. The adsorption geometries of oxygen vacancies at different lattice sites in PBCFIn05. (a) Fe-O-Co, (b) In-O-Co, (c) CO-O-Co, (d) Fe-O-In, (e) Fe-O-Fe. Source data for Supplementary Fig. 13 are provided as a Supplementary data 1 file.

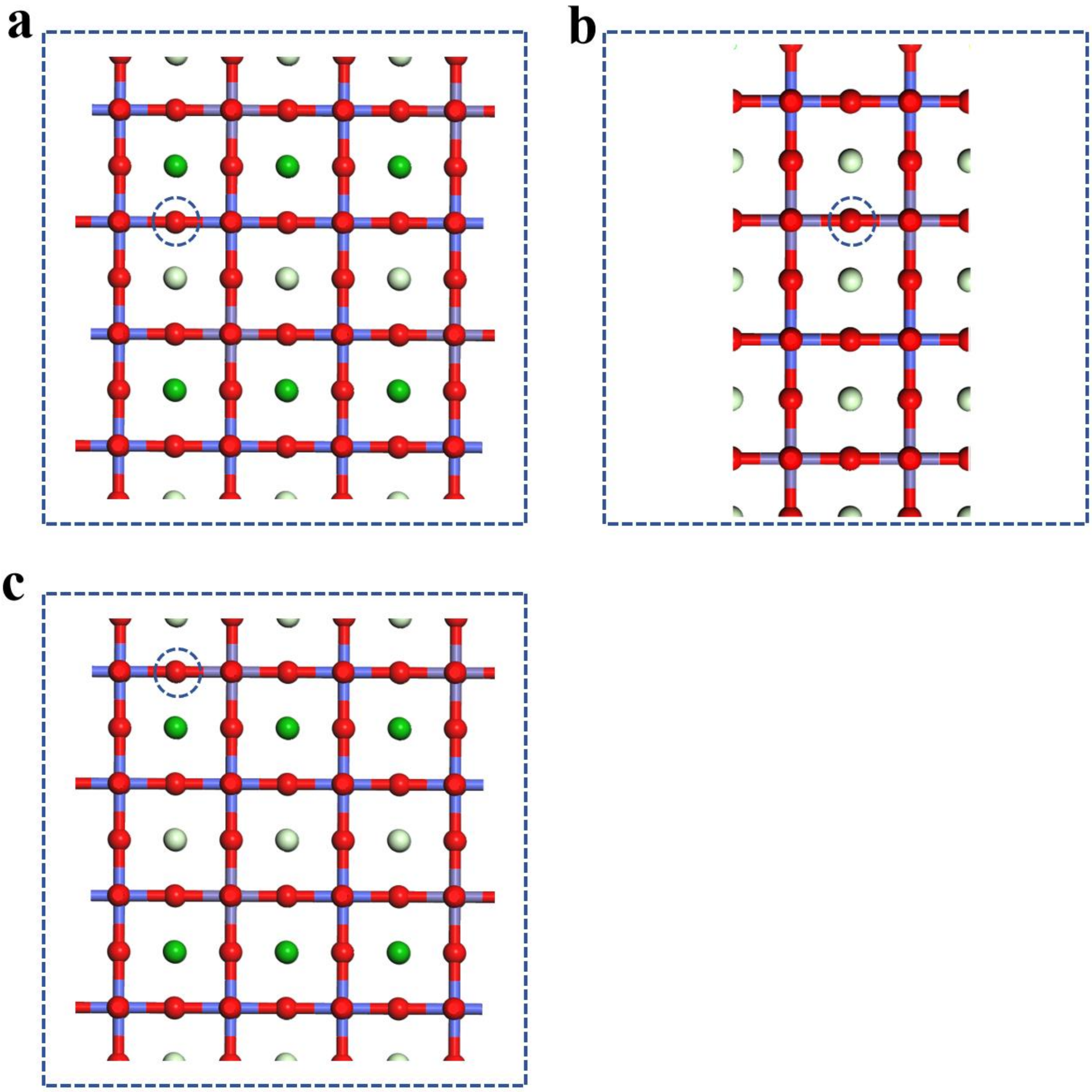

Supplementary Fig. 14. The adsorption geometries of oxygen vacancies at different lattice sites in PBCFIn05. (a) Co-O-Co, (b) Fe-O-Fe, (c) Fe-O-Co. Source data for Supplementary Fig. 14 are provided as a Supplementary data 1 file..

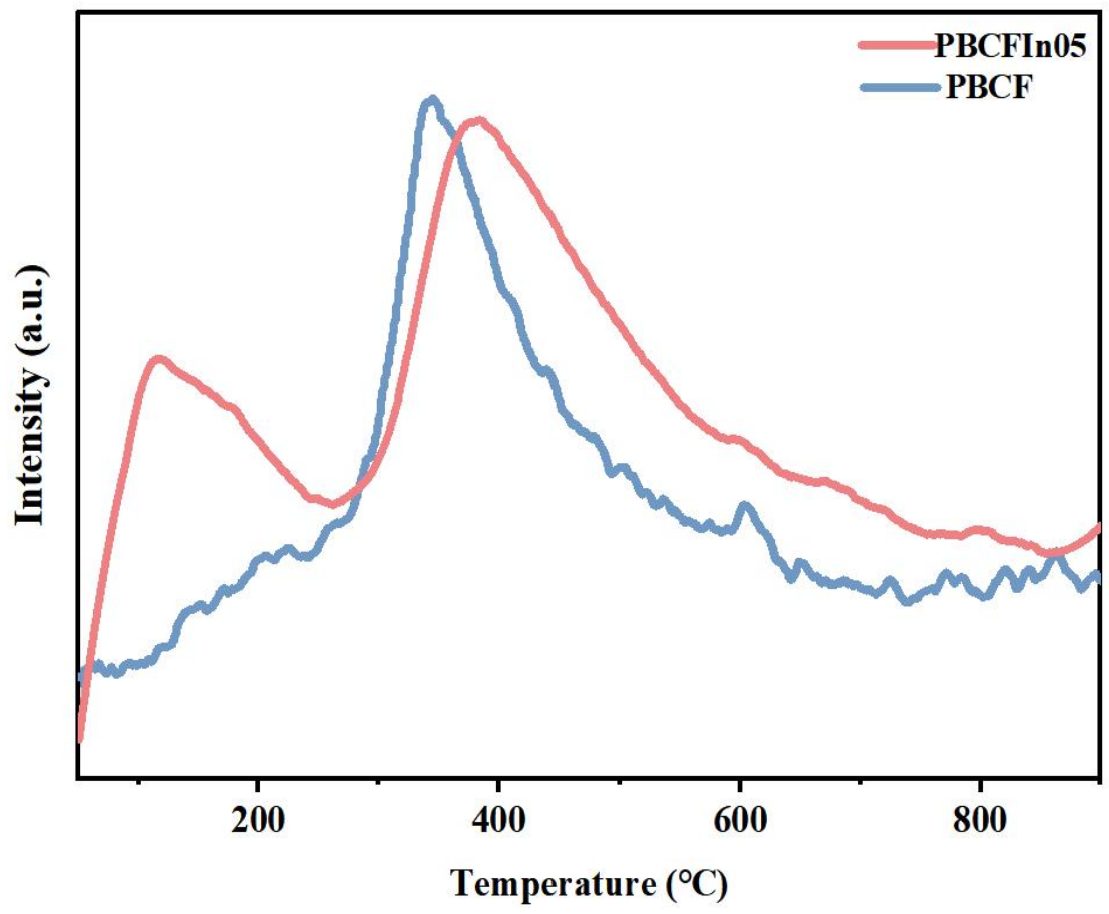

Supplementary Fig. 15. H_2O-TPD profiles from room temperature to 900 ℃. Source data for Supplementary Fig. 15 are provided as a Source Data file.

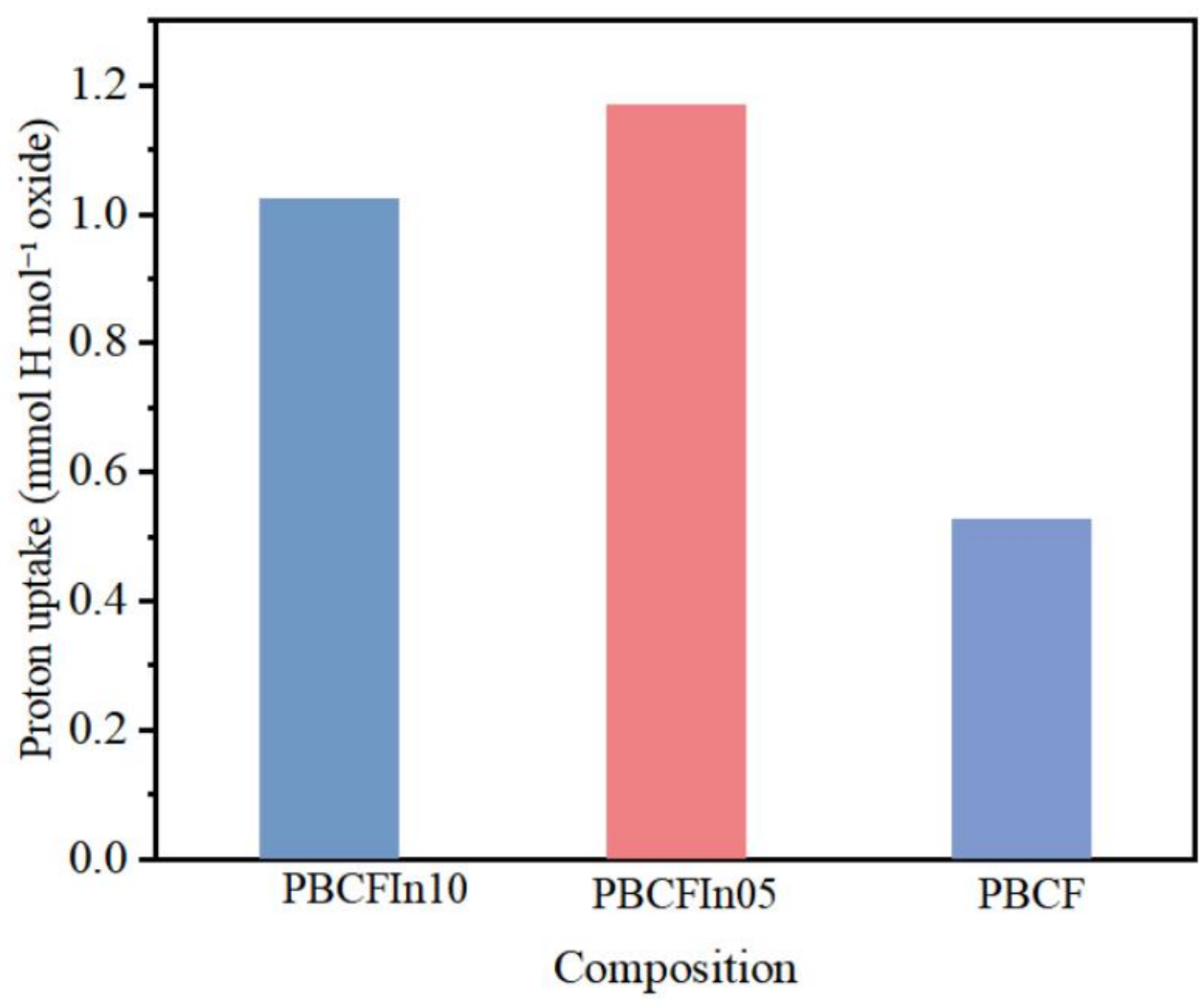

Supplementary Fig. 16. Proton uptake of PBCF， PBCFIn05 and PBCFIn10 samples measured by Karl Fischer titration. Source data for Supplementary Fig. 16 are provided as a Source Data file.

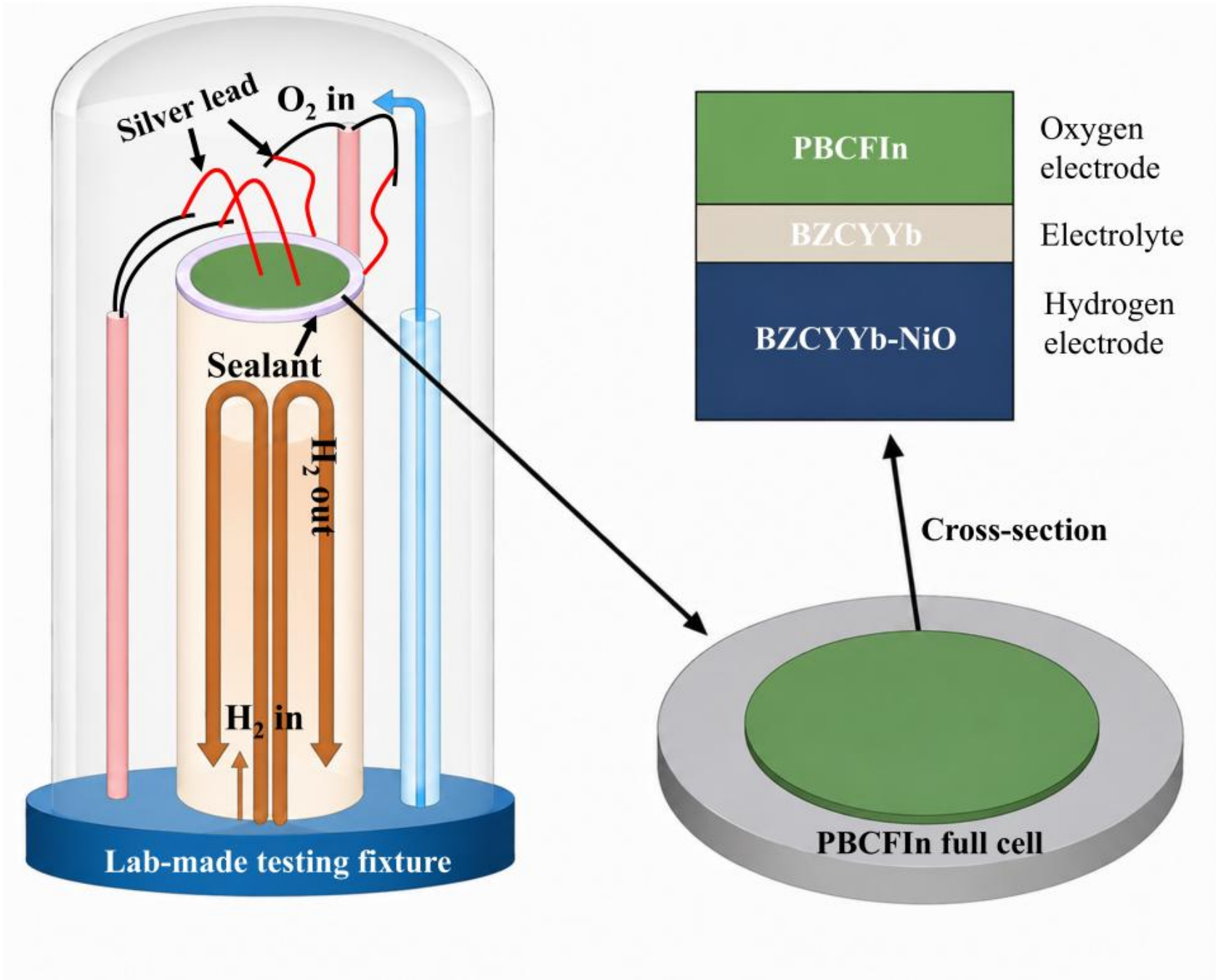

Supplementary Fig. 17. Lab-made testing fixture used in this work.

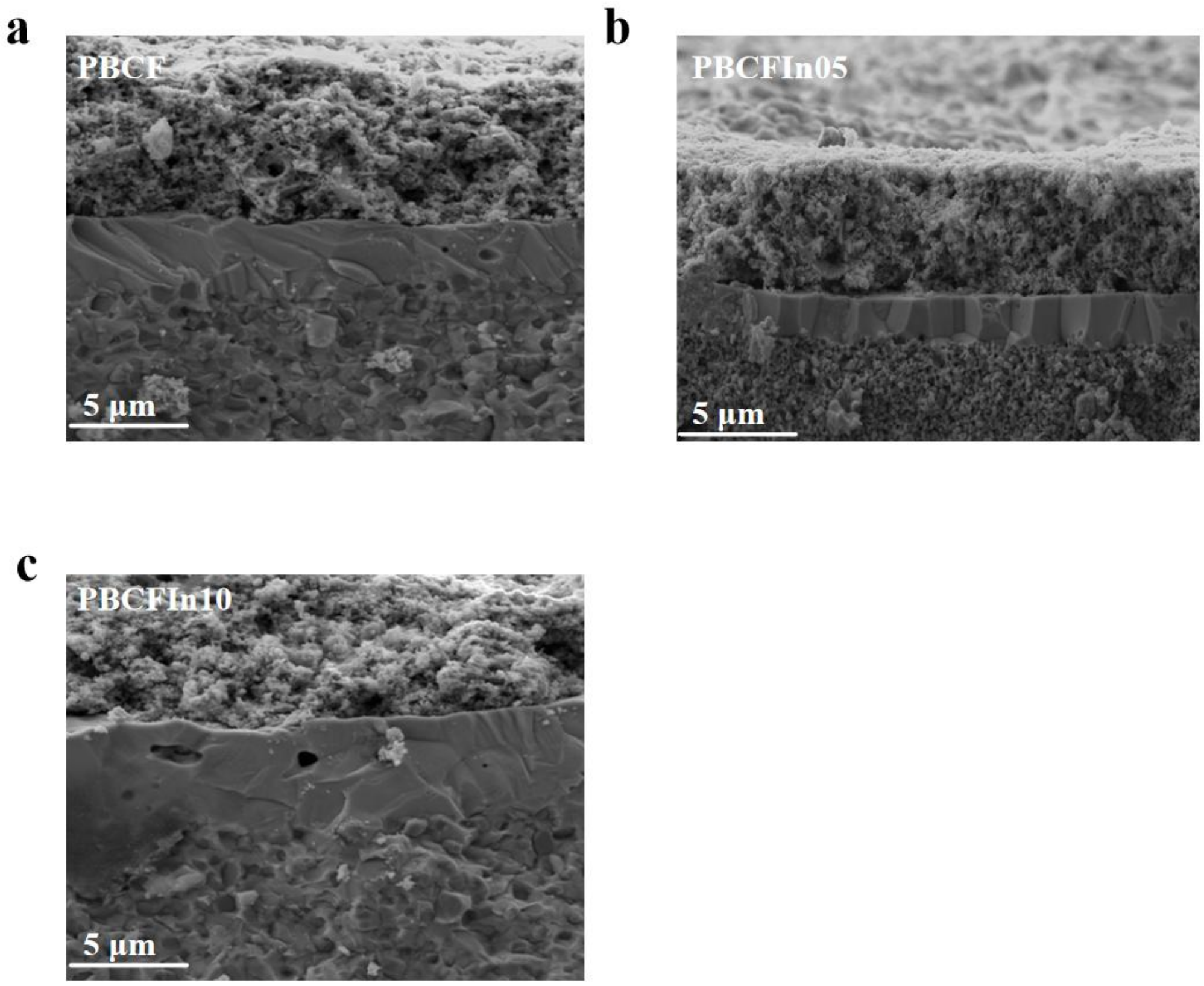

Supplementary Fig. 18. SEM image of PBCF, PBCFIn05 and PBCFIn10 single cell structure.

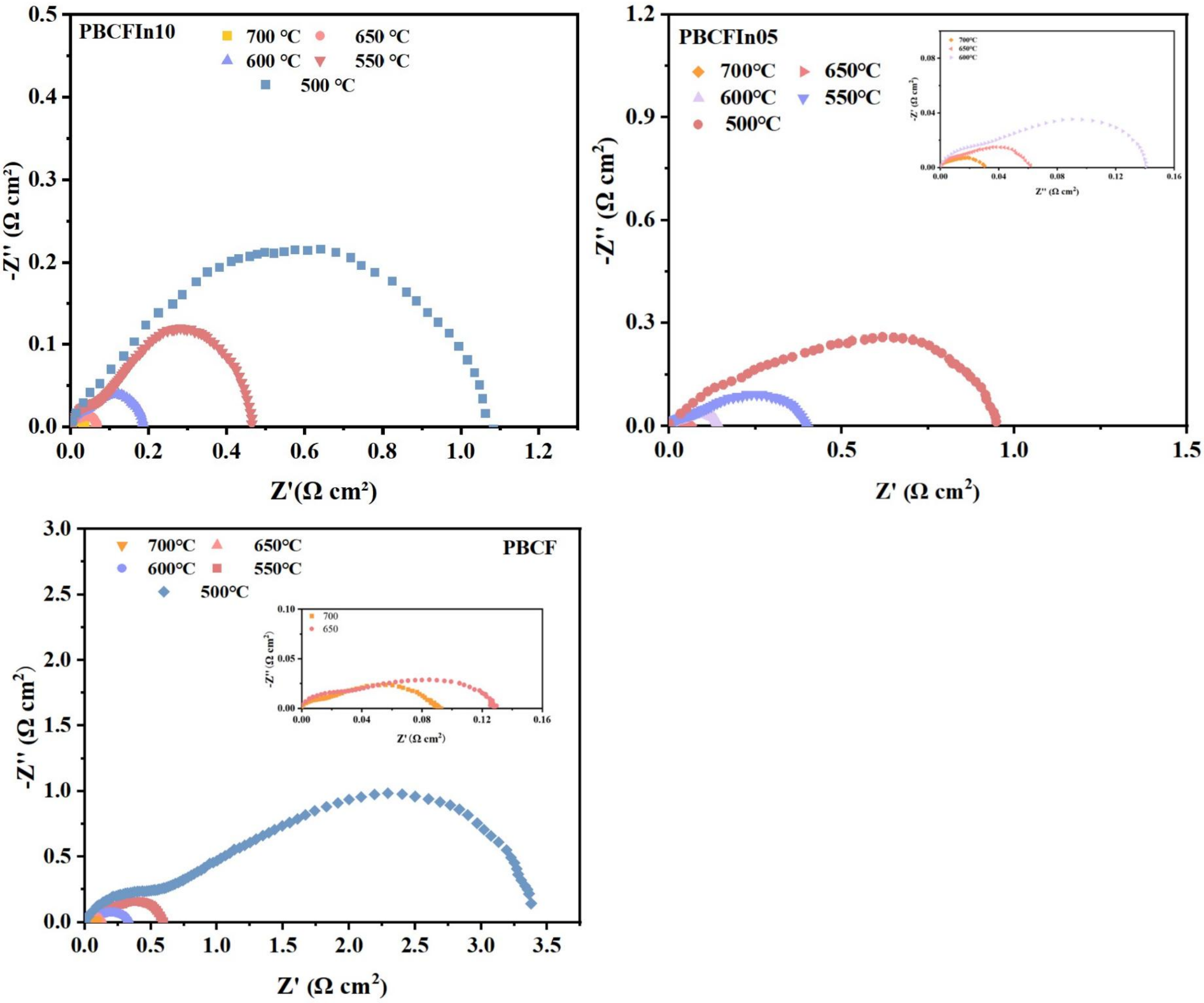

Supplementary Fig. 19. EIS plots of BZCYYb-supported single cells with PBCF, PBCFIn05 and PBCFIn10 at 500–700 °C under 3% H_2O–H_2. The ASRs of the single cell with PBCF air electrode are 3.3, 0.6, 0.32, 0.13, and 0.09 Ω·cm^2 at 500–700°C，respectively. Under the same test conditions, the ASRs of the single cell with PBCFIn10 air electrode are 1.1, 0.46, 0.19, 0.07, and 0.04 Ω·cm^2 at 500–700°C, respectively. The ASRs of the single cell with PBCFIn05 air electrode are 0.94, 0.39, 0.14, 0.06, and 0.03 Ω·cm^2 at 500–700 °C respectively. Source data for Supplementary Fig. 19 are provided as a Source Data file.

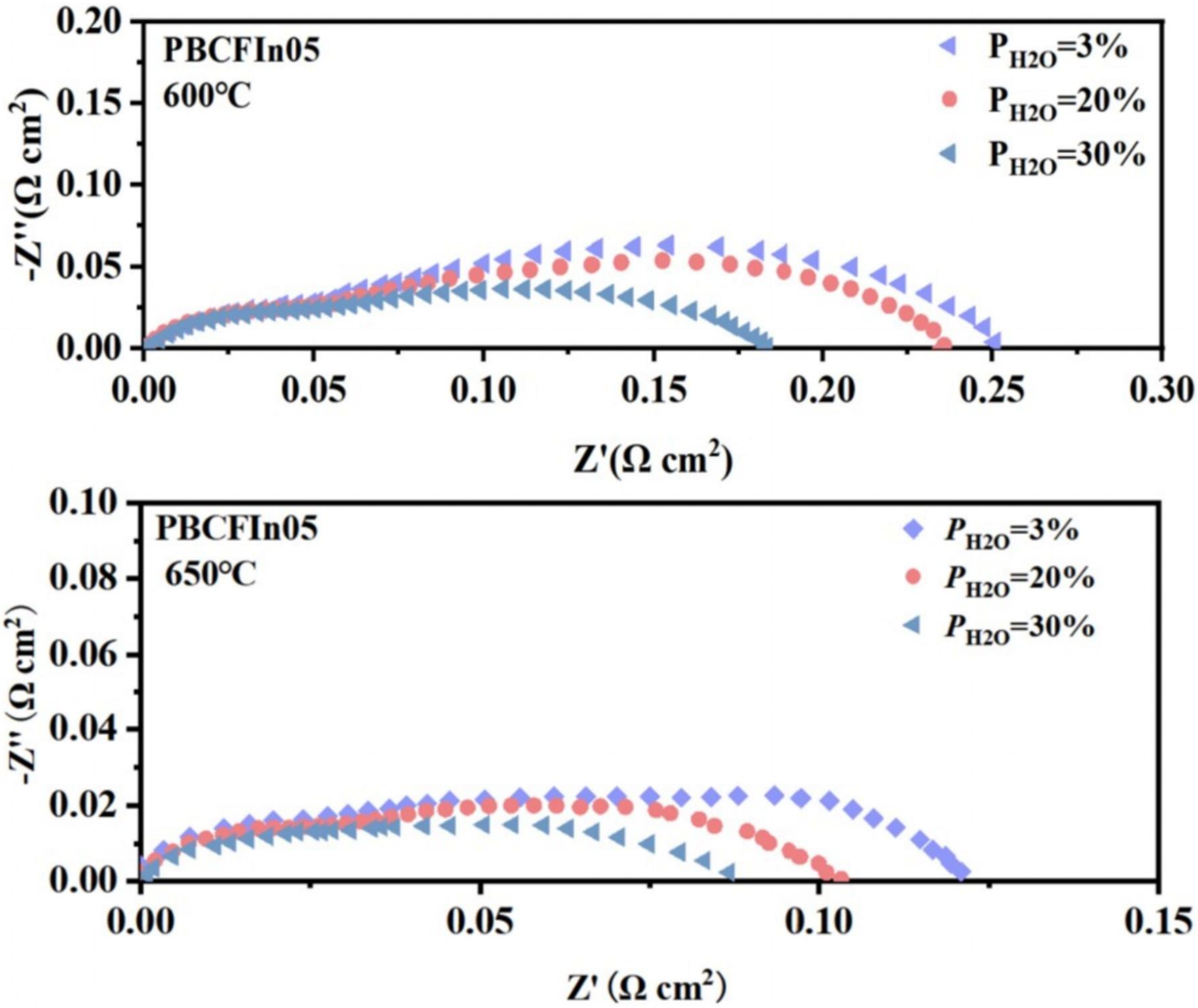

Supplementary Fig. 20. EIS plots of cell with PBCFIn05 air electrode tested at 600–650 ℃ under 3, 10, 30% H_2O-air. The ASRs of the single cell with PBCFIn05 at 600 ℃ are 0.18, 0.23,0.25 Ω·cm² respectively. The ASRs of the single cell with PBCFIn05 at 650 ℃ are 0.08, 0.1, 0.12 Ω·cm², respectively. Source data for Supplementary Fig. 20 are provided as a Source Data file.

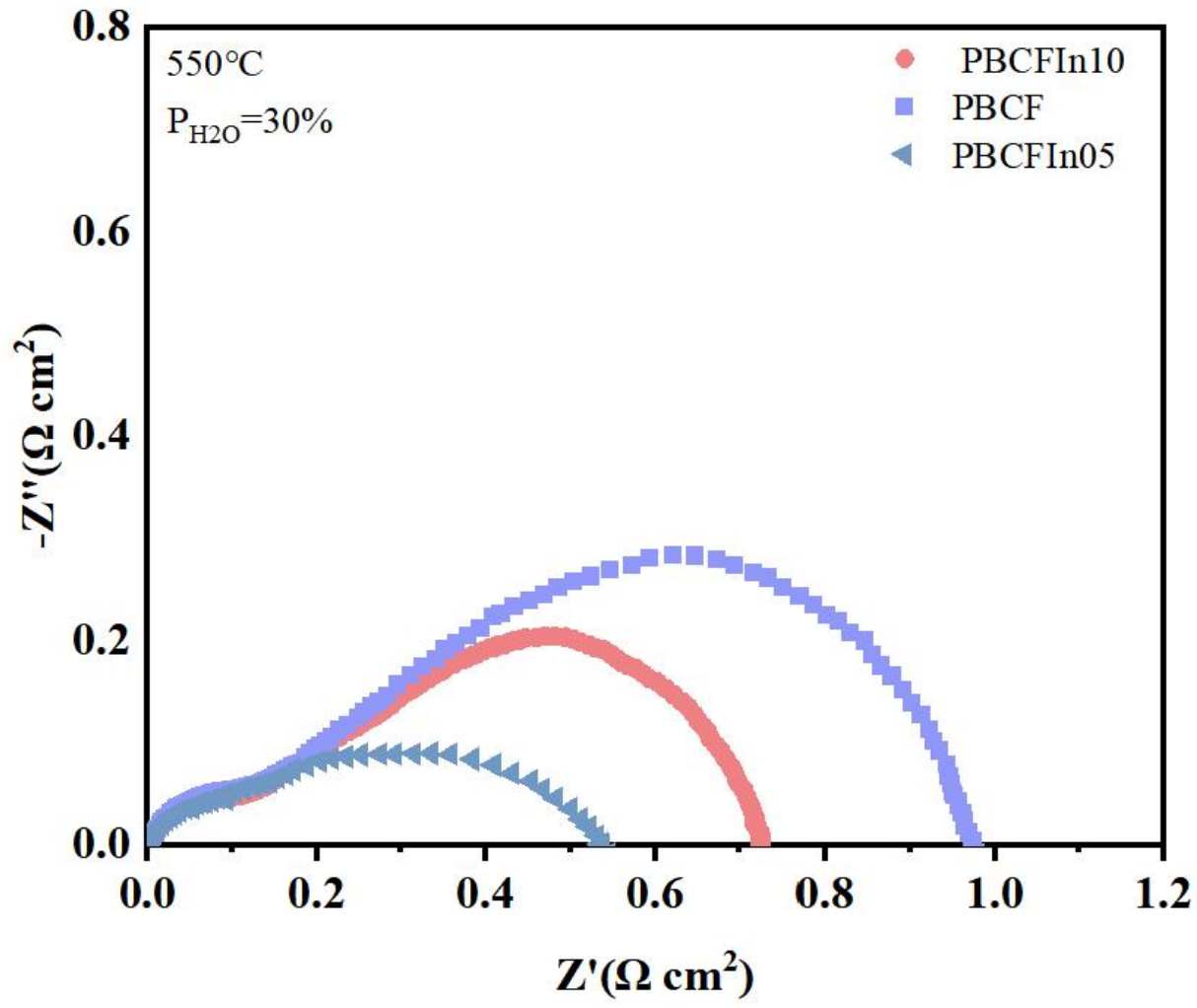

Supplementary Fig. 21. EIS plots of single cell with PBCF PBCFIn05 and PBCFIn10 at 550 ℃ under 30% H_2O. Source data for Supplementary Fig. 21 are provided as a Source Data file.

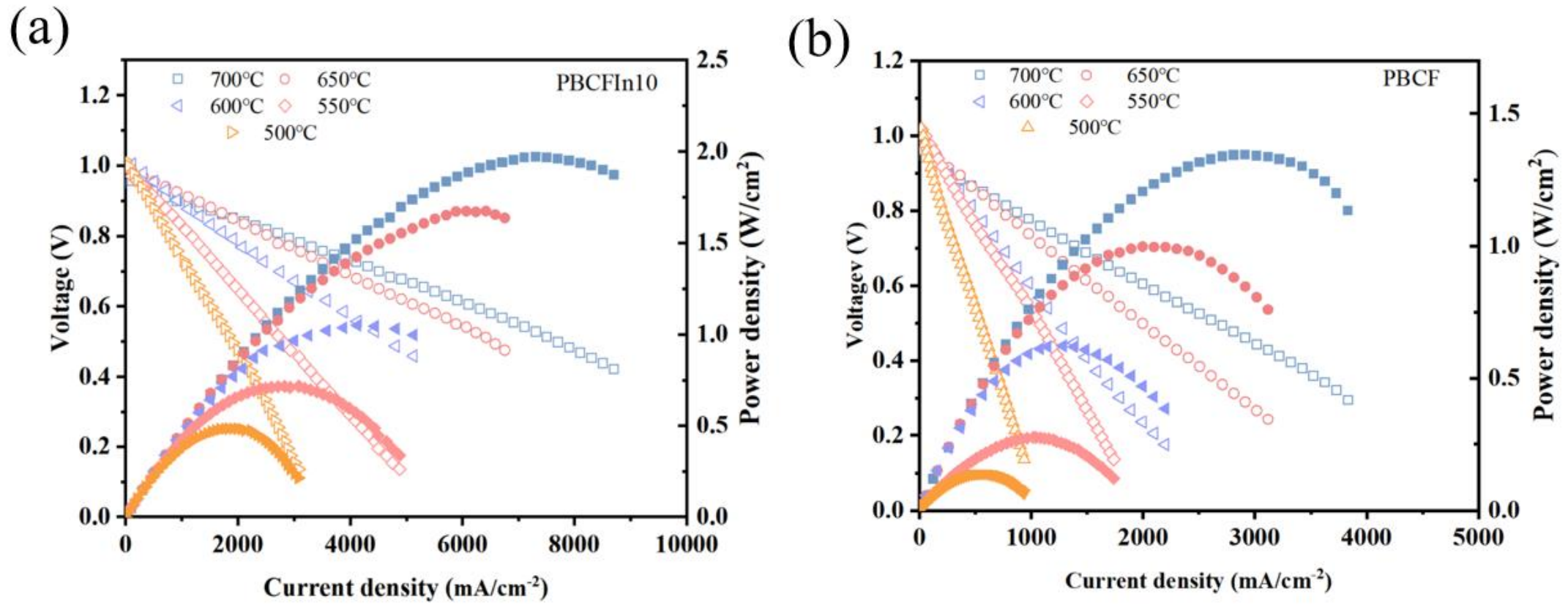

Supplementary Fig. 22. I–V and I–P curves of a BZCYYb-supported single cell with PBCF and PBCFIn10 air electrodes in FC mode at 500–700 °C. (a) The peak power densities (PPDs) of the single cell with the PBCFIn10 air electrode are 0.48, 0.71, 1.20, 1.67, and 1.97 W·cm^{-2} at at 500–700 °C, respectively. (b) The PPDs of the single cell with the PBCF air electrode are 0.14, 0.28, 0.62, 1.01, and 1.34 W·cm^{-2} at at 500–700 °C, respectively. Notably, the PBCFIn10 electrode achieves ~47% higher PPD at 700 °C compared with pristine PBCF, and this enhancement is consistently observed across the measured temperature range, highlighting the beneficial role of In doping in promoting electrode reaction kinetics. By comparison, the PBCFIn05-based cell (Figure 5f) delivers much higher PPDs of 0.79, 1.21, 1.58, 1.95, and 2.47 W·cm^{-2} at 500–700 °C, corresponding to improvements of ~82%, ~76%, ~155%, ~93%, and ~84% over pristine PBCF, respectively. These results indicate that moderate In doping (In05) is more effective than excessive doping (In10), underscoring the optimized defect engineering and superior catalytic activity of PBCFIn05. Source data for Supplementary Fig. 22 are provided as a Source Data file.

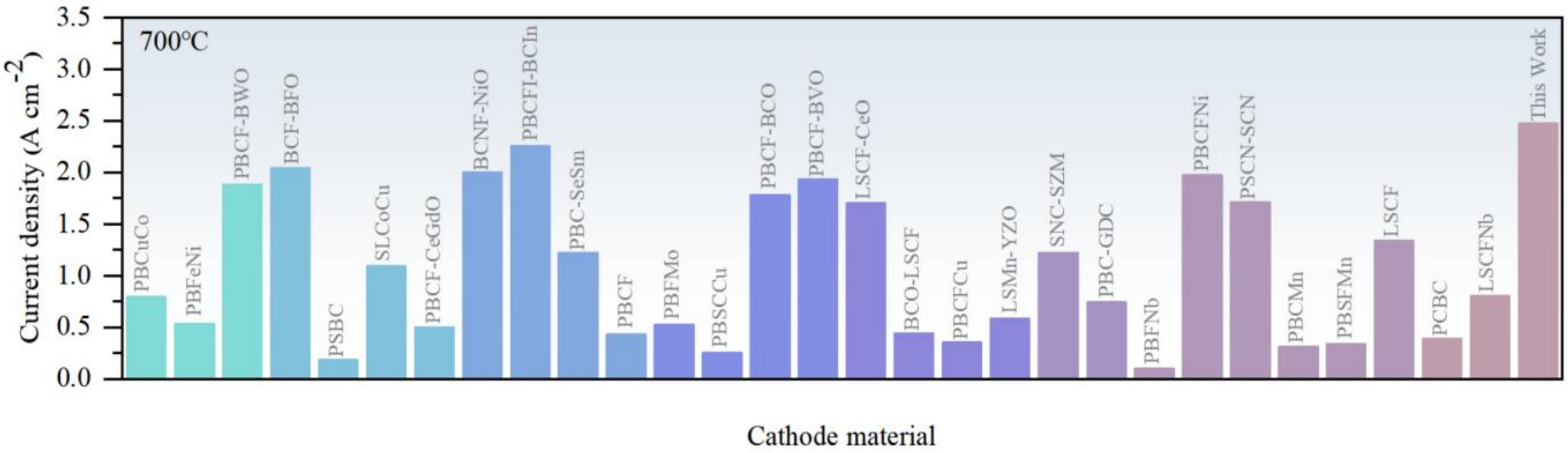

Supplementary Fig. 23. Comparison of PDDs obtained with PBCFIn05 electrode and advanced electrodes in the literature at different temperatures. Source data for Supplementary Fig. 23 are provided as a Source Data file.

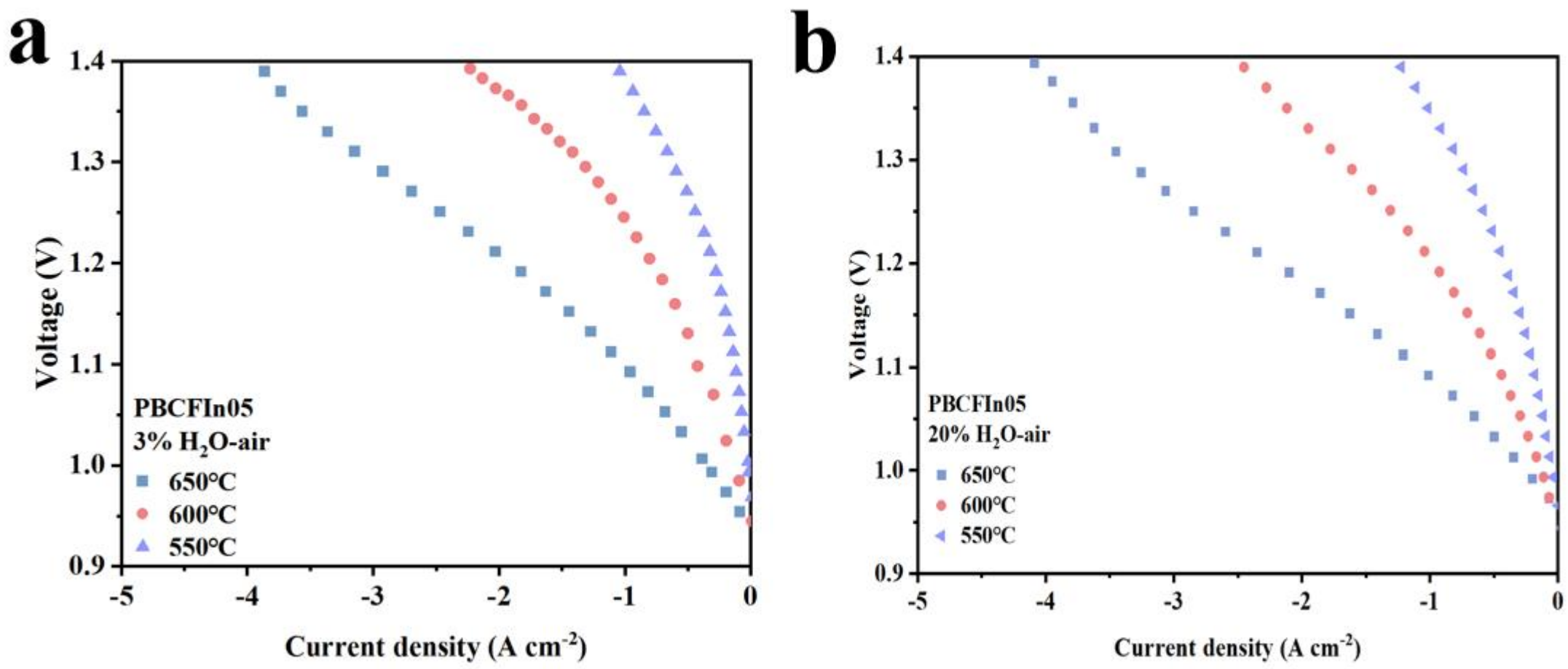

Supplementary Fig. 24. I-V curves of cells with PBCFIn05 electrode measured in electrolysis cell (EC) mode at 650−550 °C under 3 and 20% H_2O-air. Source data for Supplementary Fig. 24 are provided as a Source Data file.

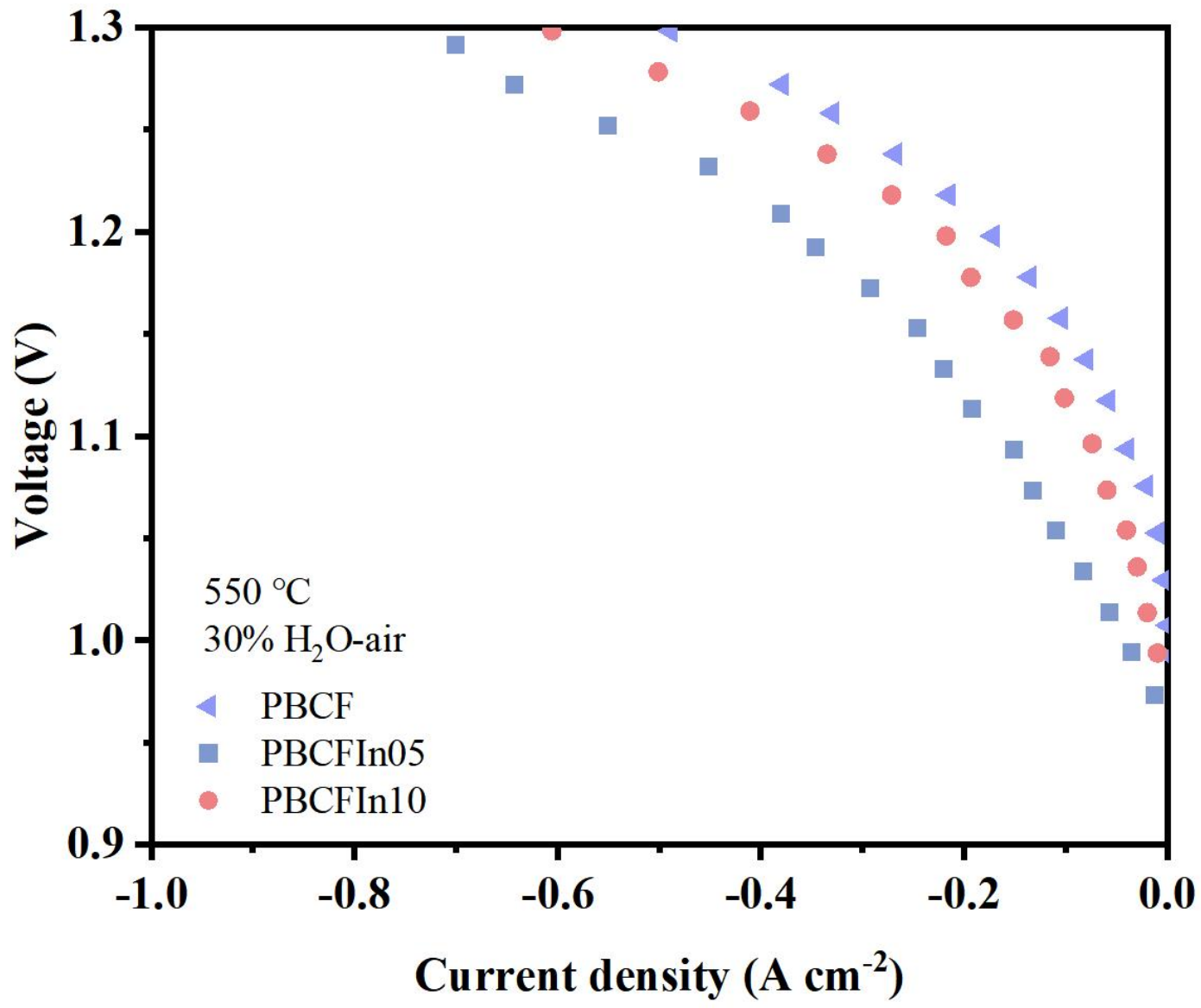

Supplementary Fig. 25. Electrochemical performance of BZCYYb-supported single cells with PBCF, PBCFIn05, and PBCFIn10 air electrodes in electrolysis cell (EC) mode at 550 °C with 30% H_2O–air. The current densities at 1.3 V are 0.71, 0.60, and 0.48, A·cm^{-2} for PBCFIn05, PBCFIn10, and PBCF cells, respectively, confirming the superior electrolysis activity of the moderately doped PBCFIn05 electrode. Source data for Supplementary Fig. 25 are provided as a Source Data file.

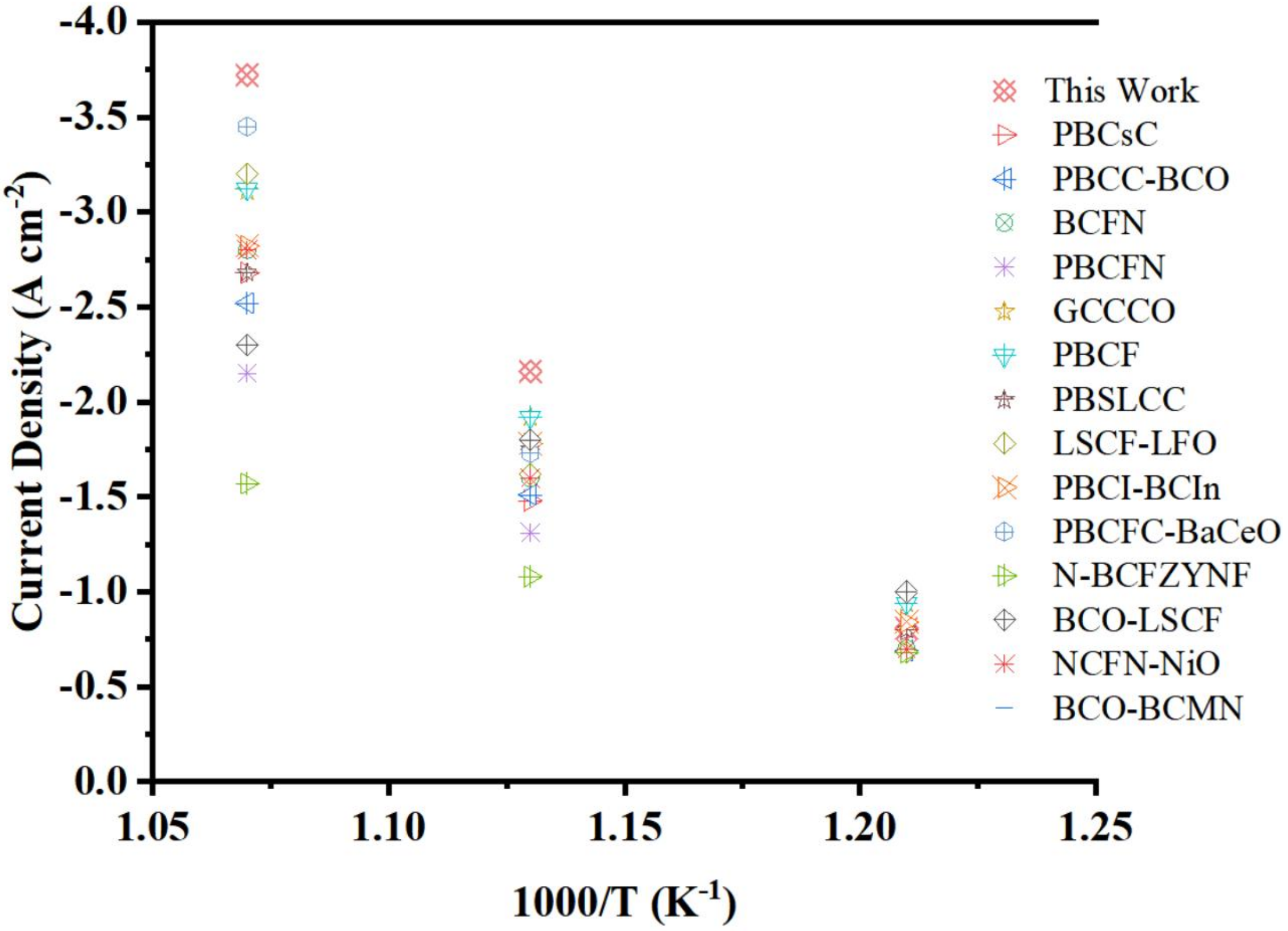

Supplementary Fig. 26. Comparison of PDDs obtained with PBCFIn05 electrode and advanced electrodes in the literature at 1.3 V and 600 °C. Source data for Supplementary Fig. 26 are provided as a Source Data file.

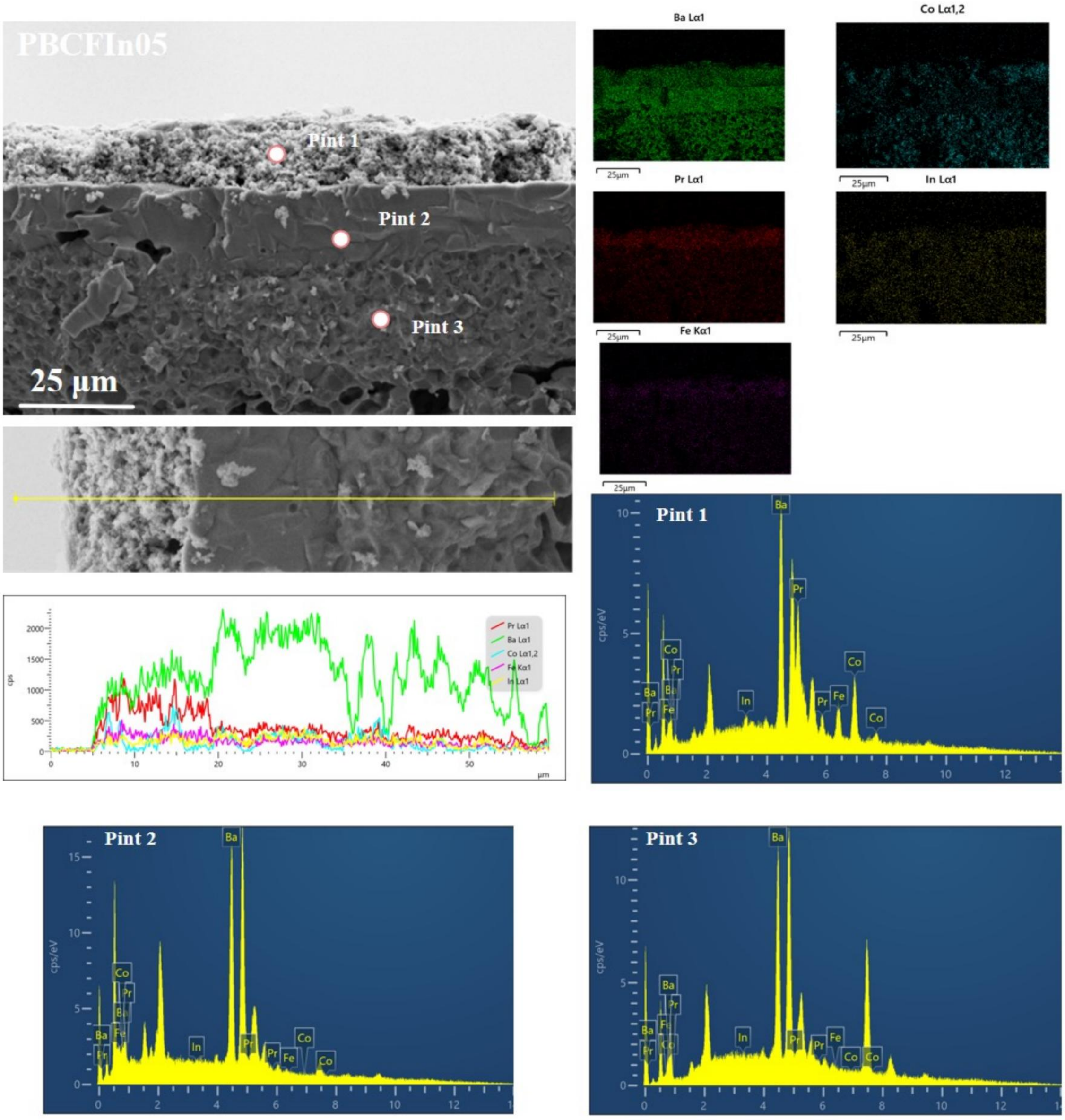

Supplementary Fig. 27. SEM image of PBCFIn05 single cell structure after tested. Source data for Supplementary Fig. 27 are provided as a Source Data file.

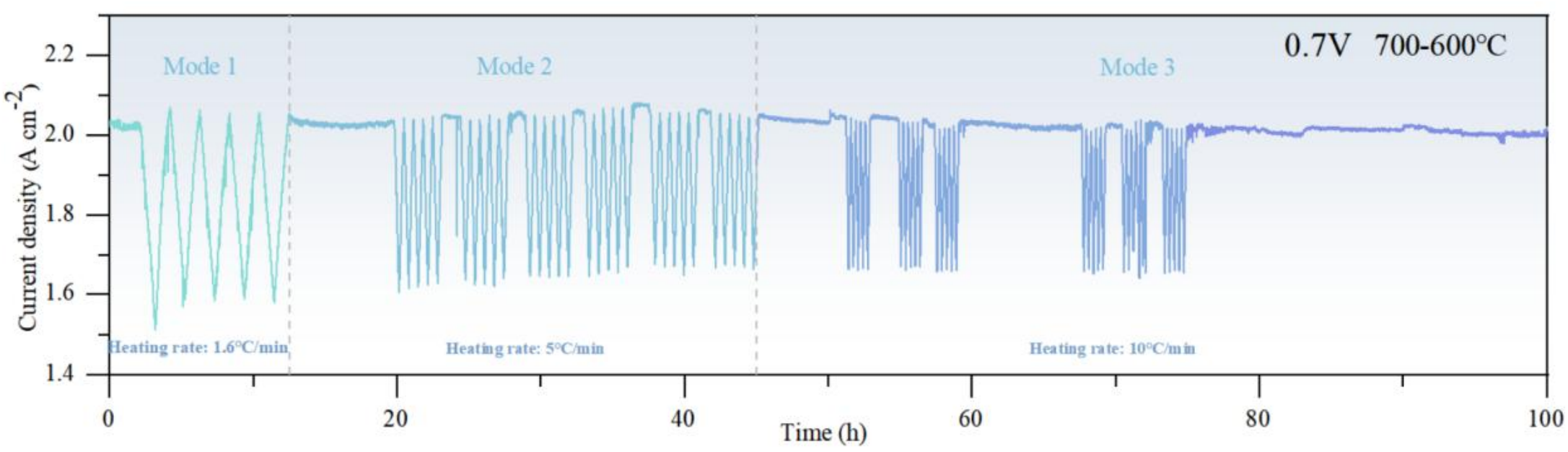

Supplementary Fig. 28. A stability test of PBCFIn05 in 0.7 V at 700–600 °C. Source data for Supplementary Fig. 28 are provided as a Source Data file.

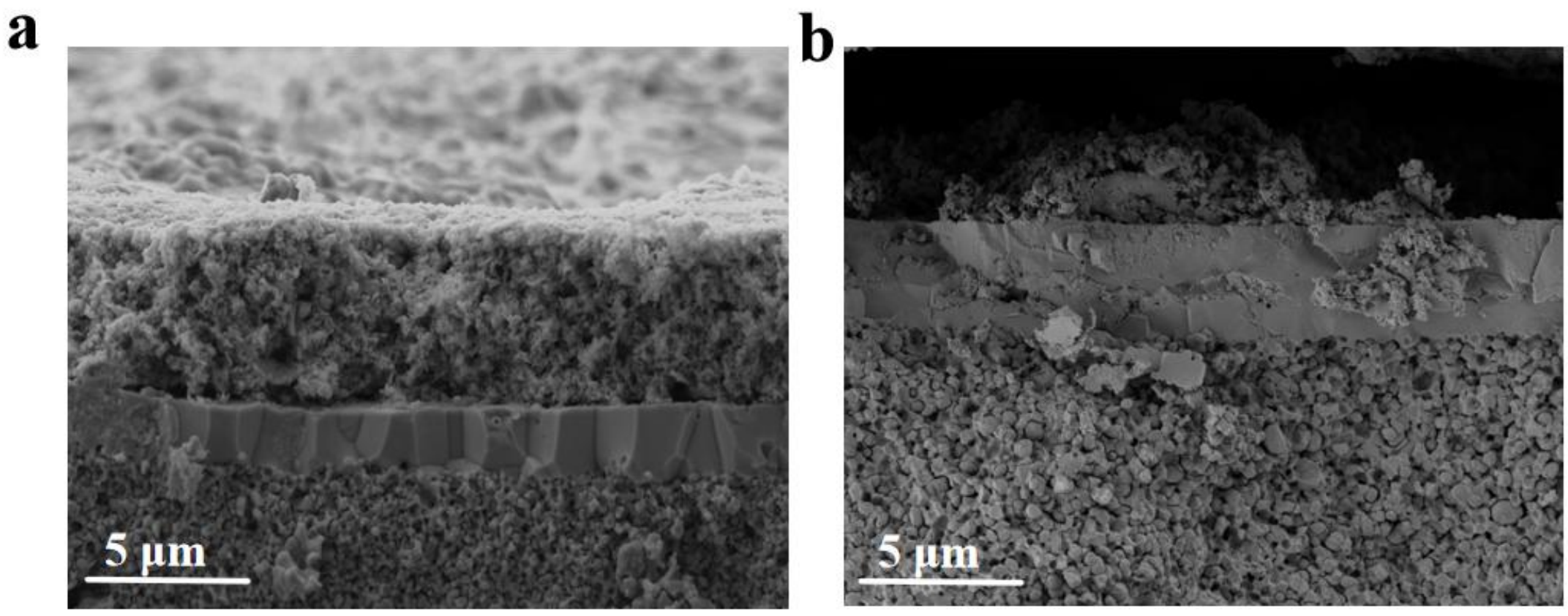

Supplementary Fig. 29. SEM image of PBCFIn05 single cell structure before and after the electrochemical tests at temperature cycling mode.

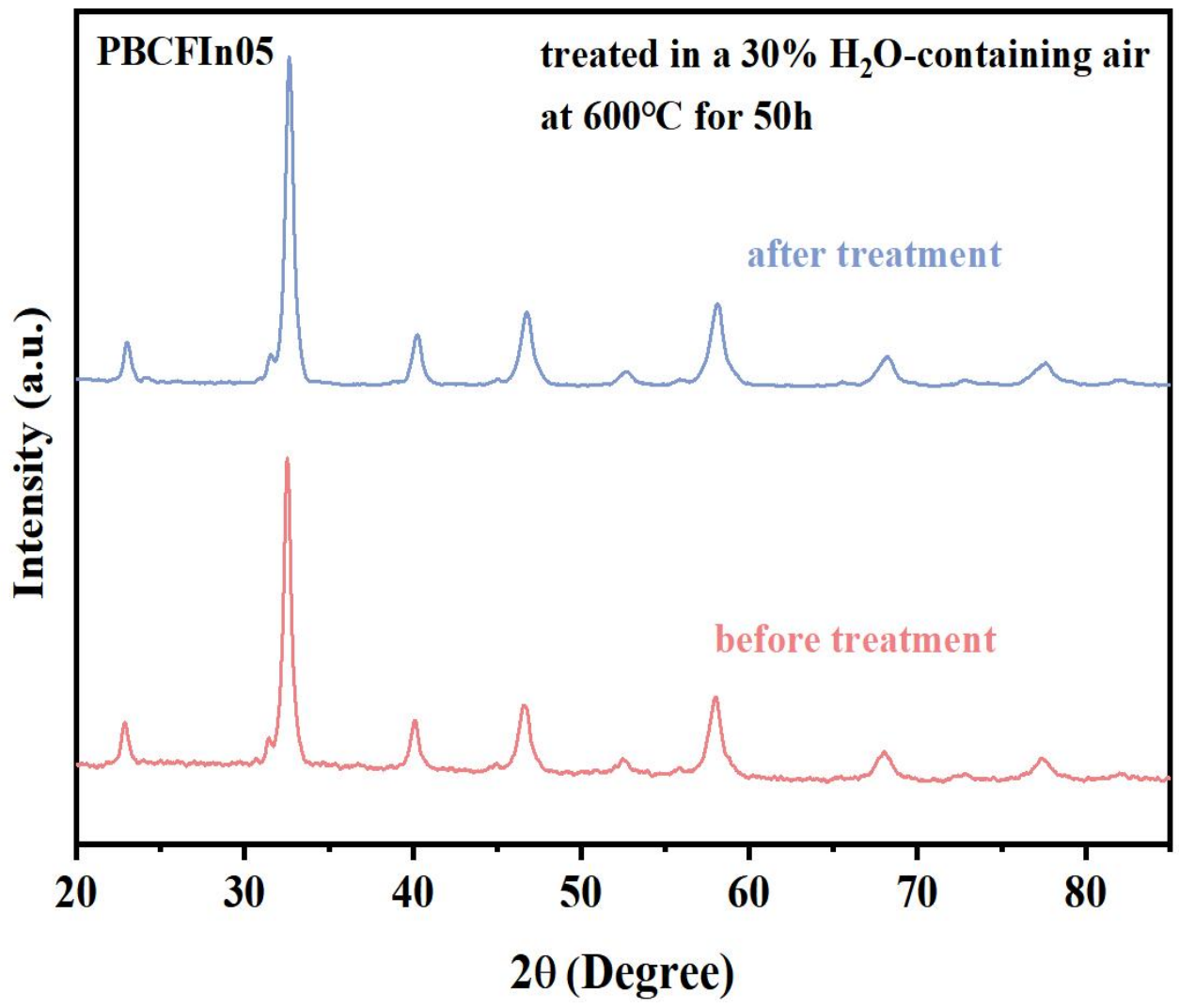

Supplementary Fig. 30. Typical XRD patterns of the PBCFIn05 under 30% H_2O-containing air at 600 °C for 50 h. Source data for Supplementary Fig. 30 are provided as a Source Data file.

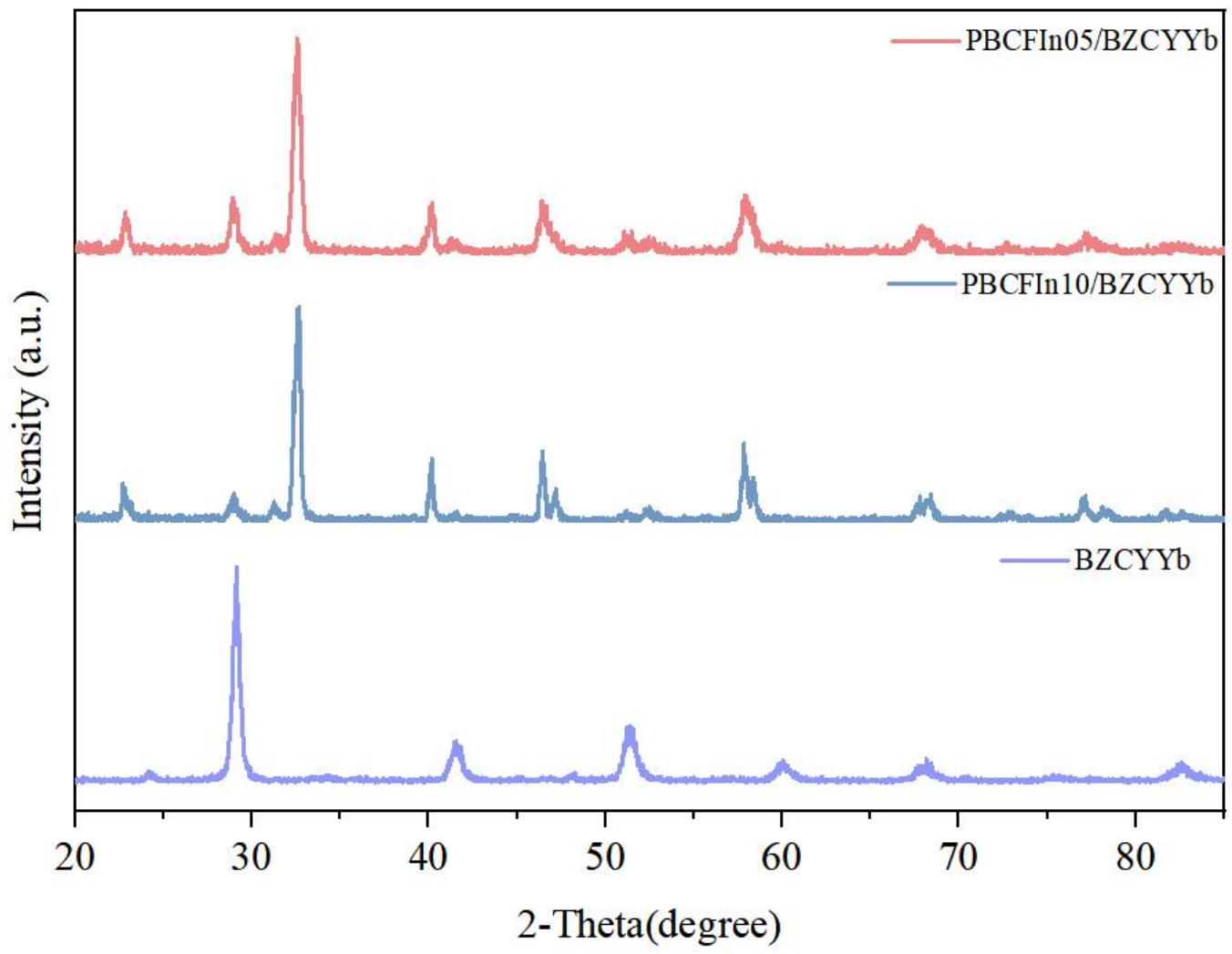

Supplementary Fig. 31. XRD patterns of the PBCFIn05/BZCYYb, PBCFIn10/BZCYYb and BZCYYb. Source data for Supplementary Fig. 31 are provided as a Source Data file.

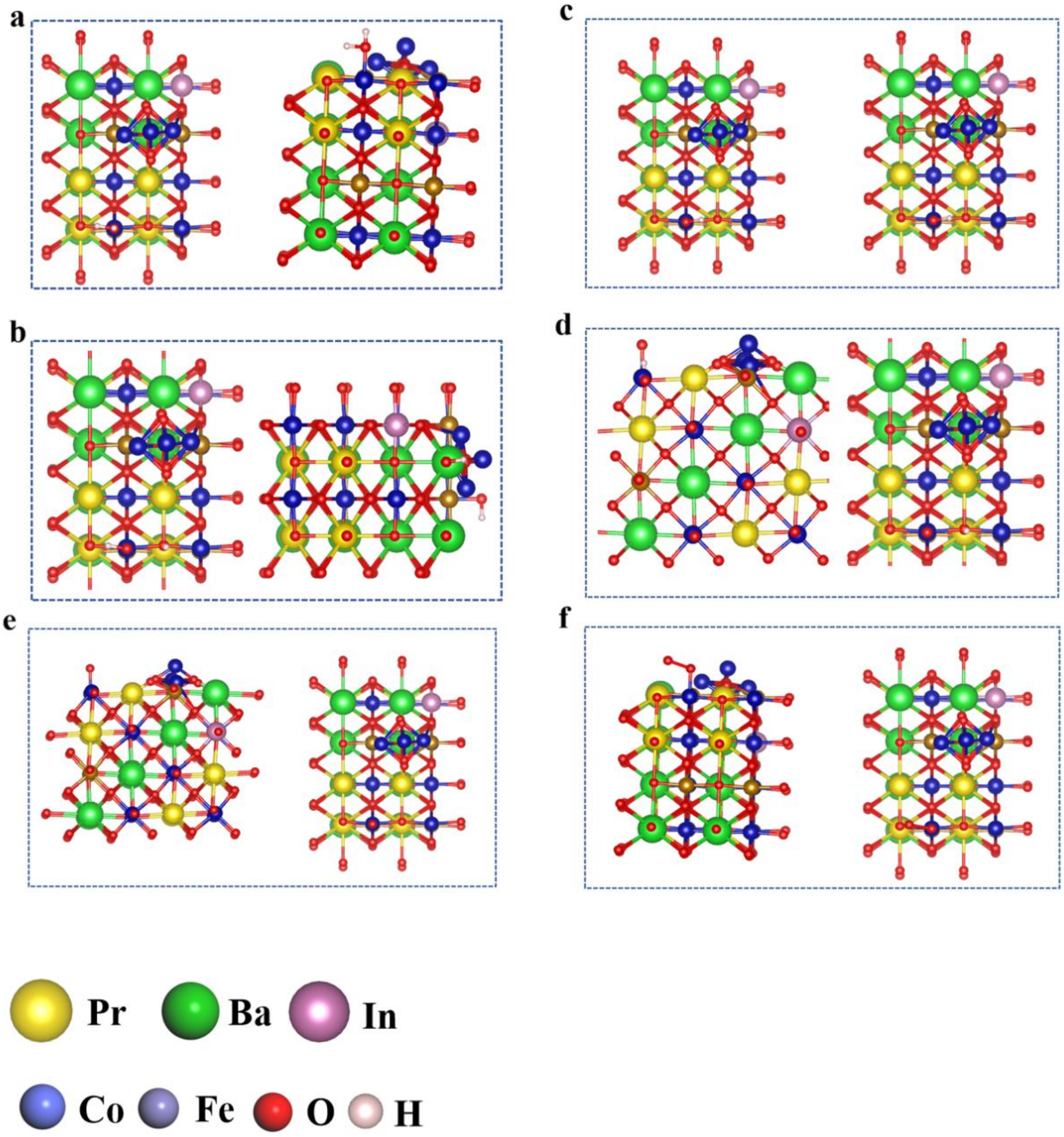

Supplementary Fig. 32. Optimized surface terminations and adsorption geometries of key OER/ORR intermediates on PBCFIn05.(a)*H_2O, (b)*OH+*H, (c) *OH (d) *O+*H (e) *O, (f) *OO. Source data for Supplementary Fig. 32 are provided as a Supplementary data 1 file.

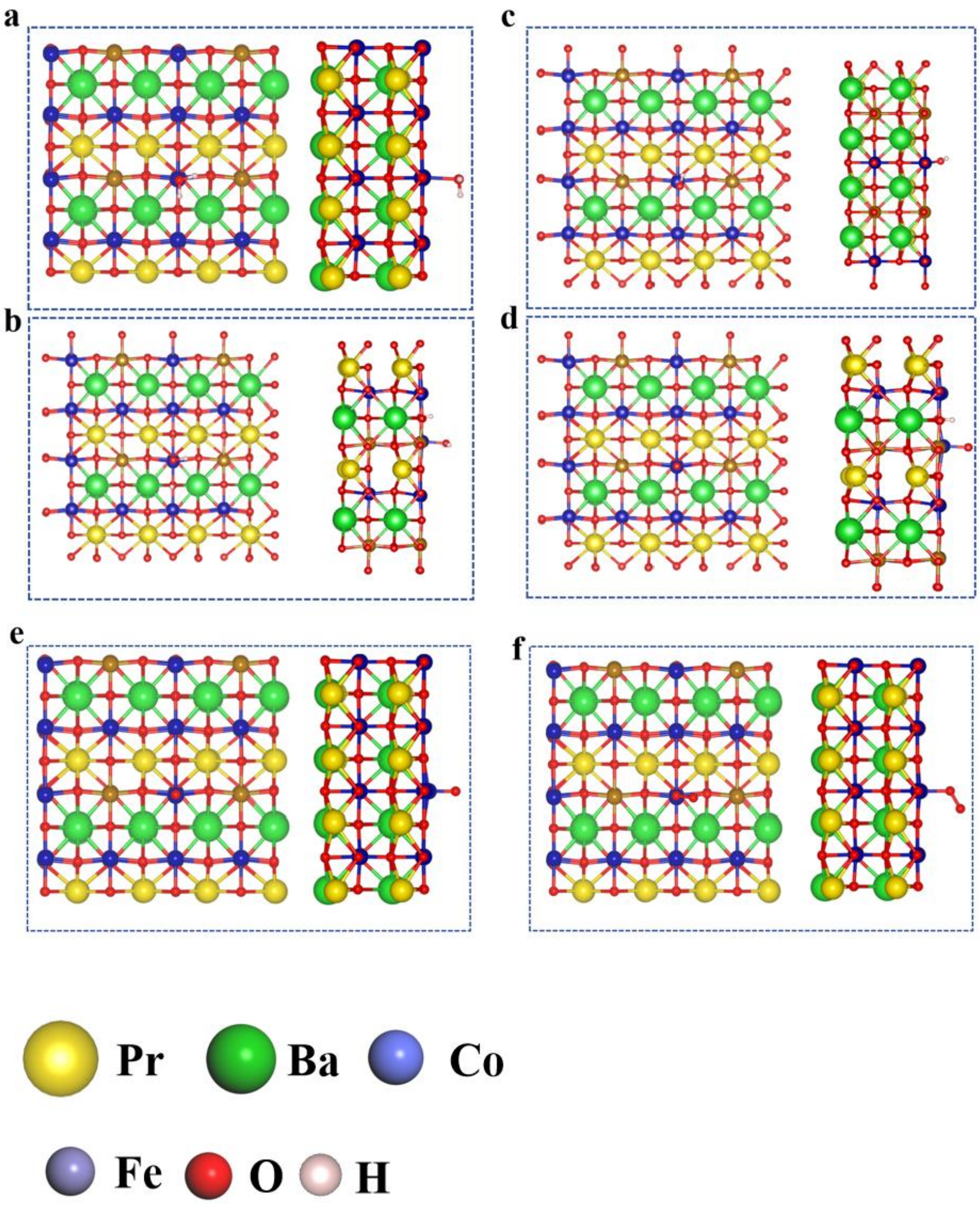

Supplementary Fig. 33. Optimized surface terminations and adsorption geometries of key OER/ORR intermediates on PBCF. (a)$*H_2O$, (b)*OH+*H, (c) *OH (d) *O+*H (e) *O, (f) *OO. Source data for Supplementary Fig. 33 are provided as a Supplementary data 1 file.

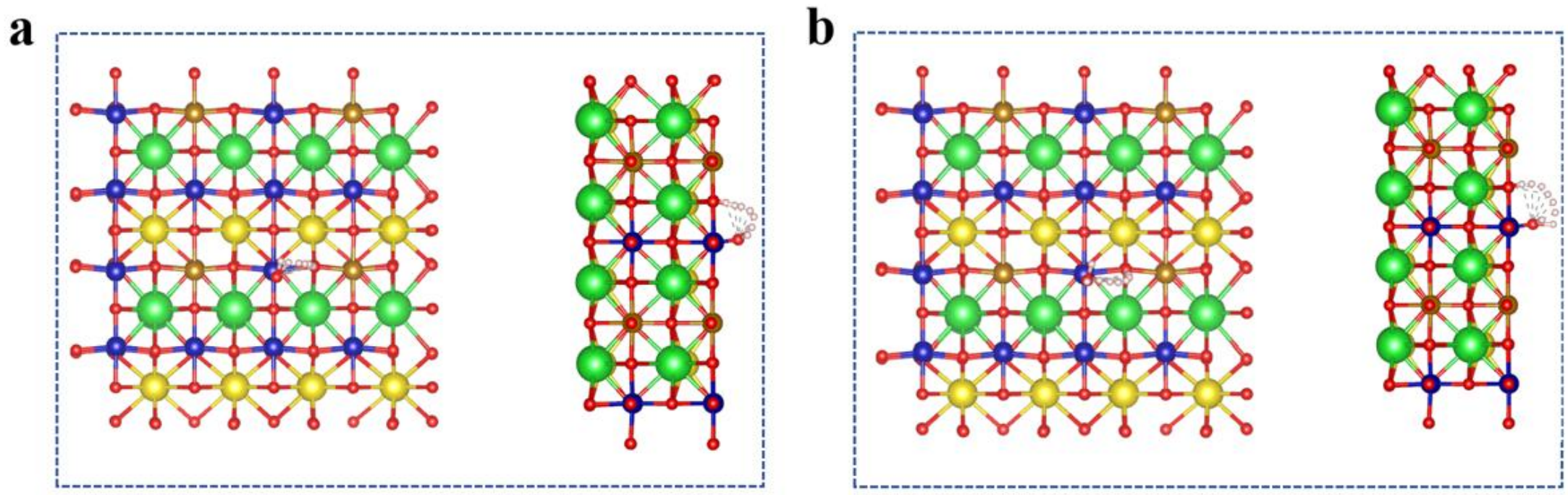

Supplementary Fig. 34. CI-NEB calculations of the **a)**$*H_2O \rightarrow *OH + H$ and **b)** $*OH \rightarrow *O + H$ dissociation pathway for PBCF. Source data for Supplementary Fig. 34 are provided as a Supplementary data 1 file.

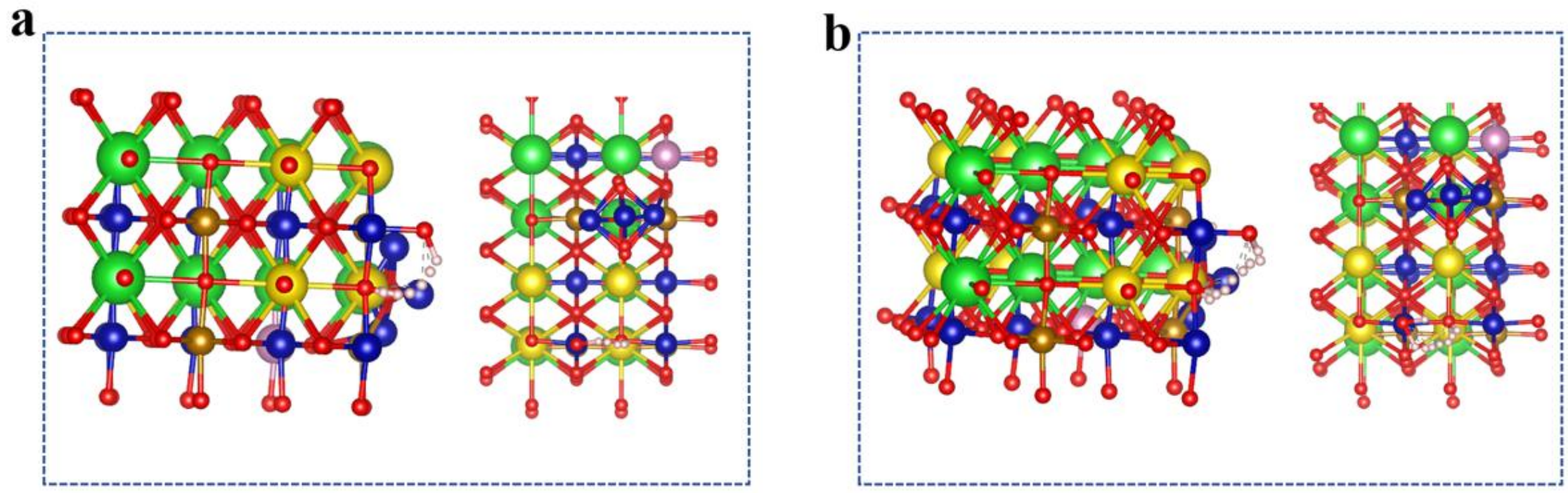

Supplementary Fig. 35. CI-NEB calculations of the **a)**$*H_2O \rightarrow *OH + H$ and **b)** $*OH \rightarrow *O + H$ dissociation pathway for PBCFIn05. Source data for Supplementary Fig. 35 are provided as a Supplementary data 1 file.

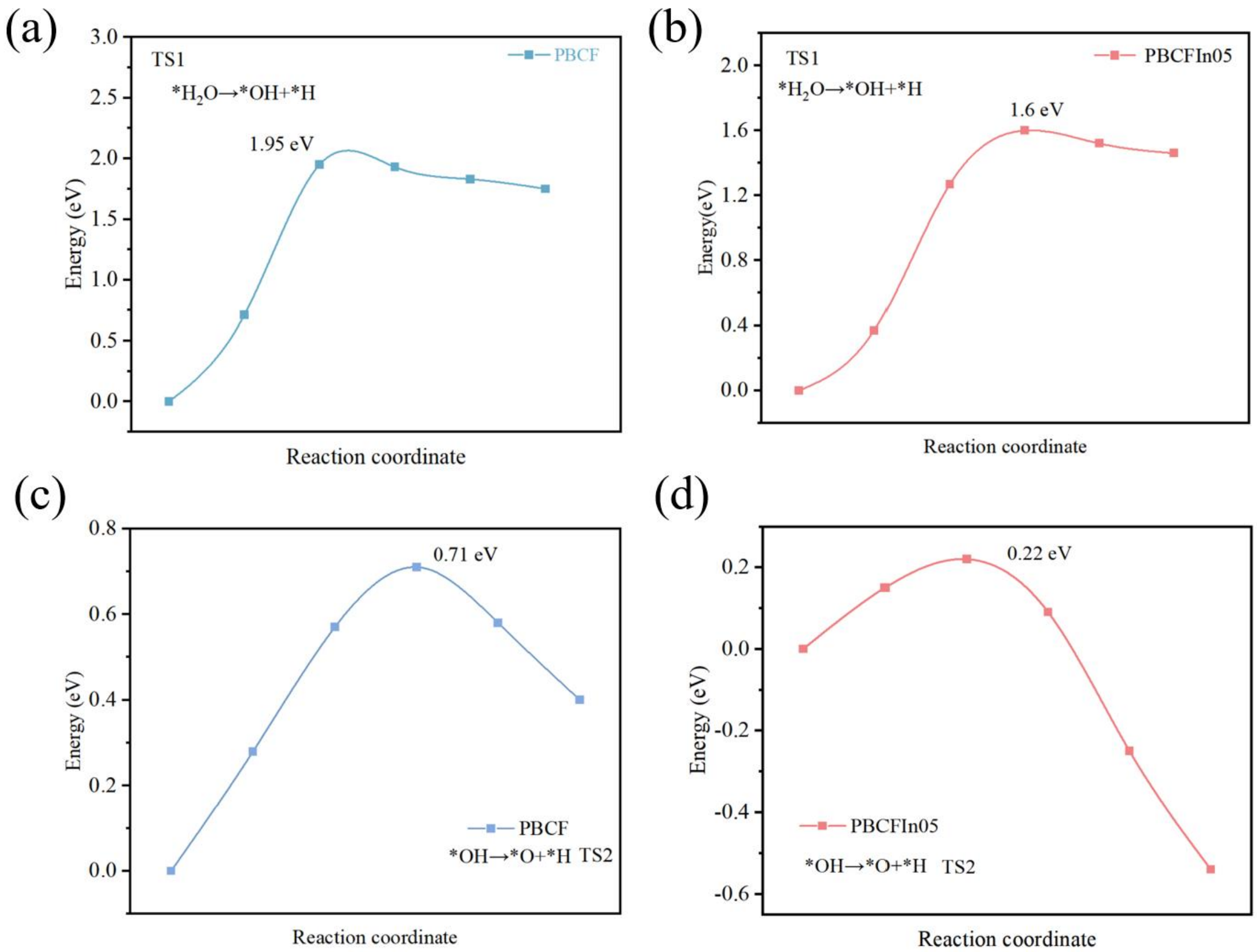

Supplementary Fig. 36. Calculated relative energy profiles for OER stepson PBCF and PBCFIn05. （a,b） CI-NEB energy profiles for *H_2O→*OH+*H; (c,d) CI-NEB energy profiles for *OH→*O+*H. Source data for Supplementary Fig. 36 are provided as a Source Data file.

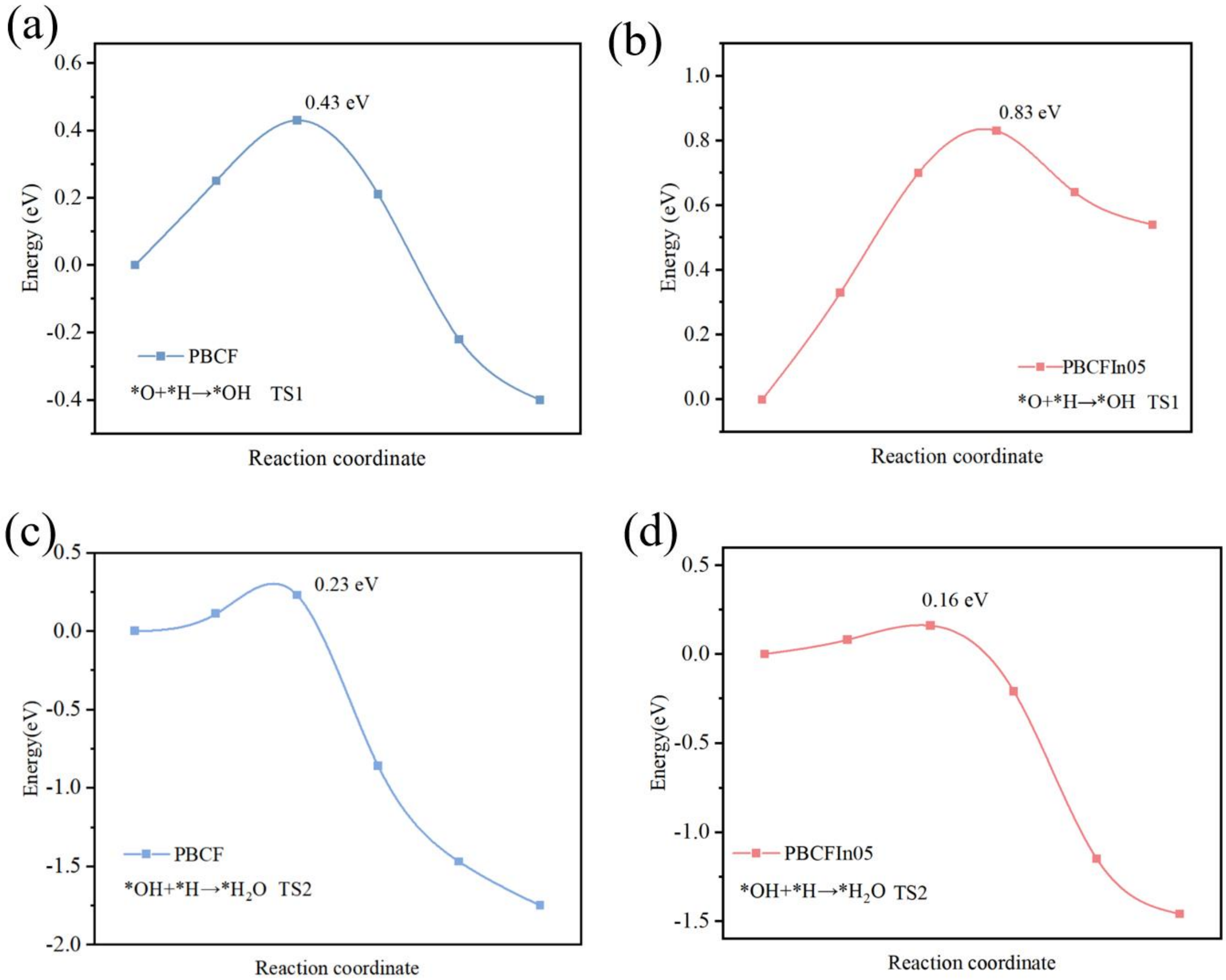

Supplementary Fig. 37. Calculated relative energy profiles for ORR stepson PBCF and PBCFIn05. （a,b）CI-NEB energy profiles for *O+*H→*OH ; (c,d) CI-NEB energy profiles for *OH+*H→H_2O. Source data for Supplementary Fig. 37 are provided as a Source Data file.

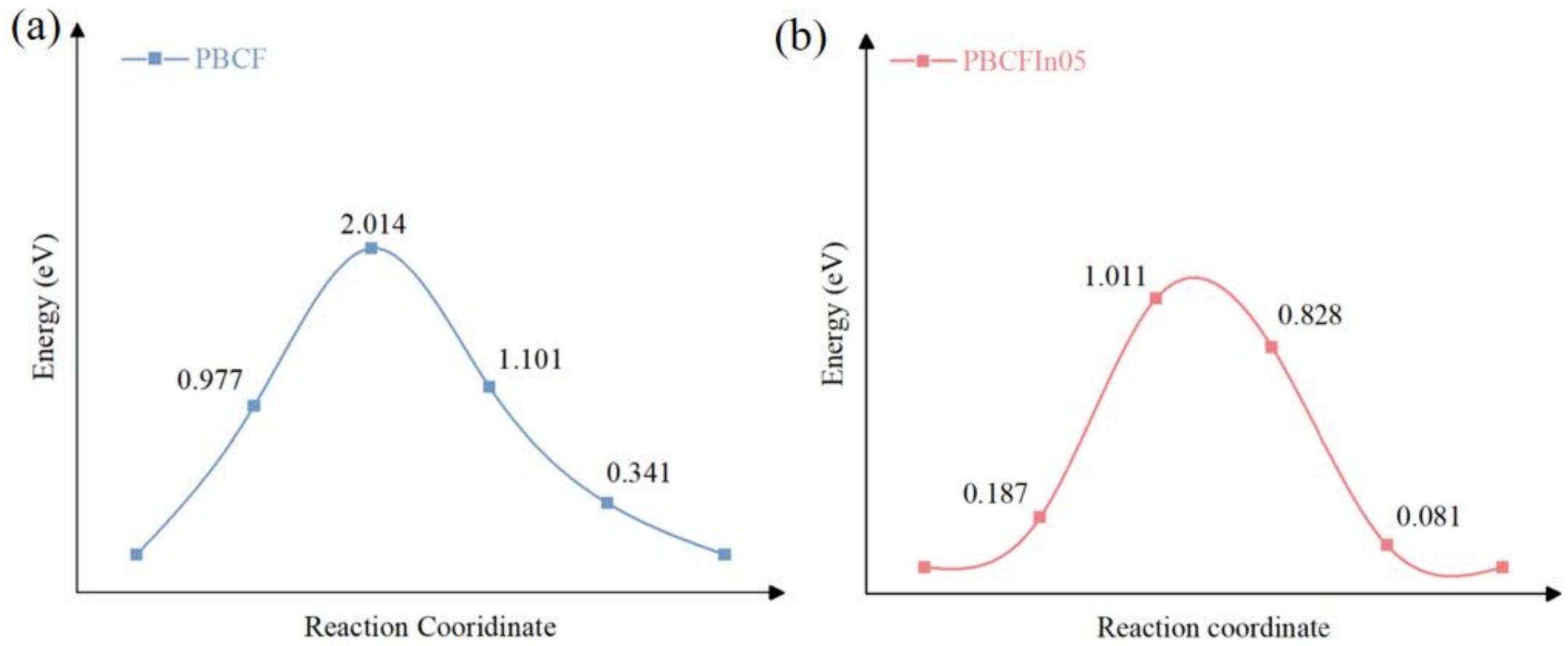

Supplementary Fig. 38. Calculated relative energy profiles for proton migration of **a)** PBCF and **b)** PBCFIn05. Source data for Supplementary Fig. 38 are provided as a Source Data file.

Supplementary Table 1: Rietveld refinement results of PBCF, PBCFIn05, and PBCFIn10 obtained from the first independently synthesized batch.

**Sample**	**PBCF**	**PBCFIn05**	**PBCFIn10**
Percentage and Space group	100% Pm-3m	97.7% Pm-3m<br>2.3% Fd-3m	97.13% P4/mm<br>2.87% Fd-3m
a	3.88360	3.90107	3.92592
b	3.88360	3.90107	3.92592
c	3.88360	3.90107	7.72156
α	90°	90°	90°
γ	90°	90°	90°
β	90°	90°	90°
R_{wp}	10.05	4.56	9.52
R_p	7.73	3.61	13.7
R_{exp}	5.66	4.23	11.35

Supplementary Table 2: Rietveld refinement results of PBCFIn05, and PBCFIn10 obtained from the second independently synthesized batch.

**Sample**	**PBCFIn05**	**PBCFIn10**
Percentage and Space group	97.20% Pm-3m<br>2.80% Fd-3m	97.14% P4/mm<br>2.86% Fd-3m
a	3.88709	3.90500
b	3.88709	3.90500
c	3.88709	7.69565
α	90°	90°
γ	90°	90°
β	90°	90°
R_{wp}	8.10	8.50
R_p	6.32	6.76
R_{exp}	3.00	3.25

Supplementary Table 3: Rietveld refinement results of PBCFIn05, and PBCFIn10 obtained from the third independently synthesized batch.

Sample	PBCFIn05	PBCFIn10
Percentage and Space group	97.15% Pm-3m<br>2.85% Fd-3m	97.17% P4/mm<br>2.83% Fd-3m
a	3.88651	3.90449
b	3.88651	3.90449
c	3.88651	7.69491
α	90°	90°
γ	90°	90°
β	90°	90°
R_{wp}	8.06	8.13
R_p	6.30	6.46
R_{exp}	2.85	2.98

Supplementary Table 4: EXAFS fitting parameters at the Fe K-edge for various samples (S_0^2=0.73 from Fe-foil)

$^a CN$: coordination numbers; $^b R$: bond distance; $^c \sigma^2$: Debye-Waller factors; ^{d} ΔE_0: the inner

	shell	CN^a	R^b(Å)	σ^{2c}(Å^2)	$\Delta E_0{}^d$(eV)	R factor
Fe-foil	Fe-Fe1	8	2.47±0.01	0.0046	6.5±2.3	0.0084
	Fe-Fe2	6	2.85±0.01	0.0057		
PBCFIn05-Fe	Fe-O	5.7±0.4	1.97±0.01	0.0091	-2.8±1.5	0.0184
	Fe-Pr/Ba	8.1±0.8	3.35±0.01	0.0098		
	Fe-Co	5.5±0.9	4.02±0.02	0.0076		
PBCF-Fe	Fe-O	5.7±0.4	2.00±0.01	0.0082	-2.5±1.3	0.0148
	Fe-Pr/Ba	7.8±0.8	3.36±0.02	0.0086		
	Fe-Co	4.6±0.9	4.01±0.02	0.0067		

potential correction. R factor: goodness of fit. A reasonable range of EXAFS fitting parameters: $0.70 < S_0^2 < 1.00$; CN > 0; $\sigma^2 > 0$ Å^2; $|\Delta E_0| < 15$ eV; R factor < 0.02.

The obtained XAFS data was processed in Athena (version 0.9.26) [J. Synchrotron Rad. 2005, 12, 537] for background, pre-edge line and post-edge line calibrations. Then Fourier transformed fitting was carried out in Artemis (version 0.9.26) [J. Synchrotron Rad. 2005, 12, 537]. The k^3 weighting, k-range of 3 - 12.5 Å^{-1} and R range of 1 - 3 Å were used for the fitting of Fe-foil; the k^3 weighting, k-range of 3 - 10 Å^{-1} and R range of 1 -4 Å were used for the fitting of Fe-Sample.

Supplementary Table 5: EXAFS fitting parameters at the Co K-edge for various samples (S_0^2=0.74 from Co-foil).

	Shell	CN^a	R^b(Å)	σ^{2c}(Å^2)	$\Delta E_0{}^d$(eV)	R factor
Co-foil	Co-Co	12	2.49±0.01	0.0062	7.4±0.3	0.0015
PBCFIn05-Co	Co-O	5.3±0.3	1.89±0.01	0.0040	-4.0±0.9	0.0118
	Co-Ba/Pr	4.9±0.6	3.33±0.02	0.0080		

^{a}*CN*: coordination numbers; ^{b}*R*: bond distance; $^c\sigma^2$: Debye-Waller factors; ^{d} ΔE_0: the
^{a}*CN*: coordination numbers; ^{b}*R*: bond distance; $^c\sigma^2$: Debye-Waller factors; ^{d} ΔE_0: the inner potential correction. R factor: goodness of fit. A reasonable range of EXAFS fitting parameters: $0.70 < S_0^2 < 1.00$; CN > 0; $\sigma^2 > 0$ Å^2; $|\Delta E_0| < 15$ eV; R factor < 0.02.

The obtained XAFS data was processed in Athena (version 0.9.26) [J. Synchrotron Rad. 2005, 12, 537] for background, pre-edge line and post-edge line calibrations. Then Fourier transformed fitting was carried out in Artemis (version 0.9.26) [J. Synchrotron Rad. 2005, 12, 537]. The k^3 weighting, k-range of 3- 12.5 Å^{-1} and R range of 1 - 3 Å were used for the fitting of Co-foil; The k^3 weighting, k-range of 3 - 10 Å^{-1} and R range of 1 - 4 Å were used for the fitting of Co-Sample.

Supplementary Table 6: O 1s species of obtained from analysis for PBCF, PBCFIn05 and PBCFIn10.

**Sample**	**Lattice oxygen (Olat)**	**O^-/O^{2-}**	**Adsorbed oxygen (Oads)**	**OH**	**（O_{ads}/O_{lat}）**
PBCF	23.1%	20.4%	41.6%	14.9%	1.8
PBCFIn05	15.2%	10.0%	54.1%	20.7%	3.56
PBCFIn10	17.8%	17.1%	45.6%	19.5%	2.56

Supplementary Table 7: Fe 2p species of obtained from analysis for PBCF, PBCFIn05 and PBCFIn10.

**Sample**	**Fe^{3+}**	**Fe^{4+}**	**Fe^{3+}/Fe^{4+}**
PBCF	69.6%	30.4%	2.29
PBCFIn05	65.5%	34.5%	1.89
PBCFIn10	60.1%	39.9%	1.51

Supplementary Table 8: Co 3d species of obtained from analysis for PBCF, PBCFIn05 and PBCFIn10.

**Sample**	**Co^{3+}**	**Co^{4+}**	**Co^{3+}/Co^{4+}**
PBCF	45.6%	54.34%	0.84
PBCFIn05	39.6%	60.32%	0.66
PBCFIn10	37.04%	62.96%	0.588

Supplementary Table 9: Quantification of proton uptake by Karl Fischer titration

Sample	Mass (g)	Released H_2O (μg, KF)	Released H_2O (μg g-1)	Released H_2O (wt%)	H_2O uptake*(mmol H_2O /mol oxide)	Proton uptake * (mmol H/ mol oxide)
PBCFIn10	0.5524	20.3	36.75	0.0037	0.5117	1.0235
PBCFIn05	0.4838	18	41.04	0.0041	0.5845	1.1691
PBCF	0.5334	10.3	19.31	0.0019	0.2627	0.5255

Supplementary Table 10: Performance (FC mode) comparison between PBCFIn05 and other air electrode materials

Air electrode	Temp. (°C)	Power density ($W\cdot cm^{-2}$)	Refs.
PBCFIn05	700	2.47	This work
(PB)0.95FNb	700	0.10	[3]
PBSCCu	700	0.25	[4]
PBCMn	700	0.31	[5]
PBCFCu	700	0.35	[6]
PCBC	700	0.39	[7]
PBCF	700	0.43	[8]
PBC-GDC	700	0.74	[9]
PBC-SeSm	700	1.22	[10]
LSCF	700	1.34	[11]
PBCFNi	700	1.97	[12]
PBCuCo	700	0.79	[13]
BCO@LSCF	700	0.44	[14]
LSMn-YZO	700	0.58	[15]
LSCFNb	700	0.80	[16]

PBCF-CeGdO	700	0.50	[17]
PBFeNi	700	0.53	[18]
PBFMo	700	0.52	[19]
PBSFMn	700	0.34	[20]
SLCoCu	700	1.09	[21]
PSBC	700	0.18	[22]
PBCF-BWO	700	1.88	[23]
BCF-BFO	700	2.04	[24]
PCFCs-RuO	700	1.78	[25]
BCNF-NiO	700	2.0	[26]
PBCI-BCI	700	2.25	[27]
PBCFC-BCO	700	1.78	[28]
PBCF-BVO	700	1.93	[29]
LSCF-CeO	700	1.7	[30]
SNC-SZM	700	1.22	[31]
PSCN-SCN	700	1.71	[32]

Supplementary Table 11: Performance (EC mode) comparison between PBCFIn05 and other air electrode materials

Air electrode	Electrolyte	Temp.(ºC)	Current density @ 1.3 V ($A·cm^{-2}$)	Refs
PBCsC	BZCYYb	650	-2.68	33
		600	-1.48	
		550	-0.80	
PBCC-BCO	BZCYYb	650	-2.52	34
		600	-1.51	
		550	-0.69	
BCFN	BZCYYb	650	-2.80	35
		600	-1.60	
		550	-0.70	
PBCFN	BZCYYb	700	-3.85	36
		650	-2.15	
		600	-1.31	
GCCCO	BZCYYb	700	-4.49	37
		650	-3.11	
		600	-1.90	
PBCF	BZCYYb	700	-3.12	28
		650	-1.92	
		600	-0.94	
PBSLCC	BZCYYb	650	-2.68	38
		600	-1.75	
		550	-0.80	
LSCF-LFO	BZCYYb	650	-3.2	30
		600	-1.62	
		550	-0.68	
PBCI-BCIn	BZCYYb	650	-2.82	39
		600	-1.78	
		550	-0.84	
PBCFC-BaCeO	BZCYYb	650	-3.45	40
		600	-1.73	
		550	-	
N-BCFZYNF	BZCYYb	650	-1.57	41
		600	-1.08	
		550	-0.68	
BCO-LSCF	BZCYYb	650	-2.3	42
		600	-1.8	
		550	-1.0	
NCFN-NiO	BZCYYb	650	-2.8	43
		600	-1.6	
		550	-0.7	

BCO-BCMN	BZCYYb	650	2.04	44
		600	1.34	
		550	0.68	
PBCFIn05	BZCYYb	650	-3.72	This Work
		600	-2.16	
		550	-0.81	

Supplementary Table 12: Statistical standard deviations for FE. Each test was conducted for three times to get the statistical standard deviations, presented as error bars.

Current density ($A\ cm^{-2}$)	GC-quantified H_2 (vol. %)	Quantified H_2 SD	FE (%)	FE SD
0.5	11.57	±0.02	92.1	±1.13
1.0	12.93	±0.04	85.9	±1.32
1.5	14	±0.06	78.2	±1.21

Supplementary Reference

1. Funke H, Scheinost A, Chukalina M. Wavelet analysis of extended x-ray absorption fine structure data. *Physical Review B—Condensed Matter and Materials Physics* **71**, 094110 (2005).
2. Funke H, Chukalina M, Scheinost AC. A new FEFF-based wavelet for EXAFS data analysis. *Synchrotron Radiation* **14**, 426-432 (2007).
3. Wang F*, et al.* Enhanced electrochemical performance of $(PrBa)_xFe_{1.9}Nb_{0.1}O_{5+\delta}$ symmetrical electrodes with A-site deficient for symmetrical solid oxide fuel cells. *Ceramics International* **50**, 25697-25707 (2024).
4. Wang B, Long G, Ji Y, Pang M, Meng X. Layered perovskite $PrBa_{0.5}Sr_{0.5}CoCuO_{5+\delta}$ as a cathode for intermediate-temperature solid oxide fuel cells. *Journal of alloys and compounds* **606**, 92-96 (2014).
5. Huang X, Feng J, Abdellatif HR, Zou J, Zhang G, Ni C. Electrochemical evaluation of double perovskite $PrBaCo_{2-x}MnxO_{5+\delta}$ (x=0,0.5,1) as promising cathodes for IT-SOFCs. *International Journal of Hydrogen Energy* **43**, 8962-8971 (2018).
6. Jin F, Shen Y, Wang R, He T. Double-perovskite $PrBaCo_{2/3}Fe_{2/3}Cu_{2/3}O_{5+\delta}$ as cathode material for intermediate-temperature solid-oxide fuel cells. *Journal of Power Sources* **234**, 244-251 (2013).
7. Fu D, Jin F, He T. A-site calcium-doped $Pr_{1-x}CaxBaCo_2O_{5+\delta}$ double perovskites as cathodes for intermediate-temperature solid oxide fuel cells. *Journal of power sources* **313**, 134-141 (2016).
8. Zou J, Park J, Kwak B, Yoon H, Chung J. Effect of Fe doping on $PrBaCo_2O_{5+\delta}$ as cathode for intermediate-temperature solid oxide fuel cells. *Solid State Ionics* **206**, 112-119 (2012).
9. Zhang Y*, et al.* Enhanced oxygen reduction kinetics of IT-SOFC cathode with $PrBaCo_2O_{5+\delta}/Gd_{0.1}Ce_{1.9}O_{2-\delta}$ coherent interface. *Journal of Materials Chemistry A* **10**, 3495-3505 (2022).
10. Idrees A*, et al.* An optimized synthesis route for high performance composite cathode based on a layered perovskite oxide of $PrBa_{0.92}Co_2O_{6-\delta}$ with cationic deficiency. *International Journal of Hydrogen Energy* **44**, 4271-4280 (2019).
11. Zhang W*, et al.* $La_{0.6}Sr_{0.4}Co_{0.2}Fe_{0.8}O_{3-\delta}/CeO_2$ heterostructured composite nanofibers as a highly active and robust cathode catalyst for solid oxide fuel cells. *ACS applied materials & interfaces* **11**, 26830-26841 (2019).
12. Zhu W, Wang H, Xu L, Yuan J, Gong J, Liu X. $Pr_{0.7}Ba_{0.3}Co_{0.8}$-$xFe_{0.2}NixO_{3-\delta}$ perovskite: High activity and durable cathode for intermediate-to-low-temperature proton-conducting solid oxide fuel cells. *International Journal of Hydrogen Energy* **48**, 33633-33643 (2023).
13. Zhao L*, et al.* Novel layered perovskite oxide $PrBaCuCoO_{5+\delta}$ as a potential cathode for intermediate-temperature solid oxide fuel cells. *Journal of Power Sources* **195**, 453-456 (2010).
14. Li M*, et al.* A catalytic barrier strategy to combat chromium-induced degradation in SOFC cathodes. *International Journal of Hydrogen Energy* **170**, 151220 (2025).
15. Pagliari M*, et al.* Electrochemical performance of LSM-YSZ SOFC cathodes: activation, durability, and tolerance to CO_2. *Journal of Power Sources* **649**, 237458 (2025).
16. Song X, Wang C, Xu N, Xu Z, Meng J. Study on the enhanced cathodic performance of BZCYYb-based SOFCs by A-site deficient LSCFN. *International Journal of Hydrogen Energy* **100**, 519-527 (2025).
17. Tao Y, Zhou Y, Li W, Shao J, Bai L, Liu X. Intermediate-temperature solid oxide fuel cells with high performance cobalt-doped $Pr_{0.5}Ba_{0.5}FeO_{3-\delta}$ anodes. *Journal of Alloys and Compounds* **741**, 1091-1097 (2018).

18. Meng Y, *et al.* Novel cobalt and strontium-free perovskite Pr0. 5Ba0. 5Fe1-x Ni x O3-δ (x= 0 and 0.2) as cathode for intermediate-temperature solid oxide fuel cells. *Ionics* **27**, 3951-3965 (2021).
19. Zhang H-X, Yang J-X, Wang P-F, Yao C-G, Yu X-D, Shi F-N. Novel cobalt-free perovskite $PrBaFe_{1.9}Mo_{0.1}O_{5+\delta}$ as a cathode material for solid oxide fuel cells. *Solid State Ionics* **391**, 116144 (2023).
20. Song R, *et al.* Sr and Mn co-doping $PrBaFe_2O_{5+\delta}$ optimizes the electrochemical performance and stability of solid oxide fuel cell cathode. *Journal of Alloys and Compounds* **1005**, 176231 (2024).
21. Bai J, *et al.* Sc-doped Co-based perovskites as IT-SOFC cathode with high oxygen reduction reaction and CO_2 tolerance. *Solid State Ionics* **402**, 116377 (2023).
22. Liu Y, *et al.* Characterization of A-site doped $PrBaCo_2O_{5+\delta}$ perovskites as cathode materials for IT-SOFCs. *Ceramics International* **50**, 52904-52916 (2024).
23. Gao Y, *et al.* Self-Assembled Composite Cathodes with TEC Gradient for Proton-Conducting Solid Oxide Fuel Cells. *Advanced Functional Materials* **35**, 2416625 (2025).
24. Tang S, Fu M, Qin Z, Gao Y, Tao Z. Topological Ion Optimized Composite Cathode for Proton-Conducting Solid Oxide Fuel Cells. *Advanced Functional Materials* **35**, 2501995 (2025).
25. Zhao S, *et al.* Single-Atom Enables Reverse Hydrogen Spillover for High-Performance Protonic Ceramic Fuel Cells. *Advanced Materials* **37**, 2501387 (2025).
26. Wan B, Liu T, Chang Y, Fu M, Tao Z, Zhou H. Thermodynamic NiO exsolution for durable and efficient cobalt-free cathodes in proton-conducting solid oxide fuel cells. *Journal of Advanced Ceramics* **15**, 9221199 (2024).
27. Du Z, *et al.* Indium-Doping-Induced Nanocomposites with Improved Oxygen Reaction Activity and Durability for Reversible Protonic Ceramic Electrochemical Cell Air Electrodes. *Advanced Functional Materials* **34**, 2409188 (2024).
28. Hu T, *et al.* In situ engineering of a cobalt-free perovskite air electrode enabling efficient reversible oxygen reduction/evolution reactions. *Advanced Functional Materials* **33**, 2305567 (2023).
29. Fu M, Gao Y, Zhang M, Shi N, Tao Z, Shao Z. Vanadium-assisted surface engineering of heterostructured cathode for enhanced protonic ceramic fuel cell performance. *Chemical Engineering Journal* **505**, 159722 (2025).
30. Rao J, *et al.* In-situ phase formation enables graded thermal expansion and structural stability in reversible protonic ceramic electrochemical cell cathodes. *Materials Reports: Energy*, 100420 (2026).
31. Hu L, *et al.* A high-performance composite cathode based on thermal expansion complementation for SOFC. *Fuel* **362**, 130864 (2024).
32. Zhu K, *et al.* A superior catalytic air electrode with temperature-induced exsolution toward protonic ceramic cells. *ACS nano* **18**, 5141-5151 (2024).
33. Xu Y, *et al.* A low-lewis-acid-strength cation Cs+-Doped double perovskite for fast and durable oxygen reduction/evolutions on protonic ceramic cells. *ACS Energy Letters* **8**, 4145-4155 (2023).
34. Zhou Y, *et al.* An active and robust air electrode for reversible protonic ceramic electrochemical cells. *ACS Energy Letters* **6**, 1511-1520 (2021).
35. Pei K, *et al.* Surface restructuring of a perovskite-type air electrode for reversible protonic ceramic electrochemical cells. *Nature communications* **13**, 2207 (2022).
36. Xia J, Zhu F, He F, Xu K, Choi Y, Chen Y. Self-configured composites of ruddlesden-popper perovskite and Pr6O11 as efficient and durable air electrodes for reversible protonic ceramic electrochemical cells. *Advanced Energy Materials* **13**, 2302964 (2023).

37. Saqib M, *et al.* Transition from perovskite to misfit-layered structure materials: a highly oxygen deficient and stable oxygen electrode catalyst. *Energy & Environmental Science* **14**, 2472-2484 (2021).
38. He F, *et al.* An efficient high-entropy perovskite-type air electrode for reversible oxygen reduction and water splitting in protonic ceramic cells. *Advanced Materials* **35**, 2209469 (2023).
39. Du Z, *et al.* Indium-Doping-Induced Nanocomposites with Improved Oxygen Reaction Activity and Durability for Reversible Protonic Ceramic Electrochemical Cell Air Electrodes. *Advanced Functional Materials* **34**, 2409188 (2024).
40. Hu T, *et al.* In Situ Engineering of a Cobalt-free Perovskite Air Electrode Enabling Efficient Reversible Oxygen Reduction/Evolution Reactions. *Advanced Functional Materials* **33**, 2305567 (2023).
41. Chen X, *et al.* Synergistic Bulk and Surface Engineering for Expeditious and Durable Reversible Protonic Ceramic Electrochemical Cells Air Electrode. *Advanced Materials* **36**, 2403998 (2024).
42. Zhou Y, *et al.* An Efficient Bifunctional Air Electrode for Reversible Protonic Ceramic Electrochemical Cells. *Advanced Functional Materials* **31**, 2105386 (2021).
43. Pei K, *et al.* Surface restructuring of a perovskite-type air electrode for reversible protonic ceramic electrochemical cells. *Nature Communications* **13**, 2207 (2022).
44. He F, *et al.* Catalytic Self-Assembled Air Electrode for Highly Active and Durable Reversible Protonic Ceramic Electrochemical Cells. *Advanced Functional Materials* **32**, 2206756 (2022).
