# Supplementary material for: Spin-coordinated mixed-valence transport via indium-induced perovskite-spinel heterointerface in oxygen electrode for reversible protonic ceramic cells
Source: Nat Commun. 2026 Jul 15;17:8686. doi: 10.1038/s41467-026-75684-4 (PMC13490552; doi:10.1038/s41467-026-75684-4)
Supplement: Supplementary file 2 [file 41467_2026_75684_MOESM2_ESM.pdf]

Description of Additional Supplementary Files

Title: Supplementary Data 1

Description: Optimized atomic coordinates of the PBCF/Co_3O_4 heterointerface model used for the charge density difference calculations.

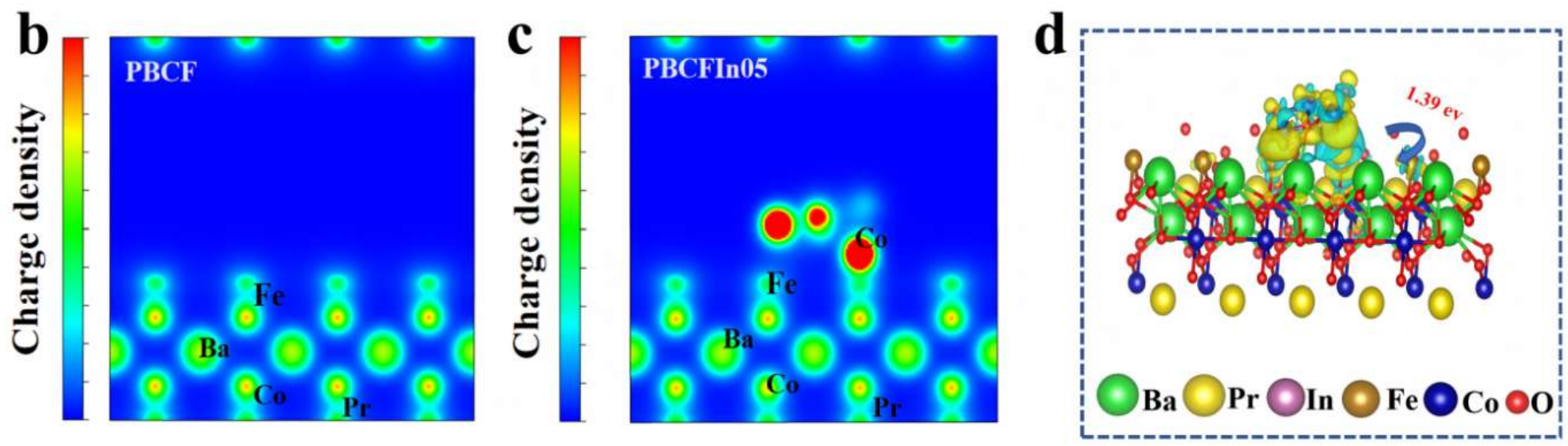

Fig. 6 d Differential charge density in PBCF and PBCFIn05

Title: Supplementary Data 2

Description: Optimized atomic coordinates of the computational models used in the DFT calculations presented for PBCF

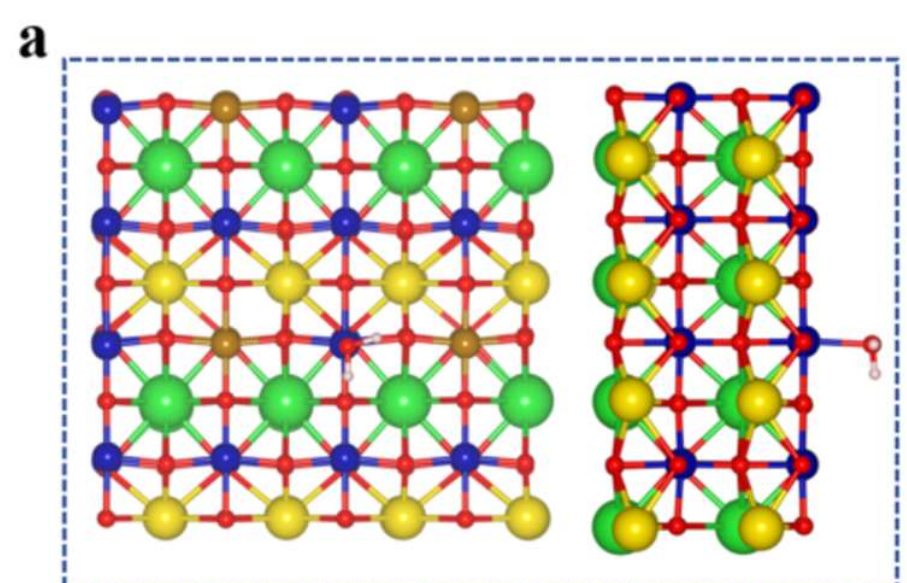

Supplementary Fig. 31 Optimized surface terminations and adsorption geometries of key OER/ORR intermediates on PBCF. (a)*H_2O

Title: Supplementary Data 3(initial); 4(final)

Description: Optimized atomic coordinates of the computational models used in the DFT calculations presented for PBCF CI-NEB TS1.

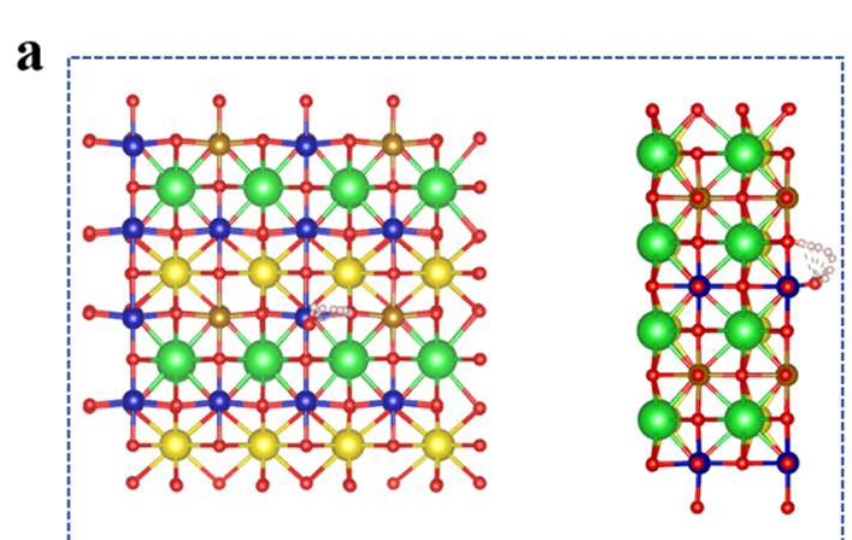

Supplementary Fig. 32. CI-NEB calculations of the **a)***H_2O → *OH + H

Title: Supplementary Data 5

Description: Optimized atomic coordinates of the computational models used in the DFT calculations presented for PBCF

b

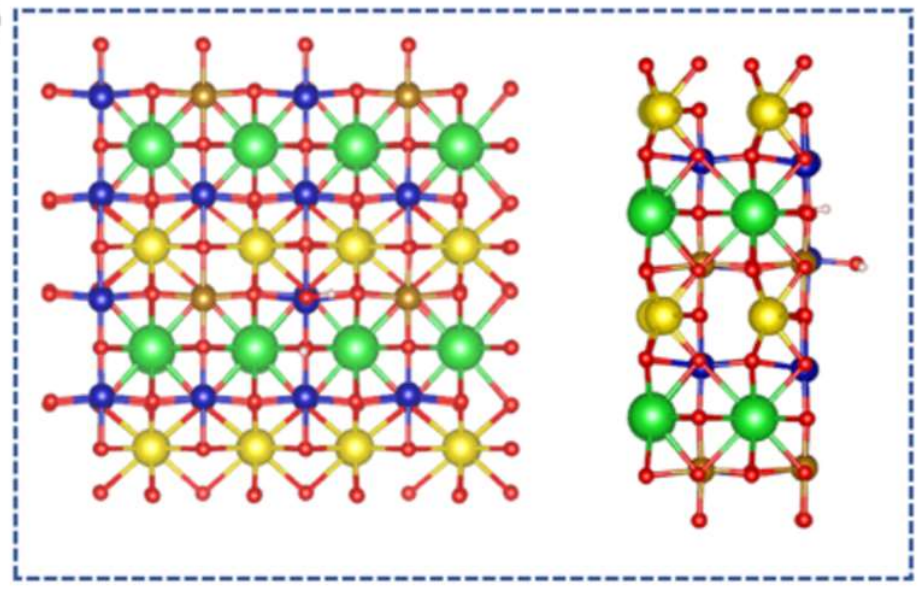

Supplementary Fig. 31 Optimized surface terminations and adsorption geometries of key OER/ORR intermediates on PBCF. (b)*OH+*H

Title: Supplementary Data 6

Description: Optimized atomic coordinates of the computational models used in the DFT calculations presented for PBCF

c

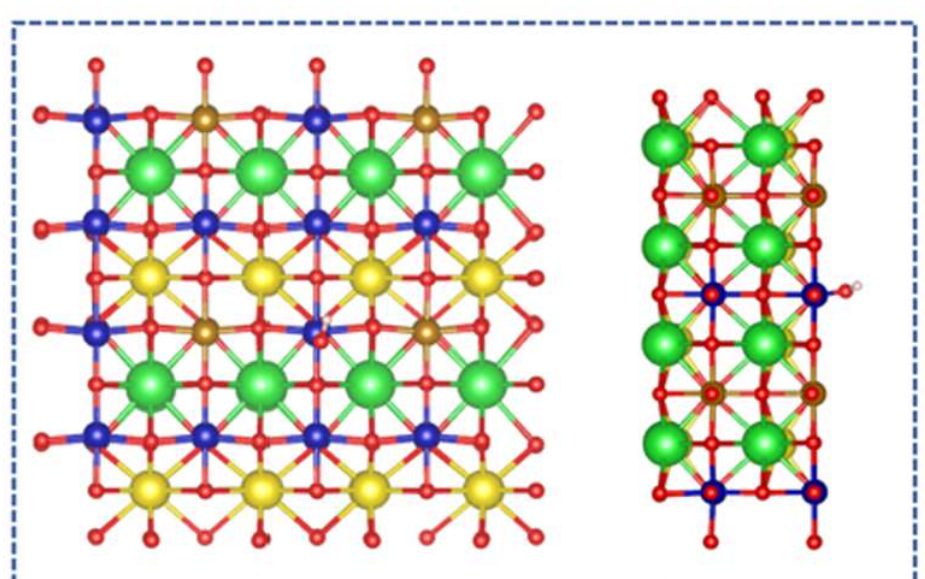

Supplementary Fig. 31 Optimized surface terminations and adsorption geometries of key OER/ORR intermediates on PBCF. (c) *OH

Title: Supplementary Data 7(initial); 8(final)

Description: Optimized atomic coordinates of the computational models used in the DFT calculations presented for PBCF CI-NEB TS1.

b

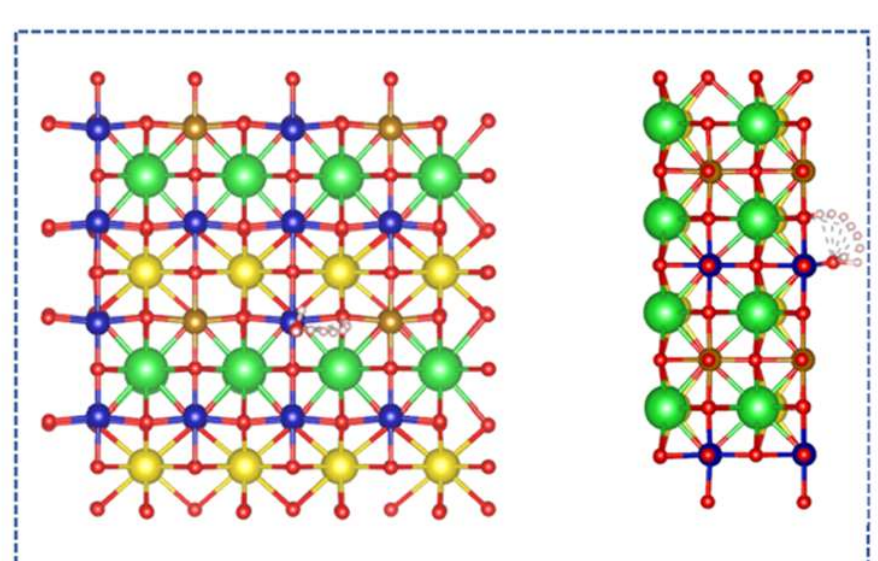

Supplementary Fig. 32. CI-NEB calculations of the **b)** *OH → *O + H

Title: Supplementary Data 9

Description: Optimized atomic coordinates of the computational models used in the DFT calculations presented for PBCF

d

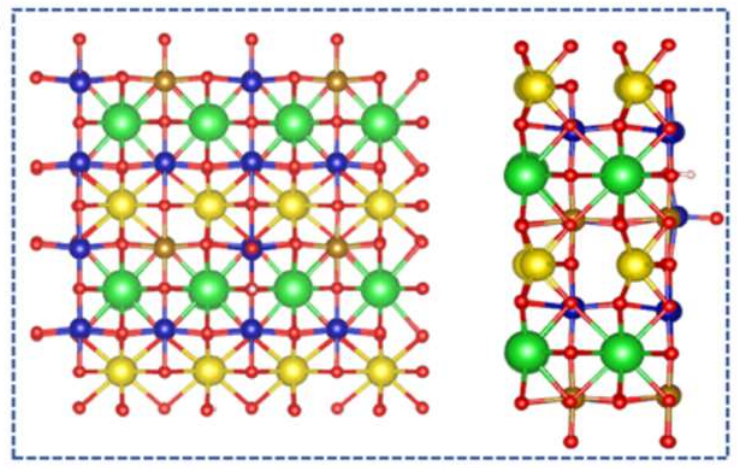

Supplementary Fig. 31 Optimized surface terminations and adsorption geometries of key OER/ORR intermediates on PBCF. (d) *O+*H

Title: Supplementary Data 10

Description: Optimized atomic coordinates of the computational models used in the DFT calculations presented for PBCF

e

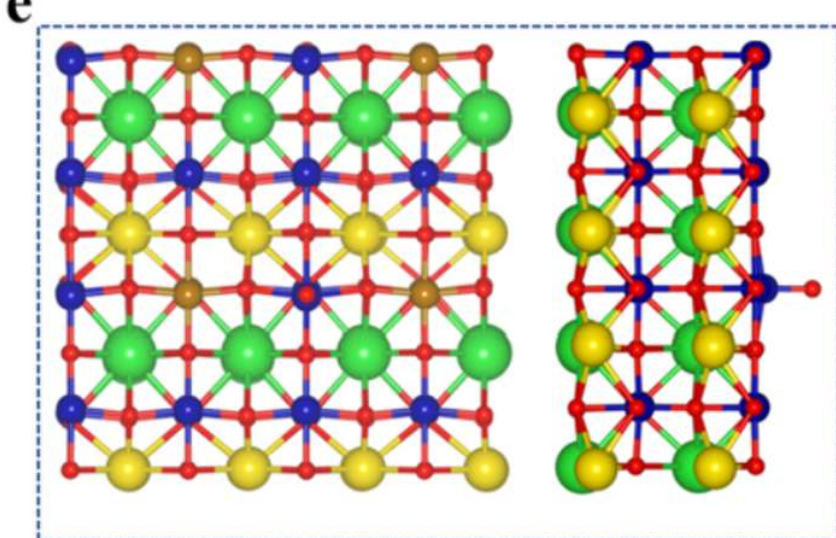

Supplementary Fig. 31 Optimized surface terminations and adsorption geometries of key OER/ORR intermediates on PBCF. (e) *O

Title: Supplementary Data 11

Description: Optimized atomic coordinates of the computational models used in the DFT calculations presented for PBCF

f

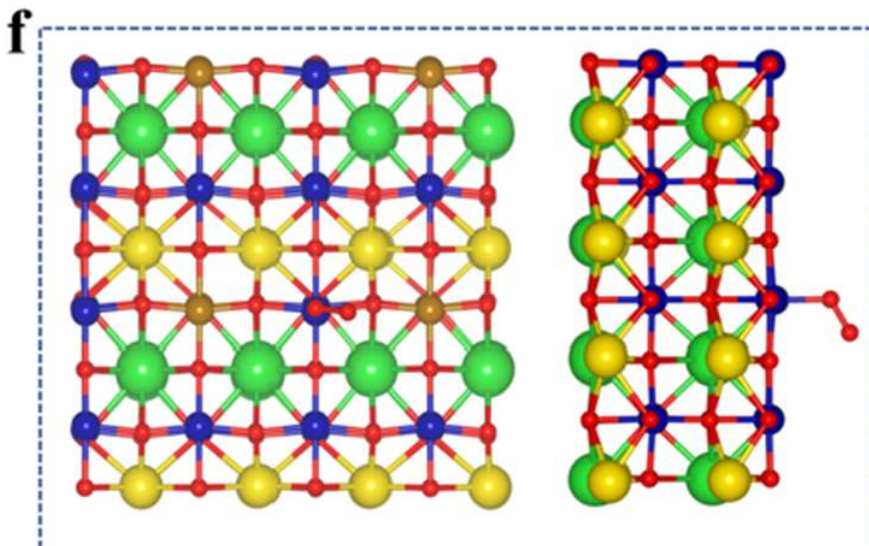

Supplementary Fig. 31 Optimized surface terminations and adsorption geometries of key OER/ORR intermediates on PBCF. (e) *OO

Title: Supplementary Data 12

Description: Optimized atomic coordinates of the computational models used in the DFT calculations presented for PBCFIn05

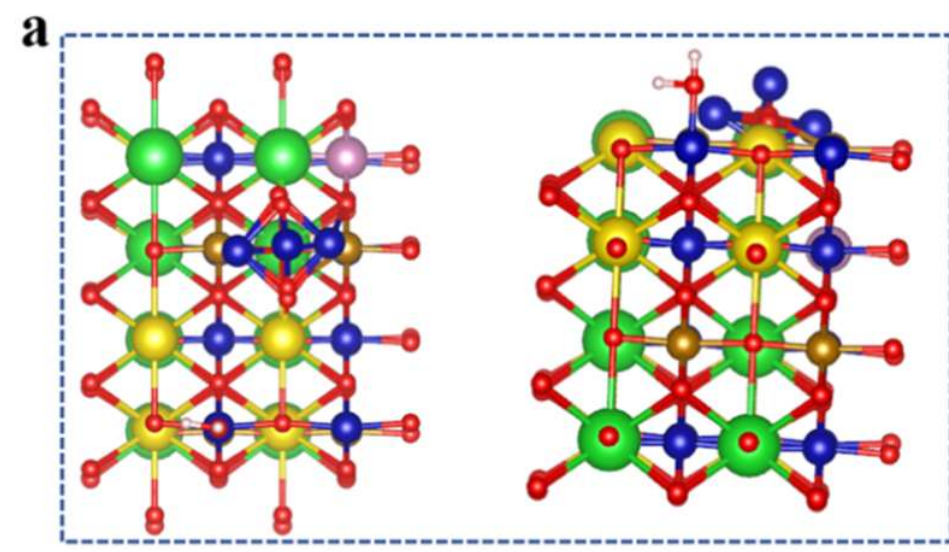

Supplementary Fig. 30 Optimized surface terminations and adsorption geometries of key OER/ORR intermediates on PBCFIn05 (a)*H_2O

Title: Supplementary Data 13(initial); 14(final)

Description: Optimized atomic coordinates of the computational models used in the DFT calculations presented for PBCFIn05 CI-NEB TS1.

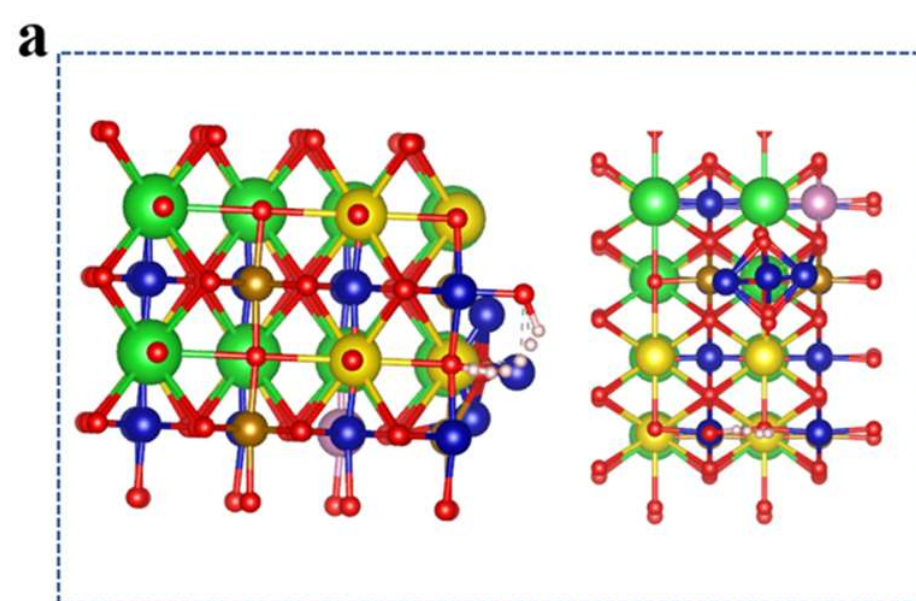

Supplementary Fig. 33. CI-NEB calculations of the **a)***H_2O → *OH + H

Title: Supplementary Data 15

Description: Optimized atomic coordinates of the computational models used in the DFT calculations presented for PBCFIn05

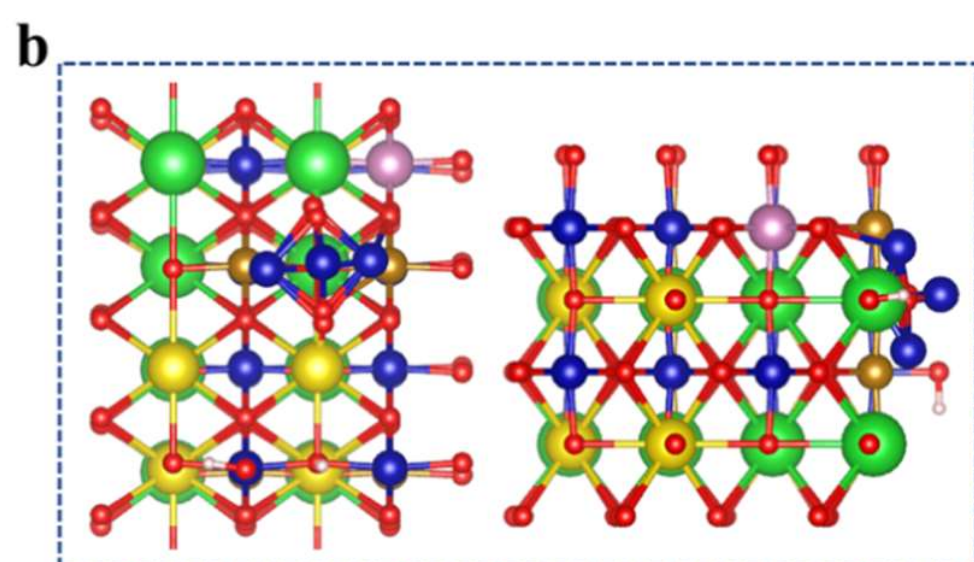

Supplementary Fig. 30 Optimized surface terminations and adsorption geometries of key OER/ORR intermediates on PBCFIn05 (b)*OH+*H

Title: Supplementary Data 16

Description: Optimized atomic coordinates of the computational models used in the DFT calculations presented for PBCFIn05

c

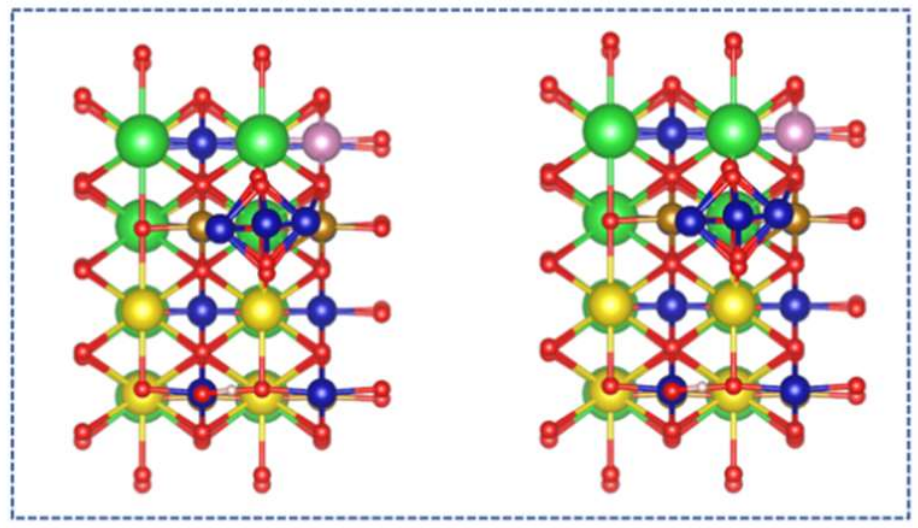

Supplementary Fig. 30 Optimized surface terminations and adsorption geometries of key OER/ORR intermediates on PBCFIn05 (c)*OH

Title: Supplementary Data 17(initial); 18(final)

Description: Optimized atomic coordinates of the computational models used in the DFT calculations presented for PBCFIn05

b

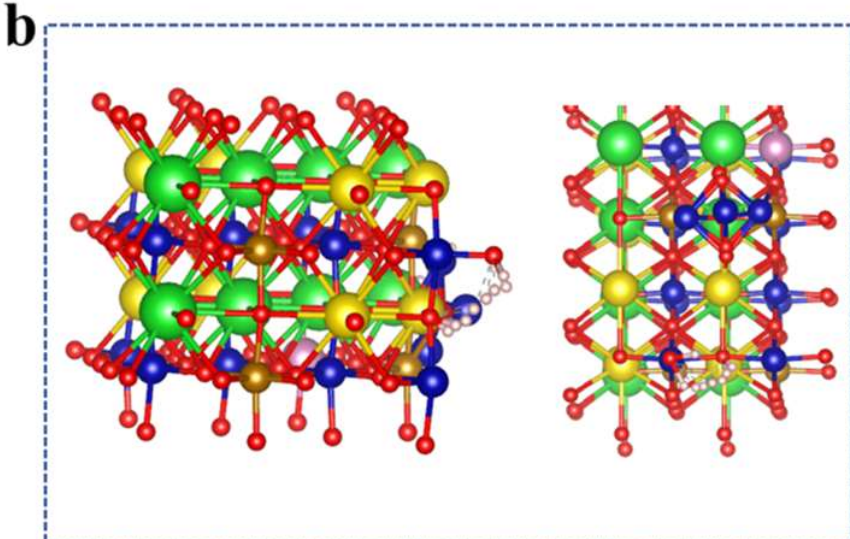

Supplementary Fig. 33. CI-NEB calculations of the **b)***OH → *O + H

Title: Supplementary Data 19

Description: Optimized atomic coordinates of the computational models used in the DFT calculations presented for PBCFIn05

d

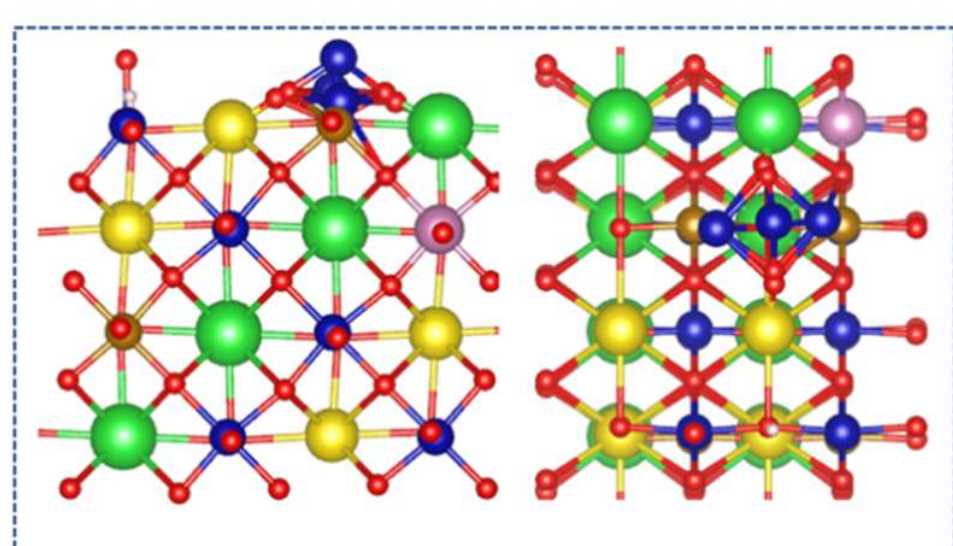

Supplementary Fig. 30 Optimized surface terminations and adsorption geometries of key OER/ORR intermediates on PBCFIn05 (d)*O+*H

Title: Supplementary Data 20

Description: Optimized atomic coordinates of the computational models used in the DFT calculations presented for PBCFIn05

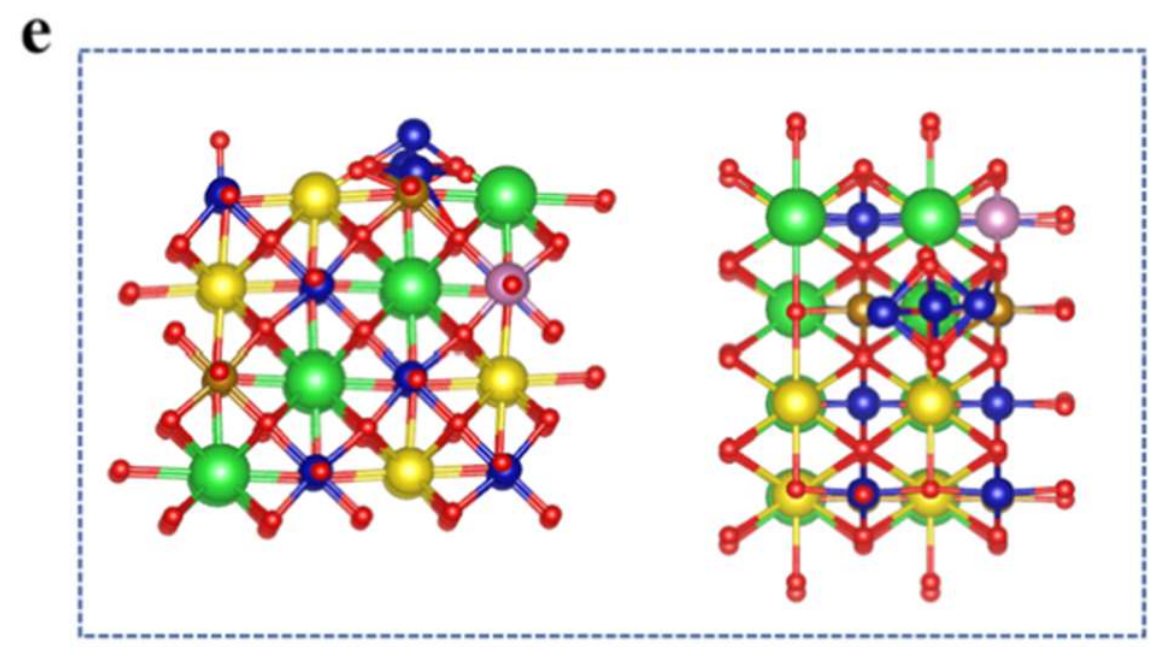

Supplementary Fig. 30 Optimized surface terminations and adsorption geometries of key OER/ORR intermediates on PBCFIn05 (e) *O

Title: Supplementary Data 21

Description: Optimized atomic coordinates of the computational models used in the DFT calculations presented for PBCFIn05

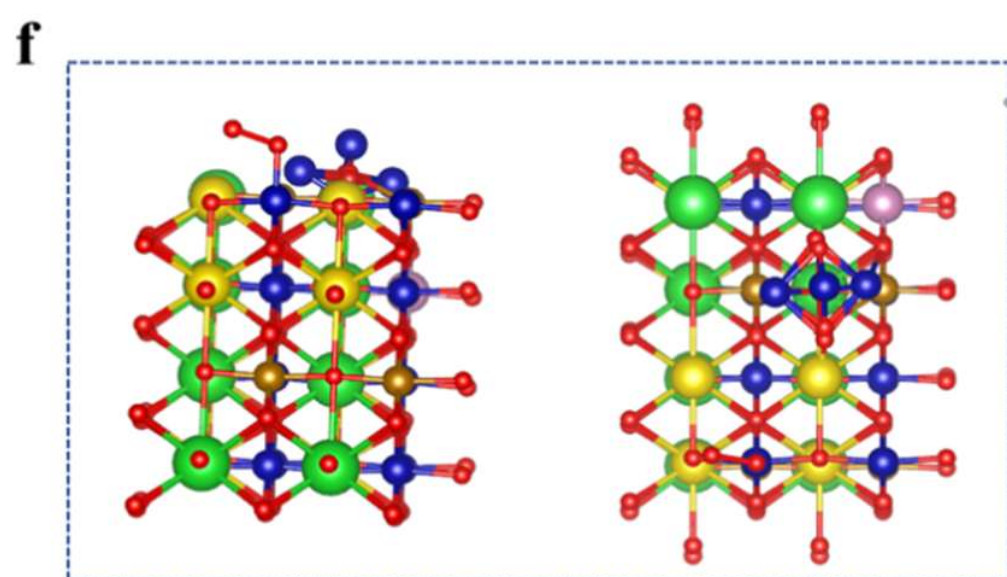

Supplementary Fig. 30 Optimized surface terminations and adsorption geometries of key OER/ORR intermediates on PBCFIn05 (e) *OO

Title: Supplementary Data 22

Description: DFT calculation data for the oxygen vacancy formation energy of the pristine PBCF model.

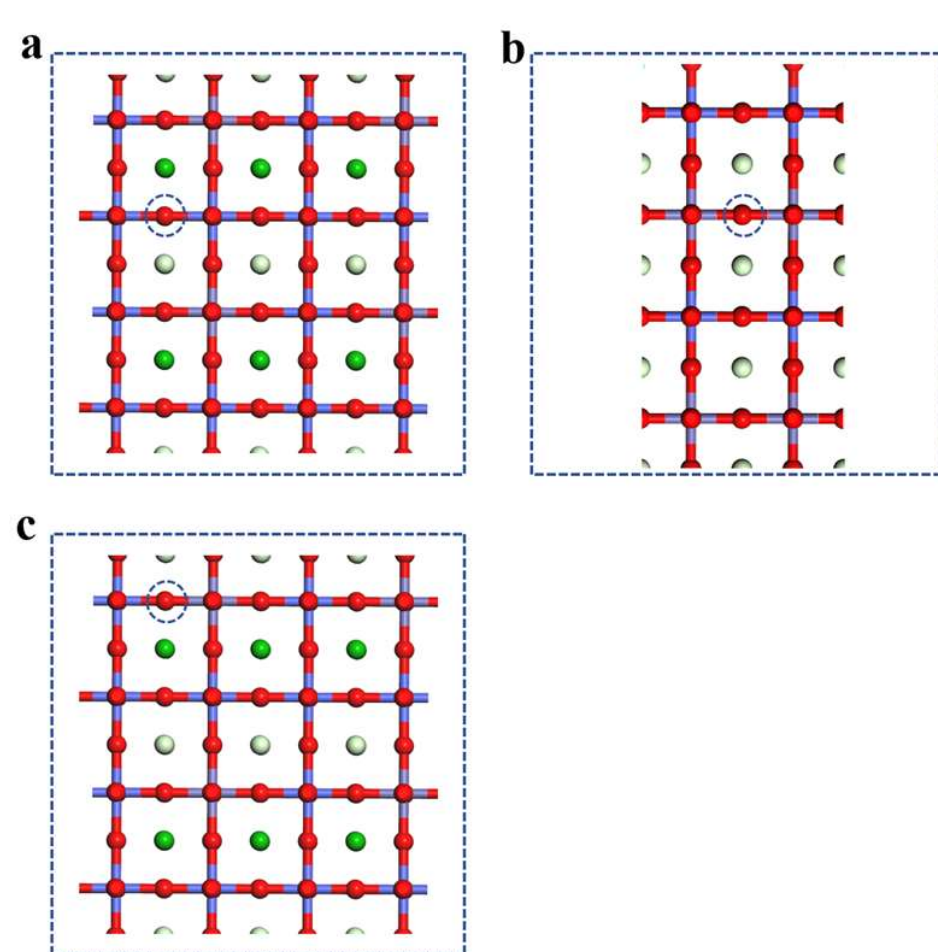

Supplementary Fig. 14. The adsorption geometries of oxygen vacancies at different lattice sites in PBCFIn05. (a) Co-O-Co, (b) Fe-O-Fe, (c) Fe-O-Co.

Title: Supplementary Data 23

Description: DFT calculation data for the oxygen vacancy formation energy of the pristine PBCFIn05 model.

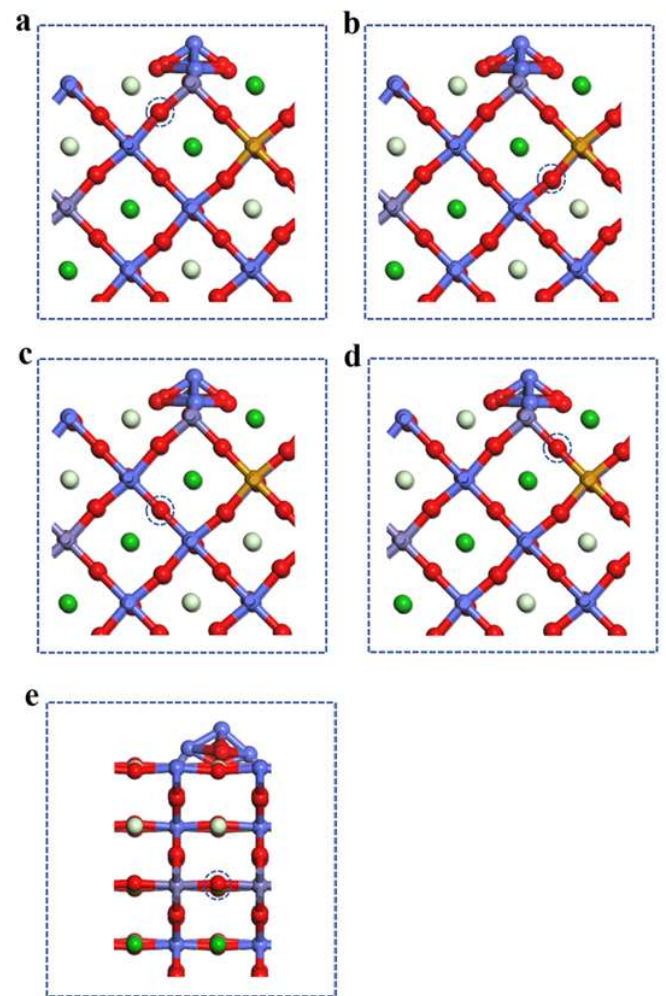

Supplementary Fig. 13. The adsorption geometries of oxygen vacancies at different lattice sites in PBCFIn05. (a) Fe-O-Co, (b) In-O-Co, (c) CO-O-Co, (d) Fe-O-In, (e) Fe-O-Fe.
