# Supplementary material for: Spin-coordinated mixed-valence transport via indium-induced perovskite-spinel heterointerface in oxygen electrode for reversible protonic ceramic cells
Source: Nat Commun. 2026 Jul 15;17:8686. doi: 10.1038/s41467-026-75684-4 (PMC13490552; doi:10.1038/s41467-026-75684-4)
Supplement: Supplementary file 3 [file 41467_2026_75684_MOESM4_ESM.pdf]

nature portfolio

Peer Review File

Spin-coordinated mixed-valence transport via Indium-induced perovskite-spinel heterointerface in oxygen electrode for reversible protonic ceramic cells

Corresponding Author: Professor Hanping Ding

This file contains all reviewer reports in order by version, followed by all author rebuttals in order by version.

Version 0:

Reviewer comments:

Reviewer #1

(Remarks to the Author)
The manuscript reports an Indium-modified $Pr_{0.5}Ba_{0.5}Co_{0.7}Fe_{0.3}O_{3-\delta}$ (PBCFInx) air electrode that achieves simultaneous gains in ORR/OER activity and durability for R-PCEC operation. The idea of using In to tune spin and covalence is novel. The manuscript is well structured, presents comprehensive data, and reaches convincing conclusions, with a clear throughline from materials chemistry to cell-level performance.

I would recommend this work for publication once the following comments have been addressed.

1 The manuscript attributes the FE decrease at higher electrolysis currents to "a drop in PO2 on the fuel side, which enhances the electrolyte's electronic conductivity and leads to electron leakage." This causal is currently under-explained. Please further elaborate this mechanism and support it with appropriate references.

2 Supplementary Fig. S15. The authors observe that, in electrolysis mode, the resistance decreases with increasing steam partial pressure. Please provide a detailed mechanistic explanation for this trend and discuss whether this resistance reduction could impact the Faradaic efficiency.

3 The manuscript assigns LF (<10 Hz) to gas diffusion, IF (10–2000 Hz) to surface oxygen exchange, and HF (>2000 Hz) to charge transfer without citation. Please provide appropriate references supporting these frequency-domain attributions for SOFC/PCFC electrodes

4 Cell fabrication details." the cells were calcined at 1000 °C for 3h to achieve strong interfacial bonding between the cathode and electrolyte." Which atmosphere? Please specify the atmosphere and report the heating rates.

5 In Figure 5l, the authors compared PBCFIn05 a single cell with single cells using other electrode materials. However, the temperature of 700°C is not clearly marked in the figure. Revising this figure would help avoid potential misunderstandings.

6 Fig. 5 only provides stability test data for PBCFIn05 after temperature cycling. To enhance the authors' arguments and more comprehensively demonstrate the material's properties, SEM images before and after temperature cycling should also be included.

7 The Methods section describes only single-cell fabrication, yet symmetric cells are used in the study. Please provide a detailed fabrication protocol for the symmetric cells and include SEM images of the symmetric electrodes. This will improve reproducibility and enable a clean comparison of electrode microstructure.

Reviewer #2

(Remarks to the Author)
This manuscript presents a method called spin-coordinated heterointerface engineering to improve oxygen electrocatalysis in protonic ceramic electrochemical cells. The authors attempt to link bulk charge transport with surface redox reactions by

establishing a mixed-valence pathway. This is achieved by forming a Co_3O_4 spinel layer through the addition of indium to $Pr_{0.5}Ba_{0.5}Co_{0.7}Fe_{0.3}O_3$, improving both oxygen reduction and evolution performance. The following comments and suggestions are provided to help improve the scientific quality of the manuscript.

1. The experimental part of this study is comprehensive, utilizing a wide range of characterization techniques. However, several important issues reduce the reliability of the conclusions. First, the phase purity and reproducibility of the synthesized materials are not clearly demonstrated. Although XRD and Rietveld refinement confirm the coexistence of perovskite and Co_3O_4 phases, the quantitative composition, spatial uniformity, and structural continuity of the spinel layer are not verified. In addition, the proposed “stress-assisted cation segregation” mechanism for Co_3O_4 formation remains qualitative and is not supported by direct compositional or thermodynamic evidence. A more detailed discussion is recommended.
2. Although the electrochemical results show good performance, important methodological details are missing. Faradaic efficiency is discussed but not confirmed by quantitative gas analysis, which creates uncertainty about reaction selectivity. Also, the testing conditions and data reproducibility are not well documented. A more detailed explanation/discussion is needed to support the electrochemical findings and to clarify whether the observed improvements are due to the material’s intrinsic catalytic activity or experimental variation.
3. Many recent computational studies on PBC- and Co, Fe-based perovskites have shown that accurate O 2p-band center values require either on-site correlation treatment (DFT+U) or hybrid exchange methods (HSE06). For example, DFT+U studies usually report O 2p-band centers in the range of -2.x eV, while HSE06 calculations show values in the range of -3.x eV relative to the Fermi level in recent reports. In contrast, the manuscript reports a much deeper value of -5.04 eV for pristine PBCF, which does not match these reference values and suggests the use of PBE without a U correction. Since the O 2p-band center is very sensitive to the exchange-correlation functional and the treatment of Co/Fe 3d electrons, the authors should: (i) clearly state which functional was used; (ii) explain why on-site correlation corrections were not applied; and (iii) discuss how this choice affects the accuracy of the band-center values. Ideally, recalculating the projected DOS using PBE+U or HSE06 would give more reliable values for comparison with the literature. Doping only the In atom may not be able to alter the O 2p-band center from -5.04 eV to -2.74 eV. Please refer to Jacobs et al., Advanced Energy Materials 8, 1702708 (2018) and their other publications.
4. It should be noted that the Computational Hydrogen Electrode (CHE) framework, while widely used in aqueous electrocatalysis, has been increasingly and incorrectly used in high-temperature solid oxide and protonic ceramic fuel cells. Several recent papers, including some by well-known groups, have presented CHE-based OER/ORR free-energy diagrams for perovskite oxides working at high temperatures. However, this approach does not align with the actual reaction mechanisms in such systems, as CHE assumes proton-electron transfer in a liquid environment, rather than oxide-ion movement or lattice oxygen redox in the solid state. The current manuscript appears to follow this incorrect approach by presenting a CHE-based thermodynamic diagram. The authors should carefully reconsider this modeling method and, if possible, use a solid-state thermodynamic framework supported by mechanistic studies such as transition-state calculations. It is suggested to refer to the original papers reported by Norskov et al. This point also relates to the comment below about CI-NEB (see Comment 5).
5. The authors state in the Computational Methods section that the CI-NEB method was used to calculate reaction energetics. However, the energy diagrams in the manuscript clearly follow the CHE model, showing free-energy changes for *OH, *O, and *OOH intermediates instead of transition-state barriers. These two approaches describe different physical quantities: CI-NEB provides activation energies along the reaction path, while CHE shows equilibrium thermodynamic data. If CI-NEB was actually used, the corresponding transition-state energies and migration barriers should be clearly reported.
6. For the reference section, several important original references appear to have been omitted, and some cited computational methods were inaccurately interpreted or incorrectly applied. This suggests possible misuse or neglect of key literature, which reduces confidence in the validity of the computational results and may have contributed to inconsistent or misleading correlations with the experimental findings.

Reviewer #3

(Remarks to the Author)
The manuscript presents an interesting concept that highly efficient oxygen electrode in protonic ceramic electrochemical cells requires not only enhanced surface reaction kinetics but also precise coordination of electronic and spin states across the bulk–interface continuum. The authors introduce spin-coordinated heterointerface engineering and report that In doping in PBCF (PBCFIn05) induces formation of a Co_3O_4 spinel phase accompanied by selective Co migration and interfacial reconstruction. This heterointerface is proposed to reduce the activation barriers for both the ORR and OER, thereby improving catalytic activity and enabling application in a R-PCEC platform. While the concept and the reported performance are promising, the current version of the manuscript does not yet provide the level of mechanistic clarity, experimental validation, and overall completeness required for publication in this journal.

Some specific concerns are as follows:
1. The manuscript refers to indium-modified variants as $Pr_{0.5}Ba_{0.5}Co_{0.7}Fe_{0.3}O_{3-\delta}$–$In_{0.05}$ (PBCFIn05) and states that In^{3+} is introduced at the B-site to redistribute lattice valence and covalency while promoting the in situ formation of a self-assembled Co_3O_4 interfacial phase. However, the use of the term “indium-modified” is ambiguous, as it is unclear whether indium is incorporated as an excess B-site dopant or as a substitutional dopant within the B-site lattice.
2. Rietveld refinements of the XRD data (Fig. 2b, Supplementary Fig. S2, and Table 1) assign all compositions to a tetragonal perovskite structure. However, this assignment is not reasonable for a 3-delta system and likely reflects an incorrect structural model. Therefore, the conclusion that all materials are tetragonal perovskite appears to be simply wrong and should be re-examined.

3. Although the authors analyze the electronic structure and surface valence-state distribution using XAS, XPS, and related techniques and correlate these results with ORR/OER catalytic activity, the developed air electrode is ultimately applied in R-PCECs However, the manuscript provides no experimental characterization relevant to protonic properties, such as hydration behavior, proton uptake, or proton conductivity of the air electrode.
4. Regarding the electrochemical measurements, most of the measurements and analyses were conducted at temperature above 600 °C, which is fundamentally incorrect. At such temperatures, the electrolyte can no longer sustain predominantly protonic conduction. Therefore, to ensure relevance to R-PCEC operation, the electrochemical performance should be re-measured and re-analyzed at lower temperatures.
5. In Fig. 5j, the stability test in fuel cell (FC) mode appears to have been conducted under an excessively low applied bias, which limits the significance of the stability evaluation. Moreover, although the authors mentioned that the initial minor performance decay observed in FC mode may stem from transient surface-site deactivation or interfacial reaction kinetics, the performance in fact shows a monotonic decline rather than a transient drop followed by stabilization. This behavior suggests the presence of a more persistent degradation process. However, no post-analysis is provided to determine whether the decay originates from interfacial degradation, electrode surface evolution, or other factors.
6. In Fig. 5k, the authors report thermal cycling durability tests under three distinct heating–cooling protocols; however, the corresponding temperature ranges are not specified, rendering the experimental conditions unclear and the results difficult to interpret. Moreover, thermal cycling durability is not particularly relevant to the main scope of this study. Given that the proposed air electrode is intended for use in a reversible protonic ceramic electrochemical cell (R-PCEC), stability evaluations under electrolysis mode and reversible (fuel cell/electrolysis) operation would be far more appropriate and scientifically meaningful.
7. Recently, many studies have been published reporting dual-phase composite electrodes with concepts similar to the present work to enhance electrochemical performance. However, the literature cited for the electrochemical performance comparison (Fig. 5l, Supplementary Fig. 20, and Supplementary Tables 8 and 9) is largely outdated and does not include these more recent and conceptually related studies. As a result, the performance benchmarking is incomplete and weakens the credibility of the authors' performance claims.

Minor points:
1. The chemical compatibility with the electrolyte has not been evaluated in this study.
2. There are many typos and errors throughout the manuscript, including the supporting information, which need to be carefully checked and corrected.

Version 1:

Reviewer comments:

Reviewer #1

(Remarks to the Author)
Accept

Reviewer #2

(Remarks to the Author)
The authors have made substantial efforts to address the reviewers' comments, and most issues have been clarified to the extent possible. However, the section on the energy profile and DFT analysis still requires further revision, as the current presentation is insufficient. The energy diagram shown in Fig. 6e appears schematic and mixes thermodynamic free energies with activation barriers obtained from CI-NEB calculations. In addition, several intermediates (*O and *OO) and the symmetric ORR/OER pathway raise concerns about the physical validity of the proposed reaction mechanism. The authors should clearly define the reference states used in the energy calculations and present the CI-NEB results (i.e., the activation barriers) separately from the thermodynamic energy diagram, for example, by explicitly identifying the transition states (e.g., TS1 and TS2). Furthermore, although reversibility between OER and ORR should, in principle, be consistent with statistical mechanics, such symmetry is generally difficult to reproduce accurately in practical DFT calculations. The current representation in Fig. 6e, therefore, appears somewhat artificial and suggests that the energy diagram may have been constructed schematically rather than being directly derived from DFT-based energetics.
The authors are encouraged to reconstruct the energy profiles by treating OER and ORR separately and to move the detailed energy diagrams to the Supporting Information in order to avoid potential misunderstandings. It is noted that numerous relevant publications are available in the literature. The discussion of this part in the main text should also be minimized unless the methodology and its physical interpretation are clearly justified.

Reviewer #3

(Remarks to the Author)
The current manuscript is suitable for publication.

Version 2:

Reviewer comments:

Reviewer #2

(Remarks to the Author)
The authors have satisfactorily addressed the reviewers' comments, and the manuscript has been substantially improved. The revised DFT analysis is clearer, and the mechanistic discussion is now adequately supported.

Only a minor clarification is recommended before final publication. The terminology used for the reported "energy profiles" should be carefully checked in both the main text and the computational methods. Based on the presented methodology, these profiles appear to be relative DFT energy profiles rather than rigorous "free-energy profiles" obtained from a Computational Hydrogen Electrode (CHE) framework. Therefore, wording such as "free-energy profiles," including in the caption of Fig. 6, should be revised accordingly. In addition, statements such as "Thermodynamic feasibility was examined using the computational hydrogen electrode (CHE) framework to estimate Gibbs free energy changes" in the computational methods should be removed or revised after careful proofreading if CHE was not actually employed. The supplementary tables listing the calculated total energies for Supplementary Tables 13–20 should also be deleted, as they are not necessary for the revised presentation.

Overall, the manuscript is suitable for publication after these minor editorial and terminology corrections.

Version 3:

Reviewer comments:

Reviewer #2

(Remarks to the Author)
The manuscript is suitable for publication. As a minor editorial revision, the authors are encouraged to include the appropriate reference for the CI-NEB method used in the computational analysis.

Open Access This Peer Review File is licensed under a Creative Commons Attribution 4.0 International License, which permits use, sharing, adaptation, distribution and reproduction in any medium or format, as long as you give appropriate credit to the original author(s) and the source, provide a link to the Creative Commons license, and indicate if changes were made.
In cases where reviewers are anonymous, credit should be given to 'Anonymous Referee' and the source.
The images or other third party material in this Peer Review File are included in the article's Creative Commons license, unless indicated otherwise in a credit line to the material. If material is not included in the article's Creative Commons license and your intended use is not permitted by statutory regulation or exceeds the permitted use, you will need to obtain permission directly from the copyright holder.
To view a copy of this license, visit https://creativecommons.org/licenses/by/4.0/

Point-to-point Responses to Reviewers' Comments and Suggestions

We sincerely thank the Editor and the reviewers for their careful evaluation of our manuscript (NCOMMS-25-93329) and for the constructive comments and suggestions. These insightful remarks have helped us significantly improve the clarity and quality of the manuscript. We have carefully revised the manuscript to address all comments. All changes are highlighted in red in the revised version, and detailed point-by-point responses are provided below.

Reviewer # 1

Comments to the Author

The manuscript reports an Indium-modified $Pr_{0.5}Ba_{0.5}Co_{0.7}Fe_{0.3}O_{3-\delta}$ (PBCFInx) air electrode that achieves simultaneous gains in ORR/OER activity and durability for R-PCEC operation. The idea of using In to tune spin and covalence is novel. The manuscript is well structured, presents comprehensive data, and reaches convincing conclusions, with a clear throughline from materials chemistry to cell-level performance. I would recommend this work for publication once the following comments have been addressed.

Response: We sincerely thank the reviewer for the positive evaluation of our manuscript and for recognizing the novelty of using indium to tune spin and covalency, as well as the overall structure, data completeness, and coherence from materials chemistry to cell-level performance. We appreciate the recommendation for publication and have carefully addressed all the comments and suggestions raised by the reviewer, as detailed below. We sincerely thank the reviewer for the positive evaluation of our manuscript and for recognizing the novelty of using indium to tune spin states and metal–oxygen covalency in the PBCF system. We also appreciate the reviewer's comments on the overall organization of the manuscript, the comprehensiveness of the data, and the clear connection established between materials chemistry and cell-level performance. We are grateful for the reviewer's

recommendation for publication. All comments and suggestions have been carefully considered, and the manuscript has been revised accordingly. Our detailed responses are provided below.

Comment #1:

The manuscript attributes the FE decrease at higher electrolysis currents to "a drop in P_{O2} on the fuel side, which enhances the electrolyte's electronic conductivity and leads to electron leakage." This causal is currently under-explained. Please further elaborate this mechanism and support it with appropriate references.

Response: We thank the reviewer for pointing out that this mechanism requires further clarification. We agree that a more detailed explanation will improve the clarify of this discussion.

Under high electrolysis current densities, rapid oxygen removal at the fuel electrode/electrolyte interface generates a strongly reducing local environment, resulting in a significant decrease in the local oxygen partial pressure (pO_2). For proton-conducting perovskite electrolytes such as BZCYYb, a lower pO_2 thermodynamically favors the formation of electronic charge carriers (electrons) according to defect equilibria. Consequently, the electrolyte exhibits increased n-type electronic conductivity under strongly reducing conditions. When a sufficiently large electric field is applied during high-current electrolysis, these electrons can migrate through the electrolyte and form an internal electronic leakage current that does not participate in the intended water-splitting reaction.

As a result, although the total external current remains unchanged, only a portion of the current contributes to hydrogen production, while the remainder is carried by electronic leakage. This reduces the fraction of current associated with the electrochemical reaction, leading to a decrease in the measured Faradaic efficiency. Similar phenomena have been widely reported in protonic ceramic electrolysis cells operating under high current densities and strongly reducing environments.

Corresponding references have been added to support this explanation. The revised discussion has been incorporated into the manuscript and highlighted in red (Page 15, Lines 481–488).

"...As the applied electrolysis current density increases from 0.5 A cm^{-2} to 1.5 A cm^{-2}, the FE gradually decreased from 92% to 78%, which is attributed to a reduced oxygen partial pressure at the fuel electrode that enhances the electrolyte's electronic conductivity and induces minor electron leakage. The electrons generated within the electrolyte under these conditions can be driven by the applied electric field, forming an internal electronic leakage current that does not contribute to the intended electrolysis reaction. As a result, although the total external current remains unchanged, the fraction of current effectively utilized for hydrogen production decreases, leading to a reduction in the measured Faradaic efficiency[71, 72]. Conversely, the FE increased with rising steam partial pressure, consistent with the suppression of electronic defects and improved hydration under high-humidity conditions."

Comment #2:

Supplementary Fig. S15. The authors observe that, in electrolysis mode, the resistance decreases with increasing steam partial pressure. Please provide a detailed mechanistic explanation for this trend and discuss whether this resistance reduction could impact the Faradaic efficiency.

Response: We thank the reviewer for this insightful comment. We agree that the decrease in resistance with increasing steam partial pressure in electrolysis mode (Supplementary Fig. S19) requires a clearer mechanistic explanation.

Under electrolysis operation, increasing the steam partial pressure (P_{H_2O}) enhances the hydration degree of the proton-conducting electrolyte and electrode/electrolyte interfacial region. According to the hydration equilibrium of proton-conducting perovskites, higher humidity promotes the incorporation of protons through the reaction $H_2O + V_O^{\bullet\bullet} + O_O^x \rightarrow 2OH_O^{\bullet}$, thereby increasing the concentration of protonic defects (OH$^{\bullet}$). The resulting higher proton concentration improves proton transport in the electrolyte and lowers the overall ohmic resistance. In addition, higher steam availability facilitates surface reaction steps associated with water splitting and proton transfer, which can further reduce interfacial polarization resistance.

Regarding Faradaic efficiency, higher steam partial pressure also helps stabilize the

defect chemistry of the electrolyte under electrolysis conditions. Increased hydration shifts the equilibrium toward protonic defects and suppresses the formation of electronic carriers that may arise under strongly reducing environments. As a result, electronic leakage through the electrolyte is mitigated, which helps maintain a higher fraction of current contributing to hydrogen production. Therefore, the resistance reduction observed at higher P_{H_2O} is consistent with, and may partly contribute to, the improved Faradaic efficiency under high-humidity conditions rather than causing an efficiency loss.

The corresponding discussion has been added to the revised manuscript (**Page 14, Lines 453–458; Page 15, Lines 489–492.** Figure S15 is now Figure S19).

"...Supplementary Fig. 19 shows the ASR evolution of PBCFIn05 at 650 °C and 600 °C under different steam partial pressures (3%, 20%, and 30%), while Supplementary Fig. 20 compares R_p values for PBCF and PBCFIn10 at 550 °C and 30% H_2O (0.97 and 0.72 Ω·cm^2, respectively). The progressive decrease in ASR and Rp with increasing steam partial pressure reflects enhanced electrolyte hydration and increased protonic defect concentration, which facilitate proton transport and interfacial charge-transfer kinetics. In addition, excess indium incorporation further lowers R_p under humid conditions by strengthening metal–oxygen covalency and promoting more efficient proton-coupled oxygen electrochemistry at the heterointerface."

"...Conversely, the FE increased with rising steam partial pressure, consistent with the suppression of electronic defect formation and the enhanced hydration of the electrolyte and interfacial region under high-humidity conditions, which favor protonic transport and reduce electronic leakage[79, 84]."

Comment #3:

The manuscript assigns LF (<10 Hz) to gas diffusion, IF (10–2000 Hz) to surface oxygen exchange, and HF (>2000 Hz) to charge transfer without citation. Please provide appropriate references supporting these frequency-domain attributions for SOFC/PCFC electrodes

Response: We thank the reviewer for this helpful suggestion. We agree that the attribution of impedance processes in the LF/IF/HF frequency domains should be supported by appropriate literature references.

In the revised manuscript, we have added relevant references that establish the commonly accepted interpretation of impedance spectra for SOFC and PCFC oxygen electrodes. In general, the high-frequency (HF) response (>2000 Hz) is associated with charge-transfer processes at the electrode/electrolyte interface, the intermediate-frequency (IF) region (10–2000 Hz) corresponds to surface oxygen exchange and adsorption/desorption kinetics, and the low-frequency (LF) region (<10 Hz) is typically attributed to gas-phase diffusion and mass transport processes within porous electrodes. These assignments are consistent with previous impedance analyses of mixed-conducting oxide electrodes in solid oxide and protonic ceramic cells.

The corresponding references have been added to support these assignments in the revised manuscript and are highlighted in red (Page 14, Lines 440–444).

"...To further resolve the kinetic processes governing ORR, distribution of relaxation time (DRT) analysis was applied to EIS data at 600 °C. The DRT profiles were divided into three characteristic frequency regions: low frequency (LF, <10 Hz) associated with gas diffusion, intermediate frequency (IF, 10–2000 Hz) corresponding to surface oxygen exchange, and high frequency (HF, >2000 Hz) attributed to charge transfer. As shown in Fig. 5d, PBCF and PBCFIn10 exhibit larger LF and IF response areas, indicating sluggish oxygen diffusion and surface exchange kinetics[47, 48]. In contrast, PBCFIn05 shows significantly reduced relaxation intensities in both regions, suggesting more facile O_2 (g) → $2O_{ad}$ dissociation and faster O_{ad} → O_{ad}^{-} charge transport, which together accelerate overall ORR kinetics[46]." [Xu Y, et al. A low-Lewis-acid-strength cation Cs^+-Doped double perovskite for fast and durable oxygen reduction/evolutions on protonic ceramic cells. ACS Energy Letters ***8****, 4145-4155 (2023).][He F, et al. Conformal high-entropy oxide coatings enable fast*

and durable surface oxygen reactions. Joule 9, (2025)].[Hu T, et al. In situ engineering of a cobalt-free perovskite air electrode enabling efficient reversible oxygen reduction/evolution reactions. Advanced Functional Materials ***33****, 2305567 (2023).];*

Comment #4:

Cell fabrication details. "the cells were calcined at 1000 °C for 3h to achieve strong interfacial bonding between the cathode and electrolyte." Which atmosphere? Please specify the atmosphere and report the heating rates.

Response: We thank the reviewer for pointing out that the fabrication conditions were insufficiently specified. In the revised manuscript, we have clarified the calcination atmosphere and heating protocol used for cell fabrication. Specifically, the cathode–electrolyte assemblies were calcined in ambient air with a heating rate of 3.3 °C min^{-1} and held at 1000 °C for 3 h to ensure strong interfacial bonding.

In addition, considering the potential chemical interactions between the cathode and electrolyte during high-temperature firing, we performed post co-firing XRD analysis to verify their chemical compatibility. The results show no detectable secondary phases, confirming that the electrode and electrolyte remain chemically stable under the fabrication conditions (Supplementary Fig. 29).

The revised text has been added on page 19-20, lines 667 and 673.

"...The resulting green bodies were co-sintered in ambient air using a multistep heating program: the temperature was first increased to 200°C at a heating rate corresponding to a 60 min ramp and held at 200 °C for 60 min to remove organic residues, followed by heating to 1350 °C over 350 min and holding at 1350 °C for 5h. After natural cooling, dense electrolytes with porous anode backbones were obtained"

"...For cathode preparation, PBCF and In-doped PBCF powders were mixed with terpineol and 10 wt.% ethyl cellulose for 2 h in an agate mortar to form homogeneous pastes. The slurry was then screen-printed onto the sintered electrolyte surface

through a mask to define an active area of 0.196 cm^2. After drying at 80 °C, the cathode-coated cells were calcined in ambient air following a multistep heating program: the temperature was first ramped to 200 °C within 30 min and held for 60 min, then increased to 1000 °C over 330 min, followed by a dwell at 1000 °C for 3 h to achieve robust interfacial bonding between the cathode and electrolyte."

Comment #5:

In Figure 5l, the authors compared PBCFIn05 a single cell with single cells using other electrode materials. However, the temperature of 700°C is not clearly marked in the figure. Revising this figure would help avoid potential misunderstandings.

Response: We thank the reviewer for pointing out this ambiguity. We agree that the operating temperature should be explicitly indicated to avoid potential misunderstanding. In the revised manuscript, we have clearly marked the operating temperature (700 °C) in Fig. 5l and updated the figure caption, accordingly, indicating that all performance data used for benchmarking were obtained at this temperature. This revision ensures that the comparison among different electrode materials is presented under consistent operating conditions.

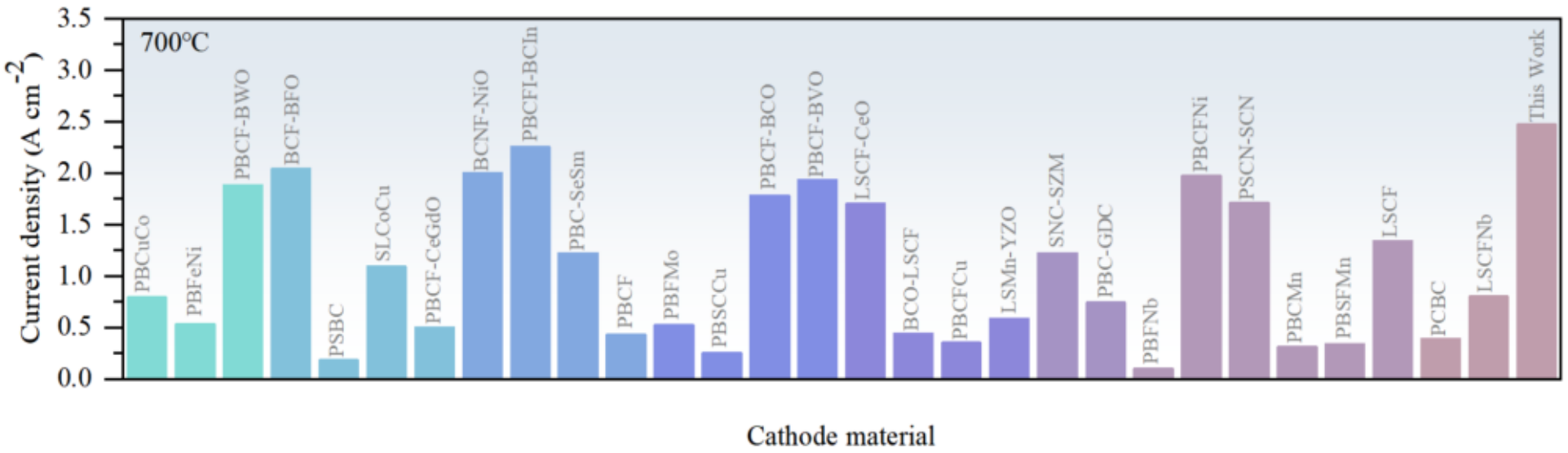

Fig. 5l Comparison of PDDs obtained with PBCFIn05 electrode and advanced electrodes in the literature at different temperatures.

Comment #6:

Fig. 5 only provides stability test data for PBCFIn05 after temperature cycling. To enhance the authors' arguments and more comprehensively demonstrate the material's properties, SEM images before and after temperature cycling should also

be included.

Response: We thank the reviewer for this constructive suggestion. We agree that providing microstructural characterization results before and after thermal cycling helps to better demonstrate the structural stability of the PBCFIn05 electrode.

Accordingly, SEM images of the air electrode surface and cross-sectional morphology before and after temperature cycling have been included in the revised Supporting Information. The post-cycling SEM results show no noticeable particle coarsening, cracking, or interfacial delamination compared with the pristine electrode, indicating that the electrode microstructure and the electrode–electrolyte interface remain largely preserved after thermal cycling. These observations provide direct morphological evidence supporting the excellent thermal durability of the PBCFIn05 electrode.

The corresponding figures and discussion have been added to Supplementary Fig. 27 and **page 15, lines 501.**

"…Thermal cycling durability was subsequently evaluated under three distinct heating-cooling protocols between 700 and 600 °C (Supplementary Fig. 26). In Mode 1, the cell experienced five moderate thermal cycles at a rate of 1.6 °C min^{-1} to simulate fatigue conditions. In Modes 2 and 3, more aggressive cycling profiles were applied (heating at 10 and 20 °C min^{-1}; cooling at 5 and 10 °C min^{-1}) for up to 65 cycles. Remarkably, the PBCFIn05 air electrode maintained nearly constant current output with negligible degradation across all thermal stress tests, confirming its exceptional thermomechanical resilience. The post-cycling SEM results show no noticeable particle coarsening, cracking, or interfacial delamination compared with the pristine electrode, indicating that the microstructure and electrode–electrolyte interface remain largely preserved after thermal cycling (Supplementary Fig. 27)…"

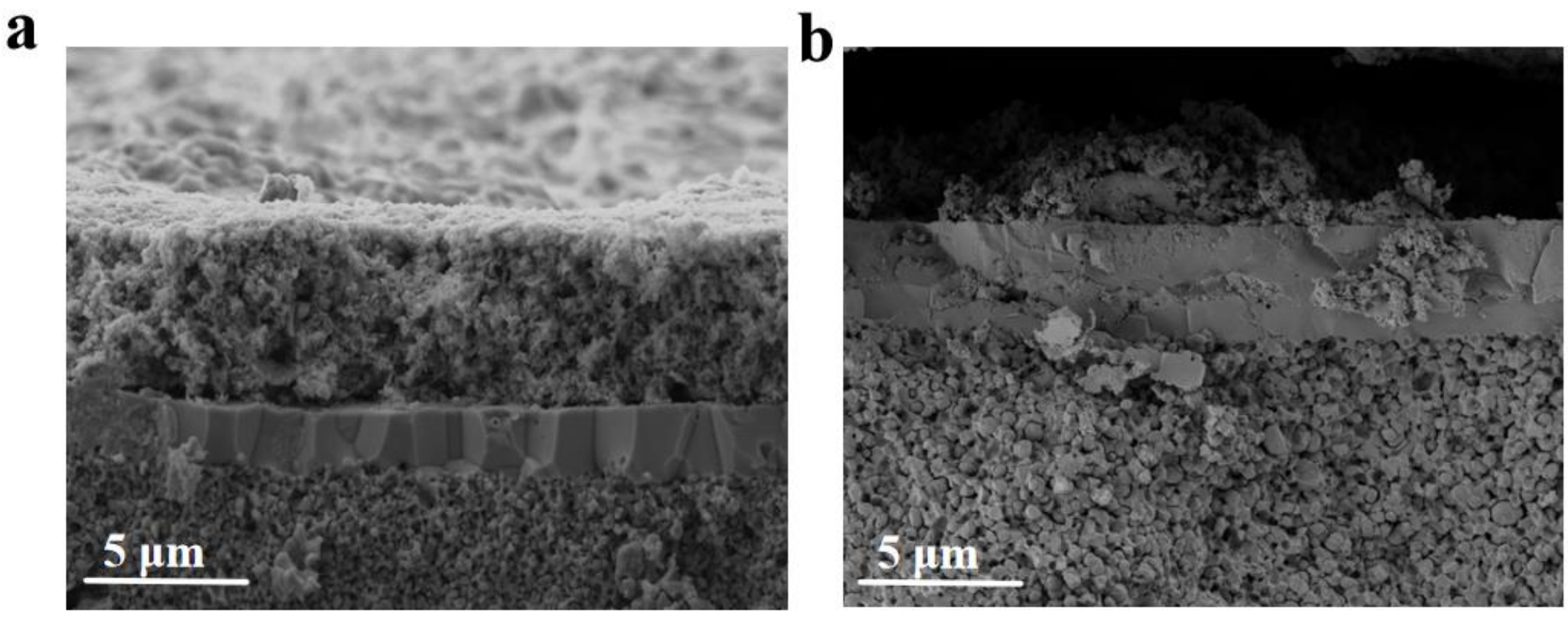

Supplementary Fig. 27 SEM image of PBCFIn05 single cell structure before and after the electrochemical tests at temperature cycling mode.

Comment #7:

The Methods section describes only single-cell fabrication, yet symmetric cells are used in the study. Please provide a detailed fabrication protocol for the symmetric cells and include SEM images of the symmetric electrodes. This will improve reproducibility and enable a clean comparison of electrode microstructure.

Response: We thank the reviewer for this helpful comment. We apologize for the confusion caused by the description in the original manuscript. In fact, symmetric cells were not used in this study. All electrochemical measurements, including impedance analyses, were performed using anode-supported single cells or half-cell configurations, as clarified in the revised Methods section.

To eliminate any ambiguity and improve reproducibility, we have carefully revised the manuscript to explicitly state the cell configuration used for each electrochemical measurement. In addition, SEM images of the air electrode microstructure in the single-cell configuration have been included in the Supporting Information to provide detailed structural information and facilitate comparison of electrode morphology.

The corresponding figures and discussion have been added to Supplementary Fig. 17 and **page 12, lines 415.**

"..As shown in Fig. 5a and Supplementary Fig. 17, the cross-sectional SEM image of PBCF, PBCFIn05 and PBCFIn10 based single cell clearly reveals a dense electrolyte and well-bonded porous electrode layers."

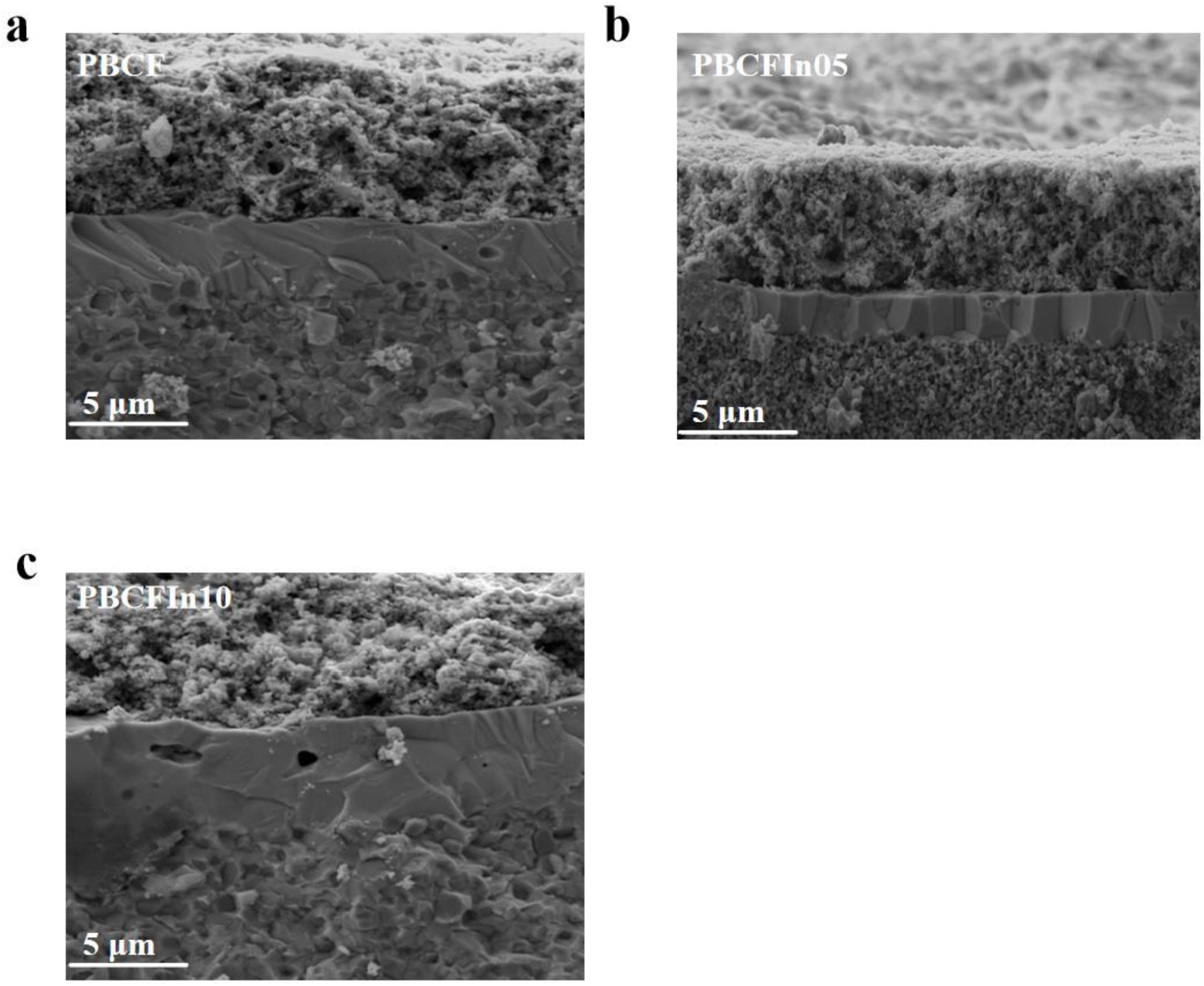

Supplementary Fig. 17 SEM image of PBCF, PBCFIn05 and PBCFIn10 single cell structure.

Reviewer # 2

Comments to the Author

This manuscript presents a method called spin-coordinated heterointerface engineering to improve oxygen electrocatalysis in protonic ceramic electrochemical cells. The authors attempt to link bulk charge transport with surface redox reactions by establishing a mixed-valence pathway. This is achieved by forming a Co3O4 spinel layer through the addition of indium to $Pr_{0.5}Ba_{0.5}Co_{0.7}Fe_{0.3}O_3$, improving both oxygen reduction and evolution performance. The following comments and suggestions are provided to help improve the scientific quality of the manuscript.

Response: We sincerely thank Reviewer #2 for the careful evaluation of our manuscript and for the constructive comments and suggestions. We appreciate the reviewer's summary of our work and the helpful feedback aimed at improving the scientific quality and clarity of the manuscript. We have carefully considered all the comments and revised the manuscript accordingly. Our detailed point-by-point responses are provided below.

Comment #1:

The experimental part of this study is comprehensive, utilizing a wide range of characterization techniques. However, several important issues reduce the reliability of the conclusions. First, the phase purity and reproducibility of the synthesized materials are not clearly demonstrated. Although XRD and Rietveld refinement confirm the coexistence of perovskite and Co_3O_4 phases, the quantitative composition, spatial uniformity, and structural continuity of the spinel layer are not verified. In addition, the proposed "stress-assisted cation segregation" mechanism for Co_3O_4 formation remains qualitative and is not supported by direct compositional or thermodynamic evidence. A more detailed discussion is recommended.

Response: We thank the reviewer for this important comment and agree that the phase composition, spatial distribution of the Co_3O_4 phase, and the mechanism of its formation should be clarified. The manuscript has been revised accordingly, and additional analyses and discussions have been included to address these concerns.

Below we respond your comment one by one:

(1) Phase purity and reproducibility.

To verify the reproducibility of the synthesized material, we performed two independent re-syntheses of PBCFIn05 following the same preparation protocol. The resulting XRD patterns show highly consistent diffraction features, and Rietveld refinements yield comparable phase fractions and lattice parameters within experimental uncertainty, demonstrating that the coexistence of the perovskite and Co_3O_4 phases is reproducible and intrinsic to the In-modified system. These results have been added to the revised manuscript (Supplementary Fig. 3 and Supplementary Tables 2–3).

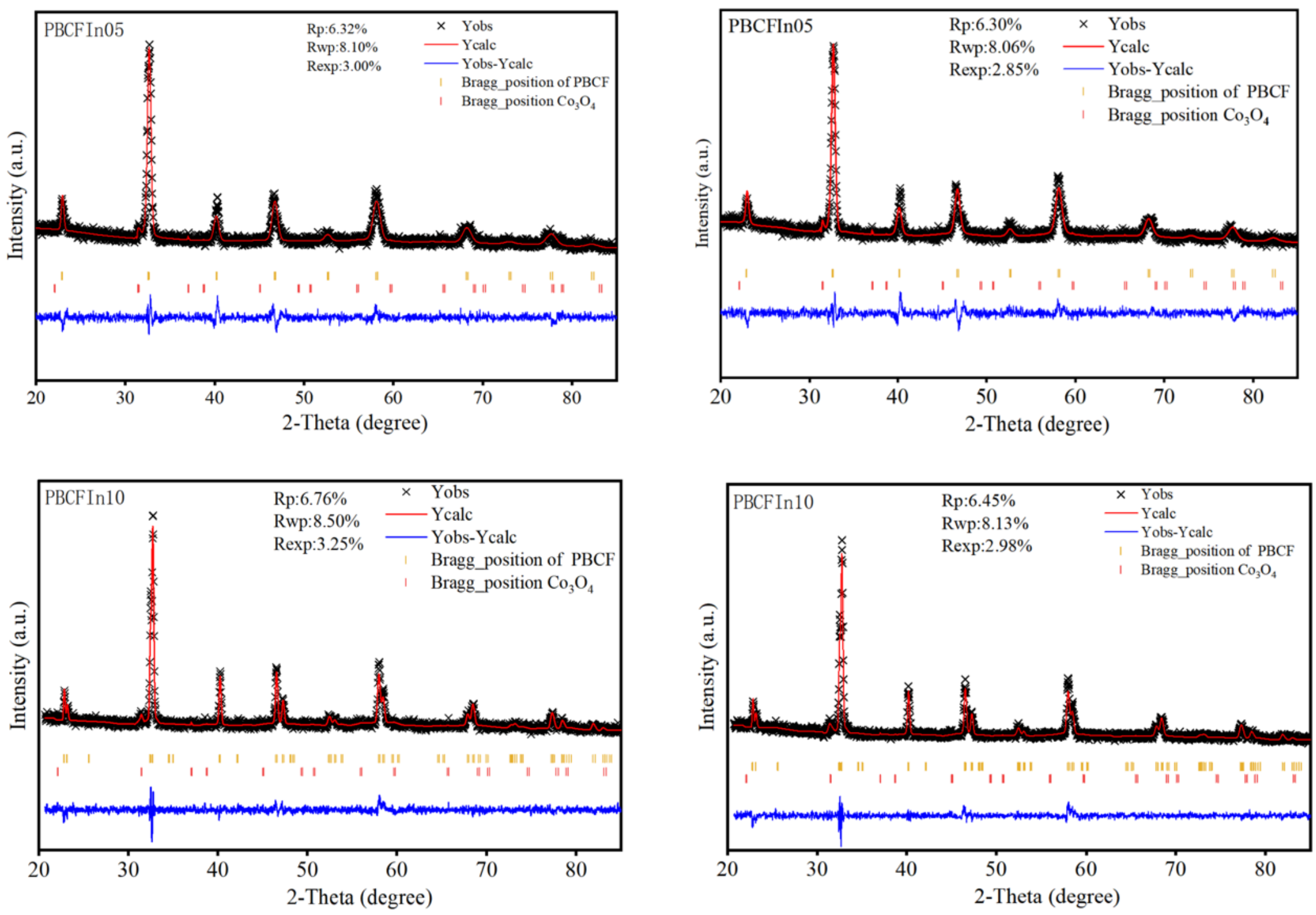

Supplementary Fig. 3 Rietveld refinement results of XRD patterns collected from independently synthesized batches of PBCFIn05 and PBCFIn10. Observed (Y_{obs}),

calculated (Y_{calc}), and difference (Y_{obs}-Y_{calc}) profiles are shown together with the Bragg positions of the perovskite PBCF and spinel Co_3O_4 phases, demonstrating the reproducibility of the refinement results.

Supplementary Table 2: Rietveld refinement results of PBCF, PBCFIn05, and PBCFIn10 obtained from the second independently synthesized batch.

**Sample**	**PBCFIn05**	**PBCFIn10**
Percentage and Space group	97.20% Pm-3m<br>2.80% Fd-3m	97.14% P4/mm<br>2.86% Fd-3m
a	3.88709	3.90500
b	3.88709	3.90500
c	3.88709	7.69565
α	90°	90°
γ	90°	90°
β	90°	90°
R_{wp}	8.10	8.50
R_p	6.32	6.76

R_{exp}	3.00	3.25

Supplementary Table 3: Rietveld refinement results of PBCF, PBCFIn05, and PBCFIn10 obtained from the third independently synthesized batch.

**Sample**	**PBCFIn05**	**PBCFIn10**
Percentage and Space group	97.15% Pm-3m<br>2.85% Fd-3m	97.17% P4/mm<br>2.83% Fd-3m
a	3.88651	3.90449
b	3.88651	3.90449
c	3.88651	7.69491
α	90°	90°
γ	90°	90°
β	90°	90°
R_{wp}	8.06	8.13
R_p	6.30	6.46

R_{exp}	2.85	2.98

(2) *Spatial distribution and structural continuity of the Co_3O_4 phase.*

We agree that the term "spinel layer" could imply a continuous coating, which may lead to ambiguity. Based on our structural characterization, Co_3O_4 does not form a continuous layer, but instead appears as surface-confined domains that generate localized perovskite–spinel heterointerfaces. To clarify this point, we performed HRTEM–EDS mapping at multiple representative regions of the sample. These analyses consistently reveal discrete Co-enriched domains with non-continuous distributions, indicating that the Co_3O_4 phase is dispersed as isolated surface domains rather than forming a uniform coating. The terminology throughout the manuscript has been revised accordingly to better reflect this structural feature.

The corresponding figures and discussion have been added to Supplementary Fig. 5 and **page 8, lines 258.**

"....Additional multi-regional STEM-EDS mapping analyses further confirm the reproducibility and spatial distribution of the surface Co_3O_4 domains (Supplementary Fig. 5). Collectively, these findings demonstrate that indium substitution drives lattice strain-mediated Co segregation and self-assembly of a surface spinel layer which reconstructs a coherent interface that underpins the enhanced electrochemical performances of the In-modified electrodes."

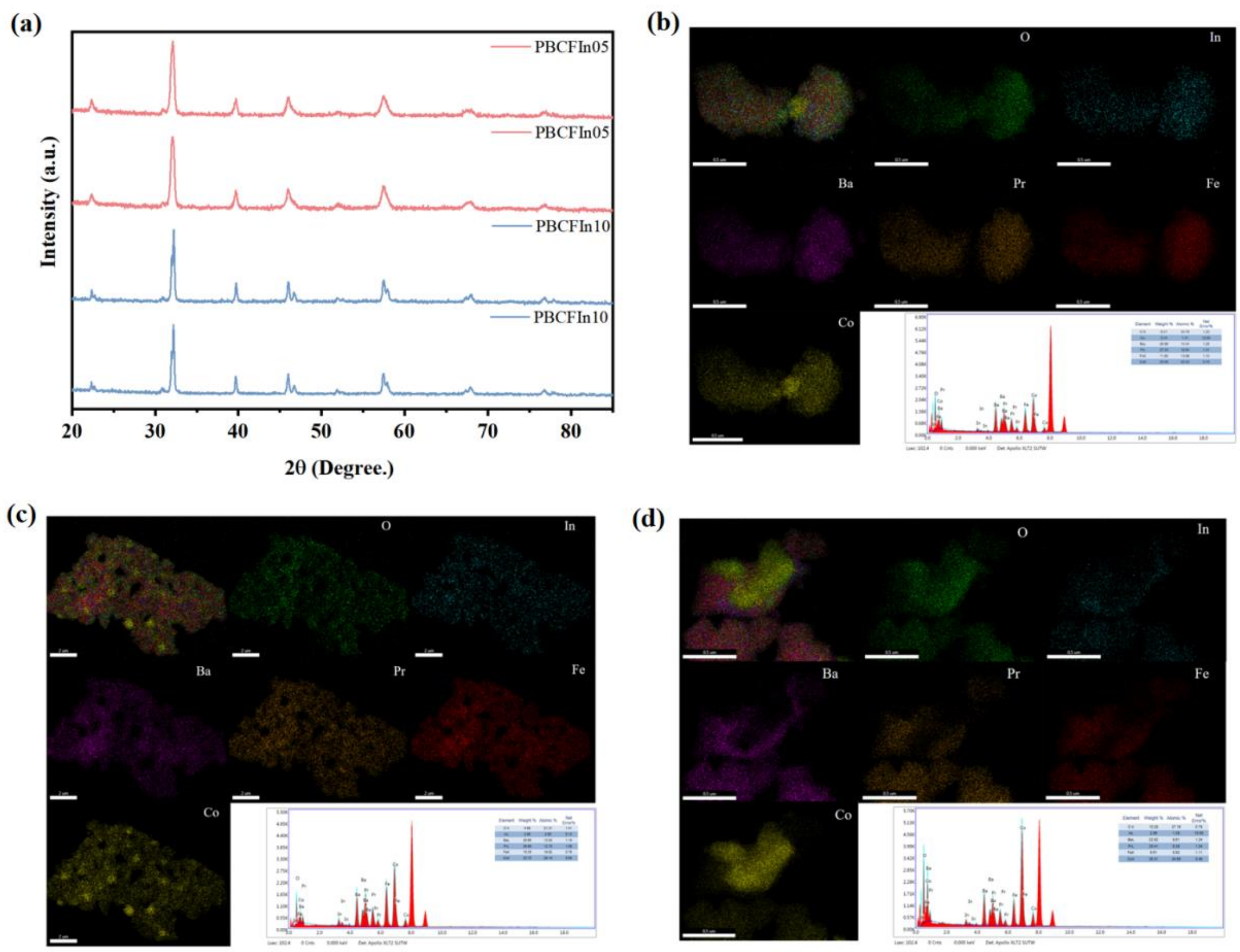

Supplementary Fig. 5 Reproducibility and spatial distribution of surface Co_3O_4 domains in PBCFIn samples. (a) XRD patterns of independently synthesized PBCFIn05 and PBCFIn10 samples, confirming the reproducibility of the phase composition and the emergence of a minor Co_3O_4 phase upon In doping, (b-d) STEM–EDS elemental mappings of PBCFIn05 collected from spatial regions. Co shows a non-continuous, localized surface distribution rather than a uniform layer.

(3) Mechanism of stress-assisted Co segregation.

We appreciate the reviewer's suggestion to further clarify the mechanism responsible for Co_3O_4 formation. From a thermodynamic perspective, the phase evolution associated with In incorporation can be expressed as:

$$Pr_{0.5}Ba_{0.5}Co_{0.7}Fe_{0.3}O_3+(x/2)In_2O_3\rightarrow(x/3)Co_3O_4+Pr_{0.5}Ba_{0.5}Co_{0.7-x}In_xFe_{0.3}O_3+(x/12)O_2$$

Because O_2 appears on the product side, lower oxygen partial pressure thermodynamically favors Co_3O_4 precipitation. Under high-temperature calcination conditions, In substitution introduces lattice distortion and defect redistribution, which

can lower the energetic barrier for Co migration and promote cation segregation toward the surface. Similar strain- or defect-driven cation segregation and surface spinel formation have been reported in doped perovskite oxides (Nat. Commun. 2022; Appl. Catal. B 2025; Adv. Funct. Mater. 2025).

Experimentally, the reproducible emergence of Co_3O_4 (XRD/Rietveld) together with STEM–EDS observations of Co-enriched surface domains supports the occurrence of such compositional redistribution. While we acknowledge that *in situ* thermodynamic measurements would provide more direct quantitative evidence, the combined structural characterization, reproducibility tests, and thermodynamic considerations support strain-assisted Co segregation as a plausible mechanism for the observed phase evolution. The relevant discussion has been expanded in the revised manuscript.

The corresponding discussion was added to page 6, Line221.

"...From a thermodynamic perspective, the phase evolution associated with In incorporation can be expressed as:

$$Pr_{0.5}Ba_{0.5}Co_{0.7}Fe_{0.3}O_3+(x/2)In_2O_3 \rightarrow (x/3)Co_3O_4+Pr_{0.5}Ba_{0.5}Co_{0.7-x}In_xFe_{0.3}O_{3+}(x/12)O_2$$

Because O_2 appears on the product side of the reaction, a lower oxygen partial pressure is expected to facilitate the formation of Co_3O_4. Under high-temperature calcination conditions, lattice strain induced by In substitution may further reduce the energetic stability of the homogeneous perovskite lattice, making partial cation redistribution thermodynamically accessible. To verify that the observed Co_3O_4 phase is intrinsic rather than an artifact of synthesis fluctuation, two independent re-syntheses of PBCFIn05 were performed. The resulting XRD patterns and Rietveld refinements show highly consistent phase fractions and lattice parameters within experimental uncertainty (Supplementary Fig. 3 and Table 2-3), confirming the reproducibility of spinel formation."

Comment #2:

Although the electrochemical results show good performance, important

methodological details are missing. Faradaic efficiency is discussed but not confirmed by quantitative gas analysis, which creates uncertainty about reaction selectivity. Also, the testing conditions and data reproducibility are not well documented. A more detailed explanation/discussion is needed to support the electrochemical findings and to clarify whether the observed improvements are due to the material's intrinsic catalytic activity or experimental variation.

Response: We thank the reviewer for this important comment regarding the methodological details and interpretation of the electrochemical results. We agree that a clearer description of the Faradaic efficiency (FE) determination, testing conditions, and data reproducibility is necessary to support the electrochemical conclusions. Accordingly, the manuscript has been revised to provide additional methodological details and reproducibility analyses.

(1) Faradaic efficiency determination and gas analysis.

In the revised manuscript, we have clarified the procedure used to determine the Faradaic efficiency for hydrogen production. The hydrogen generation rate was quantified by gas chromatography, and the FE was calculated by comparing the experimentally measured hydrogen production rate with the theoretical value derived from the applied current according to Faraday's law. These details have now been explicitly described in the revised Methods section and Supporting Information to ensure transparency in the FE evaluation.

(2) Electrochemical testing conditions.

To improve methodological transparency, we have expanded the description of the electrochemical testing conditions used for FE measurements. The revised manuscript now specifies the operating temperature, gas compositions supplied to both electrodes, total gas flow rates, applied current densities, and stabilization time prior to data acquisition.

These details are included in the revised Methods section and Supporting Information.

Faradaic efficiency measurement

"Faradaic efficiency (FE) was determined by comparing the electrochemically generated hydrogen rate with the theoretical hydrogen production rate at a given current density. The hydrogen evolution rate was quantified by gas chromatography (GC) under SOEC operation. During the measurements, a sweep gas consisting of 10% H_2/90% Ar at a total flow rate of 40 mL min^{-1} was supplied to the hydrogen electrode, while the PBCFIn05 electrode operated as the oxygen electrode and was fed with humidified air (40 mL min^{-1}). GC analysis was used to determine the outlet H_2 concentration, and the electrochemically produced hydrogen rate was calculated from the increase in H_2 concentration relative to the inlet sweep gas under steady-state conditions. The FE was then calculated by comparing this experimentally determined hydrogen production rate with the theoretical value derived from the applied current. Hydrogen output was monitored under both open-circuit voltage (OCV) and steady-current electrolysis conditions. Each data set was collected in triplicate, and the corresponding mean values and standard deviations were used to assess reproducibility.

The Faradaic efficiency (FE) was calculated using:

$$v=\frac{V_m}{2F}\times i$$

$$FE(\%)=\frac{v_e}{v}\times 100\%$$

where v is the theoretical hydrogen production flow rate (standard cubic centimeter per minute, sccm), i is the input current (A), F is the Faraday constant (9.6485×10^4 C mol^{-1}); and V_m is the molar volume of a gas (22,400 mL mol^{-1}). The experimental hydrogen production flow rate v_e."

(3) Reproducibility and origin of performance enhancement.

To verify data reproducibility, key electrochemical measurements were repeated under identical experimental conditions. In particular, the Faradaic efficiency for hydrogen production using the PBCFIn05 electrode in electrolysis mode was independently measured three times, and the corresponding mean values and standard deviations were calculated to quantify experimental uncertainty. The results show

consistent performance trends with only minor variations within experimental error. The reproducibility data have been added to Table S12 in the Supporting Information.

Based on these statistically consistent results and the complementary structural and spectroscopic analyses presented in the manuscript, we attribute the observed electrochemical improvements primarily to the intrinsic catalytic properties of the In-modified electrode rather than to experimental variation. The relevant descriptions have been revised accordingly in the manuscript.

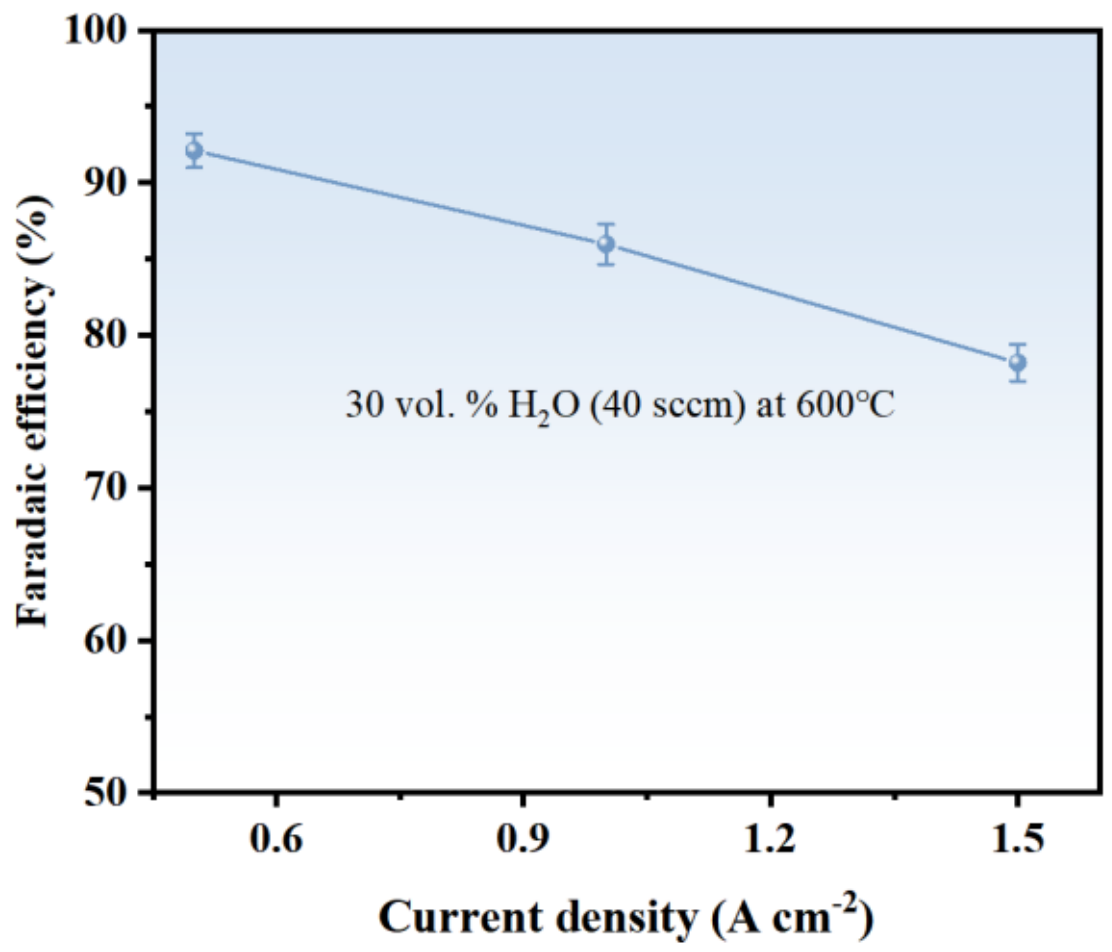

Fig. 5h. FE of cells at different current densities of PBCFIn05 in electrolysis mode at 600 °C.

Supplementary Table 12. Statistical standard deviations for FE. Each test was conducted for three times to get the statistical standard deviations, presented as error bars.

Current density (A cm^{-2})	GC-quantified H_2 (vol. %)	Quantified H_2 SD	FE (%)	FE SD
0.5	11.57	±0.02	92.1	±1.13
1.0	12.93	±0.04	85.9	±1.32
1.5	14	±0.06	78.2	±1.21

The corresponding discussion was added to page 15, Line 480.

“.... The FE of the PBCFIn05-based cell was evaluated using gas chromatography

under varying current densities and steam concentrations at 600 ° C (Fig. 5h–i and Supplementary Table 12). As the applied electrolysis current density increases from 0.5 A cm^{-2} to 1.5 A cm^{-2}, the FE gradually decreased from 92% to 78%, which is attributed to a reduced oxygen partial pressure at the fuel electrode that enhances the electrolyte's electronic conductivity and induces minor electron leakage. The electrons generated within the electrolyte under these conditions can be driven by the applied electric field, forming an internal electronic leakage current that does not contribute to the intended electrolysis reaction[81]. As a result, although the total external current remains unchanged, the fraction of current effectively utilized for hydrogen production decreases, leading to a reduction in the measured Faradaic efficiency[82, 83]. Conversely, the FE increased with rising steam partial pressure, consistent with the suppression of electronic defect formation and the enhanced hydration of the electrolyte and interfacial region under high-humidity conditions, which favor protonic transport and reduce electronic leakage[79, 84]. Detailed calculation formulas and procedures are provided in the Supporting Information.."

Comment #3:

Many recent computational studies on PBC- and Co, Fe-based perovskites have shown that accurate O 2p-band center values require either on-site correlation treatment (DFT+U) or hybrid exchange methods (HSE06). For example, DFT+U studies usually report O 2p-band centers in the range of -2.x eV, while HSE06 calculations show values in the range of -3.x eV relative to the Fermi level in recent reports. In contrast, the manuscript reports a much deeper value of -5.04 eV for pristine PBCF, which does not match these reference values and suggests the use of PBE without a U correction. Since the O 2p-band center is very sensitive to the exchange-correlation functional and the treatment of Co/Fe 3d electrons, the authors should: (i) clearly state which functional was used; (ii) explain why on-site correlation corrections were not applied; and (iii) discuss how this choice affects the accuracy of the band-center values. Ideally, recalculating the projected DOS using PBE+U or HSE06 would give more reliable values for comparison with the literature.

Doping only the In atom may not be able to alter the O 2p-band center from -5.04 eV to -2.74 eV. Please refer to Jacobs et al., Advanced Energy Materials 8, 1702708 (2018) and their other publications.

Response: We thank the reviewer for this important comment regarding the accuracy of the calculated O 2p-band center values and the choice of exchange–correlation functional. We agree that the electronic structure of transition-metal perovskites is sensitive to the treatment of on-site electron correlation for Co/Fe 3d states, and that methods such as DFT+U provide a more reliable description for comparison with literature values.

(1) Exchange–correlation functional used in the original calculations.

We clarify that the original calculations were performed using the Perdew–Burke–Ernzerhof (PBE) functional within the generalized gradient approximation (GGA) without on-site Coulomb corrections. This information has now been explicitly stated in the revised Computational Methods section to avoid ambiguity.

(2) Inclusion of on-site correlation corrections.

Following the reviewer's suggestion, we have recalculated the electronic structure using the DFT+U approach to better account for electron correlation effects in the transition-metal d states. The on-site Coulomb interaction parameters were chosen consistent with those commonly adopted in the Materials Project database, namely U = 5.3 eV for Fe and U = 3.32 eV for Co.

(3) Impact on O 2p-band center values.

The revised DFT+U calculations yield O 2p-band center values that fall within the range reported in recent literature, including the values reported by Jacobs et al. (Adv. Energy Mater., 2018, 8, 1702708). As expected, incorporating on-site Coulomb interactions increases the localization of the Co/Fe 3d states and shifts the O 2p-band center closer to the Fermi level compared with the PBE results. Importantly, while the absolute values of the band centers are corrected, the relative trend upon In incorporation remains consistent, supporting the conclusion that In modification

strengthens metal–oxygen covalency and enhances oxygen redox activity.

4) Recalculation of projected DOS for reliable comparison.

We have recalculated the projected density of states (PDOS) using the DFT+U method with U = 5.3 eV for Fe and U = 3.32 eV for Co. These revised calculations provide O 2p-band center values consistent with literature reports (−2.x to −3.x eV), in contrast to the deeper −5.04 eV obtained using plain PBE. Importantly, while the absolute values changed, the trend upon In incorporation remains, with the O 2p-band center shifting upward, confirming that In strengthens metal–oxygen covalency and enhances oxygen redox activity. The revised PDOS plots, numerical values, and related discussions have been updated in the revised manuscript.

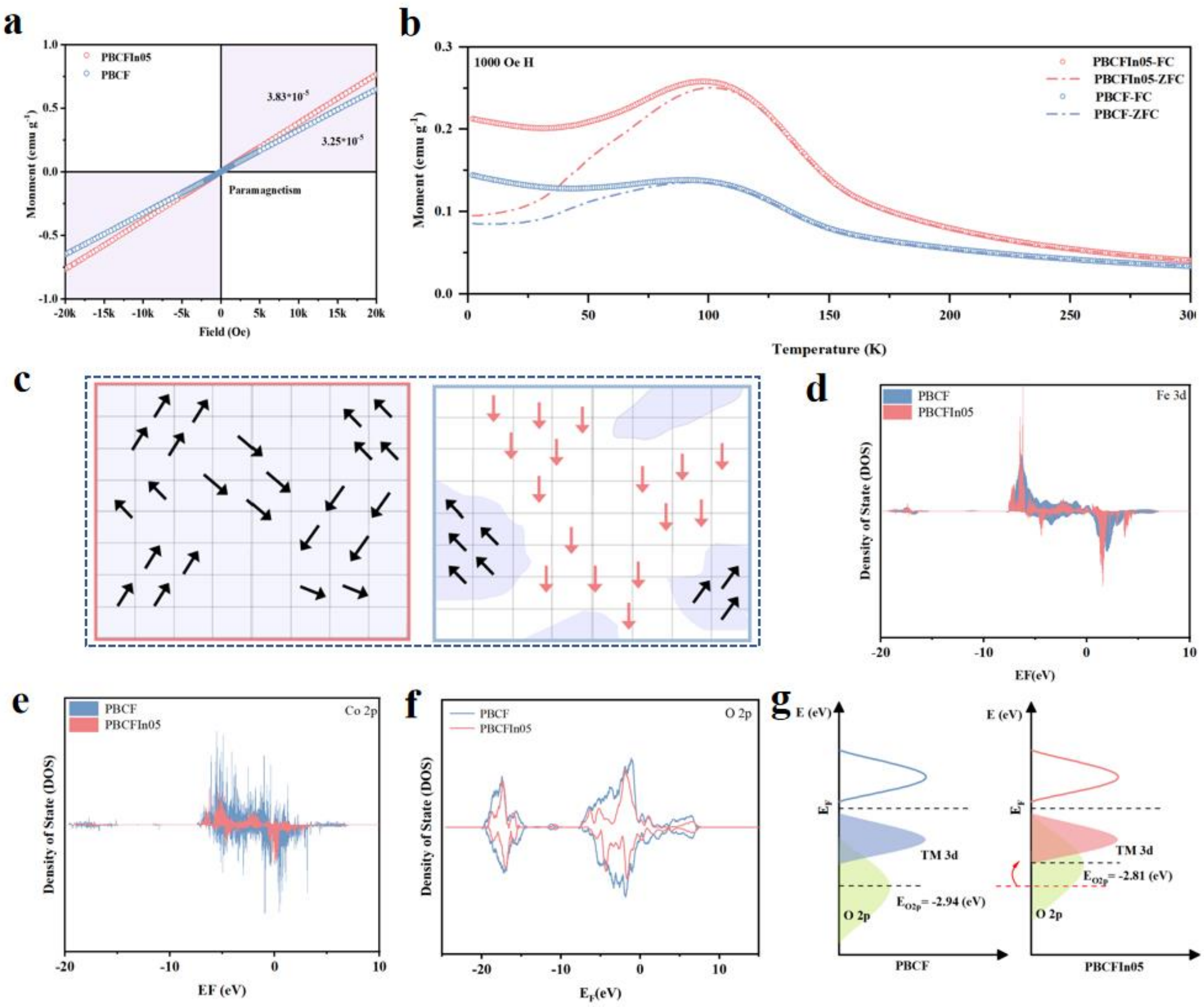

Fig. 1. Spin-related mixed-valence transport across bulk–interface pathways. **a** Magnetic hysteresis loops of PBCF and PBCFIn05. **b** Zero-field-cooled (ZFC) and

field-cooled (FC) magnetization curves of PBCF and PBCFIn05. **c** Schematic illustration of the competition between cluster spin-glass (CSG) and ferromagnetic (FM) phases. Black arrows represent randomly oriented spin clusters, while red arrows indicate collinearly aligned spins. The left panel shows the dominant CSG state in pristine PBCF, whereas the right panel depicts the coexistence of CSG and FM clusters in In-doped PBCFIn05. **d** Density of states (DOS) plots of Fe 3d orbital. **e** DOS of Co 3d orbitals. **f** schematic energy-level diagrams of O 2p. **g** DOS of O 2p states near the Fermi level.

The corresponding discussion was added to page 5, Line178.

"...Density-functional-theory (DFT) calculations (Fig. 1f–g) reveal that In3+ substitution shifts the O–2p band center upward from −2.53 eV to −2.39 eV, narrowing the energy gap between O–2p and transition-metal 3d orbitals. Notably, the obtained O–2p band-center values fall within the range reported in recent DFT+U studies on Co/Fe-based perovskite oxides, indicating consistency with the literature30. This upshift enhances M–3d–O–2p hybridization, signifying stronger Co/Fe–O covalency and improved lattice-oxygen mobility."

Comment #4:

It should be noted that the Computational Hydrogen Electrode (CHE) framework, while widely used in aqueous electrocatalysis, has been increasingly and incorrectly used in high-temperature solid oxide and protonic ceramic fuel cells. Several recent papers, including some by well-known groups, have presented CHE-based OER/ORR free-energy diagrams for perovskite oxides working at high temperatures. However, this approach does not align with the actual reaction mechanisms in such systems, as CHE assumes proton-electron transfer in a liquid environment, rather than oxide-ion movement or lattice oxygen redox in the solid state. The current manuscript appears to follow this incorrect approach by presenting a CHE-based thermodynamic diagram. The authors should carefully reconsider this modeling method and, if

possible, use a solid-state thermodynamic framework supported by mechanistic studies such as transition-state calculations. It is suggested to refer to the original papers reported by Norskov et al. This point also relates to the comment below about CI-NEB (see Comment 5).

Response: We thank the reviewer for this insightful and technically important comment. We fully agree that the Computational Hydrogen Electrode (CHE) framework, originally developed for aqueous electrocatalysis, does not accurately capture the reaction environment in high-temperature solid oxide or protonic ceramic electrochemical systems. In the original manuscript, the CHE-based reaction-energy diagram was intended primarily as a simplified thermodynamic descriptorto compare the relative stability of oxygen-related surface intermediates on PBCF and PBCFIn05. We acknowledge that such thermodynamic representations cannot fully capture the elementary reaction pathways in solid-state electrochemical systems.

Importantly, the main conclusions of this work are based on experimental observations, while the computational analyses serve to provide qualitative mechanistic insights supporting these trends. Nevertheless, in response to the reviewer's suggestion, we have re-examined and strengthened the theoretical methodology:

1. CI-NEB Transition-State Calculations: We performed climbing-image nudged elastic band (CI-NEB) calculations for the key elementary surface reactions to obtain physically meaningful activation barriers along the reaction pathways in the solid state. These calculations explicitly account for the energetics of lattice-oxygen participation and oxide-ion transfer, which better reflect the real reaction environment in protonic ceramic electrodes.

2. Results: The CI-NEB analysis shows that the activation barrier for the $^{*}H_2O \rightarrow {}^{*}OH + H$ step decreases from 1.95 eV on pristine PBCF to 1.60 eV on PBCFIn05, while the subsequent $^{*}OH \rightarrow {}^{*}O + H$ step is reduced from 0.71 eV to 0.22 eV after In incorporation. These results demonstrate that In substitution significantly lowers the kinetic barriers of key oxygen reaction

steps, in excellent agreement with the observed enhancements in experimental oxygen reduction and evolution performance.

3. Revised Discussion and References: The computational discussion in the manuscript has been updated to clarify the solid-state reaction pathway analysis and to explicitly present the CI-NEB results. References to the theoretical framework of Nørskov and co-workers have also been added to contextualize the methodology and ensure correct attribution.

These improvements provide a more accurate, physically grounded description of oxygen electrocatalysis in PBCF-based electrodes, while maintaining the role of DFT calculations as mechanistic support rather than as the primary evidence.

The corresponding discussion was added to page 17, Line 573.

"...To gain mechanistic insight into the oxygen reaction energetics on PBCF and PBCFIn05 surfaces, first-principles calculations were performed on the most stable surface terminations (Supplementary Figs. 30-33). The reaction pathway was analyzed by evaluating both the relative energies of surface intermediates and the activation barriers of key elementary steps. A thermodynamic reaction-energy diagram was first constructed to compare the relative stability of oxygen-related surface intermediates ($^*OH + H$, $^*OH$, $^*O$) on the two surfaces.The oxygen evolution reaction proceeds through sequential steps involving H_2O adsorption, hydroxyl formation, and oxygen evolution, while the oxygen reduction reaction follows the reverse sequence. For pristine PBCF, the dissociation of adsorbed water ($^*H_2O \rightarrow {}^*OH + H$) exhibits a high activation barrier of 1.95 eV, indicating sluggish surface reaction kinetics. In contrast, the barrier decreases to 1.60 eV on PBCFIn05, suggesting that indium incorporation significantly facilitates the water dissociation process. Similarly, the subsequent $^*OH \rightarrow {}^*O+H$ step shows a barrier reduction from 0.71 eV on PBCF to 0.22 eV on PBCFIn05."

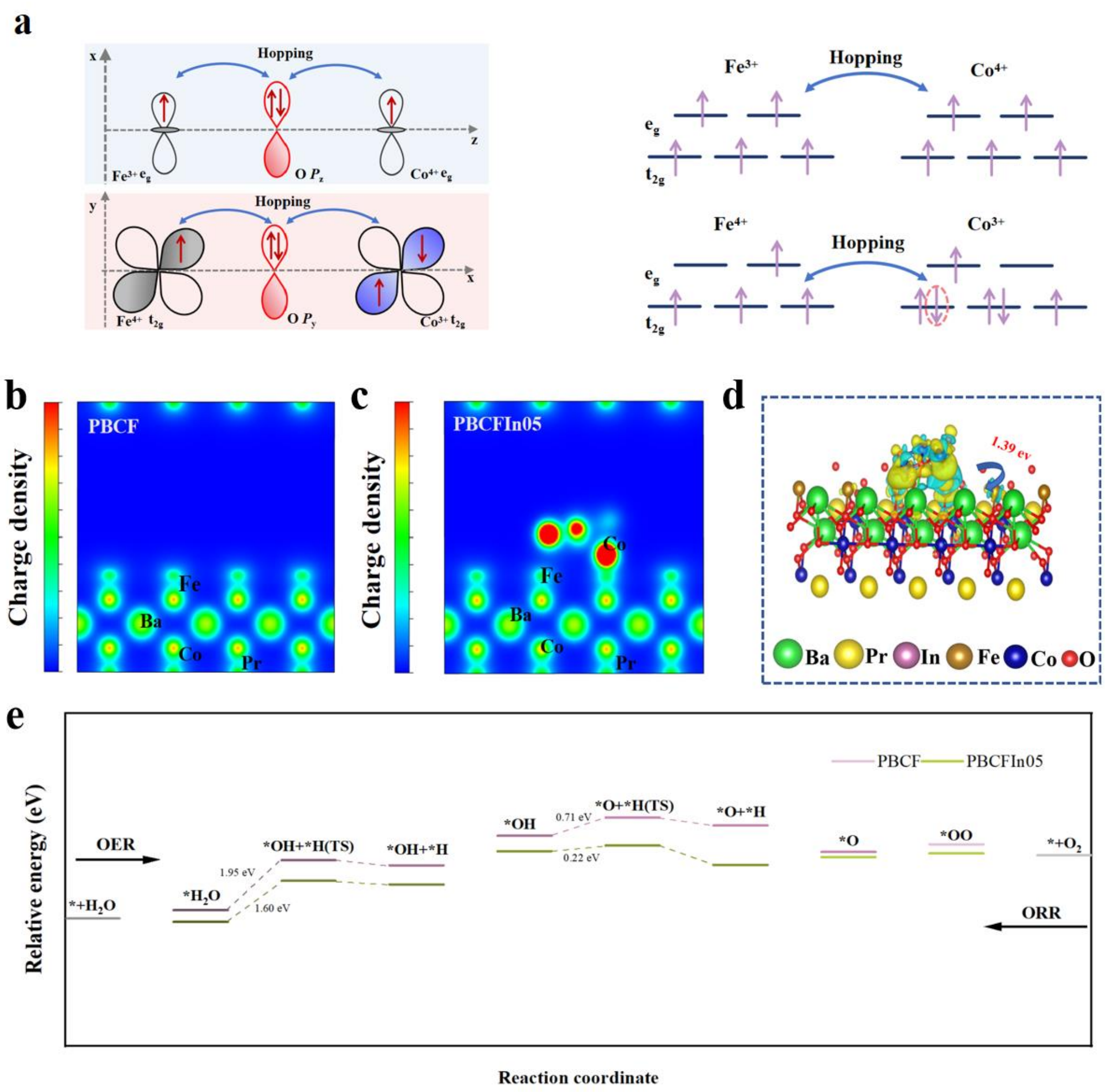

Fig. 6. DFT calculations regarding mechanisms on the air electrodes of R–PCECs. **a** Schematic diagram of electronic hopping pathways in PBCFIn05. Top: Fe^{3+}–O–Co^{4+} chain with e_g–p_σ–e_g overlap. Bottom: Fe^{4+}–O–Co^{3+} linkage with t_{2g}–p_π–t_{2g} overlap; **b-c** Differential charge density in PBCF and PBCFIn05; **d** Differential charge density of PBCFIn05 (side view); **e** Calculated free-energy profiles of the ORR and OER on PBCF and PBCFIn05.

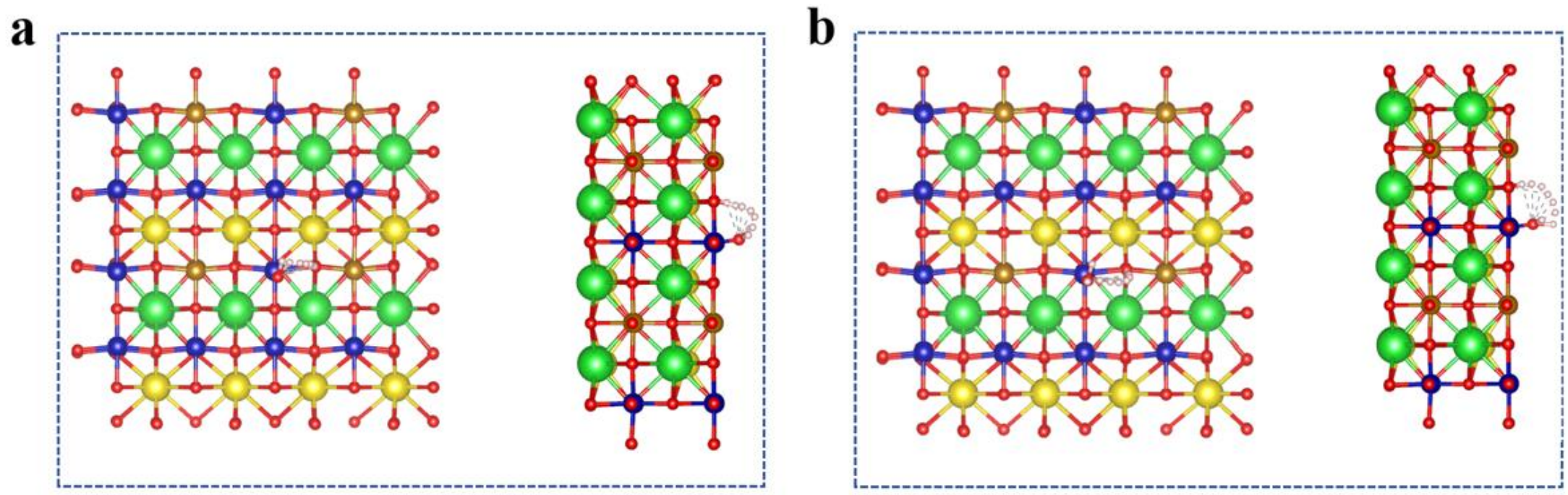

Supplementary Fig. 32. CI-NEB calculations of the **a)** $^*H_2O \rightarrow {}^*OH$ + H and **b)** $^*OH \rightarrow {}^*O$ + H dissociation pathway for PBCF.

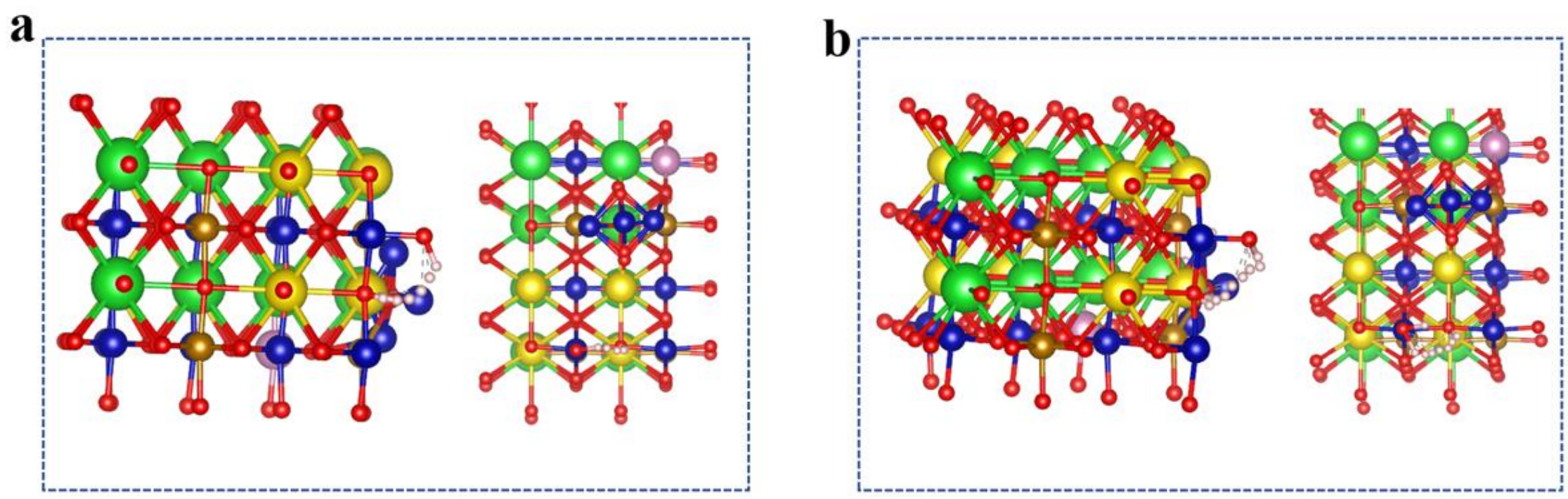

Supplementary Fig. 33. CI-NEB calculations of the **a)** $^*H_2O \rightarrow {}^*OH$ + H and **b)** $^*OH \rightarrow {}^*O$ + H dissociation pathway for PBCFIn05.

Comment #5:

*The authors state in the Computational Methods section that the CI-NEB method was used to calculate reaction energetics. However, the energy diagrams in the manuscript clearly follow the CHE model, showing free-energy changes for *OH, *O, and *OOH intermediates instead of transition-state barriers. These two approaches describe different physical quantities: CI-NEB provides activation energies along the reaction path, while CHE shows equilibrium thermodynamic data. If CI-NEB was actually used, the corresponding transition-state energies and migration barriers should be clearly reported.*

Response: We thank the reviewer for this important comment and for pointing out the distinction between thermodynamic reaction-energy diagrams and transition-state calculations obtained from the CI-NEB method. We fully agree that these two approaches describe different physical quantities: CI-NEB provides activation energies along the reaction pathway, whereas the CHE model reports equilibrium free-energy changes of surface intermediates.

In the original manuscript, the reaction-energy diagram was presented mainly as

a qualitative descriptor to illustrate the relative energetics of oxygen-related intermediates ($^*OH$, $^*O$, $^*OOH$) on PBCF and PBCFIn05 surfaces. We acknowledge that this may have caused confusion regarding the role of the CI-NEB calculations.

Following the reviewer's suggestion, we have revised the computational analysis to explicitly report the transition-state barriers obtained from CI-NEB calculations:

Water Dissociation Steps:

- $^*H_2O$ → $^*OH$ + H: activation barrier decreases from 1.95 eV (pristine PBCF) to 1.60 eV (PBCFIn05).
- $^*OH$ → $^*O$ + H: barrier decreases from 0.71 eV to 0.22 eV after In incorporation.

Proton Migration on the Surface:

- The calculated proton migration barrier decreases from 2.014 eV on PBCF to 1.011 eV on PBCFIn05, indicating that indium incorporation significantly facilitates surface proton transport.

These CI-NEB results demonstrate that In substitution effectively lowers the kinetic barriers of key oxygen reaction steps and proton transport on the PBCF surface, which directly supports the experimentally observed enhancement in ORR/OER kinetics.

The revised manuscript now clearly distinguishes thermodynamic descriptors from kinetic transition-state barriers, explicitly reports all CI-NEB-calculated activation energies, and updates the discussion of reaction pathways accordingly.

The corresponding discussion was added to page 17, Line 573.

"…To gain mechanistic insight into the oxygen reaction energetics on PBCF and PBCFIn05 surfaces, first-principles calculations were performed on the most stable surface terminations (Supplementary Figs. 22–23). The reaction pathway was analyzed by evaluating both the relative energies of surface intermediates and the activation barriers of key elementary steps. A thermodynamic reaction-energy

diagram was first constructed to compare the relative stability of oxygen-related surface intermediates ($^*OH$ + H, $^*OH$, $^*O$) on the two surfaces.The oxygen evolution reaction proceeds through sequential steps involving H_2O adsorption, hydroxyl formation, and oxygen evolution, while the oxygen reduction reaction follows the reverse sequence. For pristine PBCF, the dissociation of adsorbed water ($^*H_2O \rightarrow {}^*OH + H$) exhibits a high activation barrier of 1.95 eV, indicating sluggish surface reaction kinetics. In contrast, the barrier decreases to 1.60 eV on PBCFIn05, suggesting that indium incorporation significantly facilitates the water dissociation process. Similarly, the subsequent $^*OH \rightarrow {}^*O+H$ step shows a barrier reduction from 0.71 eV on PBCF to 0.22 eV on PBCFIn05. To further evaluate hydrogen-related surface transport processes, CI-NEB calculations were also performed to determine the proton migration barriers on the PBCF and PBCFIn05 surfaces (Supplementary Fig. 25-26). The calculated migration barrier decreases from 2.014 eV on pristine PBCF to 1.011 eV on PBCFIn05, indicating that indium incorporation significantly facilitates proton transport across the surface. This reduced migration barrier further contributes to the enhanced oxygen reaction kinetics observed experimentally."

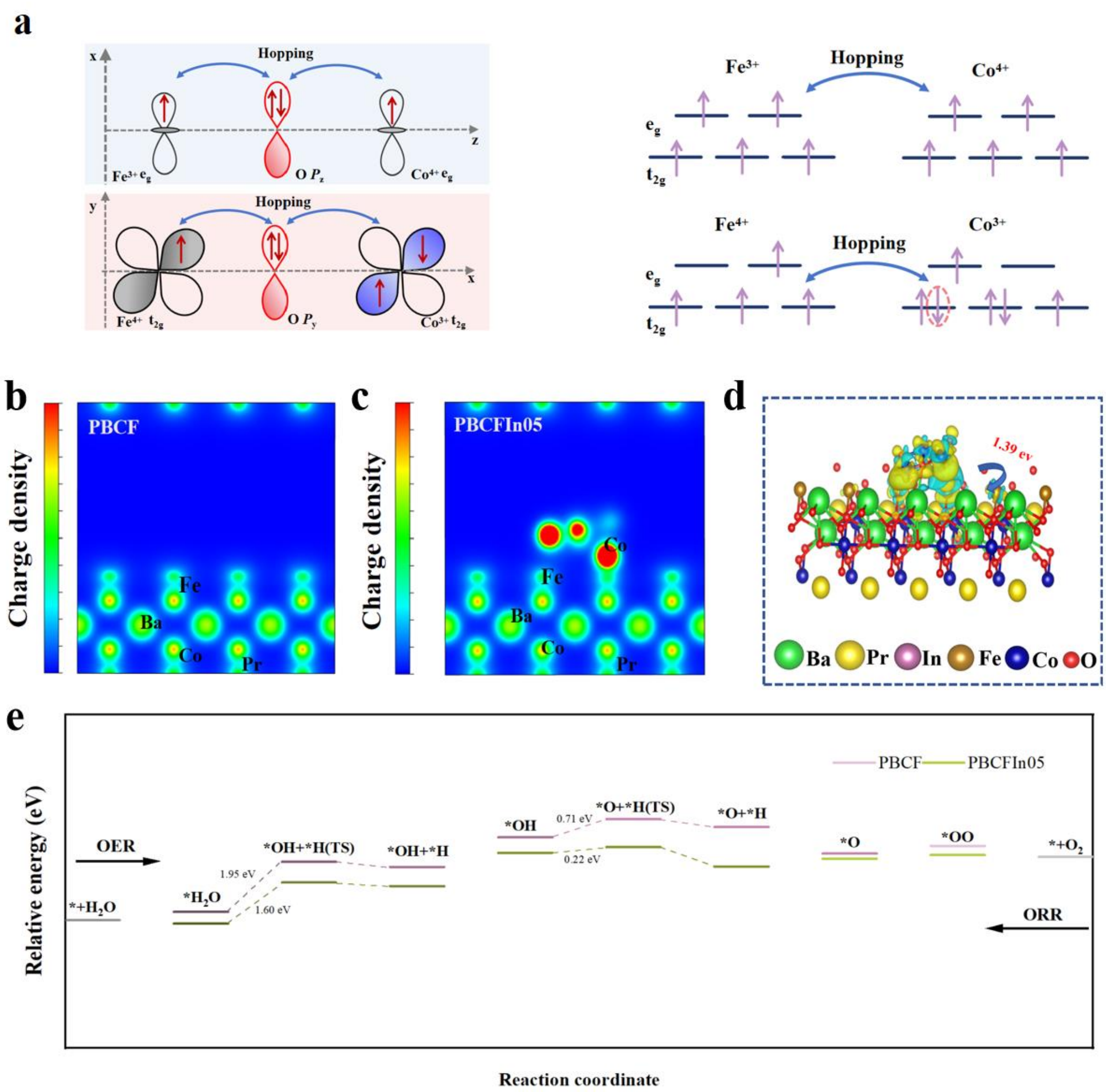

Fig. 6 DFT calculations regarding mechanisms on the air electrodes of R–PCECs. **a** Schematic diagram of electronic hopping pathways in PBCFIn05. Top: Fe^{3+}–O–Co^{4+} chain with e_g–p_σ–e_g overlap. Bottom: Fe^{4+}–O–Co^{3+} linkage with t_{2g}–p_π–t_{2g} overlap; **b-c** Differential charge density in PBCF and PBCFIn05; **d** Differential charge density of PBCFIn05 (side view); **e** Calculated free-energy profiles of the ORR and OER on PBCF and PBCFIn05.

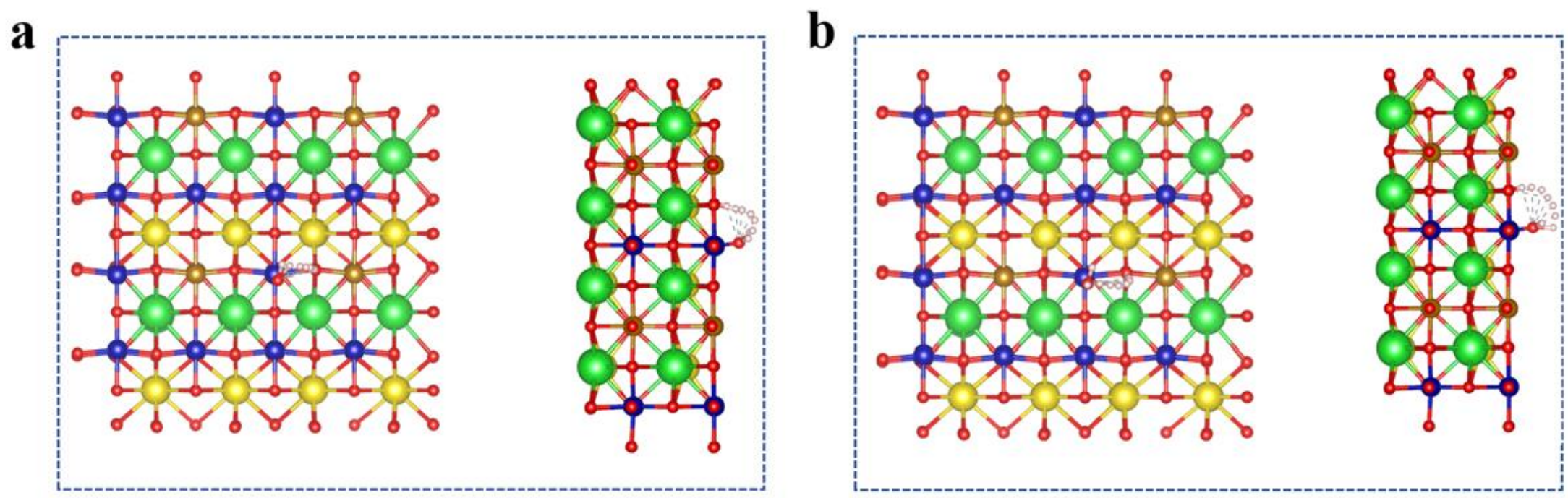

Supplementary Fig. 32. CI-NEB calculations of the **a)** $^*H_2O \rightarrow {}^*OH + H$ and **b)** $^*OH \rightarrow {}^*O + H$ dissociation pathway for PBCF.

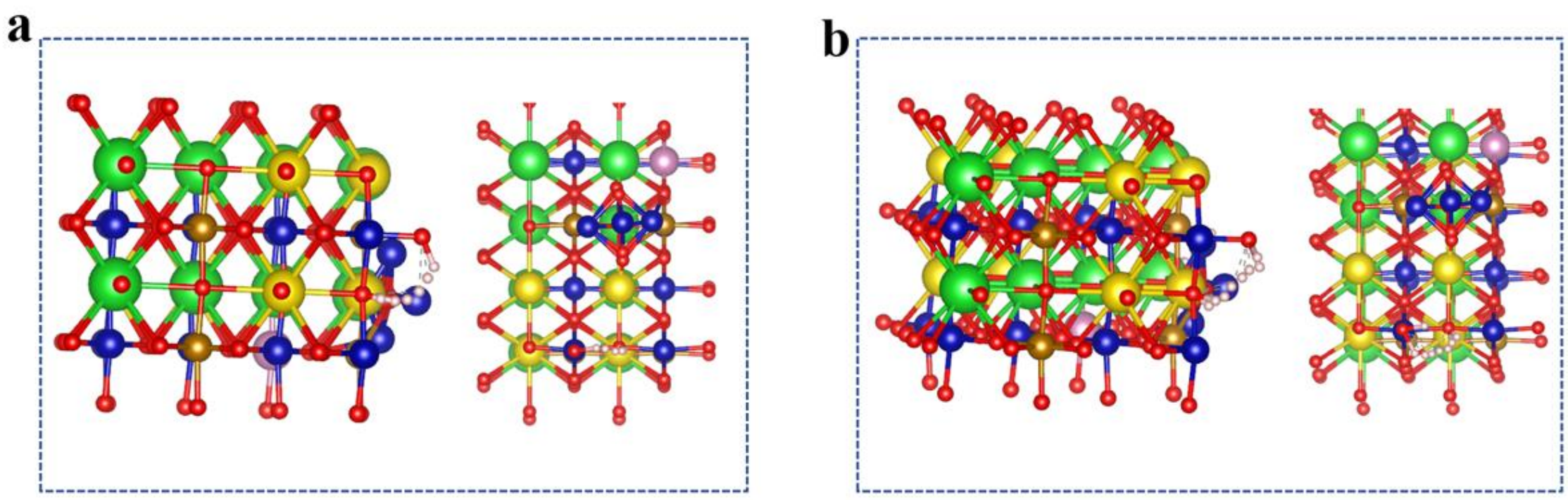

Supplementary Fig. 33. CI-NEB calculations of the **a)** $^*H_2O \rightarrow {}^*OH + H$ and **b)** $^*OH \rightarrow {}^*O + H$ dissociation pathway for PBCFIn05.

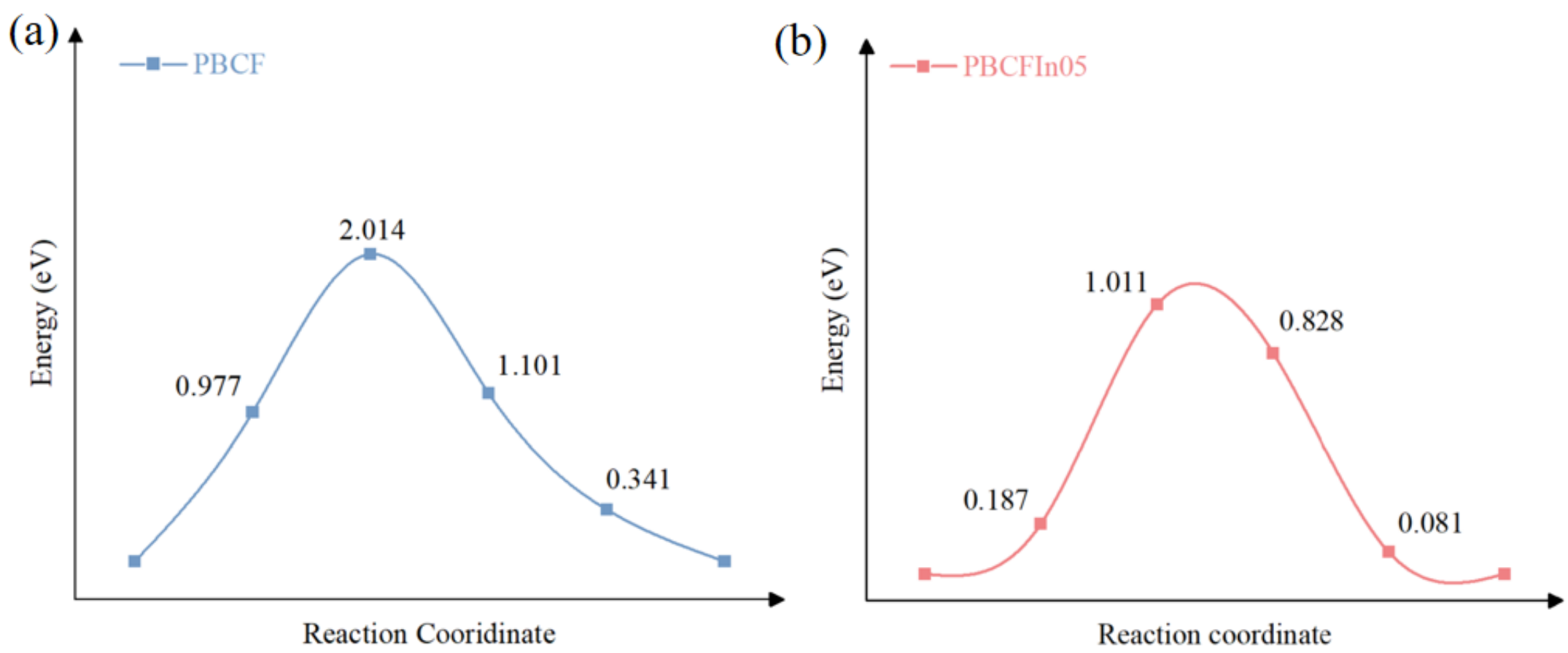

Supplementary Fig. 34. Calculated energy profiles for proton migration of **a)** PBCF and **b)** PBCFIn05.

Comment #6:

For the reference section, several important original references appear to have been omitted, and some cited computational methods were inaccurately interpreted or incorrectly applied. This suggests possible misuse or neglect of key literature, which reduces confidence in the validity of the computational results and may have

contributed to inconsistent or misleading correlations with the experimental findings.

Response: We thank the reviewer for this important comment. We acknowledge that the reference list and discussion of computational methods in the original manuscript were incomplete and, in some cases, insufficiently precise, which may have contributed to ambiguity in interpreting the computational results.

In response, we have carefully reviewed and updated the reference section to include key original studies relevant to electronic-structure calculations of Co/Fe-based perovskite oxides, particularly those employing DFT+U and hybrid functionals. All relevant references recommended by the reviewer have now been incorporated and explicitly cited in the revised manuscript. In addition, the description of our computational methods has been clarified to accurately reflect the exchange-correlation functionals, on-site Coulomb interaction parameters, and methodological assumptions used. Any previous oversimplification or misinterpretation of prior computational studies has been corrected, ensuring that the application of DFT+U and CI-NEB is consistent with established literature.

These revisions enhance the rigor, transparency, and reproducibility of the computational analysis, and strengthen the consistency between theoretical predictions and experimental observations, thereby increasing confidence in the validity of our findings.

Reviewer # 3

Comments to the Author

The manuscript presents an interesting concept that highly efficient oxygen electrode in protonic ceramic electrochemical cells requires not only enhanced surface reaction kinetics but also precise coordination of electronic and spin states across the bulk–interface continuum. The authors introduce spin-coordinated heterointerface engineering and report that In doping in PBCF (PBCFIn05) induces formation of a Co3O4 spinel phase accompanied by selective Co migration and interfacial reconstruction. This heterointerface is proposed to reduce the activation barriers for both the ORR and OER, thereby improving catalytic activity and enabling application in a R-PCEC platform. While the concept and the reported performance are promising, the current version of the manuscript does not yet provide the level of mechanistic clarity, experimental validation, and overall completeness required for publication in this journal. Some specific concerns are as follows:

Response: We sincerely thank Reviewer #3 for the thoughtful and constructive feedback. We greatly appreciate your recognition of the novelty and potential impact of our work on spin-coordinated heterointerface engineering in protonic ceramic electrochemical cells. In response to your comments, we have conducted additional experiments and analyses to strengthen the mechanistic evidence and clarify the role of In-induced Co_3O_4 formation, Co migration, and interfacial reconstruction. These revisions enhance the clarity, rigor, and completeness of the manuscript, and provide stronger support for the proposed correlation between bulk–interface spin/electronic states and the observed improvements in ORR/OER activity. Detailed responses to the specific concerns are provided below.

Comment #1:

The manuscript refers to indium-modified variants as $Pr_{0.5}Ba_{0.5}Co_{0.7}Fe_{0.3}O_{3-\delta}$-$In_{0.05}$ (PBCFIn05) and states that In^{3+} is introduced at the B-site to redistribute lattice valence and covalency while promoting the in situ formation of a self-assembled Co_3O_4 interfacial phase. However, the use of the term "indium-modified" is

ambiguous, as it is unclear whether indium is incorporated as an excess B-site dopant or as a substitutional dopant within the B-site lattice.

Response: We thank the reviewer for pointing out the ambiguity regarding the term "indium-modified." We agree that this description was insufficiently precise. In this work, indium is deliberately introduced as an excess B-site additive rather than as a substitutional dopant within the perovskite B-site lattice. This clarification has now been explicitly stated in the revised manuscript to avoid any potential confusion.

The revised text has been added on page 3, lines 99 and 113.

"...In this work, we demonstrate a practical realization of this concept through spin-coordinated heterointerface engineering in an $Pr_{0.5}Ba_{0.5}Co_{0.7}Fe_{0.3}O_{3-\delta}$ (PBCFIn0.5) with excess indium incorporation."

"...To probe the interplay between lattice valence distribution and magnetic exchange, we examined a compositional series of $Pr_{0.5}Ba_{0.5}Co_{0.7}Fe_{0.3}O_{3-\delta}$ (PBCF) and its excess indium incorporation variants, $Pr_{0.5}Ba_{0.5}Co_{0.7}Fe_{0.3}O_{3-\delta}$-In0.05 (PBCFIn05) and $Pr_{0.5}Ba_{0.5}Co_{0.7}Fe_{0.3}O_{3-\delta}$ –In0.1 (PBCFIn10)."

Comment #2:

Rietveld refinements of the XRD data (Fig. 2b, Supplementary Fig. S2, and Table 1) assign all compositions to a tetragonal perovskite structure. However, this assignment is not reasonable for a 3-delta system and likely reflects an incorrect structural model. Therefore, the conclusion that all materials are tetragonal perovskite appears to be simply wrong and should be re-examined.

Response: We thank the reviewer for pointing this out. We acknowledge that assigning all compositions to a tetragonal perovskite structure was inappropriate for this 3-delta system. In the revised manuscript, we have re-examined the structural description and corrected the corresponding statements.

The revised text has been marked in red in the revised manuscript, and the corresponding discussion has been added on page 6, lines 192.

"...Rietveld refinements of the XRD data (Fig. 2b and Table 1) reveals that PBCFIn05 consist primarily of a cubic perovskite PBCF phase (97.7 %, a=b=c=3.90 Å, space group Pm-3m) accompanied by a minor spinel Co_3O_4 phase (~2.7 %, a=b=c=8.10 Å,

space group Fd-3m). The low refinement residuals (R_{wp} = 4.56, R_p = 3.61, R_{exp} = 4.23; Table S1) confirm the reliability of the fits. The detailed refinement results for all compositions are summarized in Supplementary Fig. 2 and Table 1".

Supplementary Table 1: Summary of the Rietveld refinement for PBCF and PBCFIn05 and PBCFIn10

Sample	PBCF	PBCFIn05	PBCFIn10
Percentage and Space group	100% Pm-3m	97.7% Pm-3m<br>2.3% Fd-3m	97.13% P4/mm<br>2.87% Fd-3m
a	3.88360	3.90107	3.92592
b	3.88360	3.90107	3.92592
c	3.88360	3.90107	7.72156
α	90°	90°	90°
γ	90°	90°	90°
β	90°	90°	90°
R_{wp}	10.05	4.56	9.52
R_p	7.73	3.61	13.7

R_{exp}	5.66	4.23	11.35

Comment #3:

Although the authors analyze the electronic structure and surface valence-state distribution using XAS, XPS, and related techniques and correlate these results with ORR/OER catalytic activity, the developed air electrode is ultimately applied in R-PCECs However, the manuscript provides no experimental characterization relevant to protonic properties, such as hydration behavior, proton uptake, or proton conductivity of the air electrode.

Response: We thank the reviewer for this important comment. We acknowledge that the previous version of the manuscript did not provide direct experimental characterization of protonic properties of the air electrode. To address this, we have added hydration-related measurements and proton uptake experiments to evaluate the protonic behavior of the electrode. These additional results provide direct evidence of proton involvement in the air electrode and are now included in the revised manuscript, with corresponding discussion clarifying the relevance of protonic properties to R-PCEC operation.

The revised text has been added on page 12, lines 395.

"...Since these vacancies serve as active sites for water adsorption and dissociation, the H_2O-temperature-programmed desorption (H_2O-TPD) measurements were performed to probe hydration (Supplementary Fig. 15). Compared with PBCF, PBCFIn05 exhibits stronger H_2O desorption signals, with pronounced features in both the low-temperature region (50-200 °C) and medium-temperature (~400 °C) regions, indicating the presence of more weakly bound water and diverse binding sites. The protonic properties of the air electrode were further examined by quantifying the released water and proton uptake using Karl Fischer titration (Supplementary Fig. 16 and Table 9). Compared with pristine PBCF, the In-doped samples exhibit a markedly

enhanced water content associated with protonic species and proton uptake, with PBCFIn05 showing the highest released H_2O content (0.5845 mmol H_2O mol^{-1} oxide) and corresponding proton uptake (1.169 mmol H mol^{-1} oxide). These results suggest that In incorporation modulates the defect chemistry and surface–water interaction of the oxide, leading to an enhanced capability to accommodate water-derived protonic species. Such a change is expected to be beneficial for proton participation in the electrode reactions under R-PCEC operation."

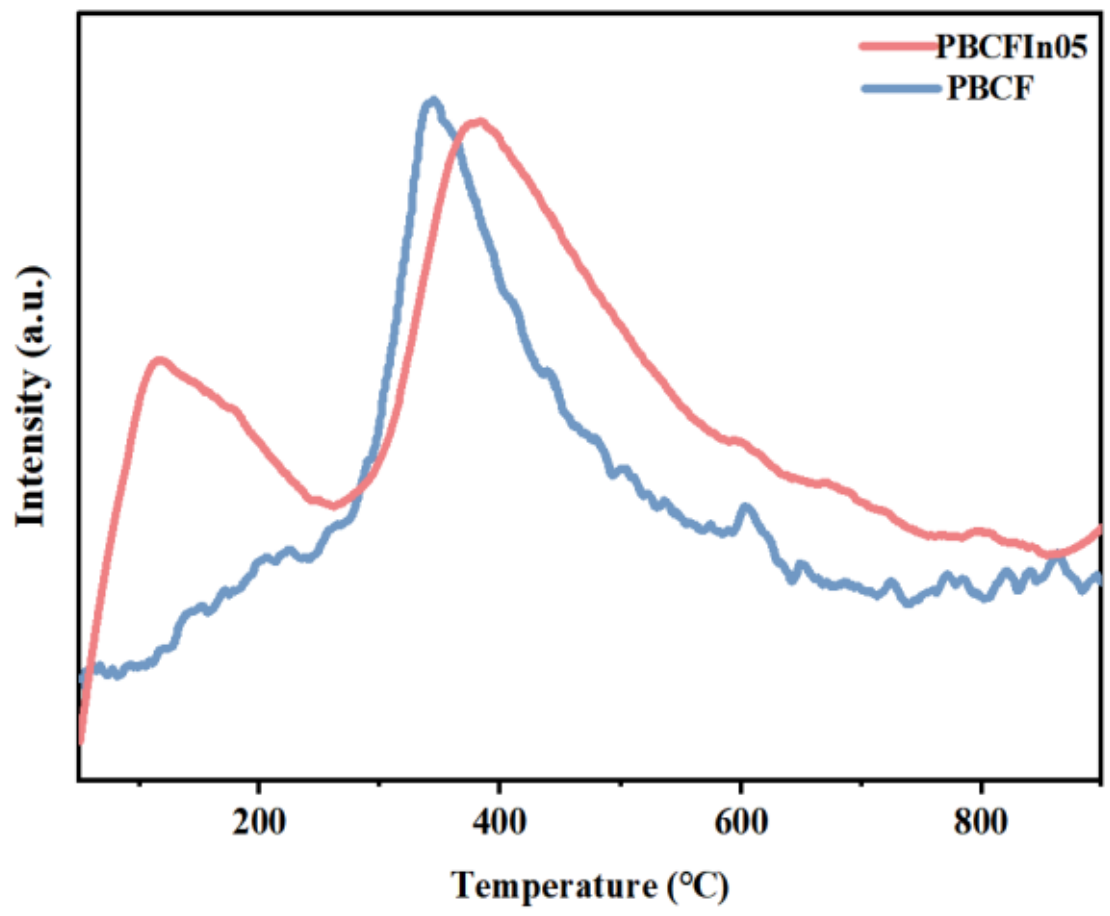

Supplementary Fig. 15 H_2O-TPD profiles from room temperature to 900 °C.

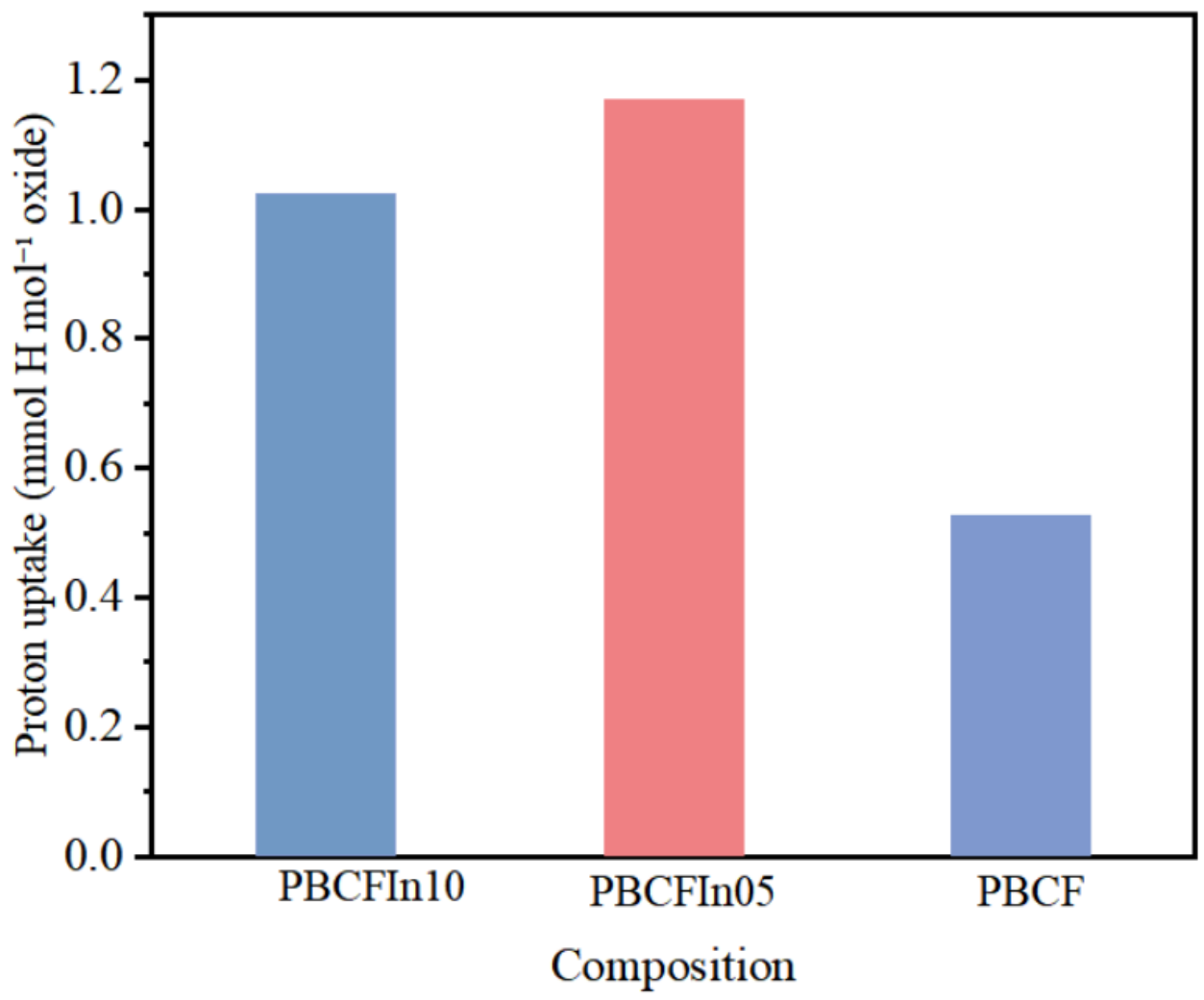

Supplementary Fig. 16 Proton uptake of PBCF，PBCFIn05 and PBCFIn10 samples measured by Karl Fischer titration.

Supplementary Table 9: Quantification of proton uptake by Karl Fischer titration

Sample	Mass (g)	Released H_2O (μg, KF)	Released H_2O (μg g^{-1})	Released H_2O (wt%)	H_2O uptake*(mmol H_2O /mol oxide)	Proton uptake * (mmol H/ mol oxide)
PBCFIn10	0.5524	20.3	36.75	0.0037	0.5117	1.0235
PBCFIn05	0.4838	18	41.04	0.0041	0.5845	1.1691
PBCF	0.5334	10.3	19.31	0.0019	0.2627	0.5255

Comment #4:

Regarding the electrochemical measurements, most of the measurements and analyses were conducted at temperature above 600 °C, which is fundamentally incorrect. At such temperatures, the electrolyte can no longer sustain predominantly protonic conduction. Therefore, to ensure relevance to R-PCEC operation, the electrochemical performance should be re-measured and re-analyzed at lower temperatures.

Response: We thank the reviewer for raising this important point. We acknowledge that at elevated temperatures, proton-conducting electrolytes may exhibit significant contributions from electronic and oxide-ion conduction, which can complicate interpretation of electrochemical results in the context of R-PCEC operation. To address this, we have performed additional electrochemical measurements in the lower temperature range of 450–550 °C, where protonic conduction dominates. Key performance metrics have been re-analyzed within this temperature window to ensure that the reported results accurately reflect proton-conduction–limited behavior. These new data have been incorporated into the revised manuscript and Supporting Information, and the corresponding discussion has been added on page 12, lines 417; page 14, lines 448; and page 14, lines 464.

"...The temperature-dependent EIS spectra (Fig. 5b and Supplementary Fig. 18) show that PBCFIn05 exhibits the lowest area-specific resistance (ASR), reaching 0.39

Ω·cm² at 550 °C, substantially lower than both the undoped and heavily doped counterparts. As summarized in Fig. 5c, PBCFIn05 consistently shows the lowest ASR and apparent activation energy across the 500-700°C tested range, underscoring its superior ORR catalytic performance."

"....In addition, the effect of water partial pressure on electrode behavior under electrolysis operation was studied, and EIS spectra were recorded at 550 °C with varying water pressure. As shown in Fig. 5e, the polarization resistance (Rp) decreases from 0.60 to 0.53 Ω·cm² as the water pressure increases from 3% to 30%, confirming that elevated humidity enhances proton transport and hydration at the electrode-electrolyte int erface[50, 51]. Supplementary Fig. 19 shows the ASR evolution of PBCFIn05 at 650 °C and 600 °C under different steam partial pressures (3%, 20%, and 30%), while Supplementary Fig. 20 compares R_p values for PBCF and PBCFIn10 at 550 °C and 30% H_2O (0.97 and 0.72 Ω·cm², respectively). The progressive decrease in ASR and R_p with increasing steam partial pressure reflects enhanced electrolyte hydration and increased protonic defect concentration, which facilitate proton transport and interfacial charge-transfer kinetics. In addition, excess indium incorporation further lowers R_p under humid conditions by strengthening metal–oxygen covalency and promoting more efficient proton-coupled oxygen electrochemistry at the heterointerface. These results demonstrate that moderate In doping (x = 0.05) achieves an optimal balance between electronic conductivity, ORR catalytic activity, and gas transport characteristics…"

"...The excellent catalytic properties of PBCFIn05 are further validated under both fuel cell (FC) and electrolysis cell (EC) modes. Fig. 5f presents the I-V and power density curves in FC mode. The PBCFIn05-based single cell delivers peak power densities (PPD) of 2.47, 1.95, 1.58, 1.21, and 0.79 W cm^{-2} at 700-500 °C respectively. For comparison, the undoped PBCF-based cell exhibits significantly lower PPD values of 1.34, 1.01, 0.62, 0.28 and 0.14 W cm^{-2} at 700-500 °C under identical testing conditions (Supplementary Fig. 21)."

"...Supplementary Fig. 22 presents the I-V curves under different steam partial

pressures (3% and 20%), while Supplementary Fig. 23 compares the corresponding results for PBCFIn10 and PBCF at 550 °C."

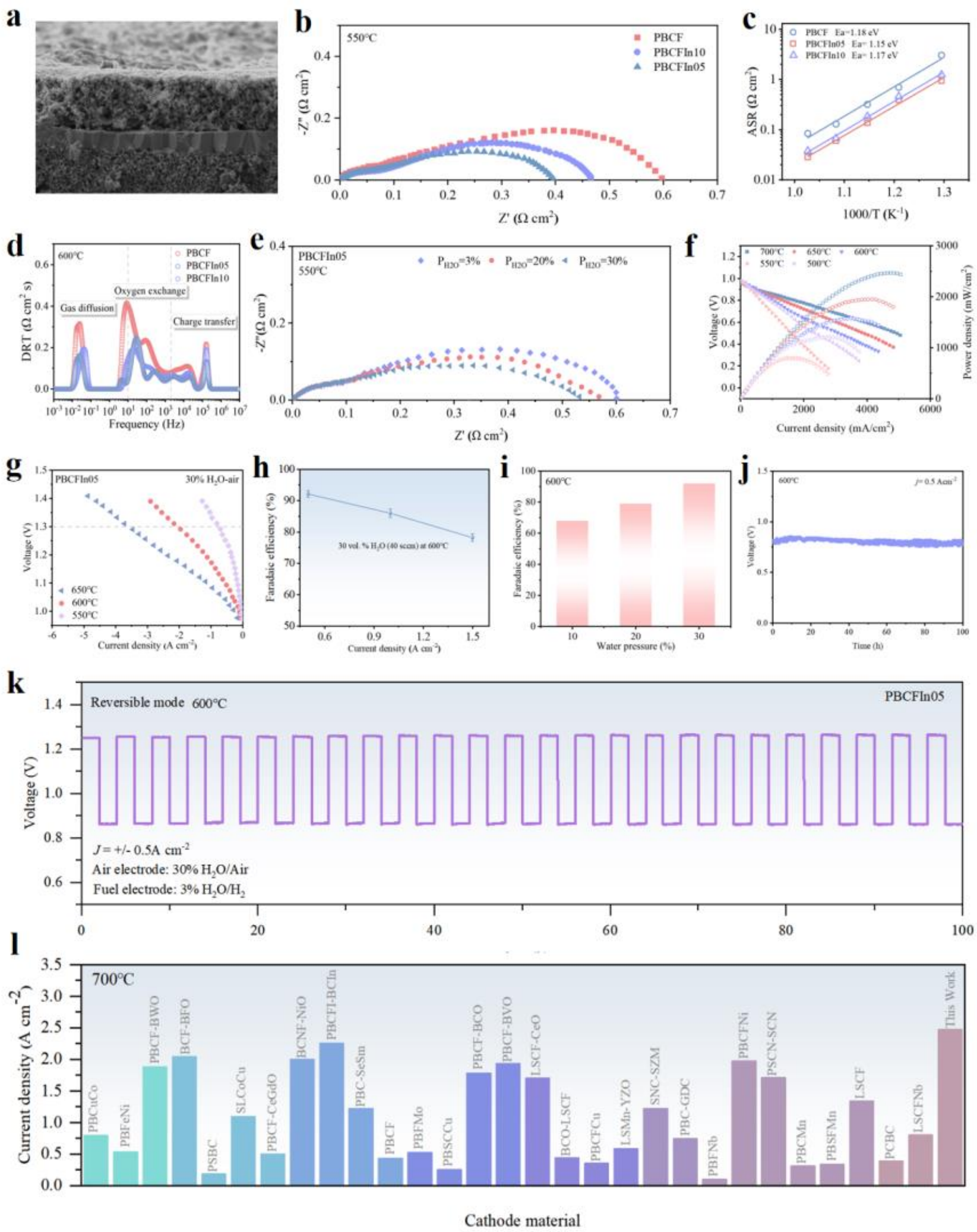

Fig. 5. Electrochemical performance and stability of single cells in fuel-cell (FC) mode. a Cross-sectional SEM image of PBCFIn05 single cell structure; **b** EIS plots of BZCYYb-supported single cell with PBCFIn05 electrode at 550 °C under 3%H_2O–H_2; **c** Arrhenius plot of the ASRs for PBCF, PBCFIn05 and PBCFI10; **d** The DRT analysis of EIS was measured at PBCF, PBCFIn05, and PBCFIn10 electrodes at 600 °C; **e** EIS plots of cell with PBCFIn05 air electrode tested at 550°C under 3, 10, 30%H_2O–air; **f** I–V and I–P curves of an BZCYYb-supported single cell with PBCFIn05 air electrode in FC mode at 500–700 °C; **g** I−V curves of cells with PBCFIn05 electrode measured

in EC mode at 650–550 °C under 30% H_2O–air; **h** FE of cells at different current densities; **i** FE of cells at different P_{H2O}; **j** A short durability assessment of a PCFCIn05 at a consistent current density of 0.5 A cm^{-2} and a temperature of 600 °C; **k** a stability test of PBCFIn05 in ±0.5 A cm^{-2} for 100 h at 600 °C; **l** Comparison of PDDs obtained with PBCFIn05 electrode and advanced electrodes in the literature at different temperatures.

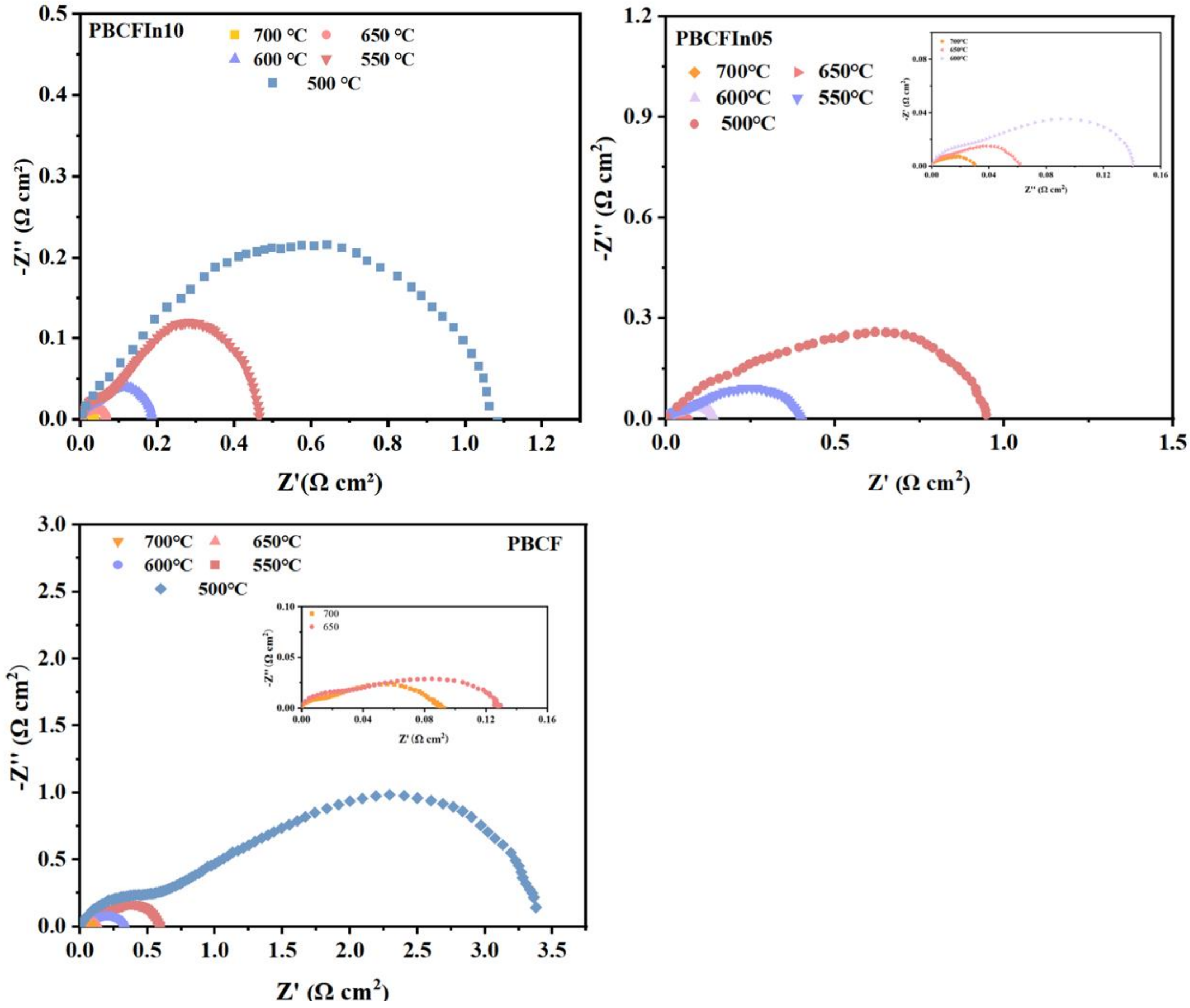

Supplementary Fig. 18. EIS plots of BZCYYb-supported single cells with PBCF, PBCFIn05 and PBCFIn10 at 500–700 °C under 3%H_2O–H_2. The ASRs of the single cell with PBCF air electrode are 3.3, 0.6, 0.32, 0.13, and 0.09 Ω·cm^2 at 500–700°C, respectively. Under the same test conditions, the ASRs of the single cell with PBCFIn10 air electrode are 1.1, 0.46, 0.19, 0.07, and 0.04 Ω·cm^2 at 500–700°C, respectively. The ASRs of the single cell with PBCFIn05 air electrode are 0.94, 0.39, 0.14, 0.06, and 0.03 Ω·cm^2 at 500–700 °C respectively.

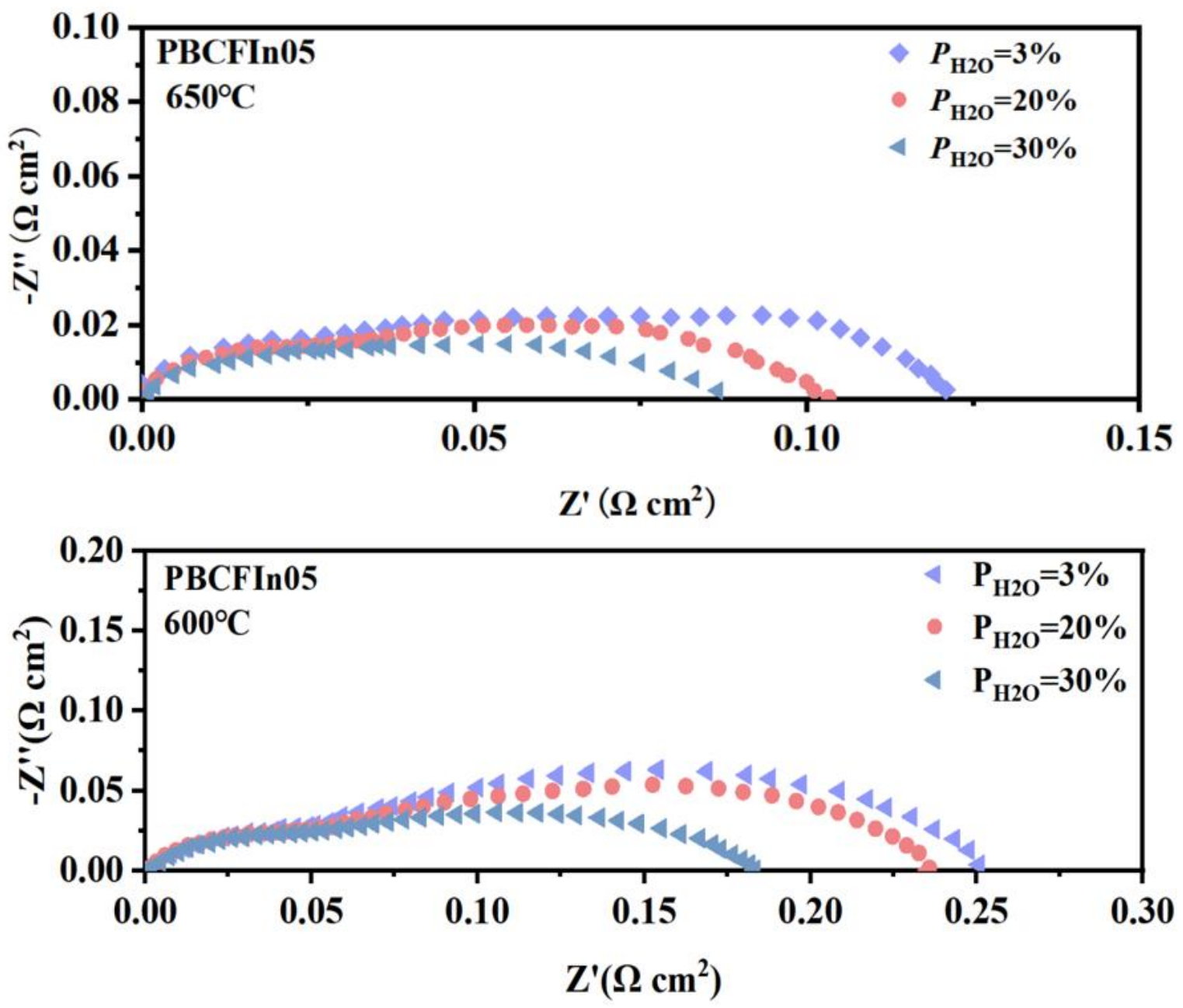

Supplementary Fig. 19. EIS plots of cell with PBCFIn05 air electrode tested at 600–650 °C under 3, 10, 30% H_2O-air. The ASRs of the single cell with PBCFIn05 at 600 °C are 0.18, 0.23,0.25 Ω·cm^2 respectively. The ASRs of the single cell with PBCFIn05 at 650 °C are 0.08, 0.1, 0.12 Ω·cm^2 respectively.

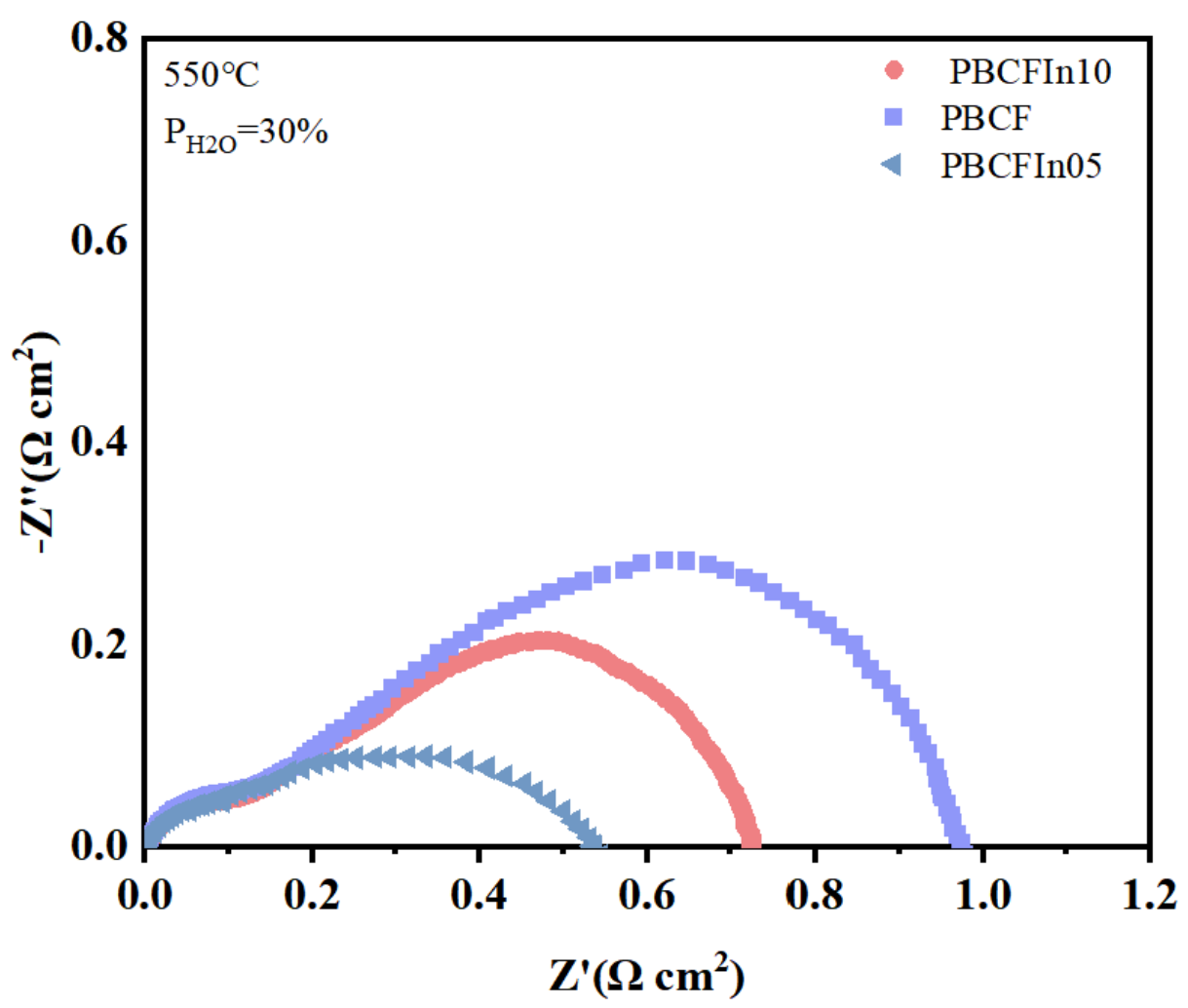

Supplementary Fig. 20. EIS plots of single cell with PBCF PBCFIn05 and PBCFIn10 at 550 °C under 30% H_2O.

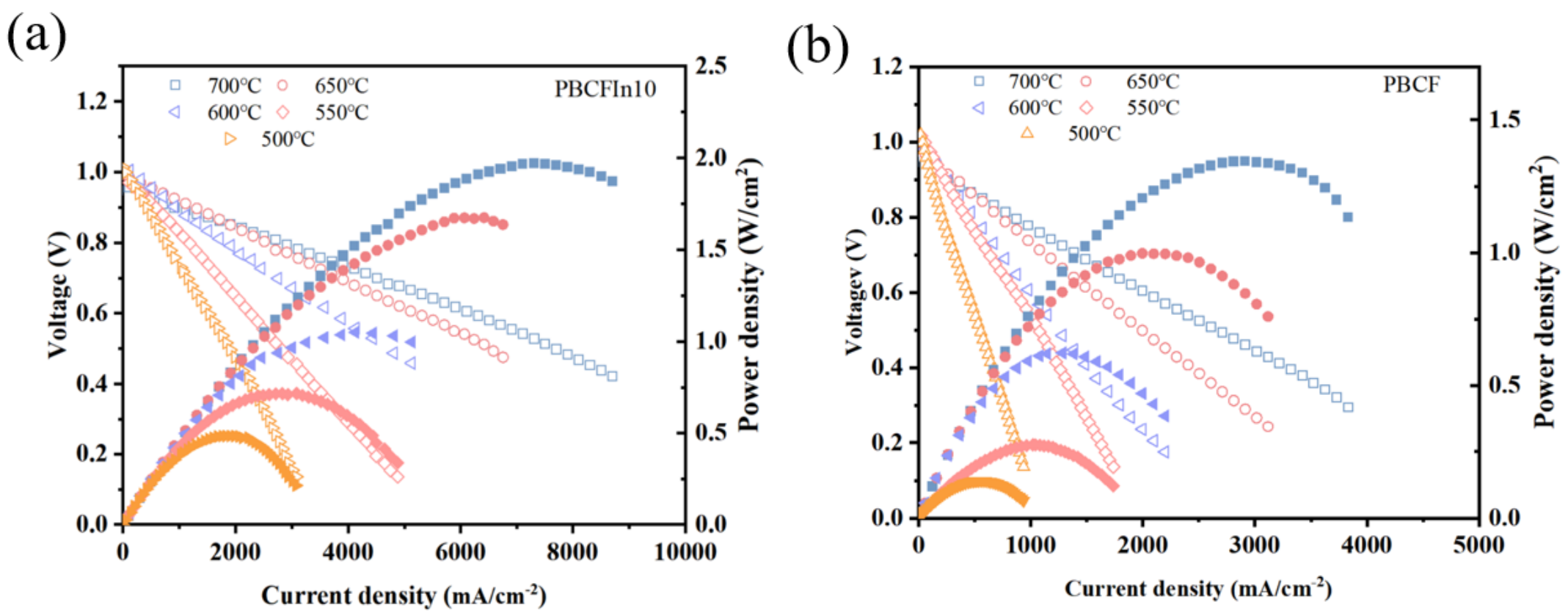

Supplementary Fig. 21. I-V and I-P curves of a BZCYYb-supported single cell with PBCF and PBCFIn10 air electrodes in FC mode at 500-700 °C. (a) The peak power densities (PPDs) of the single cell with the PBCFIn10 air electrode are 0.48, 0.71, 1.20, 1.67, and 1.97 W·cm^{-2} at 500–700 °C, respectively. (b) The PPDs of the single cell with the PBCF air electrode are 0.14, 0.28, 0.62, 1.01, and 1.34 W·cm^{-2} at at 500–700 °C, respectively. Notably, the PBCFIn10 electrode achieves ~47% higher PPD at 700 °C compared with pristine PBCF, and this enhancement is consistently observed across the measured temperature range, highlighting the beneficial role of In doping in promoting electrode reaction kinetics. By comparison, the PBCFIn05-based cell (Figure 5f) delivers much higher PPDs of 0.79, 1.21, 1.58, 1.95, and 2.47 W·cm^{-2} at at 500–700 °C, corresponding to improvements of ~82%, ~76%, ~155%, ~93%, and ~84% over pristine PBCF, respectively. These results indicate that moderate In doping (In05) is more effective than excessive doping (In10), underscoring the optimized defect engineering and superior catalytic activity of PBCFIn05.

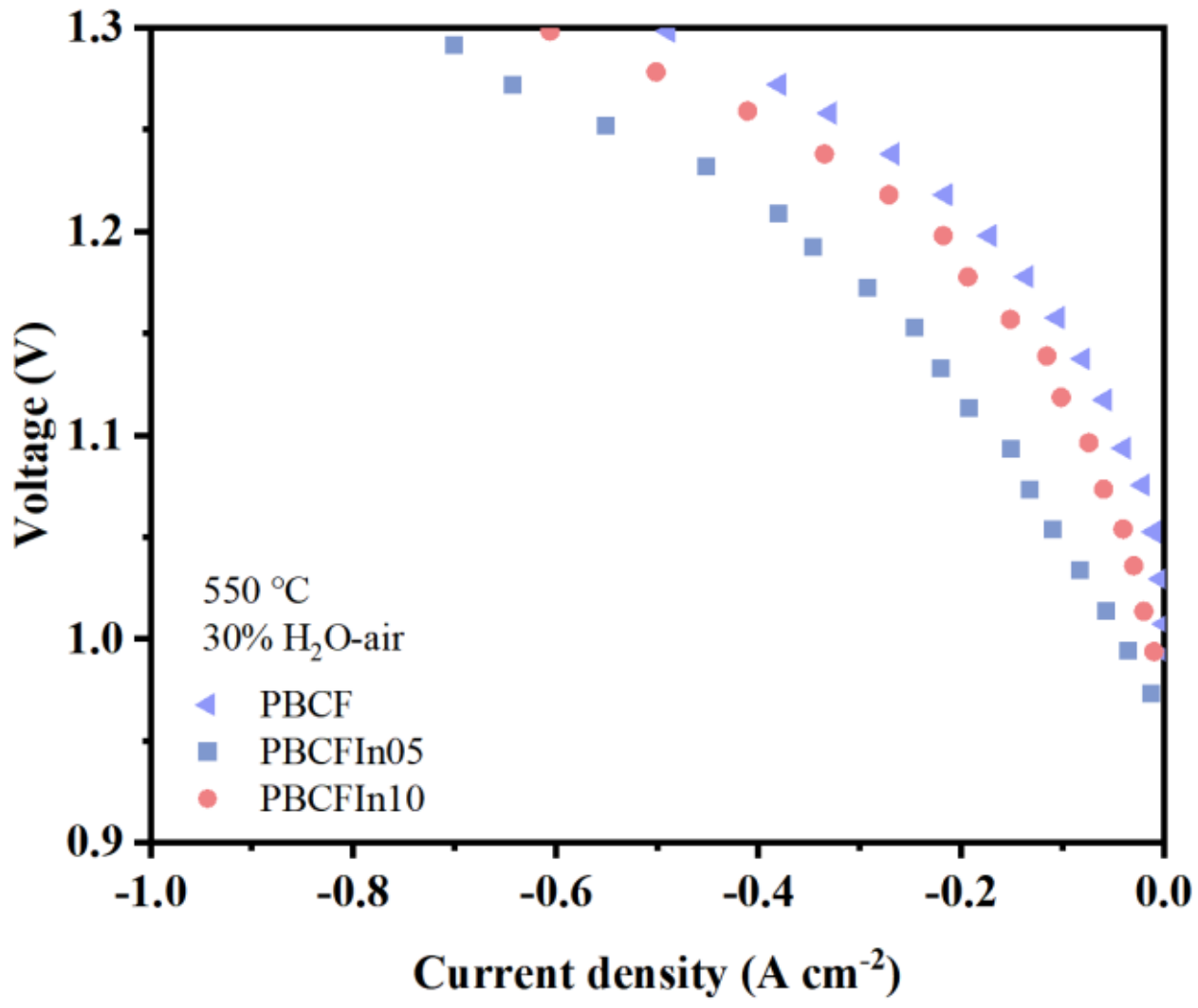

Supplementary Fig. 23. Electrochemical performance of BZCYYb-supported single cells with PBCF, PBCFIn05, and PBCFIn10 air electrodes in electrolysis cell (EC) mode at 550 °C with 30% H_2O–air. The current densities at 1.3 V are 0.71, 0.60, and 0.48, A·cm^{-2} for PBCFIn05, PBCFIn10, and PBCF cells, respectively, confirming the superior electrolysis activity of the moderately doped PBCFIn05 electrode.

Comment #5:

In Fig. 5j, the stability test in fuel cell (FC) mode appears to have been conducted under an excessively low applied bias, which limits the significance of the stability evaluation. Moreover, although the authors mentioned that the initial minor performance decay observed in FC mode may stem from transient surface-site deactivation or interfacial reaction kinetics, the performance in fact shows a monotonic decline rather than a transient drop followed by stabilization. This behavior suggests the presence of a more persistent degradation process. However, no post-analysis is provided to determine whether the decay originates from interfacial degradation, electrode surface evolution, or other factors.

Response: We thank the reviewer for this insightful comment regarding the stability evaluation in fuel cell (FC) mode.

Below we respond your comment one by one:

1) *"In Fig. 5j, the stability test in fuel cell (FC) mode appears to have been conducted under an excessively low applied bias, which limits the significance of the*

stability evaluation."

We acknowledge that the applied current density used in the initial FC stability test (0.2 A cm^{-2}) represents a relatively mild operating condition, which may limit the stringency of the durability assessment. This operating condition was originally selected based on prior studies that employed moderate current densities for preliminary stability evaluation (Xu et al., *Journal of Advanced Ceramics*, 2022, 11, 1849–1859; Tang et al., *Advanced Functional Materials*, 2025, 35, 2501995). However, we agree with the reviewer that stability testing under higher applied bias provides a more rigorous and scientifically meaningful assessment for air electrodes intended for R-PCEC operation. In response and following stability-testing conditions commonly adopted in recent literature, we have conducted additional long-term FC stability experiments under a higher and more representative applied current density. The corresponding results have now been added to the revised manuscript.

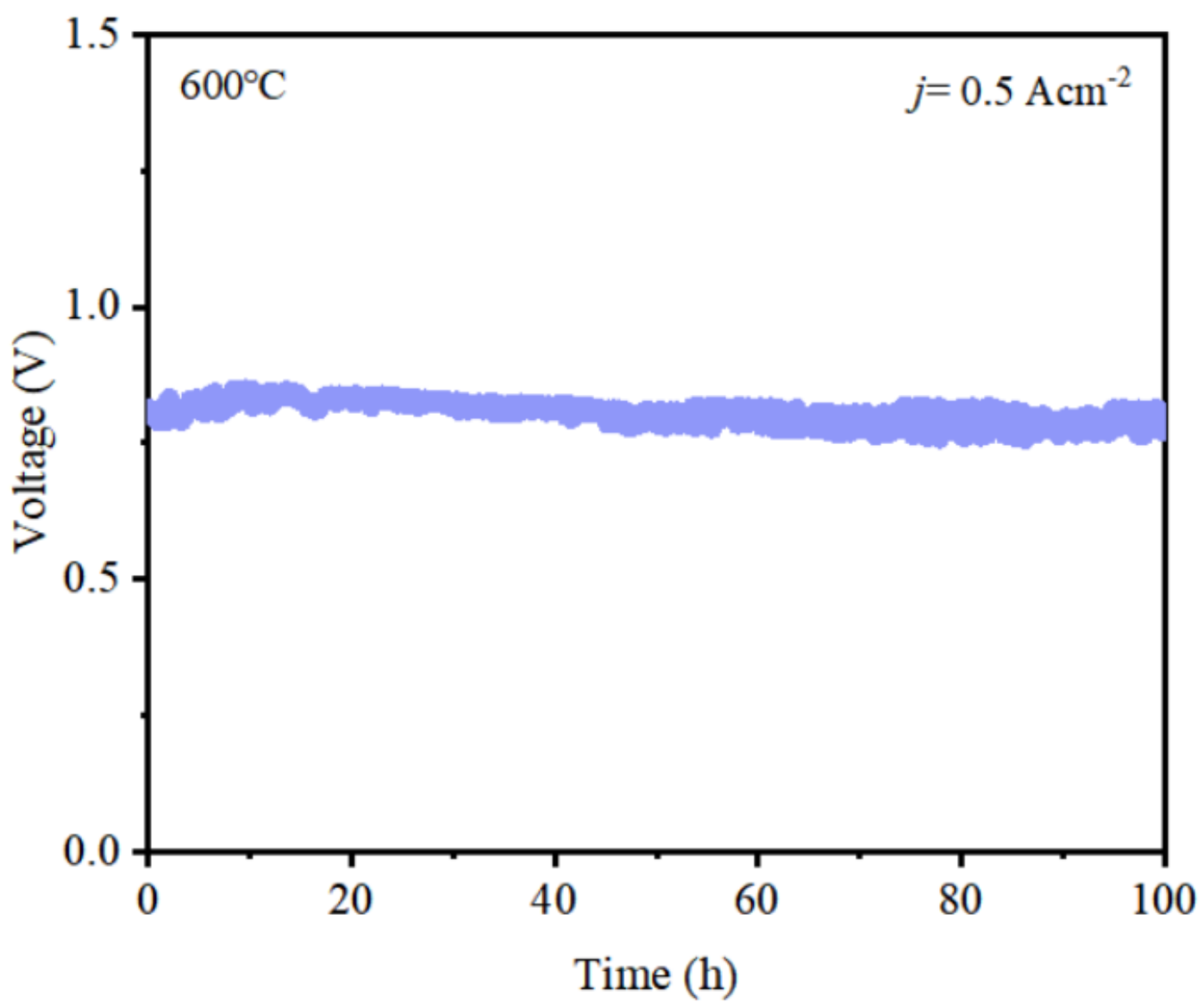

Fig. 5J. A short durability assessment of a PCFCIn05 at a consistent current density of 0.5A cm^{-2} and a temperature of 600 °C.

2) *"Although the authors mentioned that the initial minor performance decay observed in FC mode may stem from transient surface-site deactivation or interfacial reaction kinetics, the performance in fact shows a monotonic decline rather than a transient drop followed by stabilization."*

We also agree with the reviewer that the performance evolution shown in Fig. 5j exhibits a monotonic decline rather than a transient drop followed by stabilization. Therefore, the previous interpretation attributing the initial performance decrease to transient surface-site deactivation was inappropriate. In the revised manuscript, we have corrected this description and now discuss the observed behavior as a gradual and persistent degradation process.

The revised text has been added on page 15, lines 494.

"...Electrochemical stability was further examined through short-term durability tests conducted at 0.5 A cm^{-2} for 100 h at 600 °C (Fig. 5j). Under these conditions, the cell exhibited a gradual and monotonic voltage decay without abrupt performance loss, indicating reasonably stable operation over the examined time scale. The observed mild degradation behavior is consistent with a persistent but slow interfacial or surface evolution process, rather than transient surface-site deactivation. Post-test SEM analyses reveal no pronounced morphological, structural, or chemical changes Supplementary Fig. 25)"

3) *"no post-analysis is provided to determine whether the decay originates from interfacial degradation, electrode surface evolution, or other factors."*

We thank the reviewer for pointing out the lack of post-analysis in the initial version of the manuscript. To further investigate the origin of the performance decay, we have performed post-operation characterizations of the air electrode after prolonged FC operation, including SEM analysis. The post-test SEM images reveal no obvious morphological degradation, particle coarsening, or interfacial delamination compared with the pristine electrode, indicating that the electrode microstructure remains largely preserved during operation.

The revised text has been added on **page 15, lines 494.**

"...Electrochemical stability was further examined through short-term durability tests conducted at 0.5 A cm^{-2} for 100 h at 600 °C (Fig. 5j). Under these conditions, the cell exhibited a gradual and monotonic voltage decay without abrupt performance loss,

indicating reasonably stable operation over the examined time scale. The observed mild degradation behavior is consistent with a persistent but slow interfacial or surface evolution process, rather than transient surface-site deactivation. Post-test SEM analyses reveal no pronounced morphological, structural, or chemical changes Supplementary Fig. 25)”

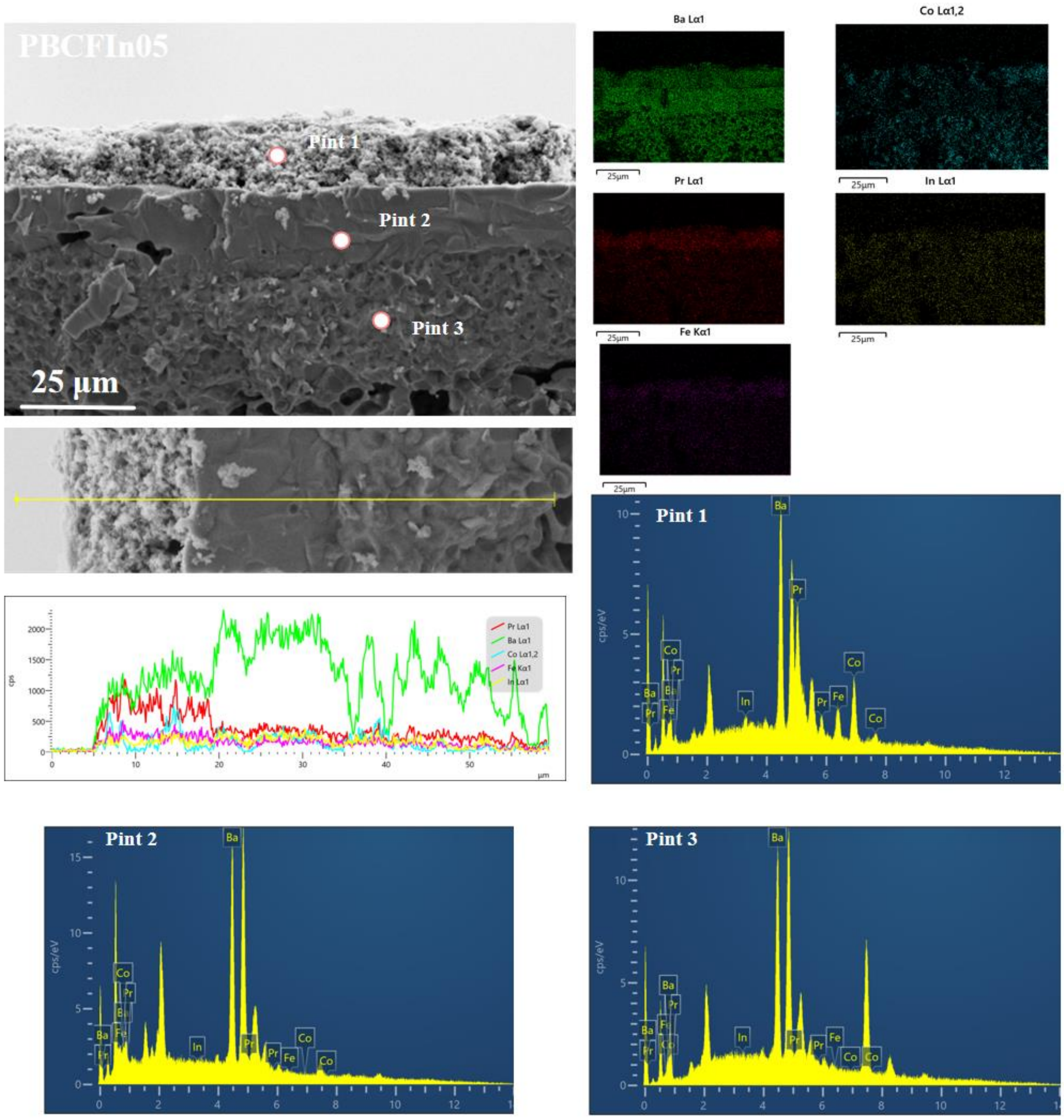

Supplementary Fig. 24. SEM image of PBCFIn05 single cell structure after tested.

Comment #6:

In Fig. 5k, the authors report thermal cycling durability tests under three distinct heating–cooling protocols; however, the corresponding temperature ranges are not specified, rendering the experimental conditions unclear and the results difficult to

interpret. Moreover, thermal cycling durability is not particularly relevant to the main scope of this study. Given that the proposed air electrode is intended for use in a reversible protonic ceramic electrochemical cell (R-PCEC), stability evaluations under electrolysis mode and reversible (fuel cell/electrolysis) operation would be far more appropriate and scientifically meaningful.

Response: We thank the reviewer for this helpful comment. We acknowledge that the temperature ranges corresponding to the three thermal cycling protocols in Fig. 5k were not clearly specified in the previous version of the manuscript, which could make the experimental conditions difficult to interpret. In the revised manuscript, we have explicitly added the corresponding heating–cooling temperature ranges to clearly define the testing conditions.

We also agree with the reviewer that thermal cycling durability is not central to the main scope of this study. Since the proposed air electrode is intended for application in R-PCECs, stability evaluations under electrolysis mode and reversible (fuel cell/electrolysis) operation are more directly relevant. Accordingly, in the revised manuscript we have emphasized the stability results obtained under electrolysis and reversible operation. The discussion has also been revised to clarify that the thermal cycling test is provided only as supplementary information rather than as a primary metric for evaluating electrode durability.

The revised text has been marked in red in the manuscript, and the corresponding discussion has been added on page 15, line 510.

"...The durability under reversible operation was further evaluated by galvanostatic current cycling between ±0.5 A cm^{-2} for 100 h at 600 °C with periodic switching between FC and EC modes (Fig. 5k). The R-PCEC employing the PBCFIn05 electrode exhibits excellent reversibility and tolerance during prolonged bidirectional cycling."

"...Thermal cycling durability was subsequently evaluated under three distinct heating-cooling protocols between 700 and 600 °C (Supplementary Fig. 26). In Mode 1, the cell experienced five moderate thermal cycles at a rate of 1.6 °C min^{-1} to

simulate fatigue conditions. In Modes 2 and 3, more aggressive cycling profiles were applied (heating at 10 and 20 °C min^{-1}; cooling at 5 and 10 °C min^{-1}) for up to 65 cycles. Remarkably, the PBCFIn05 air electrode maintained nearly constant current output with negligible degradation across all thermal stress tests, confirming its exceptional thermomechanical resilience.”

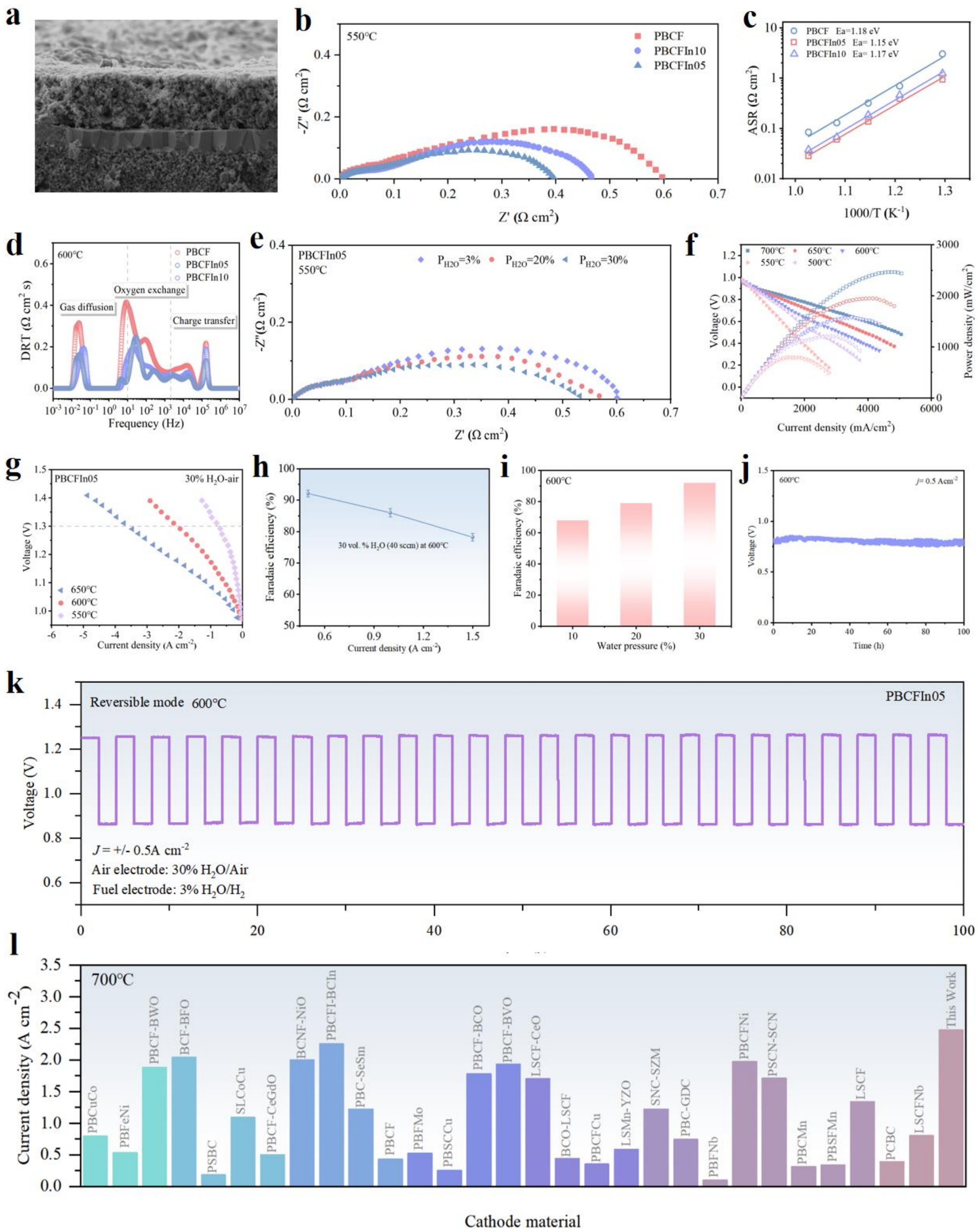

Fig. 5. Electrochemical performance and stability of single cells in fuel-cell (FC) mode. a Cross-sectional SEM image of PBCFIn05 single cell structure; b EIS plots of BZCYYb-supported single cell with PBCFIn05 electrode at 550 °C under 3%H_2O–H_2;

c Arrhenius plot of the ASRs for PBCF, PBCFIn05 and PBCFI10; d The DRT analysis of EIS was measured at PBCF, PBCFIn05, and PBCFIn10 electrodes at 600 °C; e EIS plots of cell with PBCFIn05 air electrode tested at 550 °C under 3, 10, 30%H_2O–air; f I–V and I–P curves of an BZCYYb-supported single cell with PBCFIn05 air electrode in FC mode at 500–700 °C; g I−V curves of cells with PBCFIn05 electrode measured in EC mode at 650–550 °C under 30% H_2O–air; h FE of cells at different current densities; i FE of cells at different P_{H2O}; j A short durability assessment of a PCFCIn05 at a consistent current density of 0.5 A cm^{-2} and a temperature of 600 °C; k a stability test of PBCFIn05 in ±0.5 A cm^{-2} for 100 h at 600°C; l Comparison of PDDs obtained with PBCFIn05 electrode and advanced electrodes in the literature at different temperatures.

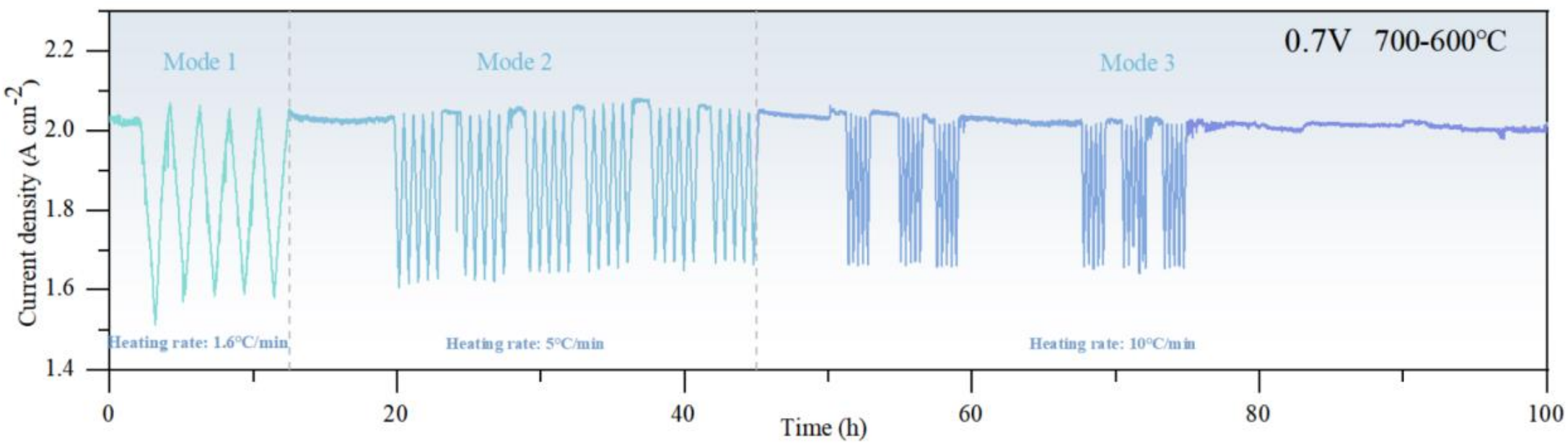

Supplementary Fig. 26 a stability test of PBCFIn05 in 0.7 V at 700-600 °C.

Comment #7:

Recently, many studies have been published reporting dual-phase composite electrodes with concepts similar to the present work to enhance electrochemical performance. However, the literature cited for the electrochemical performance comparison (Fig. 5l, Supplementary Fig. 20, and Supplementary Tables 8 and 9) is largely outdated and does not include these more recent and conceptually related studies. As a result, the performance benchmarking is incomplete and weakens the credibility of the authors' performance claims.

Response: We thank the reviewer for this important comment. We acknowledge that the literature included in the original performance comparison was not sufficiently up to date and did not fully capture several recently reported dual-phase composite

electrodes and conceptually related strategies. In response, we have comprehensively updated the benchmarking analysis by incorporating several recent studies published in the past few years that are directly relevant to the present work.

Accordingly, the comparison figures (Fig. 5l and Supplementary Fig. 24) as well as Supplementary Tables 10 and 11 have been revised to include these additional references. These updates provide a more comprehensive and current performance context, thereby strengthening the credibility and completeness of the performance benchmarking presented in this study.

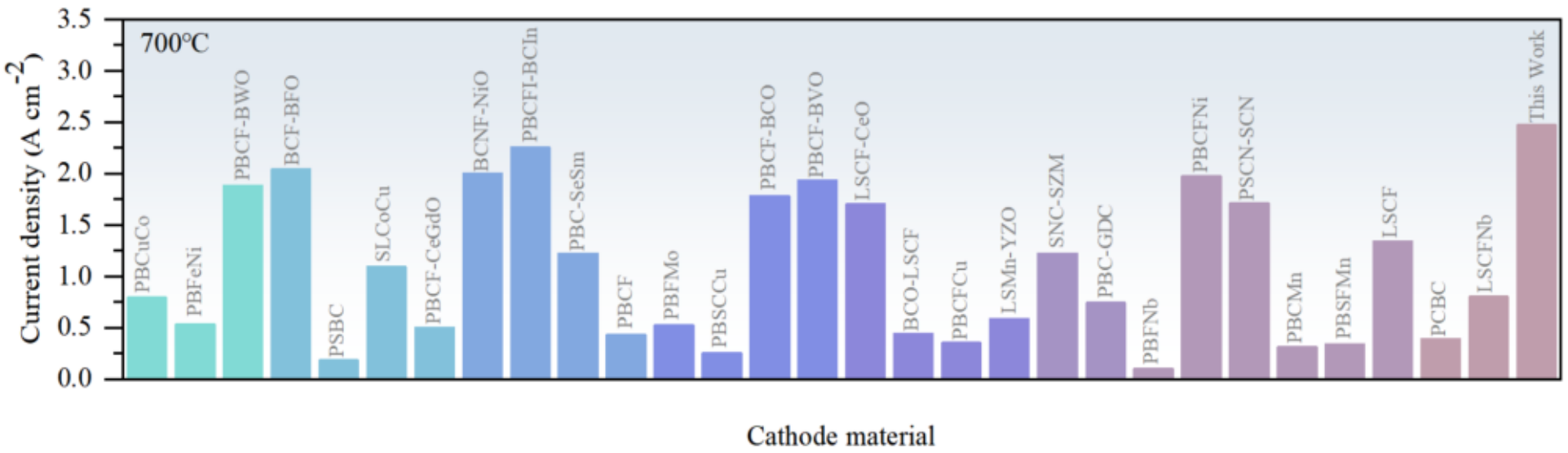

Fig. 5l. Comparison of PDDs obtained with PBCFIn05 electrode and advanced electrodes in the literature at different temperatures.

Supplementary Table 10: Performance (FC mode) comparison between PBCFIn05 and other air electrode materials.

**Air electrode**	**Temp. (°C)**	**Power density ($W \cdot cm^{-2}$)**	**Refs.**
PBCFIn05	700	2.47	This work
(PB)0.95FNb	700	0.10	[3]
PBSCCu	700	0.25	[5]
PBCMn	700	0.31	[17]
PBCFCu	700	0.35	[6]

PCBC	700	0.39	[9]
PBCF	700	0.43	[18]
PBC-GDC	700	0.74	[19]
PBC-SeSm	700	1.22	[20]
LSCF	700	1.34	[21]
PBCFNi	700	1.97	[22]
PBCuCo	700	0.79	[11]
BCO@LSCF	700	0.44	[12]
LSMn-YZO	700	0.58	[14]
LSCFNb	700	0.80	[23]
PBCF-CeGdO	700	0.50	[24]
PBFeNi	700	0.53	[25]
PBFMo	700	0.52	[26]
PBSFMn	700	0.34	[15]
SLCoCu	700	1.09	[16]
PSBC	700	0.18	[27]
PBCF-BWO	700	1.88	[28]

BCF-BFO	700	2.04	29
PCFCs-RuO	700	1.78	30
BCNF-NiO	700	2.0	31
PBCI-BCI	700	2.25	32
PBCFC-BCO	700	1.78	33
PBCF-BVO	700	1.93	34
LSCF-CeO	700	1.7	35
SNC-SZM	700	1.22	36
PSCN-SCN	700	1.71	37

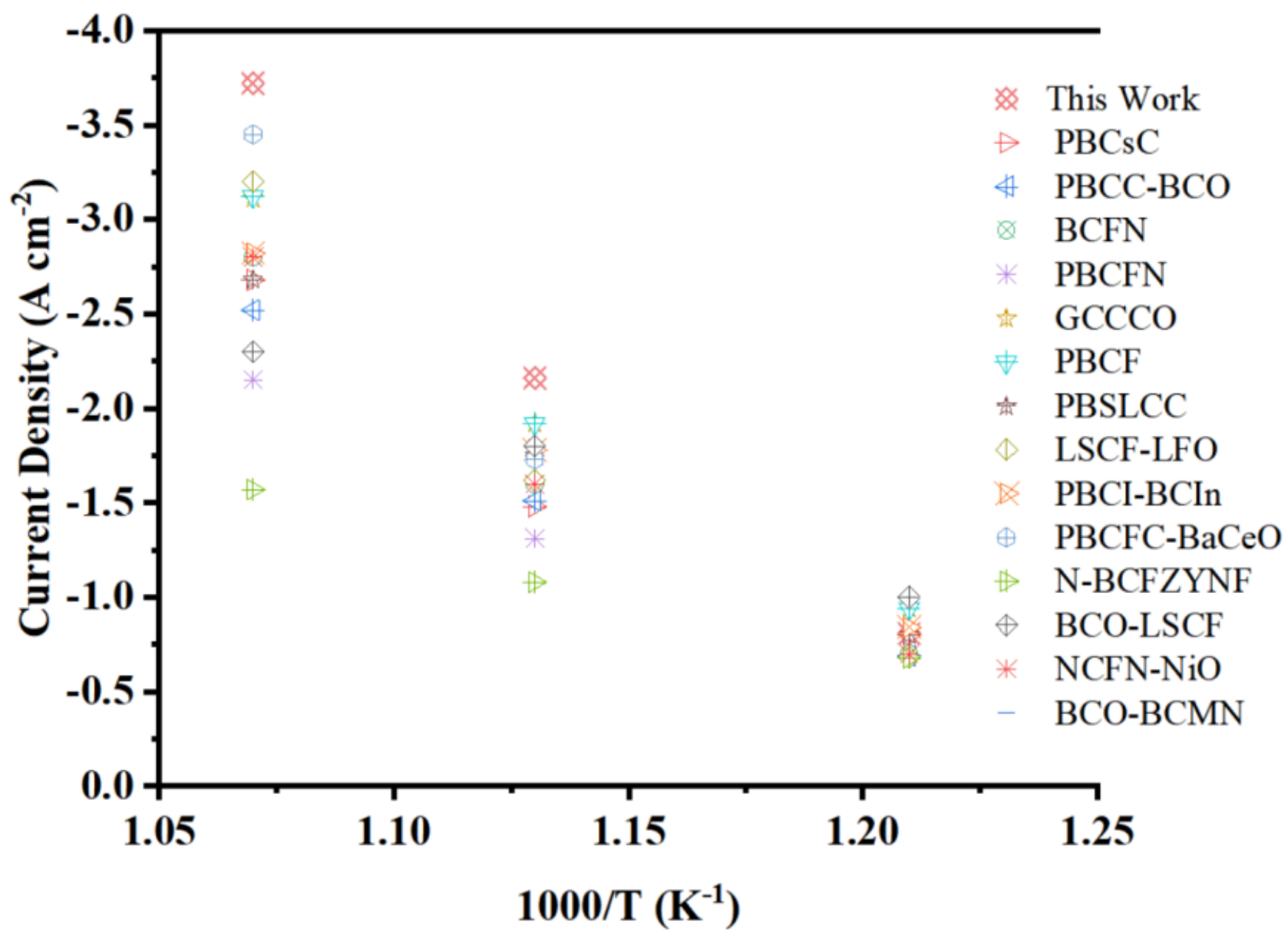

Supplementary Fig 24. Comparison of PDDs obtained with PBCFIn05 electrode and advanced electrodes in the literature at 1.3 V and 600 °C.

Supplementary Table 11: Performance (EC mode) comparison between PBCFIn05 and other air electrode materials.

Air electrode	Electrolyte	Temp.(°C)	Current density @ 1.3 V ($A\cdot cm^{-2}$)	Refs
PBCsC	BZCYYb	650	-2.68	[38]
		600	-1.48	
		550	-0.80	
PBCC-BCO	BZCYYb	650	-2.52	[39]
		600	-1.51	
		550	-0.69	
BCFN	BZCYYb	650	-2.80	[40]
		600	-1.60	
		550	-0.70	
PBCFN	BZCYYb	700	-3.85	[41]
		650	-2.15	
		600	-1.31	
GCCCO	BZCYYb	700	-4.49	[42]
		650	-3.11	
		600	-1.90	
PBCF	BZCYYb	700	-3.12	[33]
		650	-1.92	
		600	-0.94	

		650	-2.68	
PBSLCC	BZCYYb	600	-1.75	43
		550	-0.80	
		650	-3.2	
LSCF-LFO	BZCYYb	600	-1.62	35
		550	-0.68	
		650	-2.82	
PBCI-BCIn	BZCYYb	600	-1.78	44
		550	-0.84	
		650	-3.45	
PBCFC-BaCeO	BZCYYb	600	-1.73	45
		550	-	
		650	-1.57	
N-BCFZYNF	BZCYYb	600	-1.08	46
		550	-0.68	
		650	-2.3	
BCO-LSCF	BZCYYb	600	-1.8	47
		550	-1.0	
		650	-2.8	
NCFN-NiO	BZCYYb	600	-1.6	48
		550	-0.7	
BCO-BCMN	BZCYYb	650	2.04	49
		600	1.34	

		550	0.68	
		650	-3.72	
PBCFIn05	BZCYYb	600	-2.16	This Work
		550	-0.81	

Minor points:

1. The chemical compatibility with the electrolyte has not been evaluated in this study.

2. There are many typos and errors throughout the manuscript, including the supporting information, which need to be carefully checked and corrected.

Response: We thank the reviewer for these helpful suggestions.

Below we respond your comment one by one:

1) *"The chemical compatibility with the electrolyte has not been evaluated in this study."*

To evaluate the chemical compatibility between the PBCFIn05 air electrode and the BZCYYb electrolyte, a mixture of the two powders was heated at 900 °C for 3 h in air and subsequently analyzed by X-ray diffraction. As shown in the newly added Supplementary Fig. 29, no additional impurity peaks were detected after heat treatment, indicating that PBCFIn05 is chemically compatible with the BZCYYb electrolyte under the typical cell fabrication conditions.

The revised text has been marked in red in the revised manuscript, and the corresponding discussion has been added on **page 15, lines 520.**

"...As shown in Supplementary Fig. 29, the diffraction peaks can be indexed to the PBCFIn05 and BZCYYb phases without detectable impurity phases after heat treatment, indicating good chemical compatibility between the electrode and electrolyte under typical cell fabrication conditions."

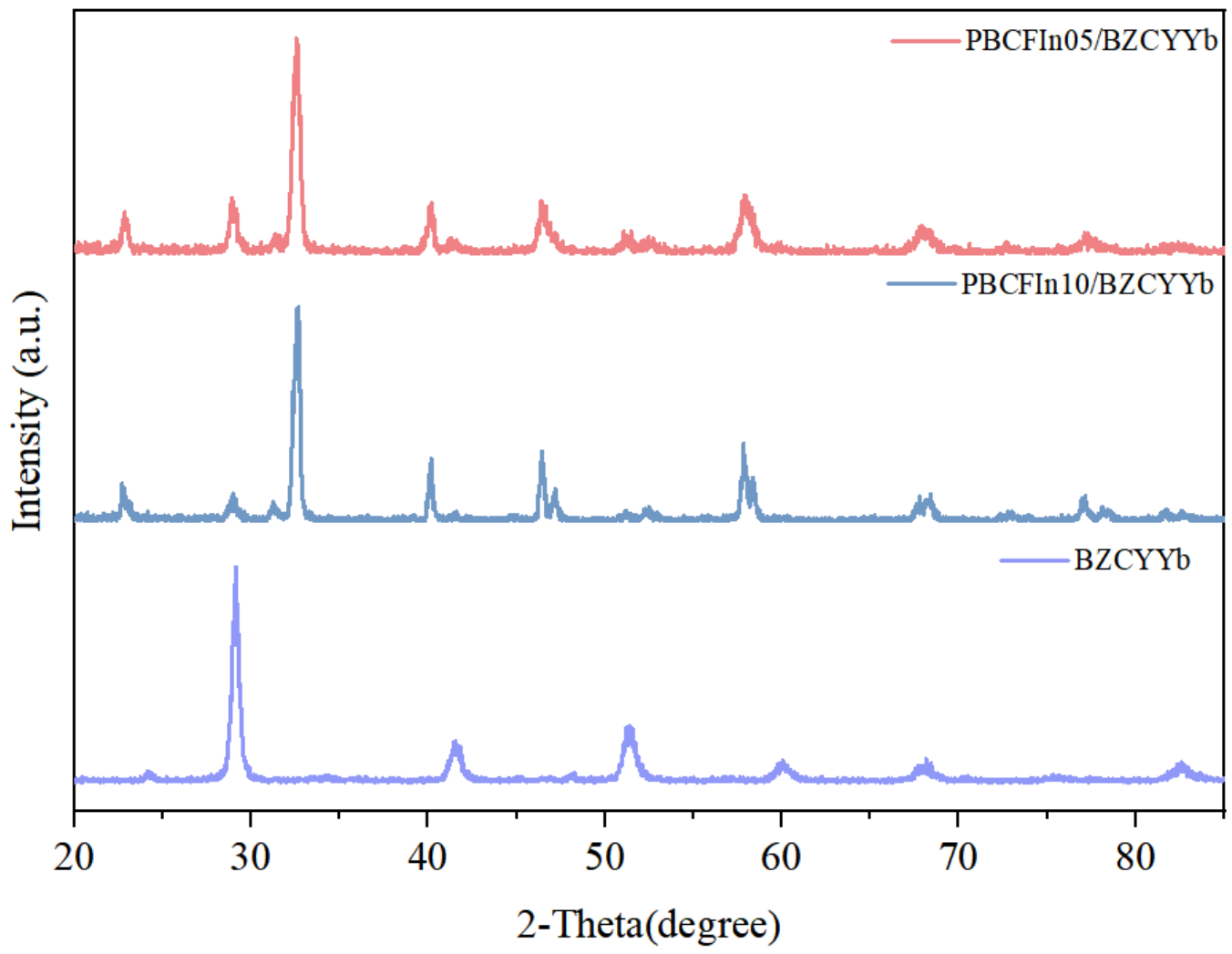

Supplementary Fig. 29. XRD patterns of the PBCFIn05/BZCYYb, PBCFIn10/BZCYYb and BZCYYb.

2)*"There are many typos and errors throughout the manuscript, including the supporting information, which need to be carefully checked and corrected."*

We have carefully rechecked the entire manuscript, including the Supporting Information, and corrected typographical errors, formatting issues, and minor language inconsistencies throughout the text.

Point-to-point Responses to Reviewers' Comments and Suggestions

We sincerely thank the Editor and the reviewers for their careful evaluation of our manuscript (NCOMMS-25-93329) and for the constructive comments and suggestions. We greatly appreciate the reviewers' recognition of the substantial improvements made in the revised manuscript. The insightful remarks have further helped us improve the clarity and rigor of the DFT analysis and the overall presentation of the manuscript. We have carefully revised the manuscript to address all comments. All changes are highlighted in red in the revised version, and detailed point-by-point responses are provided below.

Reviewer # 1 (Remarks to the Author): Accept.

Response: We sincerely thank the reviewer for the positive evaluation of our revised manuscript and for recommending acceptance. We greatly appreciate the reviewer's time and constructive feedback throughout the review process, which helped improve the quality of our work.

Reviewer # 3 (Remarks to the Author): The current manuscript is suitable for publication

Response: We sincerely thank Reviewer #3 for the positive assessment of our revised manuscript and for considering it suitable for publication. We greatly appreciate the reviewer's constructive comments and valuable suggestions, which helped us improve the clarity and quality of the manuscript.

Reviewer # 2

Comment #1:

*The authors have made substantial efforts to address the reviewers' comments, and most issues have been clarified to the extent possible. However, the section on the energy profile and DFT analysis still requires further revision, as the current presentation is insufficient. The energy diagram shown in Fig. 6e appears schematic and mixes thermodynamic free energies with activation barriers obtained from CI-NEB calculations. In addition, several intermediates (*O and *OO) and the symmetric*

ORR/OER pathway raise concerns about the physical validity of the proposed reaction mechanism. The authors should clearly define the reference states used in the energy calculations and present the CI-NEB results (i.e., the activation barriers) separately from the thermodynamic energy diagram, for example, by explicitly identifying the transition states (e.g., TS1 and TS2). Furthermore, although reversibility between OER and ORR should, in principle, be consistent with statistical mechanics, such symmetry is generally difficult to reproduce accurately in practical DFT calculations. The current representation in Fig. 6e, therefore, appears somewhat artificial and suggests that the energy diagram may have been constructed schematically rather than being directly derived from DFT-based energetics.The authors are encouraged to reconstruct the energy profiles by treating OER and ORR separately and to move the detailed energy diagrams to the Supporting Information in order to avoid potential misunderstandings. It is noted that numerous relevant publications are available in the literature. The discussion of this part in the main text should also be minimized unless the methodology and its physical interpretation are clearly justified.

Response: We sincerely thank the reviewer for the careful evaluation and constructive comments on the DFT energy-profile analysis. We agree that the previous presentation of Fig. 6e was insufficiently clear and could lead to misunderstanding, because thermodynamic intermediate energetics and CI-NEB-derived activation barriers were not clearly distinguished. We also acknowledge that presenting the ORR/OER pathway in a nearly symmetric manner may give the impression of a schematic construction rather than a directly calculated energy profile. In response, we have substantially revised this section.

Below, we respond to the reviewer’s comments point by point:

(1) The energy diagram shown in Fig. 6e appears schematic and mixes thermodynamic free energies with activation barriers obtained from CI-NEB calculations. The authors should clearly define the reference states used in the energy calculations and present the CI-NEB results (i.e., the activation barriers) separately from the thermodynamic energy diagram, for example, by explicitly identifying the transition states (e.g., TS1 and TS2).

Response: We thank the reviewer for pointing out this important issue. We agree that

the previous Fig. 6e did not clearly distinguish between thermodynamic intermediate energetics and CI-NEB-derived activation barriers, which could make the figure appear schematic and lead to ambiguity in the physical meaning of the plotted energy values.In the revised manuscript, we have separated these two types of energetic information. The thermodynamic energy diagram is now presented independently, with all intermediate-state energies referenced to the initial state of the corresponding reaction pathway. The CI-NEB results are presented separately as kinetic activation barriers, and the corresponding transition states have been explicitly labeled as TS1 and TS2.

This revision clearly distinguishes thermodynamic energy differences from kinetic activation barriers and avoids mixing different physical quantities in a single energy diagram. The corresponding figures and descriptions have been revised accordingly.

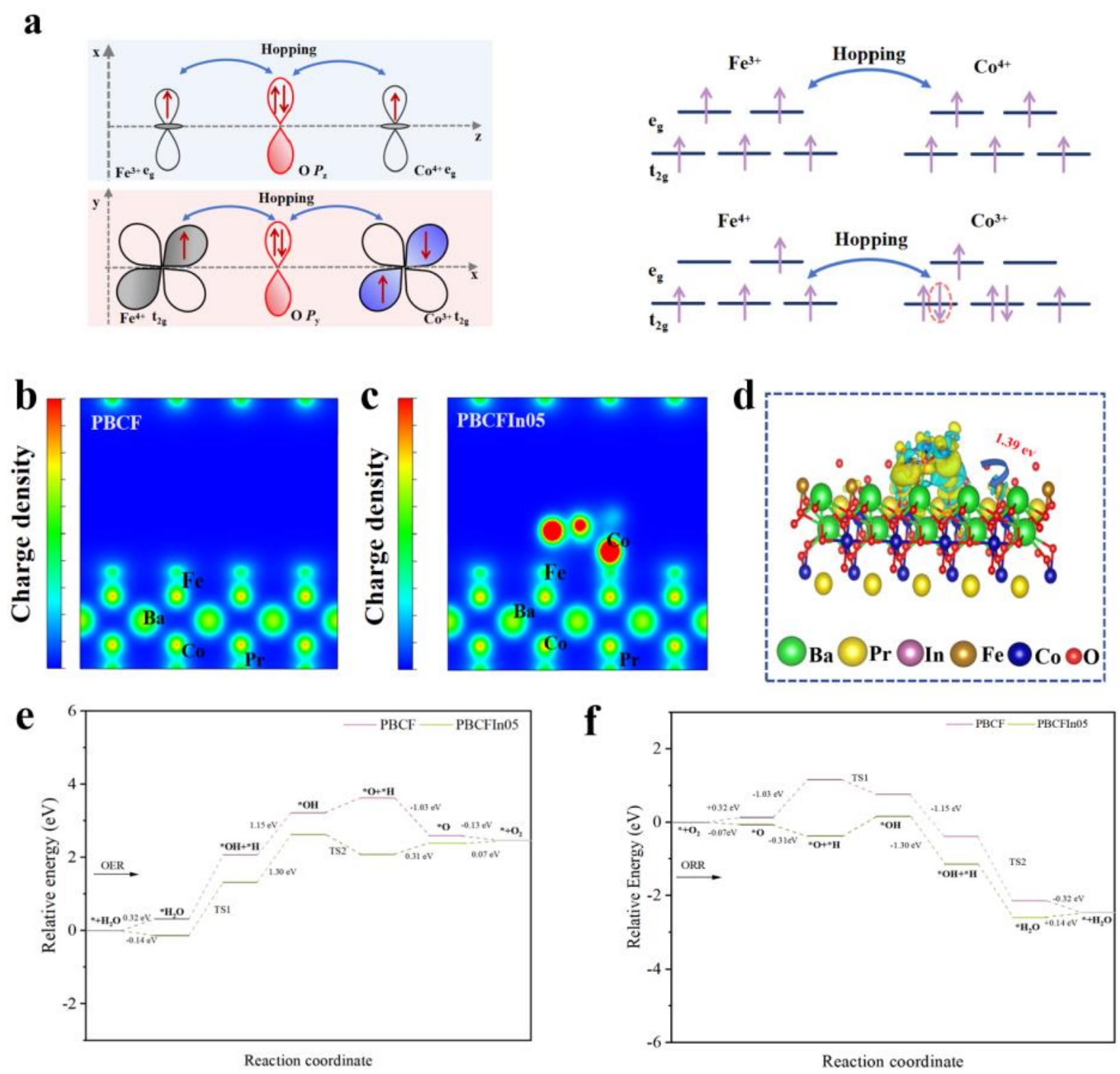

Fig. 6: e,f Calculated free-energy profiles of the OER and ORR on PBCF and PBCFIn05.

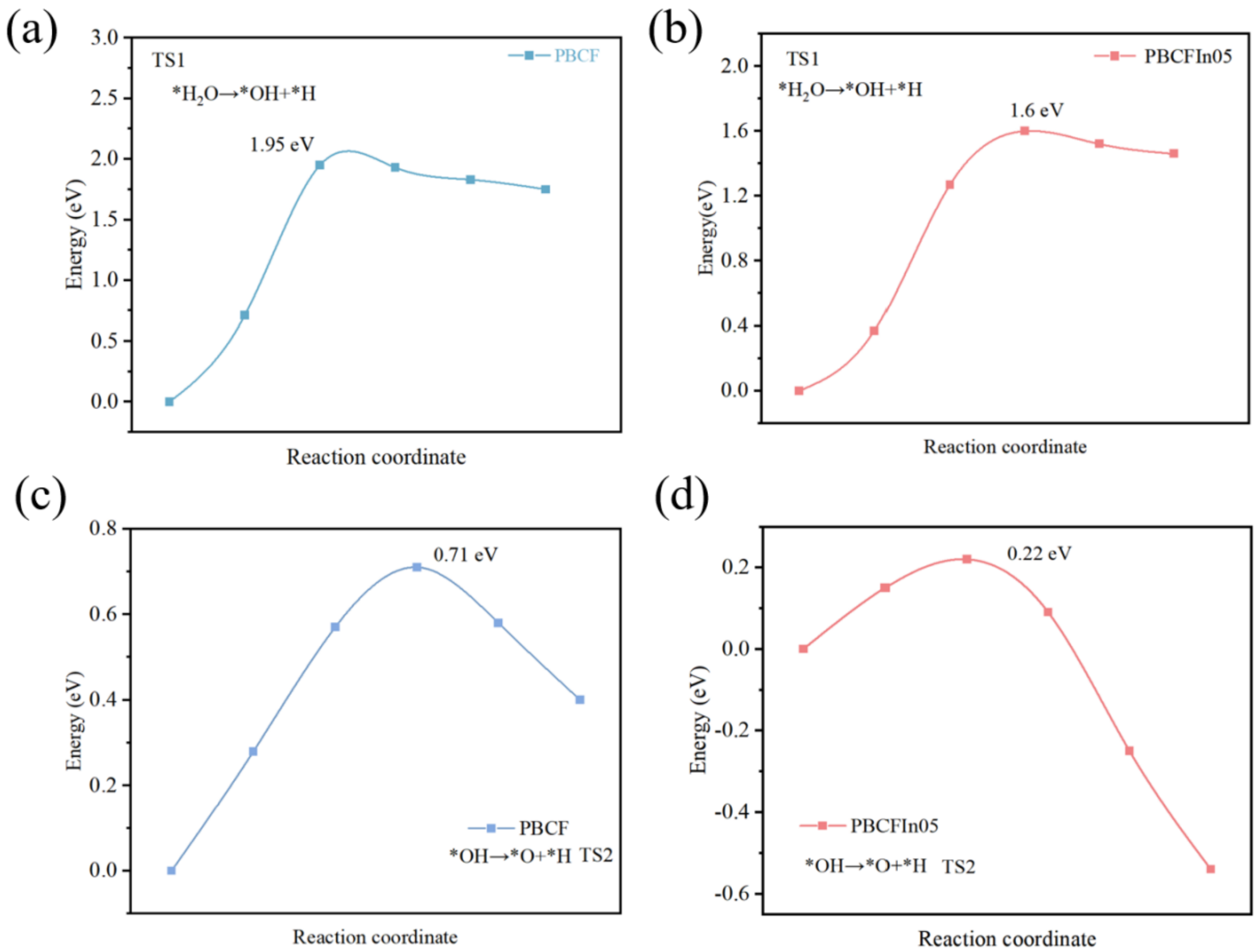

Supplementary Fig. 34. Calculated energy profiles for OER stepson PBCF and PBCFIn05. （a,b）CI-NEB energy profiles for $*H_2O \rightarrow *OH+*H$; (c,d) CI-NEB energy profiles for $*OH \rightarrow *O+*H$.

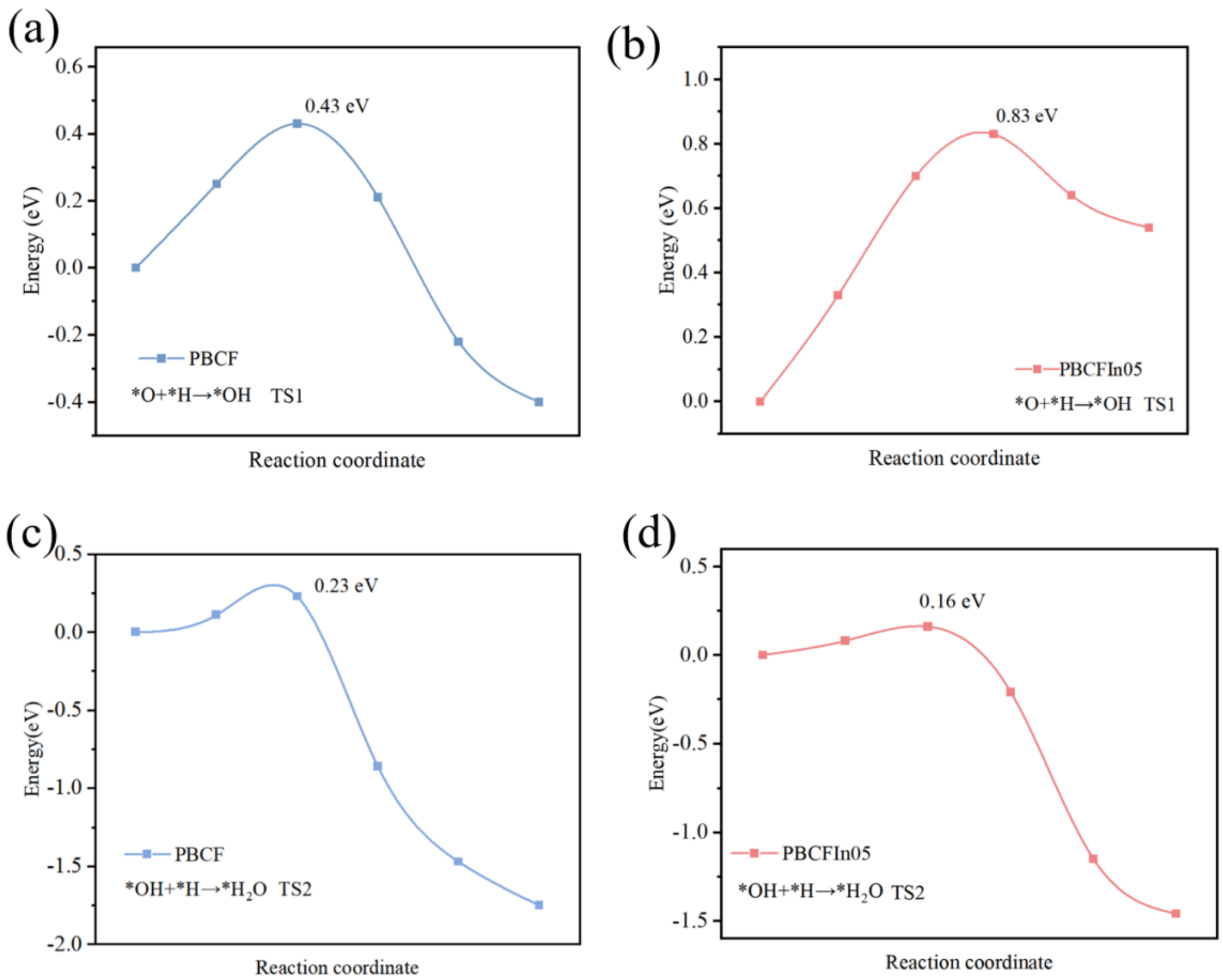

Supplementary Fig. 35. Calculated energy profiles for ORR stepson PBCF and PBCFIn05. （a,b）CI-NEB energy profiles for *O+*H→*OH ; (c,d) CI-NEB energy profiles for *OH+*H→H_2O.

(2) Furthermore, although reversibility between OER and ORR should, in principle, be consistent with statistical mechanics, such symmetry is generally difficult to reproduce accurately in practical DFT calculations. The current representation in Fig. 6e, therefore, appears somewhat artificial and suggests that the energy diagram may have been constructed schematically rather than being directly derived from DFT-based energetics.The authors are encouraged to reconstruct the energy profiles by treating OER and ORR separately and to move the detailed energy diagrams to the Supporting Information in order to avoid potential misunderstandings.The reviewer suggested reconstructing the energy profiles by treating OER and ORR separately and moving the detailed energy diagrams to the Supporting Information to avoid potential misunderstandings.

Response: We thank the reviewer for this insightful comment.We thank the reviewer for this insightful comment. We agree that the previous symmetric representation of the ORR/OER pathway in Fig. 6e could appear artificial, since practical DFT calculations generally do not reproduce perfectly mirrored forward and reverse reaction profiles. In the revised manuscript, we have removed the symmetric ORR/OER depiction and treated the OER- and ORR-related energetics separately.

In the revised version, we have added the relative-energy axis, explicitly labeled all intermediate and transition-state energies, and defined the corresponding reference states. The detailed energy profiles are now provided in the Supporting Information to clarify that the plotted energy levels are derived from DFT-based calculations rather than schematic construction.

Supplementary Table 13 Total energies for OER TS1 on PBCF

Configuration names	Total energies (eV)
0	-553.76
1	-553.05
2	-551.81
3	-551.83
4	-551.93
5	-552.01

Supplementary Table 14 Total energies for OER TS2 on PBCF

Configuration names	Total energies(eV)
0	-553.16
1	-552.88
2	-552.59
3	-552.45
4	-552.58
5	-552.76

Supplementary Table 15 Total energies for OER TS1 on PBCFIn05

Configuration names	Total energies(eV)
0	-604.88

1	-604.51
2	-603.61
3	-603.28
4	-603.36
5	-603.42

Supplementary Table 16 Total energies for OER TS2 on PBCFIn05

Configuration names	Total energies(eV)
0	-602.27
1	-602.19
2	-602.05
3	-602.19
4	-602.57
5	-602.81

Supplementary Table 17 Total energies for ORR TS2 on PBCFIn05

Configuration names	Total energies(eV)
0	-552.01
1	-551.90
2	-551.78
3	-552.87
4	-553.48
5	-553.76

Supplementary Table 18 Total energies for ORR TS1 on PBCFIn05

Configuration names	Total energies(eV)
0	-552.76
1	-552.51
2	-552.33
3	-552.55
4	-552.98
5	-553.16

Supplementary Table 19 Total energies for ORR TS2 on PBCFIn05

Configuration names	Total energies(eV)
0	-603.42
1	-603.34

2	-603.26
3	-603.63
4	-604.57
5	-604.88

Supplementary Table 20 Total energies for ORR TS1 on PBCFIn05

Configuration names	Total energies(eV)
0	-602.81
1	-602.48
2	-602.11
3	-601.98
4	-602.17
5	-602.27

(3) It is noted that numerous relevant publications are available in the literature. The discussion of this part in the main text should also be minimized unless the methodology and its physical interpretation are clearly justified.

Response: We thank the reviewer for this helpful suggestion. We agree that the discussion of the DFT energy-profile analysis in the main text should be minimized unless the methodology and physical interpretation are clearly justified. In the revised manuscript, we have shortened the main-text discussion related to the energy profiles. These revisions reduce the possibility of overinterpretation and improve the clarity and rigor of the computational discussion.

The revised text has been marked in red in the revised manuscript, and the corresponding discussion has been added on **page 17, lines574.**

"....To gain mechanistic insight into the oxygen reaction energetics on PBCF and PBCFIn05 surfaces, density functional theory (DFT) calculations were performed to evaluate surface intermediates and elementary reaction steps (Supplementary Fig. 30-33). The relative energies of selected surface intermediates and the CI-NEB-derived activation barriers were analyzed separately (Fig.6e-f). The OER and ORR related transition states are denoted as TS1 and TS2.. For the OER-related steps, CI-NEB calculations show that the activation barrier for water dissociation ∗$H_2O \rightarrow *OH+*H$ (TS1) decreases from 1.95 eV on pristine PBCF to 1.60 eV on PBCFIn05. The

*subsequent hydroxyl deprotonation step *OH→*O+*H (TS2) also exhibits a reduced barrier, decreasing from 0.71 eV on PBCF to 0.22 eV on PBCFIn05 (Supplementary Fig. 34 and Table S13-S16). The ORR-related CI-NEB results involving TS1 and TS2 are provided in the Supplementary Fig. 35 and Supplementary Tables 17-20. These results indicate that excess indium incorporation facilitates proton-transfer and deprotonation-related surface processes.”*

Point-to-point Responses to Reviewers' Comments and Suggestions

We sincerely thank the Editor and the reviewers for their careful evaluation of our manuscript (NCOMMS-25-93329) and for the constructive comments and suggestions. We greatly appreciate the reviewers' recognition of the substantial improvements made in the revised manuscript. The insightful remarks have further helped us improve the clarity and rigor of the DFT analysis and the overall presentation of the manuscript. We have carefully revised the manuscript to address all comments. All changes are highlighted in red in the revised version, and detailed point-by-point responses are provided below.

Reviewer # 1 (Remarks to the Author): Accept.

Response: We sincerely thank the reviewer for the positive evaluation of our revised manuscript and for recommending acceptance. We greatly appreciate the reviewer's time and constructive feedback throughout the review process, which helped improve the quality of our work.

Reviewer # 3 (Remarks to the Author): The current manuscript is suitable for publication

Response: We sincerely thank Reviewer #3 for the positive assessment of our revised manuscript and for considering it suitable for publication. We greatly appreciate the reviewer's constructive comments and valuable suggestions, which helped us improve the clarity and quality of the manuscript.

Reviewer # 2

Comment #1:

The authors have satisfactorily addressed the reviewers' comments, and the manuscript has been substantially improved. The revised DFT analysis is clearer, and the mechanistic discussion is now adequately supported.

Only a minor clarification is recommended before final publication. The terminology used for the reported "energy profiles" should be carefully checked in both the main text and the computational methods. Based on the presented methodology, these

profiles appear to be relative DFT energy profiles rather than rigorous "free-energy profiles" obtained from a Computational Hydrogen Electrode (CHE) framework. Therefore, wording such as "free-energy profiles," including in the caption of Fig. 6, should be revised accordingly. In addition, statements such as "Thermodynamic feasibility was examined using the computational hydrogen electrode (CHE) framework to estimate Gibbs free energy changes" in the computational methods should be removed or revised after careful proofreading if CHE was not actually employed. The supplementary tables listing the calculated total energies for Supplementary Tables 13–20 should also be deleted, as they are not necessary for the revised presentation.

Overall, the manuscript is suitable for publication after these minor editorial and terminology corrections.

Response: We sincerely thank the reviewer for the positive assessment of the revised manuscript and for pointing out this important terminology issue.

Below, we respond to the reviewer' s comments point by point:

(1) Based on the presented methodology, these profiles appear to be relative DFT energy profiles rather than rigorous "free-energy profiles" obtained from a Computational Hydrogen Electrode (CHE) framework. Therefore, wording such as "free-energy profiles," including in the caption of Fig. 6, should be revised accordingly.

Response: We thank the reviewer for pointing out this important terminology issue. We agree that, based on the revised methodology, the reported profiles should be described as relative DFT energy profiles rather than rigorous free-energy profiles derived from a Computational Hydrogen Electrode (CHE) framework.

In the revised manuscript, we have carefully checked the main text, Fig. 6 caption, and Supporting Information, and have replaced "free-energy profiles" with "relative DFT energy profiles" or "relative energy profiles" where appropriate.

The revised text has been marked in red in the revised manuscript, and the corresponding discussion has been added on **page 17, lines572.**

"....**Fig. 6: e,f** Calculated relative DFT energy profiles of the OER and ORR on PBCF and PBCFIn05."

(2) In addition, statements such as "Thermodynamic feasibility was examined using the computational hydrogen electrode (CHE) framework to estimate Gibbs free energy changes" in the computational methods should be removed or revised after careful proofreading if CHE was not actually employed.

Response: We thank the reviewer for this helpful comment. We agree that the previous statement regarding the use of the computational hydrogen electrode (CHE) framework was not appropriate.In the revised Computational Methods section, we have removed this statement.

(3) The supplementary tables listing the calculated total energies for Supplementary Tables 13–20 should also be deleted, as they are not necessary for the revised presentation.

Response: We thank the reviewer for this helpful suggestion. Following the reviewer's recommendation, we have deleted the supplementary tables listing the calculated total energies for Supplementary Tables 13-20, as these data are not necessary for the revised presentation.

Point-to-point Responses to Reviewers' Comments and Suggestions

We sincerely thank the Editor and the reviewers for their careful evaluation of our manuscript (NCOMMS-25-93329) and for the constructive comments and suggestions. We greatly appreciate the reviewers' recognition of the substantial improvements made in the revised manuscript. The insightful remarks have further helped us improve the clarity and rigor of the DFT analysis and the overall presentation of the manuscript. We have carefully revised the manuscript to address all comments. All changes are highlighted in red in the revised version, and detailed point-by-point responses are provided below.

Reviewer # 1 (Remarks to the Author): Accept.

Response: We sincerely thank the reviewer for the positive evaluation of our revised manuscript and for recommending acceptance. We greatly appreciate the reviewer's time and constructive feedback throughout the review process, which helped improve the quality of our work.

Reviewer # 3 (Remarks to the Author): The current manuscript is suitable for publication

Response: We sincerely thank Reviewer #3 for the positive assessment of our revised manuscript and for considering it suitable for publication. We greatly appreciate the reviewer's constructive comments and valuable suggestions, which helped us improve the clarity and quality of the manuscript.

Reviewer # 2

Comment #1:

The manuscript is suitable for publication. As a minor editorial revision, the authors are encouraged to include the appropriate reference for the CI-NEB method used in the computational analysis.

Response: We sincerely thank the reviewer for the positive assessment of our manuscript and for recommending publication. In the revised manuscript, we have

added the appropriate citation for the climbing-image nudged elastic band method in the Computational Methods section to properly acknowledge the methodology used in the transition-state and activation-barrier calculations.

Added reference:

86. [Zhang S-M, Lu Y-Z, Liu Y-J, Li K, Yang W. First-principles study of oxygen conductivity in LSCF cathodes of solid oxide fuel cells. *Materials Today Communications* **42**, 111280 (2025).]

88. [Henkelman G, Uberuaga BP, Jónsson H. A climbing image nudged elastic band method for finding saddle points and minimum energy paths. *The Journal of chemical physics* **113**, 9901-9904 (2000).]
